# A randomized, double-blind, phase 2b proof-of-concept clinical trial in early Alzheimer’s disease with lecanemab, an anti-Aβ protofibril antibody

**DOI:** 10.1186/s13195-021-00813-8

**Published:** 2021-04-17

**Authors:** Chad J. Swanson, Yong Zhang, Shobha Dhadda, Jinping Wang, June Kaplow, Robert Y. K. Lai, Lars Lannfelt, Heather Bradley, Martin Rabe, Akihiko Koyama, Larisa Reyderman, Donald A. Berry, Scott Berry, Robert Gordon, Lynn D. Kramer, Jeffrey L. Cummings

**Affiliations:** 1 Eisai Inc., Woodcliff Lake, NJ USA; 2 Eisai Ltd., Hatfield, UK; 3 BioArctic AB, Warfvinges väg 35, SE-112 51 Stockholm, Sweden; 4 Department of Public Health/Geriatrics, Uppsala University, Uppsala, Sweden; 5 Berry Consultants, LLC, Austin, TX USA; 6 Chambers-Grundy Center for Transformative Neuroscience, Department of Brain Health, School of Integrated Health Sciences, University of Nevada Las Vegas, Las Vegas, NV USA

**Keywords:** Alzheimer’s disease, Amyloid, Lecanemab, BAN2401, Clinical trial, Biomarker, ADCOMS, Amyloid PET, Neurofilament light, Neurogranin, p-tau

## Abstract

**Background:**

Lecanemab (BAN2401), an IgG1 monoclonal antibody, preferentially targets soluble aggregated amyloid beta (Aβ), with activity across oligomers, protofibrils, and insoluble fibrils. BAN2401-G000-201, a randomized double-blind clinical trial, utilized a Bayesian design with response-adaptive randomization to assess 3 doses across 2 regimens of lecanemab versus placebo in early Alzheimer’s disease, mild cognitive impairment due to Alzheimer’s disease (AD) and mild AD dementia.

**Methods:**

BAN2401-G000-201 aimed to establish the effective dose 90% (ED90), defined as the simplest dose that achieves ≥90% of the maximum treatment effect. The primary endpoint was Bayesian analysis of 12-month clinical change on the Alzheimer’s Disease Composite Score (ADCOMS) for the ED90 dose, which required an 80% probability of ≥25% clinical reduction in decline versus placebo. Key secondary endpoints included 18-month Bayesian and frequentist analyses of brain amyloid reduction using positron emission tomography; clinical decline on ADCOMS, Clinical Dementia Rating-Sum-of-Boxes (CDR-SB), and Alzheimer’s Disease Assessment Scale-Cognitive Subscale (ADAS-Cog14); changes in CSF core biomarkers; and total hippocampal volume (HV) using volumetric magnetic resonance imaging.

**Results:**

A total of 854 randomized subjects were treated (lecanemab, 609; placebo, 245). At 12 months, the 10-mg/kg biweekly ED90 dose showed a 64% probability to be better than placebo by 25% on ADCOMS, which missed the 80% threshold for the primary outcome. At 18 months, 10-mg/kg biweekly lecanemab reduced brain amyloid (−0.306 SUVr units) while showing a drug-placebo difference in favor of active treatment by 27% and 30% on ADCOMS, 56% and 47% on ADAS-Cog14, and 33% and 26% on CDR-SB versus placebo according to Bayesian and frequentist analyses, respectively. CSF biomarkers were supportive of a treatment effect. Lecanemab was well-tolerated with 9.9% incidence of amyloid-related imaging abnormalities-edema/effusion at 10 mg/kg biweekly.

**Conclusions:**

BAN2401-G000-201 did not meet the 12-month primary endpoint. However, prespecified 18-month Bayesian and frequentist analyses demonstrated reduction in brain amyloid accompanied by a consistent reduction of clinical decline across several clinical and biomarker endpoints. A phase 3 study (Clarity AD) in early Alzheimer’s disease is underway.

**Trial registration:**

Clinical Trials.govNCT01767311.

**Supplementary Information:**

The online version contains supplementary material available at 10.1186/s13195-021-00813-8.

## Introduction

Alzheimer’s disease (AD) is a progressive neurodegenerative disease that slowly impairs cognition and function [1–3]. AD occurs on a continuum, progressing from asymptomatic preclinical AD, to mild cognitive impairment due to AD, and to mild, moderate, and severe AD dementia.

Amyloid β protein (Aβ) exists in various conformational states, including soluble monomers, soluble aggregates of increasing size (e.g., oligomers, protofibrils), and insoluble fibrils and plaque [4–6]. Soluble Aβ aggregates have been shown to be more toxic than monomers or insoluble fibrils [4, 6–10], and we hypothesized that reducing these soluble Aβ aggregates could represent an effective treatment approach in early stages of AD [4, 11, 12].

Lecanemab (BAN2401) is a humanized IgG1 monoclonal antibody that binds to soluble Aβ aggregates (oligomers and protofibrils) with high selectivity over monomer (> 1000-fold) and insoluble fibrils (approximately 10–15 fold) [13–16]. Reduction of Aβ protofibrils and Aβ plaque, as well as prevention of Aβ deposition before plaques develop, has been demonstrated using the murine version of lecanemab in animal models [12–14, 17, 18]. The antibody was well tolerated in a phase 1 study, with dose proportional exposure [19]. On the basis of these findings, a phase 2b proof-of-concept, dose-ranging efficacy study using a novel Bayesian adaptive design was initiated to assess the effects of lecanemab in subjects with mild cognitive impairment due to AD and mild AD dementia, collectively termed early Alzheimer’s disease.

BAN2401-G000-201 (Study 201) employed a Bayesian adaptive design with response adaptive randomization, involving frequent blinded interim analyses intended to assess for early success or futility, and designed to update subsequent subject allocation probabilities based on the predicted 12-month outcome modeled on all available clinical data on the Alzheimer’s Disease Composite Score (ADCOMS). In this design, a computer algorithm assessed accumulating ADCOMS data to allocate more subjects to a dose or doses that were most likely to be the ED90 target dose (defined as the simplest treatment group that achieves at least 90% of the modeled maximum treatment effect), while putting fewer subjects on less effective doses (NOTE: once subjects were randomized to a dose, they stayed on that dose for the duration of the study). Each interim analysis and the resulting update to subject allocation was implemented seamlessly while remaining completely blinded to subjects, sites, and the Sponsor. The choice of design was deemed a suitable approach to efficiently balance the desired potential for rapid decision-making and the need to establish clinical proof-of-concept [20]. The use of Bayesian methodology with a 12-month primary endpoint in this 18-month study was intended to afford the opportunity to move as early as possible into phase 3 if an early rigorous success criterion was met at any interim analysis, or to simply proceed per protocol to full randomization and study completion at 18 months if this condition was not met. Understanding the importance of 18-month data in the context of AD clinical trials assessing slowing of disease progression, all randomized subjects were to complete the full 18 months of treatment. The study was prospectively designed to analyze the 18-month results with Bayesian and frequentist (conventional) statistics regardless of whether the primary endpoint was met at 12 months.

## Methods

### Design

**Fig. 1:**
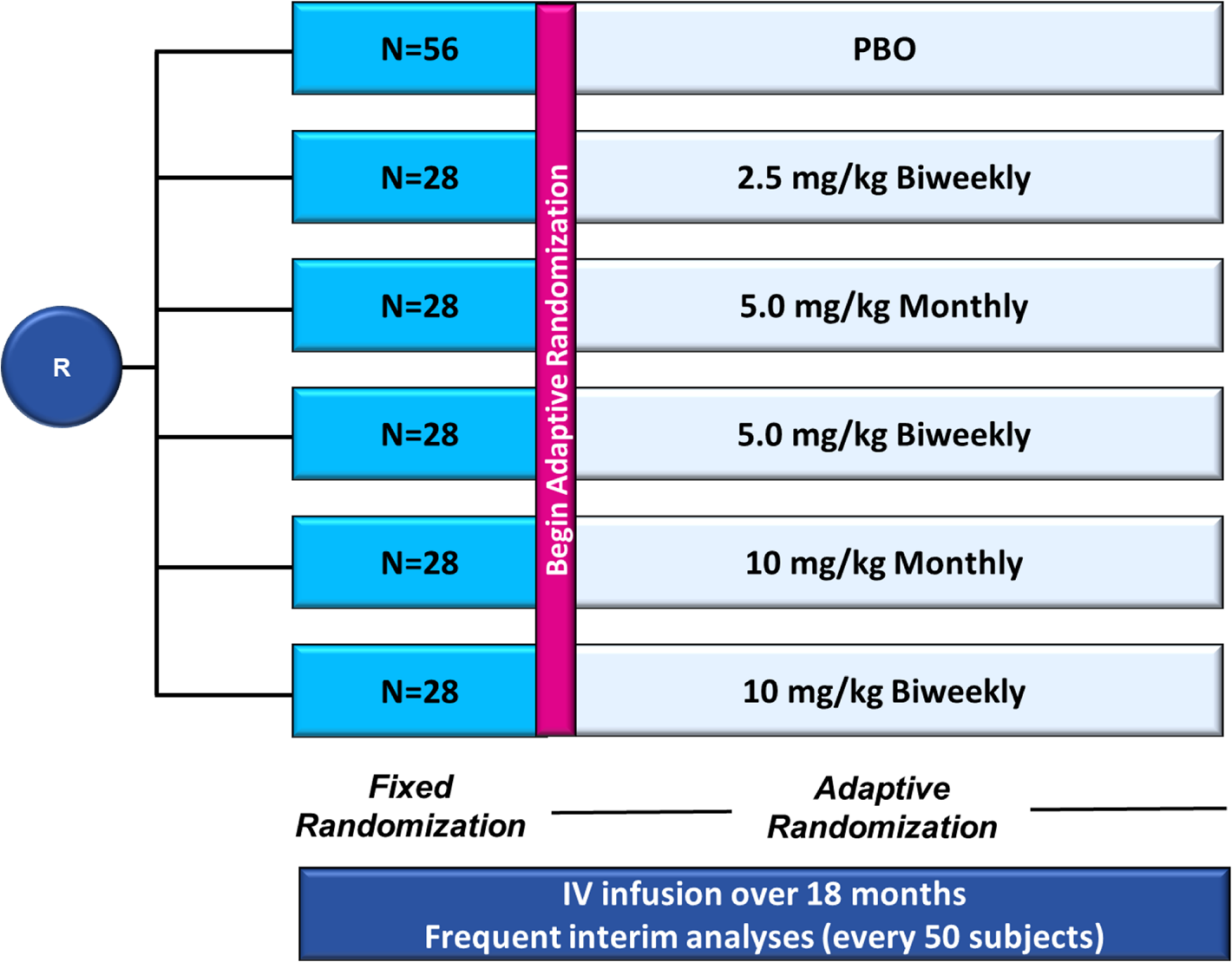
Lecanemab Study 201 study design. Study 201 (NCT01767311) was an 18-month, multicenter, double-blind, placebo-controlled Bayesian design clinical trial employing response adaptive randomization across placebo and five lecanemab arms (2.5 mg/kg biweekly, 5 mg/kg monthly, 5 mg/kg biweekly, 10 mg/kg monthly, 10 mg/kg biweekly) to assess safety and efficacy in subjects with early Alzheimer's disease. At the first three interim analyses, if there is a .5% posterior probability that the most likely ED90 is superior to placebo by the (clinically significant difference; 25%), the trial will stop early for futility. Beginning at the 350-subject IA, and continuing to completion of the trial, the futility criterion is increased to 7.5%. Interim monitoring for early success occurs at each IA beginning when 350 subjects have been enrolled. At this point, if enrollment were to stop for early success, enough subjects would be available to complete the trial so that the full dose response could be modeled. If there is a .95% posterior probability that the most likely ED90 is better than placebo by the CSD, then early success is declared. Enrollment is stopped, but all randomized subjects continue for the full 18-month duration of the study. If the trial is not stopped early for futility or success, then trial success is evaluated at the completion of the trial when both accrual and follow-up for the primary endpoint are complete. At that time, if there is a .80% probability that the most likely ED90 is better than placebo by the CSD, the trial will be considered a success. R, randomization

A full, detailed manuscript on the lecanemab Study 201 design has been previously published [20] and additional details can be found in the study protocol (supplementary appendix A). Study 201 (NCT01767311) was an 18-month, multicenter, double-blind, placebo-controlled Bayesian design clinical trial employing response adaptive randomization across placebo and five lecanemab arms (2.5 mg/kg biweekly, 5 mg/kg monthly, 5 mg/kg biweekly, 10 mg/kg monthly, 10 mg/kg biweekly) to assess safety and efficacy in subjects with early Alzheimer’s disease (Fig. 1). To maintain the blind, all subjects received biweekly infusions of either placebo or lecanemab.

Following a fixed randomization period for the first 196 subjects (*N* = 56 on placebo; *N* = 28 in each lecanemab arm), response-adaptive randomization was implemented where dose allocation probabilities were updated at each blinded interim analysis, conducted every 50 randomized subjects until the approximate target of 800 subjects were randomized (196, 250, 300, 350, …, 800), with the remaining 56 randomized subjects allocated according to the probabilities determined at the 800 subject interim analysis. The Bayesian design aimed to identify the effective dose 90% (ED90), defined as the simplest dose that achieves ≥90% of the maximum treatment effect, and to allocate more subjects to the most likely ED90 dose(s) at each interim analysis [19]. “Simplest” means the earliest in the order of convenience (5 mg/kg monthly, 10 mg/kg monthly, 2.5 mg/kg biweekly, 5 mg/kg biweekly, 10 mg/kg biweekly). Monitoring for futility was initiated at the first interim analysis (IA) and was based on the dose identified as the most likely ED90. The trial would have stopped early for futility at any of the first three IAs if there was a <5% posterior probability that the most likely ED90 is superior to placebo by the clinically significant difference (25%). From the 350-subject IA until the completion of the trial, the futility criterion was increased to 7.5%. Interim monitoring for early success occurred at each IA beginning when 350 subjects had been enrolled, where a .95% posterior probability that the most likely ED90 is better than placebo by the CSD was required. The trial was designed to continue to full completion if neither futility nor early success was achieved according to criteria. At full study completion, the study was considered a success if an 80% probability that the most likely ED90 was better than placebo by the CSD was achieved. In this phase 2 trial, success is defined as a drug effect that exceeds the placebo rate by ≥25%, rather than only being superior to placebo. We chose this criterion to ensure that any early signal of success would likely indicate a robust treatment effect [20]. Upon full randomization, three additional interim analyses were implemented at 3-month intervals from the time of the last subject randomized to assess for early success or futility prior to the final 12-month Bayesian analysis. Subjects remained on the assigned dose/regimen throughout the trial. The adaptive randomization probability for placebo mirrors the probability for the most likely ED90 dose. Details on adaptive randomization probabilities are found in the Simulation Plan provided in the supplemental appendix B. The Bayesian computer algorithm was finalized prior to study start (i.e., not modified during the course of the study). ADCOMS, a score generated from 12 items collected with 3 well-established clinical scales [21], was employed as a sensitive measure of clinical decline in early Alzheimer’s disease intended to aid in driving response-adaptive subject allocation. The trial was approved by the institutional review board or independent ethics committee at each center and all subjects provided informed consent.

There was a notable protocol amendment during the course of the study related to a safety observation for apolipoprotein E4 (ApoE4) gene carriers receiving the highest dose of lecanemab. Emerging data from the study just prior to the 350 subject interim analysis indicated that ApoE4 positive homozygous individuals on the highest dose of lecanemab (10 mg/kg biweekly) had the highest risk of developing symptomatic amyloid-related imaging abnormalities-edema/effusion (ARIA-E). Following comprehensive data review, one regulatory authority requested that ApoE4 carriers (homozygous and heterozygous; approximately 70% of the overall subject population) no longer be administered the 10 mg/kg biweekly dose of lecanemab going forward, and this approach was adopted for all subsequent randomizations. At the same time, a request was made to discontinue from study drug administration, without exception, all ApoE4 carriers (homozygous and heterozygous) who were randomized to the 10 mg/kg biweekly dose and were on study for less than 6 months. Additional details are available in Supplementary Appendix C.

### Subjects

Participants comprised 2 subgroups: mild cognitive impairment due to AD or mild AD dementia. All subjects were confirmed amyloid positive via amyloid positron emission tomography (PET) or cerebrospinal fluid (CSF) Aβ_1–42_ for eligibility. Key inclusion criteria included objective impairment in episodic memory (on Wechsler Memory Scale-IV Logical Memory II [WMS-IV LMII]), Mini Mental State Examination (MMSE) score equal to or greater than 22 at screening and baseline (amended to MMSE 22–28 in EU, except Italy), and naïve to or on stable dose (12 weeks) of approved AD medications.

### Endpoints and assessments

The primary endpoint was change from baseline at 12 months on ADCOMS [20]. Key secondary endpoints were change from baseline at 18 months in brain amyloid by PET Standard Uptake Value ratio (SUVr) in an optional sub-study of consenting participants, ADCOMS, Clinical Dementia Rating-Sum-of-Boxes (CDR-SB), Alzheimer Disease Assessment Scale-Cognitive Subscale (ADAS-Cog14), CSF biomarkers (optional sub-study), and total hippocampal volume using volumetric magnetic resonance imaging (vMRI). An evaluation of the efficacy of lecanemab compared to placebo at 18 months for ADCOMS, CDR-SB, and ADAS-Cog14 within the mild cognitive impairment due to AD (MCI) and mild AD dementia clinical subgroups was also a key secondary endpoint. Subjects were monitored for adverse events at all visits. All subjects with ARIA-E as assessed by MRI were discontinued immediately per protocol, regardless of radiologic severity or symptomatic status. ADCOMS, ADAS-Cog14, CDR-SB, and MMSE were collected every 3 months during the study. CSF samples were collected in consenting participants at baseline, 12, and 18 months, with biomarkers (Aβ_1–42_, phosphorylated tau [p-tau], total tau [t-tau] as well as exploratory biomarkers, including neurogranin, and neurofilament light chain [NfL]) measured by ELISA and SIMOA. Additional details can be found in the study protocol (supplementary appendix A).

### Statistical analyses

Bayesian dose-response data for the primary endpoint were modeled with a 2-dimensional (dose-by-frequency) first-order normal dynamic linear model, where Normal and Inverse-Gamma priors were used. The primary endpoint was met if the Bayesian analysis, when all subjects completed 12 months of treatment, met the threshold of an 80% probability that the ED90 dose achieved at least 25% less clinical decline compared to placebo on ADCOMS. This proof-of-concept study was powered for decline on active treatment being at least 25% less than decline on placebo, which was defined as the clinically significant difference (CSD) for this study. The 12-month primary analysis was intended to allow for the opportunity to accelerate decision making for phase 3. A sample size of approximately 800 subjects was deemed sufficient to meet the 80% probability threshold for the primary endpoint according to treatment-response assumptions [19].

Bayesian and conventional (frequentist) statistical analyses were prospectively defined prior to study start for key secondary endpoints (change from baseline for the treatment groups compared to placebo at 18 months for PET SUVr, ADCOMS, ADAS-Cog14, CDR-SB, vMRI, and CSF biomarkers). Bayesian analyses did not require adjustment for multiplicity by nature of the Bayesian approach. Conventional analyses were performed using a mixed effects model with repeated measures (MMRM) comparing placebo to active arms. All listed *p*-values for key secondary endpoints using conventional statistics are nominal, and no correction was applied for multiplicity. In addition to analyses for individual dose arms, a prospectively defined analysis of key secondary endpoints was performed on subjects from the combined two 10 mg/kg dose regimens, in an attempt to balance the number of ApoE4-positive subjects lost in the 10 mg/kg biweekly dose group brought about by the Regulatory Authority-imposed amendment to the design. The MMRM analysis used treatment group, visit, clinical subgroup (MCI due to AD, Mild AD dementia), the presence or absence of ongoing AD treatment at baseline, ApoE4 status, world region, and treatment group-by-visit interaction as factors, and baseline value as covariate. Safety assessments were summarized by treatment group using descriptive statistics. There was no sample size analysis for the CSF substudy and results for biomarker analyses are presented descriptively. Subjects from the 10 mg/kg biweekly and monthly doses were pooled in the CSF substudy biomarker analyses to increase the sample size.

### Role of the funding source

This trial was funded by Eisai Inc. Authors from Eisai Inc. had input into the study design; in the collection, analysis, and interpretation of data; in the writing of the report; and in the decision to submit the paper for publication. The corresponding author had full access to all the data in the study and had final responsibility for the decision to submit for publication.

## Results

A total of 856 subjects were randomized and 854 were treated (lecanemab, 609; placebo, 245) between December 2012 and November 2017 at 117 sites across North America (the USA and Canada), Europe (France, Germany, Italy, Netherlands, Spain, Sweden, and the UK), and the Asia-Pacific region (Japan and South Korea).

**Fig. 2:**
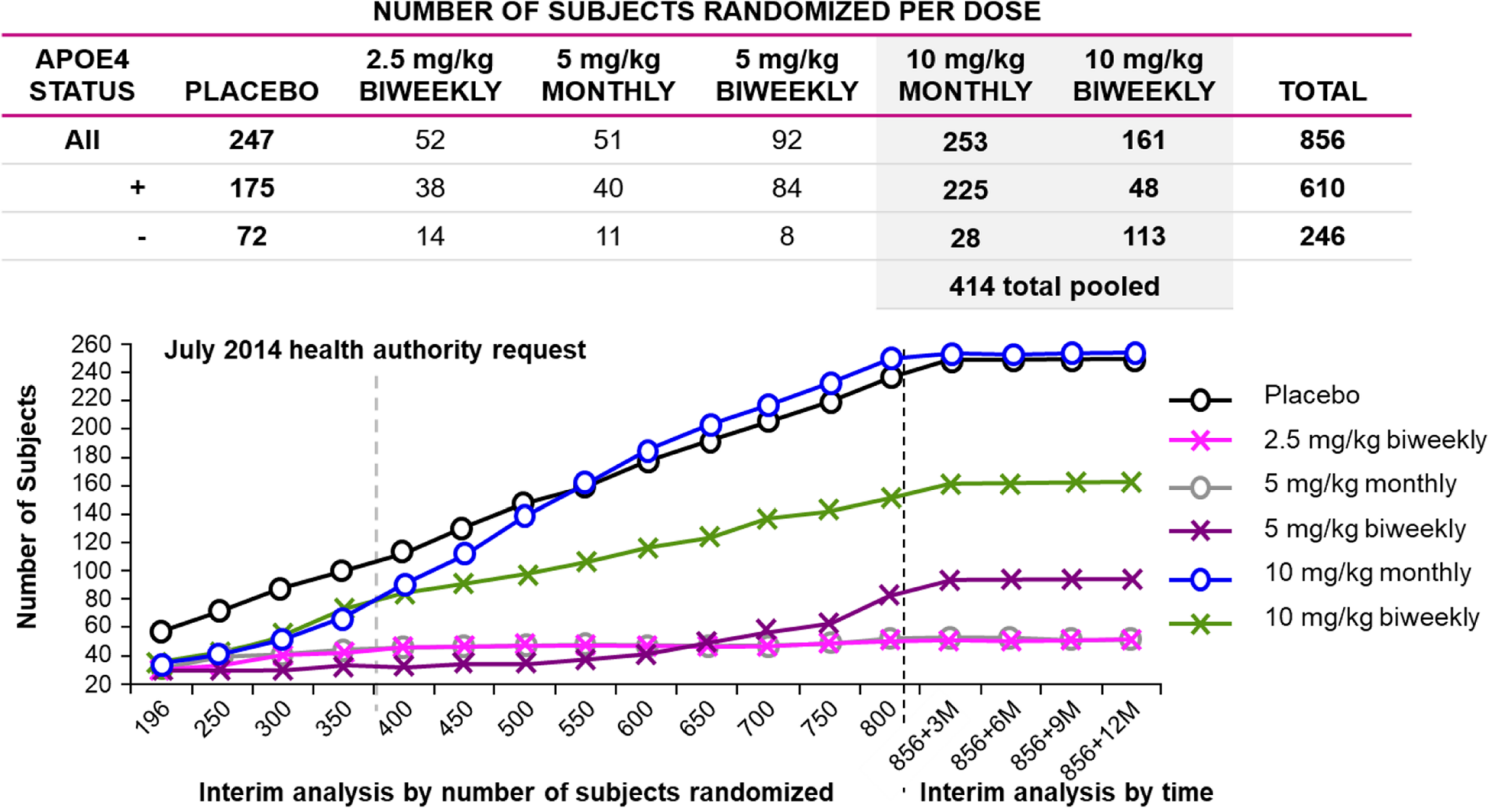
Randomization allocations by treatment group per protocol-defined interim analyses. The response adaptive randomization correctly allocated subjects into the dose groups likely to be ED90 doses (10 mg/kg monthly and biweekly) as early as the first interim analysis at 197 subjects, with both emerging by the 300th subject randomized, and these doses remained the most likely doses to demonstrate efficacy throughout the remainder of the study. However, before the interim analysis of 350 subjects, Health Authorities restricted randomization around ApoE4 carrier status, whereby ApoE4 carriers (hetero- or homozygous) were not to be randomized to the 10 mg/kg biweekly dose going forward. As a consequence, the response adaptive randomization algorithm was revised. After each subsequent interim analysis (starting with 350 subjects randomized), the randomization probability vector was split between ApoE4 carrier and non-carrier strata to ensure no ApoE4 carriers were enrolled on the 10 mg/kg biweekly dose (more details in Appendix C). At the same time, the revised response adaptive randomization preserved the overall randomization probabilities

Allocation of subjects to doses via the adaptive randomization design are shown in Fig. 2. The adaptive randomization algorithm identified both 10 mg/kg biweekly and 10 mg/kg monthly early in the study as potential ED90 doses, and they received the greatest allocation of subjects accordingly. The number of subjects randomized per dose were placebo = 247; 2.5 mg/kg biweekly = 52; 5 mg/kg monthly = 51; 5 mg/kg biweekly = 92; 10 mg/kg monthly = 253; 10 mg/kg biweekly = 161 (Fig. 2). Although the lecanemab 10 mg/kg biweekly dose was identified as the target ED90 dose, it did not receive the highest number of subjects since randomization of ApoE4 carriers (approximately 70% of the subject population) to this dose was prohibited per implementation of the Regulatory Authority request imposed between the period of 300 and 350 randomized subjects. As a result, the lecanemab 10 mg/kg biweekly group had fewer subjects compared to 10 mg/kg monthly with a lower percentage of ApoE4 carriers (30%) relative to all other treatment groups (placebo 71%; 2.5 mg/kg biweekly 73%; 5 mg/kg monthly 77%; 5 mg/kg biweekly 91%; 10 mg/kg monthly 89%). Demographic and baseline characteristics were otherwise similar among treatment groups, with the exception of more male subjects in the lecanemab group relative to placebo (54% vs 42%; Table 1). Baseline characteristics for completers and discontinued subjects are comparable across treatments for each group and can be found in Tables S1-S2.

**Table 1:** Baseline characteristics. Baseline characteristics were reasonably well balanced across doses for each category, with the exception of ApoE4 status. The imbalance in ApoE4 status on the 10 mg/kg monthly and 10 mg/kg biweekly doses is directly related to the change in study design brought about by Health Authority interactions

Category		Lecanemab					
	Placebo	2.5 mg/kg biweekly	5 mg/kg monthly	5 mg/kg biweekly	10 mg/kg Monthly	10 mg/kg biweekly	Total Lecanemab
	(***N*** = 238)	(***N*** = 52)	(***N*** = 48)	(***N*** = 89)	(***N*** = 246)	(***N*** = 152)	(***N*** = 587)
Age, median (range), years	72 (50–89)	71 (50–86)	71 (55–84)	72 (52–87)	71 (53–90)	73 (51–88)	72 (50–90)
Female, *n* (%)	137 (58)	26 (50)	24 (50)	48 (54)	110 (45)	64 (42)	272 (46)
CDR Global = 0.5	200 (84)	44 (85)	40 (83)	77 (87)	210 (85)	133 (88)	504 (86)
Mild cognitive impairment	154 (65)	34 (65)	33 (69)	52 (58)	166 (68)	90 (59)	375 (64)
ApoE4 positive	169 (71)	38 (73)	37 (77)	81 (91)	218 (89)	46 (30)	420 (72)
Ongoing treatment with AChEIs and/or memantine	128 (54)	28 (54)	25 (52)	56 (63)	131 (53)	79 (52)	319 (54)
ADCOMS, mean (SD)	0.37 (0.17)	0.39 (0.20)	0.40 (0.17)	0.39 (0.16)	0.37 (0.15)	0.37 (0.15)	0.38 (0.16)
ADAS-Cog14, mean (SD)^*^	22.6 (7.7)	22.7 (8.1)	22.9 (7.7)	22.8 (6.7)	21.9 (7.3)	22.1 (7.7)	22.2 (7.4)
CDR-SB, mean (SD)	2.9 (1.5)	3.0 (1.6)	2.9 (1.4)	3.0 (1.3)	2.9 (1.3)	3.0 (1.4)	3.0 (1.4)
MMSE, mean (SD)	26.0 (2.3)	25.7 (2.5)	25.3 (2.6)	25.6 (2.3)	25.7 (2.4)	25.6 (2.4)	25.6 (2.4)
PET SUVr, mean (SD)^†^	1.40 (0.16)	1.41 (0.11)	1.42 (0.17)	1.40 (0.12)	1.42 (0.18)	1.37 (0.16)	1.41 (0.16)

*CDR*, Clinical Dementia Rating; *ApoE4*, apolipoprotein E4; *AChEIs*, acetylcholinesterase inhibitors; *ADCOMS*, the Alzheimer’s Disease Composite Score; *ADAS-Cog14*, Alzheimer’s disease Assessment Scale-Cognitive Subscale; *CDR-SB*, Clinical Dementia Rating-Sum-of-Boxes; *MMSE*, Mini Mental State Examination; *PET SUVr*, positron emission tomography standard uptake value ratio

*In the ADAS-Cog14 assessment, there were 237 subjects in the placebo group and 586 in the total lecanemab group (2.5 mg/kg biweekly = 52; 5 mg/kg monthly = 47; 5 mg/kg biweekly = 89; 10 mg/kg monthly = 246; 10 mg/g biweekly = 152)

^†^In the PET sub-study, there were 99 subjects in the placebo group and 216 in the total lecanemab group (2.5 mg/kg biweekly = 28; 5 mg/kg monthly = 28; 5 mg/kg biweekly = 27; 10 mg/kg monthly = 89; 10 mg/g biweekly = 44)

Discontinuations occurred in 23.7% for placebo- and 36.0% for lecanemab-treated subjects. Lecanemab discontinuations were largely driven by ARIA-E events, which resulted in discontinuation per protocol (*N* = 48, 46 lecanemab [1 for 2.5 mg/kg biweekly, 1 for 5 mg/kg monthly, 3 for 5 mg/kg biweekly, 25 for 10 mg/kg monthly, 16 for 10 mg/kg biweekly], 2 placebo) and by implementation of the aforementioned Regulatory Authority request requiring discontinuation of subjects who were ApoE4 carriers and who were on 10 mg/kg lecanemab biweekly for less than 6 months (*n* = 25). Discontinuations due to non-ARIA-E adverse events were similar between placebo and lecanemab. Additional information can be found in the Supplementary Appendix (Figure S1).

### Efficacy

The Bayesian model identified 10 mg/kg biweekly as the effective dose 90% (ED90), defined as the simplest dose that achieves ≥90% of the maximum treatment effect, at the 12-month final Bayesian analysis (Table 2). The primary analysis conducted at month 12 of treatment for all subjects indicated that the 10 mg/kg biweekly (ED90) dose had a 64% probability of being better than placebo with 25% less decline on ADCOMS at 12 months, missing the pre-specified 80% probability threshold for the primary outcome (Table 2). As such, the primary endpoint was not met. Prespecified Bayesian analysis at 18 months determined that the lecanemab 10 mg/kg biweekly dose had a 76% probability of being better than placebo by 25% less decline on ADCOMS (Table S2). Despite 10 mg/kg biweekly not having achieved the threshold needed for superiority over placebo by at least 25%, additional prespecified Bayesian analyses indicated a 97.6% and 97.7% probability of being superior to placebo by any magnitude at both 12 and 18 months, respectively.

**Table 2:** Bayesian Analysis of ADCOMS at 12 months—full analysis set

		Change from baseline		Posterior quantities*			
Treatment group	Total ***N***	Mean	SD	Pr (Max)	Pr (ED90)	Pr Superiority	Pr (CSD)
**ADCOMS—overall**							
Placebo control	229	0.113	0.012	–	–	–	–
2.5 mg/kg biweekly	51	0.134	0.024	0.009	0.009	0.216	0.028
5 mg/kg monthly	48	0.119	0.021	0.022	0.031	0.416	0.070
5 mg/kg biweekly	87	0.116	0.016	0.010	0.010	0.446	0.053
10 mg/kg monthly	242	0.084	0.011	0.318	0.386	0.961	0.479
10 mg/kg biweekly	143	0.077	0.014	0.642	0.563	0.976	0.638

Only subjects with non-missing data at both baseline and the relevant post-baseline visit are included in the change from baseline summary statistics

*ADCOMS*, Alzheimer’s Disease Composite Score; *CSD*, clinically significant difference; 25% better than placebo, *ED90*, dose regimen with at least 90% of the dmax treatment effect; *Max*, maximum treatment effect; *Pr*, probability; *Pr (Max)*, probability of being maximal effective dose; *Pr (ED90)*, probability of being the ED90 dose; *Pr (Superiority)*, probability to be superior to placebo by any magnitude; *Pr (CSD)*, probability to be better than placebo by at least 25%

*Probabilities can be interpreted as a percentage (e.g., 0.10 is 10%)

**Fig. 3:**
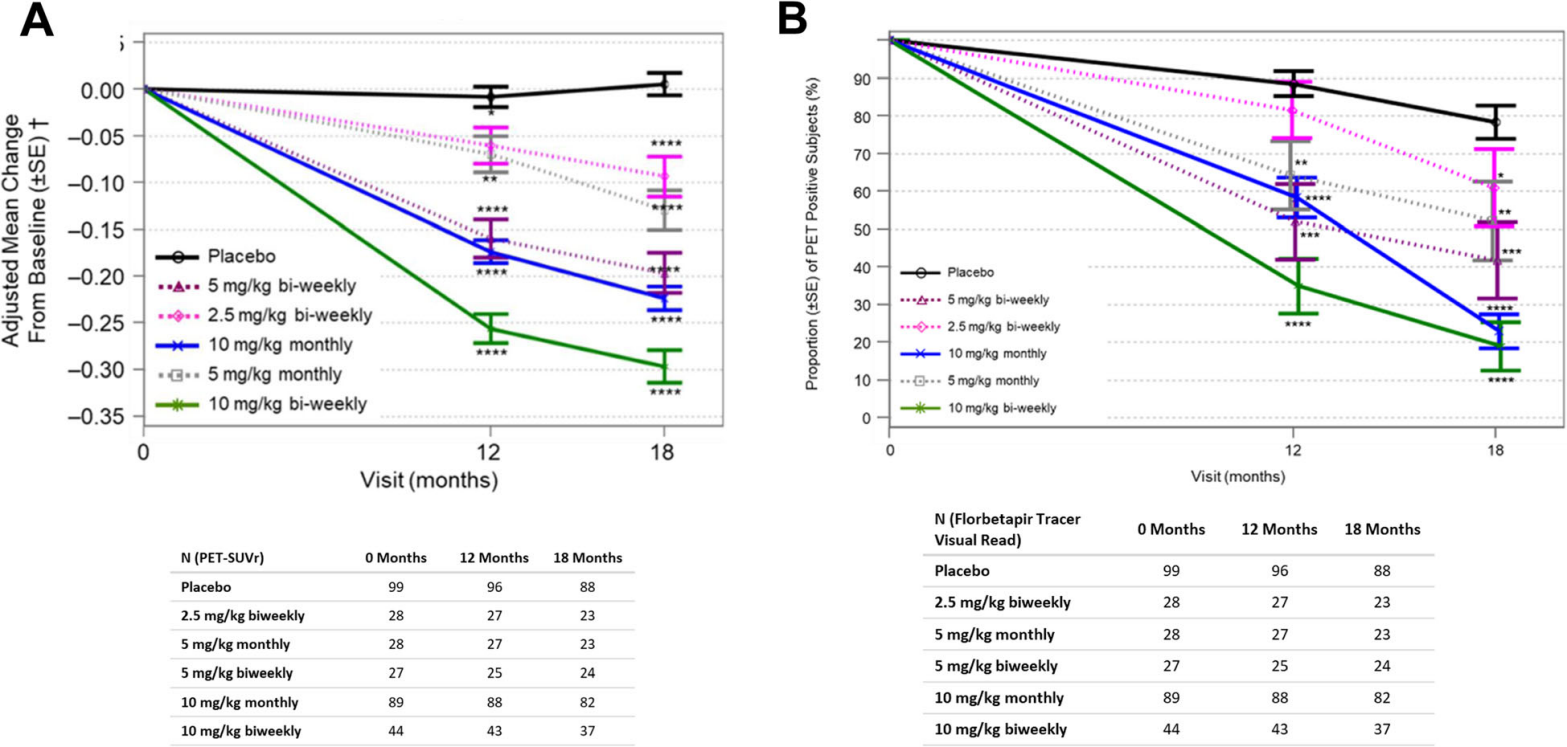
Change from baseline in brain amyloid pathophysiology. Results as measured by amyloid PET SUVr are shown in **a**. Outcomes from the qualitative (binary) visual read of the PET scans for conversion of brain amyloid pathology from positive to negative are depicted in **b**. *P < 0.05, **P < 0.01, ***P < 0.001, ****P < 0.0001 (all nominal). For PET analysis, *N* = 306 at 12 months and *N* = 288 at 18 months. The PET substudy was optional, so only a portion of the total enrolled subject population opted to participate

All reported *p* values are considered nominal because the primary endpoint was not met. Dose-dependent reductions in amyloid PET SUVr values were observed using florbetapir as the imaging agent and whole cerebellum as reference region (Fig. 3a). Binary (positive/negative) amyloid PET visual read analyses indicated results consistent with SUVr data (Fig. 3b). Least squares (LS) mean changes from baseline in amyloid PET SUVr normalized to whole cerebellum mask at 18 months were 0.004 for placebo, and −0.094, −0.131, −0.197, −0.225, and −0.306 for lecanemab 2.5 mg/kg biweekly, 5 mg/kg monthly, 5 mg/kg biweekly, 10 mg/kg monthly, and 10 mg/kg biweekly groups, respectively. The respective LS mean differences from placebo for the individual lecanemab treatment groups were −0.099, −0.136, −0.201, −0.229, and −0.310 (*P* < 0.001 for all doses).

**Fig. 4:**
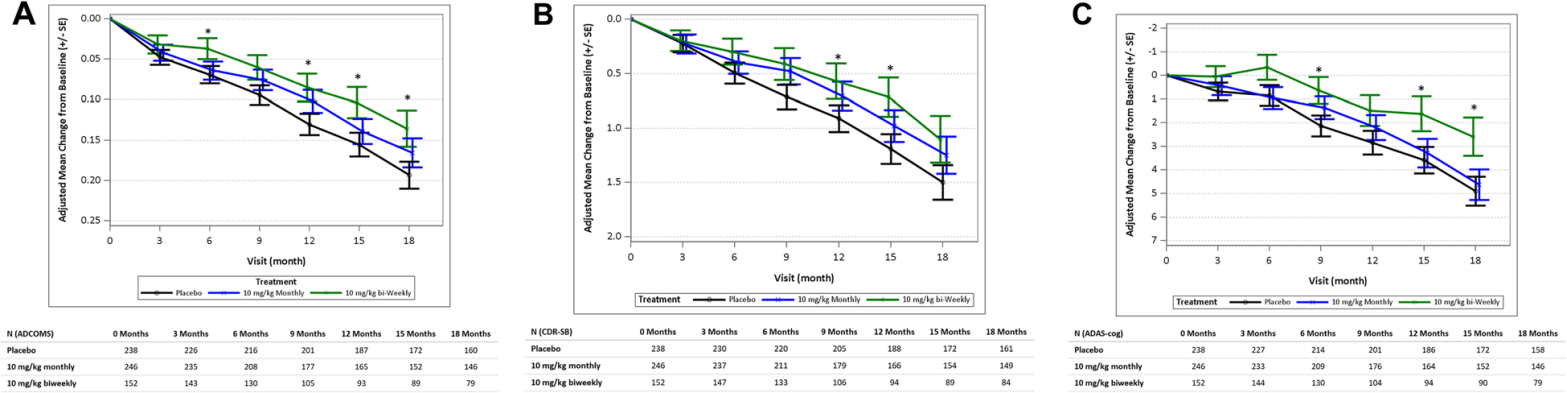
Efficacy assessments. **a** Change from baseline for 10 mg/kg biweekly and monthly doses in the Alzheimer’s Disease Composite Score (ADCOMS). The MMRM used treatment group, visit, clinical subgroup (MCI due to AD, Mild AD), the presence or absence of ongoing AD treatment at baseline, ApoE4 status (positive, negative), region, treatment group-by-visit interaction as factors, and baseline value as covariate. *P < 0.05 (nominal). The primary analysis conducted at month 12 of treatment for all subjects indicated that the 10 mg/kg biweekly dose had a 64% probability to be better than placebo by 25% on ADCOMS at 12 months, missing the pre-specified 80% probability threshold for success. Bayesian analysis at 18 months determined that the lecanemab 10 mg/kg biweekly dose had a 76% probability of being better than placebo by 25% on ADCOMS. In addition, Bayesian analyses indicated a 98% probability of being superior to placebo by any magnitude at both 12 and 18 months, respectively, which is consistent with subsequent conventional analysis results. **b** Results for 10 mg/kg biweekly and monthly doses on CDR-SB. The number of subjects that were assessed at each time point are indicated in the table. The MMRM used treatment group, visit, clinical subgroup (MCI due to AD, Mild AD), the presence or absence of ongoing AD treatment at baseline, ApoE4 status (positive, negative), region, treatment group-by-visit interaction as factors, and baseline value as covariate. *P < 0.05 (nominal). **c** Results for 10 mg/kg biweekly and monthly doses on ADAS-Cog14. The number of subjects that were assessed at each time point are indicated in the table. The MMRM used treatment group, visit, clinical subgroup (MCI due to AD, Mild AD), the presence or absence of ongoing AD treatment at baseline, ApoE4 status (positive, negative), region, treatment group-by-visit interaction as factors, and baseline value as covariate. *P < 0.05 (nominal)

The results for the Bayesian analyses for reduction of clinical decline at 18 months compared to placebo for 10 mg/kg biweekly on ADCOMS (27%; with 97.7% probability to be superior to placebo), CDR-SB (33%; with 96.4% probability to be superior to placebo), and ADAS-Cog14 (56%, with 98.8% probability to be superior to placebo) were similar to the results from the corresponding frequentist analyses for clinical measures when comparing mean change from baseline and LS mean data (additional data found in Tables S3, S4, S5, S6, S7, S8, S9). Figure 4 shows the time course of effects according to frequentist analyses for ADCOMS, CDR-SB, and ADAS-Cog14 at the two doses the Bayesian algorithm determined to be the most meaningful (i.e., 10 mg/kg biweekly and monthly lecanemab doses were allocated the majority of subjects; these two doses are shown for illustrative purposes and data for all dose groups are presented in Figures S2 and S3). In the conventional analyses, lecanemab showed dose-dependent reduction in change from baseline on ADCOMS over 18 months (Figure S2), with 30% (*P* =0.034) less clinical decline compared to placebo at 10 mg/kg biweekly and 15% (*P* = 0.23) less decline at the intermediate dose of 10 mg/kg monthly (Fig. 4a). Results for the 3 lowest doses of lecanemab did not differ from placebo (Figure S2). Lecanemab reduced clinical decline on CDR-SB, with 26% less decline (*P* =0.125) at 10 mg/kg biweekly and 17% (*P* =0.255) with 10 mg/kg monthly, compared to placebo, but these differences did not reach nominal significance (Fig. 4b). Lecanemab at 10 mg/kg biweekly reduced clinical decline on ADAS-Cog14 (Fig. 4c) over 18 months, with 47% (*P* =0.017) less decline compared to placebo. The intermediate dose of 10 mg/kg monthly decreased clinical decline by 6% (*P* = 0.74) on ADAS-Cog14 (Fig. 4a). Numerical differences from placebo were noted as early as 6 months and were maintained over the 18 months of treatment. Lecanemab at 10 mg/kg monthly was an intermediate dose on all clinical measures. Data between the mild AD dementia and the MCI subgroups within the study population were generally consistent but limited and difficult to interpret due to small sample size (Tables S10, S11, S12).

Marginally greater total hippocampal volume reduction (7.56% increased volume decline) was observed at 10 mg/kg biweekly compared to placebo without nominal significance (Table S13; Figure S4A;). Whole brain volume and ventricular volume results reflect increased volume decline in the treatment groups compared to the placebo group (Table S14-S15; Figure S4B-C).

As a post hoc sensitivity analysis to address the imbalance in ApoE4 carriers introduced by the change in allocation rules in response to regulatory input, we analyzed the combined 10 mg/kg dose groups (biweekly and monthly). The analysis for combining both of the 10 mg/kg dose groups was pre-specified and resulted in an ApoE4 carrier balance that approximated the ApoE4 balance of the placebo group and the overall population. Effect sizes were attenuated but favored lecanemab in the combined scenario compared to 10 mg/kg biweekly alone due to the addition of a large number of subjects on a dose (10 mg/kg monthly) with sub-maximum effect (Tables S4-S5, S7, S9, and S13). In frequentist analysis, at 18 months, lecanemab in the combined 10 mg/kg dose groups decreased clinical decline by 20% on ADCOMS (*P* =0.053; Table S9), 22% on ADAS-Cog14 (*P* =0.154; Table S12), and 20% on CDR-SB (*P* =0.119; Table S10) versus placebo. The LS mean difference in amyloid PET SUVr at 18 months of the combined lecanemab 10 mg/kg groups from placebo was −0.253 (*P* < 0.001). Recognizing that the study design was changed early in the study, the impact of the ApoE4 carrier imbalance for the most effective 10 mg/kg biweekly (ED90) dose was explored in Bayesian sensitivity analyses comparing effects of lecanemab in ApoE4 carriers versus non-carriers. Lecanemab at 10 mg/kg biweekly showed greater reductions of cognitive decline in ApoE4 positive subjects versus ApoE4-negative subjects compared to placebo (Table S16). Sensitivity analyses were consistent with results from key secondary MMRM analyses (Table S17).

**Fig. 5:**
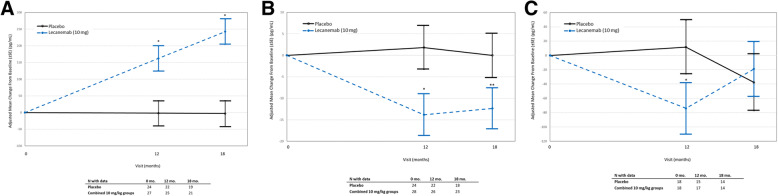
Change from baseline in CSF biomarker measures. **a** Change from baseline in CSF Aβ_1–42_ measures. The combined 10 mg/kg monthly and 10 mg/kg biweekly group is compared versus placebo. The number of subjects that were assessed at each time point are indicated in the table. The MMRM used treatment group, visit, clinical subgroup (MCI due to AD, Mild AD), the presence or absence of ongoing AD treatment at baseline, ApoE4 status (positive, negative), region, treatment group-by-visit interaction as factors, and baseline value as covariate. *P < 0.001. **b** Change from baseline in p-tau measures. The combined 10 mg/kg monthly and 10 mg/kg biweekly group is compared versus placebo. The number of subjects that were assessed at each time point are indicated in the table. The MMRM used treatment group, visit, clinical subgroup (MCI due to AD, Mild AD), the presence or absence of ongoing AD treatment at baseline, ApoE4 status (positive, negative), region, treatment group-by-visit interaction as factors, and baseline value as covariate. *P < 0.001, **P = 0.005. **c** Change from baseline in t-tau measures. The combined 10 mg/kg monthly and 10 mg/kg biweekly group is compared versus placebo. The number of subjects that were assessed at each time point are indicated in the table. The MMRM used treatment group, visit, clinical subgroup (MCI due to AD, Mild AD), the presence or absence of ongoing AD treatment at baseline, ApoE4 status (positive, negative), region, treatment group-by-visit interaction as factors, and baseline value as covariate. *P = 0.029

CSF biomarker analyses in the pooled 10 mg/kg dose arms showed an increase in CSF Aβ42 and decrease in p-tau relative to placebo, whereas inconsistent results were noted at 12 months and 18 months for total tau (Fig. 5a–c). The LS mean difference at 18 months of the combined lecanemab 10 mg/kg treatment groups from placebo was 205.6 (*P* < 0.001), 18.8 (*P* = 0.670), and − 12.3 (*P* = 0.013) for Aβ (1–42), t-tau, and p-tau, respectively. These findings were supported by post-hoc analysis of CSF neurogranin and NfL, which were conducted to further evaluate the impact of lecanemab on downstream pathophysiology of AD (Figure S5A-B).

### Safety

Lecanemab was generally well-tolerated with ARIA-E incidence <10% at the highest doses for the overall population and 14.3% for ApoE4-positive subjects (Table 3). Apart from ARIA-E and infusion reactions, the incidence rates of adverse events, serious adverse events, and treatment-emergent adverse events were consistent with those expected for this population and similar across placebo and lecanemab treatment groups. The most common treatment-emergent adverse events were infusion reaction (3.3% for placebo, 5.8% for 2.5 mg/kg biweekly, 7.8% for 5 mg/kg monthly, 12.0% for 5 mg/kg biweekly, 22.9% for 10 mg/kg monthly, and 19.9% for 10 mg/kg biweekly) and ARIA-E (0.8% for placebo, 1.9% for 2.5 mg/kg biweekly, 2.0% for 5 mg/kg monthly, 3.3% for 5 mg/kg biweekly, 9.9% for 10 mg/kg monthly, and 9.9% for 10 mg/kg biweekly). Infusion reactions were mostly mild to moderate (Grade 1–2) and typically responded to prophylactic treatment. There were no relevant treatment differences between lecanemab and placebo in labs, electrocardiograms, or vital signs. Thirty-seven of the total 48 ARIA-E cases (2 for placebo; 46 for lecanemab) were in ApoE4+ subjects. There were 5 cases of symptomatic ARIA-E (lecanemab: 5/46 [11%]), which generally consisted of headache, visual disturbances, or confusion. Radiologic severity for symptomatic cases were one severe at 2.5 mg/kg biweekly; one mild at 10 mg/kg monthly; one moderate at 10 mg/kg biweekly; and two severe at 10 mg/kg biweekly. The 2.5 mg/kg biweekly case was considered a non-serious adverse event; all other symptomatic cases were considered serious adverse events. Most ARIA-E (60%) occurred within the first 3 months of treatment and were mostly mild-to-moderate (89%) in radiologic severity. All ARIA-E cases resolved, with a typical time course of 4–12 weeks.

**Table 3:** The summary of treatment emergent adverse events and amyloid-related imaging abnormalities-edema (ARIA-E). Lecanemab was generally well-tolerated with similar incidence rates of AEs and SAEs between placebo and lecanemab, and these events were consistent with the subject population. The most common TEAEs were infusion-related reaction (most were mild to moderate in severity, most responded to prophylactic treatment, and few led to discontinuation) and ARIA-E (most were mild to moderate in severity, and were required to discontinue treatment, but encouraged to continue efficacy assessments). Incidence of treatment-related TEAE were similar for placebo and lecanemab for non-ARIA-E-related events

		Lecanemab				
Category	Placebo (*n* = 245) *n* (%)	2.5 mg/kg Biweekly (*n* = 52) *n* (%)	5 mg/kg Monthly (*n* = 51) *n* (%)	5 mg/kg Biweekly (*n* = 92) *n* (%)	10 mg/kg Monthly (*n* = 253) *n* (%)	10 mg/kg Biweekly (*n* = 161) *n* (%)
Any TEAE	216 (88.2)	46 (88.5)	48 (94.1)	81 (88.0)	238 (94.1)	39 (86.3)
Treatment-related TEAE	65 (26.5)	23 (44.2)	25 (49.0)	31 (33.7)	135 (53.4)	76 (47.2)
Serious adverse event	43 (17.6)	10 (19.2)	4 (7.8)	16 (17.4)	31 (12.3)	25 (15.5)
Deaths	2 (0.8)	2 (3.8)	0	1 (1.1)	2 (0.8)	0
AE leading to discontinuation	15 (6.1)	7 (13.5)	4 (7.8)	10 (10.9)	47 (18.6)	24 (14.9)
ARIA-E	2 (0.8)	1 (1.9)	1 (2.0)	3 (3.3)	25 (9.9)	16 (9.9)
ApoE4-positive (*n* = 436)	2 (1.1)	1 (2.6)	1 (2.5)	3 (3.6)	23 (10.2)	7 (14.3)
ApoE4-negative(*n* = 112)	0	0	0	0	2 (7.1)	9 (8.0)

*TEAE*, treatment emergent adverse event; *ARIA-E*, amyloid-related imaging abnormalities-edema; *ApoE4*, apolipoprotein E4

In this study, ARIA-H (new cerebral microhemorrhages, cerebral macrohemorrhages, and superficial siderosis) regardless of the presence of ARIA-E were observed in 13 (5.3%) subjects in the placebo group (*N* = 245) compared to 65 (10.7%) subjects in the lecanemab groups (*N* = 609). Although there was a higher incidence of ARIA-H on lecanemab than placebo, there were no dose-related trends in the incidence of ARIA-H on lecanemab (2 [3.8%], 7 [13.7%], 17 [18.5%], 28 [11.1%], and 11 [6.8%] subjects in the 2.5 mg/kg biweekly, 5 mg/kg monthly, 5 mg/kg biweekly, 10 mg/kg monthly, and 10 mg/kg biweekly groups, respectively). As seen for the incidence of ARIA-E, the incidence of ARIA-H on lecanemab was higher in ApoE4 carriers (*N* = 436) (57 [13.1%] subjects) than in ApoE4 non-carriers (*N* = 173) (8 [4.6%] subjects). ARIA-H occurred concurrently with ARIA-E, or after the onset of ARIA–E in 1 (0.4%) subject in the placebo group; 1 (2.0%), 1 (1.1%), 13 (5.1%), and 7 (4.3%) subjects in the 5 mg/kg monthly subjects, 5 mg/kg biweekly, 10 mg/kg monthly, and 10 mg/kg biweekly groups, respectively; and in 22 (3.6%) subjects in the lecanemab treatment groups overall. There were no symptomatic cases of ARIA-H in Study 201.

## Discussion

This was the first randomized phase 2 clinical trial evaluating subjects with early Alzheimer’s disease receiving treatment with lecanemab, an anti-protofibril antibody that targets soluble aggregated forms of amyloid beta (Aβ), including oligomers and protofibrils. Bayesian response adaptive randomization was employed for efficient subject allocation to the most effective dose(s) in the trial. The 10 mg/kg biweekly and monthly doses were identified as potential effective dose 90% (ED90) doses early in the study, with 10 mg/kg biweekly dose determined to be the final ED90 dose (defined as the simplest treatment group that achieves at least 90% of the modeled maximum treatment effect). The primary endpoint, requiring an 80% probability of ≥25% reduction in clinical decline compared to placebo in a Bayesian analysis of ADCOMS at 12 months, was not met. Results from prespecified key secondary endpoint analyses demonstrated that lecanemab reduced brain amyloid and showed early and sustained activity for 10 mg/kg biweekly lecanemab across the 18-month treatment period for several clinical measures of AD. Importantly, Bayesian and conventional statistical approaches yielded consistent results for lecanemab on clinical decline. Lecanemab was generally well-tolerated, with the key adverse event being ARIA-E with an incidence rate of less than 10% at the two highest doses for the overall population.

Dose-dependent and robust reductions in amyloid PET SUVr values were observed using florbetapir as the imaging agent across all doses (nominal *P* < 0.0001 at 18 months of treatment for all doses). The baseline PET SUVr for the 10 mg/kg biweekly group was 1.37, with a mean change of −0.31 at 18 months, suggesting on average, that subjects treated with this dose fall below the SUVr threshold for amyloid-PET positivity for florbetapir. Consistent with PET SUVr results, a substantially larger proportion of subjects on lecanemab 10 mg/kg biweekly converted (by visual read) to amyloid negative (81%) compared to placebo (22%; placebo variability is generally attributed to variability associated with borderline amyloid PET positive cases).

Volumetric MRI results indicated that lecanemab treatment at 10 mg/kg biweekly marginally increased hippocampal volume loss; effects on increased whole brain volume loss and total ventricular volume increase were more pronounced. While these effects are generally consistent with literature reports for other amyloid-targeting agents [22–24], the reasons for these observations are unclear, but may be related to antibody profile and Aβ clearance, as has been suggested previously [25]. The long-term implications of these findings are unknown and may be assessed in longer term follow-up and in the phase 3 study BAN2401-G000-301 (Clarity AD).

In a CSF substudy, AD-related changes in Aβ1–42 and p-tau levels were nominally increased and decreased respectively (both moving to become more normal) in the combined 10 mg/kg lecanemab dose groups relative to placebo at 18 months, whereas results for t-tau did not differ. AD diagnosis is associated with increased plaque burden in the brain and a decrease in Aβ1–42 monomers in CSF due to sequestration of monomeric Aβ1–42 into amyloid plaques [26]. The increase in CSF Aβ1–42 observed in Study 201 may reflect changes in the dynamics of amyloid aggregation or normalization of monomeric amyloid levels related to amyloid plaque clearance. Mechanistic studies are needed to better understand the meaning of the increase in CSF Aβ1–42 and how it relates to the treatment effect. Lecanemab-mediated effects on p-tau are consistent with previous reports involving amyloid therapies [27–29] and suggest that targeting amyloid may influence the downstream neurodegenerative processes associated with AD. We included the post hoc analysis of CSF neurogranin and NfL that were not prospectively defined as secondary endpoints in this study. Both these measures showed changes consistent with a treatment effect. While these biomarkers were not established at the time the study was initiated, we consider it important to include the full complement of biomarker results here, as neurogranin and NfL are rapidly gaining acceptance as markers of neurodegeneration [30], and they may help provide context to the overall results in Study 201.

Most previously published studies using other putative disease-modifying agents for AD did not show appreciable clinical efficacy in phase 3 [19, 31]. In this study, it is considered that several features helped to optimize the detection of clinical differences in a relatively slowly progressing early Alzheimer’s disease population. Importantly, eligibility requirements dictated that all subjects have confirmed amyloid pathology, a feature lacking in some phase 3 studies. Moreover, the lecanemab mechanism of action is distinct among other anti-amyloid agents in that it has high selectivity for soluble aggregate species of Aβ compared to monomeric amyloid, with moderate selectivity over fibrillar amyloid; a profile thought to convey an advantage in selectively targeting the most toxic pathologic amyloid species. Finally, the Bayesian design ensured that more subjects be allocated to the most effective dose or doses (i.e., potential ED90 dose(s)), while ADCOMS was used as a sensitive tool to help inform frequent response adaptive allocations through early detection of clinical potential [21].

The 10 mg/kg biweekly group, despite being limited in size and ApoE4+ balance, showed nominally significant differences versus placebo on ADCOMS and ADAS-Cog14 at 18 months of treatment. The 10 mg/kg monthly group demonstrated intermediate effects relative to placebo across assessments, but these differences were not nominally significant. None of the other dose groups differed from placebo most likely due to the relatively small sample size allocated to these sub-optimal doses by the Bayesian adaptive randomization algorithm.

### Limitations

There are some important limitations for this study. Most notably, a small number of symptomatic ARIA-E cases at 10 mg/kg biweekly prompted one Regulatory Authority to request that subsequent ApoE4+ subjects (approximately 70% of the overall study population) not be randomized to the 10 mg/kg biweekly dose. An additional component to the request required that all ApoE4+ subjects on 10 mg/kg biweekly who were on study but who had not yet reached 6 months of treatment be immediately discontinued (*N* = 25 subjects). Implementation of these actions led to early (initiated between 300 and 350 randomized participants) and significant changes to the study design that resulted in a marked imbalance in the number of ApoE4+ subjects on 10 mg/kg biweekly (30% ApoE4+ subjects). The Bayesian algorithm identified 10 mg/kg biweekly as the ED90 dose despite this imposed design limitation. The constraints in this analysis were specifically associated with the ED90 dose and therefore could have had an impact on the ability to interpret the results. The safety and efficacy of 10 mg/kg biweekly lecanemab is currently being evaluated in an open label extension to this study, and in the phase 3 lecanemab Clarity AD study, where there are no dosing restrictions based on ApoE status in either study.

ARIA-E rates were ~ 10% for both the 10 mg/kg monthly and 10 mg/kg biweekly doses and < 15% for ApoE4-positive subjects. The assessment of early ARIA-E cases that led to Study 201 design changes were determined based on limited experience and understanding of lecanemab at the time of trial initiation. Additional experience since that time, both with lecanemab and other anti-amyloid monoclonal antibodies, has reframed the understanding and management of ARIA-E. In general, ARIA-E is dose- and ApoE4-status (ApoE4+ > ApoE4-) dependent, is observed early in treatment, is predominantly asymptomatic, can be effectively monitored, and is radiologically reversible on MRI. Symptomatic cases generally involve mild and transient symptoms that resolve without sequelae; vigilance is continuing to improve understanding of the risks and management of this effect. Based on the emerging clinical understanding of ARIA-E and its management, the full 10 mg/kg biweekly dose is implemented in ApoE4 carriers in the open label extension to this study, and in the phase 3 lecanemab Clarity AD study, with appropriate monitoring and management criteria.

## Conclusions

This study was designed as an 18-month, proof-of-concept study. The study explored the dose response of lecanemab over three dose levels and two dosing regimens with the objective to establish the most effective (ED90) dose of lecanemab based on ADCOMS. A 12-month primary endpoint was utilized to allow for the opportunity to accelerate the development program, if possible, through the use of a Bayesian adaptive design; however, the study was to complete the blinded 18-month treatment period regardless of the 12-month outcome. The Bayesian design identified 10 mg/kg biweekly as the ED90 dose. Proof of concept was supported through prespecified key secondary endpoint analyses, where lecanemab treatment resulted in a dose dependent and consistent reduction in clinical decline relative to placebo across a number of clinical endpoints according to Bayesian and frequentist approaches. These effects were accompanied by a dose-dependent reduction in brain amyloid PET over 18 months of treatment and were reinforced by additional CSF biomarker results. Taken together, the findings in this double-blind trial on multiple cognitive endpoints and biomarkers are supportive of the therapeutic concept for the targeting specific oligomeric species (protofibrils) in the process of pathophysiological amyloid generation in AD. The confirmation of the effects of lecanemab are being evaluated in the Phase 3 Clarity AD study in early Alzheimer’s disease.

## Supplementary Information

**Additional file 1: Supplemental Figure S1.** CONSORT 2010 Flow Diagram. **Supplemental Figure S2.** Change from Baseline for all Treatment Groups in the Alzheimer's Disease Composite Score (ADCOMS). **Supplemental Figure S3.** Results for ADAS-cog (3SA) and CDR-SB (3SB) for All Dosing groups. **Supplemental Figure S4.** Results for Total Hippocampal Volume (S4A), Whole Brain Volume (S4B), and Ventricular Volume for All Dosing Groups (S4C). **Supplemental Figure S5.** Change from Baseline in Neurogranin Measures (5SA). Change from Baseline in Neurofilament Light Chain Measures (5SB). **Supplemental Table S1.** Baseline Characteristics for Completers – Full Analysis Set. **Supplemental Table S2.** Baseline Characteristics for Subjects who Discontinued Treatment – Full Analysis Set. **Supplemental Table S3.** Bayesian Analysis of ADCOMS at 18 Months – Full Analysis Set. **Supplemental Table S4.** Summary of MMRM Analyses for Change from Baseline in ADCOMS at 12 Months – Full Analysis Set. **Supplemental Table S5.** Summary of MMRM Analyses for Change from Baseline in ADCOMS at 18 Months – Full Analysis Set. **Supplemental Table S6.** Bayesian Analysis of CDR-SB at 18 Months – Full Analysis Set. **Supplemental Table S7.** Summary of MMRM Analyses for Change from Baseline in CDR-SB at 18 Months – Full Analysis Set. **Supplemental Table S8.** Bayesian Analysis of ADAS-Cog14 at 18 Months – Full Analysis Set. **Supplemental Table S9.** Summary of MMRM Analyses for Change from Baseline in ADAS-Cog14 at 18 Months – Full Analysis Set. **Supplemental Table S10.** Summary of MMRM Analyses for ADCOMS at 18 Months for Disease Stage (MCI due to AD and Mild AD Dementia) Subgroups - Full Analysis Set. **Supplemental Table S11.** Summary of MMRM Analyses for ADAS–Cog14 at 18 Months for Disease Stage (MCI due to AD and Mild AD Dementia) – Full Analysis Set. **Supplemental Table S12.** Summary of MMRM Analyses for CDR-SB at 18 Months for Disease Stage (MCI due to AD and Mild AD Dementia) – Full Analysis Set. **Supplemental Table S13.** Summary of MMRM Analyses for Change from Baseline in Total Hippocampal Volume at 18 Months. **Supplemental Table S14.** Summary of MMRM Analyses for Change from Baseline in vMRI Whole Brain Volume at 18 Months – MMRM Pharmacodynamic Analysis Set. **Supplemental Table S15.** Summary of MMRM Analyses for Change from Baseline in vMRI Total Ventricular Volume at 18 Months – MMRM Pharmacodynamic Analysis Set. **Supplemental Table S16.** Bayesian Analysis of ADCOMS at 18 Months for ApoE4 Genotype (Carrier or Non-Carrier) Subgroups - Full Analysis Set. **Supplemental Table S17.** Sensitivity Analyses for Efficacy Assessments. **Supplementary Appendix A.** Study Protocol. **Supplementary Appendix B.** Simulation Plan. **Supplementary Appendix C.** Additional Detail on Cases of ARIA-E. **Supplementary Appendix D.** Principal Investigators from Participating Enrolling Centers.

## Data Availability

The datasets used and/or analyzed during the current study are available from the corresponding author on reasonable request.
