# Correction to: A randomized, double-blind, phase 2b proof-of-concept clinical trial in early Alzheimer’s disease with lecanemab, an anti-Aβ protofibril antibody

**DOI:** 10.1186/s13195-022-00995-9

**Published:** 2022-05-21

**Authors:** Chad J. Swanson, Yong Zhang, Shobha Dhadda, Jinping Wang, June Kaplow, Robert Y. K. Lai, Lars Lannfelt, Heather Bradley, Martin Rabe, Akihiko Koyama, Larisa Reyderman, Donald A. Berry, Scott Berry, Robert Gordon, Lynn D. Kramer, Jeffrey L. Cummings

**Affiliations:** 1 Eisai Inc., Woodcliff Lake, NJ USA; 2 Eisai Ltd., Hatfield, UK; 3 BioArctic AB, Warfvinges väg 35, SE-112 51 Stockholm, Sweden; 4 Department of Public Health/Geriatrics, Uppsala University, Uppsala, Sweden; 5 Berry Consultants, LLC, Austin, TX USA; 6 Chambers-Grundy Center for Transformative Neuroscience, Department of Brain Health, School of Integrated Health Sciences, University of Nevada Las Vegas, Las Vegas, NV USA

**Correction: Alz Res Therapy 13, 80 (2021)**

**https://doi.org/10.1186/s13195-021-00813-8**

Following the publication of the original article [1] files for Supplementary Appendices A and B were not included in the publication of the original article.

**Supplementary Appendix A:** Study Protocol (BAN2401-G000-201--protocol.pdf)

**Supplementary Appendix B:** Simulation Plan (simulation plan.pdf)

The original article [1] has been updated.
