# Supplementary material for: A randomized, double-blind, phase 2b proof-of-concept clinical trial in early Alzheimer’s disease with lecanemab, an anti-Aβ protofibril antibody
Source: Alzheimers Res Ther. 2021 Apr 17;13:80. doi: 10.1186/s13195-021-00813-8 (PMC8053280; doi:10.1186/s13195-021-00813-8)
Supplement: Supplementary file 1 — Additional file 1: Supplemental Figure S1. CONSORT 2010 Flow Diagram. Supplemental Figure S2. Change from Baseline for all Treatment Groups in the Alzheimer's Disease Composite Score (ADCOMS). Supplemental Figure S3. Results for ADAS-cog (3SA) and CDR-SB (3SB) for All Dosing groups. Supplemental Figure S4. Results for Total Hippocampal Volume (S4A), Whole Brain Volume (S4B), and Ventricular Volume for All Dosing Groups (S4C). Supplemental Figure S5. Change from Baseline in Neurogranin Measures (5SA). Change from Baseline in Neurofilament Light Chain Measures (5SB). Supplemental Table S1. Baseline Characteristics for Completers – Full Analysis Set. Supplemental Table S2. Baseline Characteristics for Subjects who Discontinued Treatment – Full Analysis Set. Supplemental Table S3. Bayesian Analysis of ADCOMS at 18 Months – Full Analysis Set. Supplemental Table S4. Summary of MMRM Analyses for Change from Baseline in ADCOMS at 12 Months – Full Analysis Set. Supplemental Table S5. Summary of MMRM Analyses for Change from Baseline in ADCOMS at 18 Months – Full Analysis Set. Supplemental Table S6. Bayesian Analysis of CDR-SB at 18 Months – Full Analysis Set. Supplemental Table S7. Summary of MMRM Analyses for Change from Baseline in CDR-SB at 18 Months – Full Analysis Set. Supplemental Table S8. Bayesian Analysis of ADAS-Cog14 at 18 Months – Full Analysis Set. Supplemental Table S9. Summary of MMRM Analyses for Change from Baseline in ADAS-Cog14 at 18 Months – Full Analysis Set. Supplemental Table S10. Summary of MMRM Analyses for ADCOMS at 18 Months for Disease Stage (MCI due to AD and Mild AD Dementia) Subgroups - Full Analysis Set. Supplemental Table S11. Summary of MMRM Analyses for ADAS–Cog14 at 18 Months for Disease Stage (MCI due to AD and Mild AD Dementia) – Full Analysis Set. Supplemental Table S12. Summary of MMRM Analyses for CDR-SB at 18 Months for Disease Stage (MCI due to AD and Mild AD Dementia) – Full Analysis Set. Supplemental Table S13. Summary of MMRM Ana [file 13195_2021_813_MOESM1_ESM.zip › 13195_2021_813_MOESM1_ESM.docx]

**Supplementary Appendix**

**FIGURES**

**Supplemental Figure S1.** CONSORT 2010 Flow Diagram

**Supplemental** **Figure S2.** Change from Baseline for all Treatment Groups in the Alzheimer's Disease Composite Score (ADCOMS).

**Supplemental Figure S3.** Results for ADAS-cog (3SA) and CDR-SB (3SB) for All Dosing groups.

**Supplemental Figure S4.** Results for Total Hippocampal Volume (S4A), Whole Brain Volume (S4B), and Ventricular Volume for All Dosing Groups (S4C)

**Supplemental Figure S5.** Change from Baseline in Neurogranin Measures (5SA). Change from Baseline in Neurofilament Light Chain Measures (5SB)

**TABLES**

**Supplemental Table S1.** Baseline Characteristics for Completers – Full Analysis Set

**Supplemental Table S2.** Baseline Characteristics for Subjects who Discontinued Treatment – Full Analysis Set

**Supplemental Table S3.** Bayesian Analysis of ADCOMS at 18 Months – Full Analysis Set.

**Supplemental Table S4.** Summary of MMRM Analyses for Change from Baseline in ADCOMS at 12 Months – Full Analysis Set

**Supplemental Table S5.** Summary of MMRM Analyses for Change from Baseline in ADCOMS at 18 Months – Full Analysis Set.

**Supplemental Table S6.** Bayesian Analysis of CDR-SB at 18 Months – Full Analysis Set.

**Supplemental Table S7.** Summary of MMRM Analyses for Change from Baseline in CDR-SB at 18 Months – Full Analysis Set.

**Supplemental Table S8.** Bayesian Analysis of ADAS-Cog14 at 18 Months – Full Analysis Set.

**Supplemental Table S9.** Summary of MMRM Analyses for Change from Baseline in ADAS-Cog14 at 18 Months – Full Analysis Set.

**Supplemental Table S10.** Summary of MMRM Analyses for ADCOMS at 18 Months for Disease Stage (MCI due to AD and Mild AD Dementia) Subgroups - Full Analysis Set

**Supplemental Table S11.** Summary of MMRM Analyses for ADAS–Cog14 at 18 Months for Disease Stage (MCI due to AD and Mild AD Dementia) – Full Analysis Set

**Supplemental Table S12.** Summary of MMRM Analyses for CDR-SB at 18 Months for Disease Stage (MCI due to AD and Mild AD Dementia) – Full Analysis Set.

**Supplemental Table S13.** Summary of MMRM Analyses for Change from Baseline in Total Hippocampal Volume at 18 Months.

**Supplemental Table S14.** Summary of MMRM Analyses for Change from Baseline in vMRI Whole Brain Volume at 18 Months – MMRM Pharmacodynamic Analysis Set.

**Supplemental Table S15.** Summary of MMRM Analyses for Change from Baseline in vMRI Total Ventricular Volume at 18 Months – MMRM Pharmacodynamic Analysis Set.

**Supplemental Table S16.** Bayesian Analysis of ADCOMS at 18 Months for ApoE4 Genotype (Carrier or Non-Carrier) Subgroups - Full Analysis Set.

**Supplemental Table S17.** Sensitivity Analyses for Efficacy Assessments.

**APPENDICES**

**Supplementary Appendix A:** Study Protocol

**Supplementary Appendix B:** Simulation Plan.

**Supplementary Appendix C:** Additional Detail on Cases of ARIA-E.

**Supplementary Appendix D:** Principal Investigators from Participating Enrolling Centers.

**Supplemental Figure S1.** CONSORT 2010 Flow Diagram

**
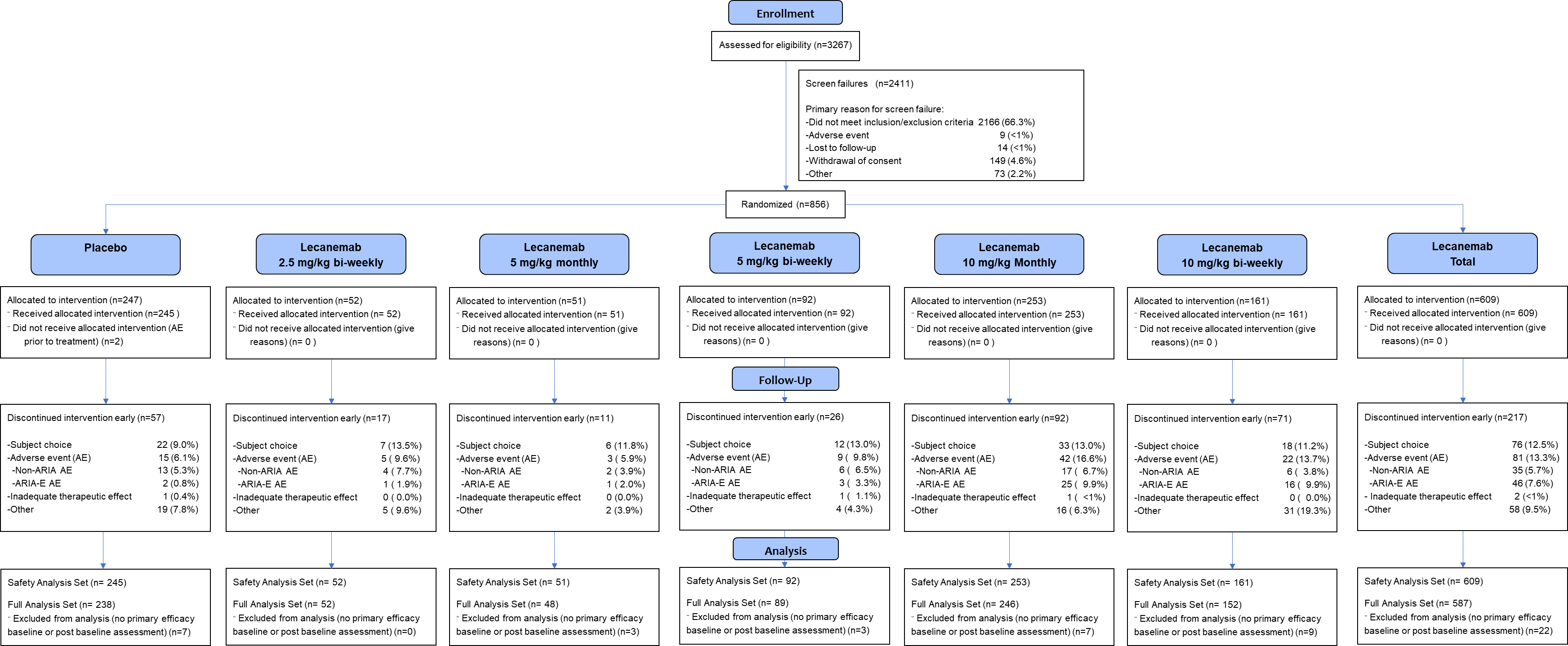
**

**Supplemental Figure S2.** Change from Baseline for all treatment groups in the Alzheimer's Disease Composite Score (ADCOMS). The MMRM used treatment group, visit, clinical subgroup (MCI due to AD, Mild AD), the presence or absence of ongoing AD treatment at baseline, APOE4 status (positive, negative), region, treatment group-by-visit interaction as factors, and baseline value as covariate. *P<0.05 (nominal). The primary analysis conducted at month 12 of treatment for all subjects indicated that the 10 mg/kg biweekly dose had a 64% probability to be better than placebo by 25% on ADCOMS at 12 months, missing the pre-specified 80% probability threshold for success. Bayesian analysis at 18 months determined that lecanemab 10 mg/kg biweekly dose had a 76% probability of being better than placebo by 25% on ADCOMS. In addition, Bayesian analyses indicated a 98% probability of being superior to placebo by any magnitude at both 12 and 18 months, respectively, which is consistent with subsequent conventional analysis results.


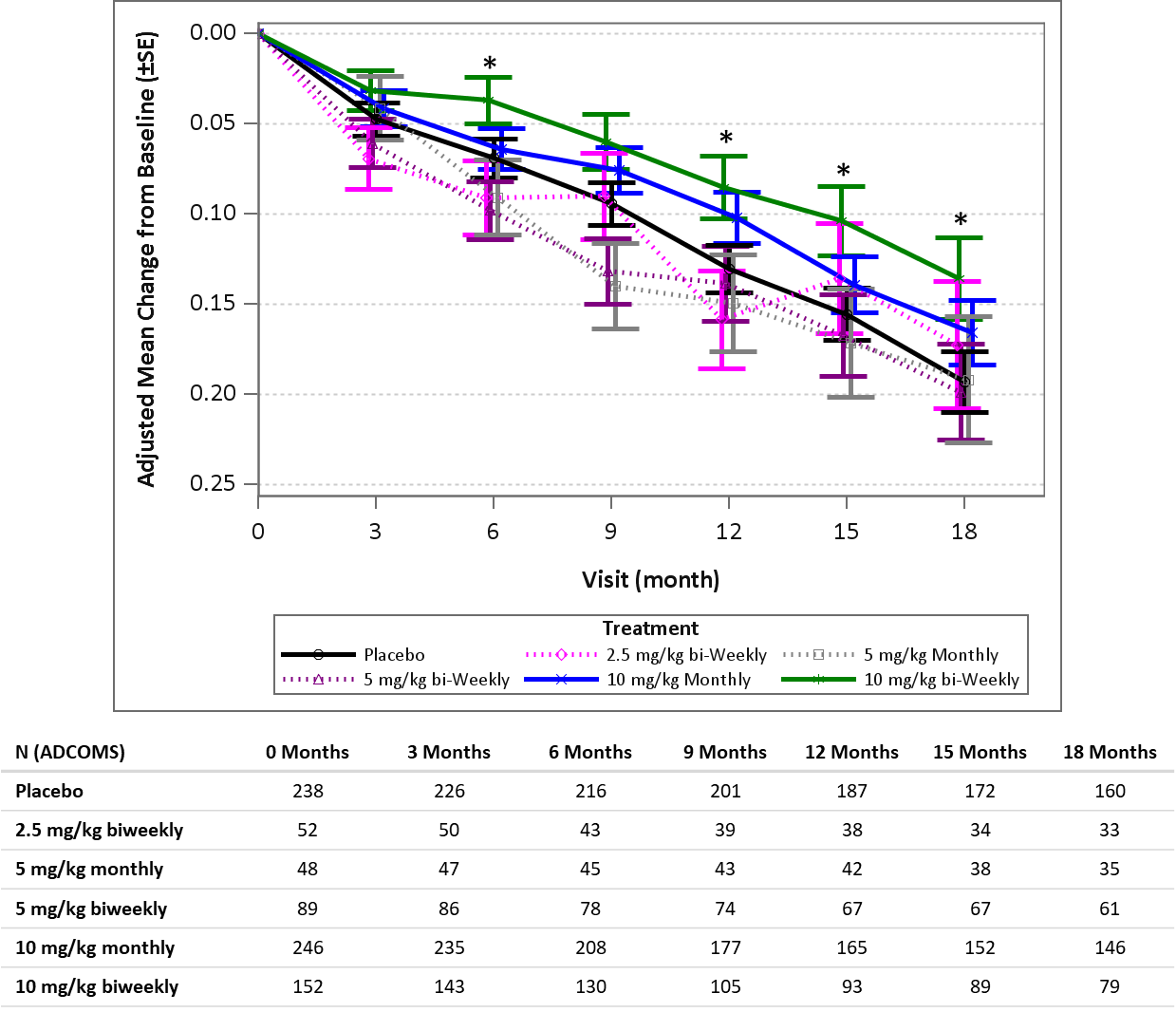


**Supplemental Figure S3.** Results for ADAS-cog (S3A) and CDR-SB (S3B) for all dosing groups. The number of subjects that were assessed at each time point are indicated in the table. The MMRM used treatment group, visit, clinical subgroup (MCI due to AD, Mild AD), the presence or absence of ongoing AD treatment at baseline, APOE4 status (positive, negative), region, treatment group-by-visit interaction as factors, and baseline value as covariate.


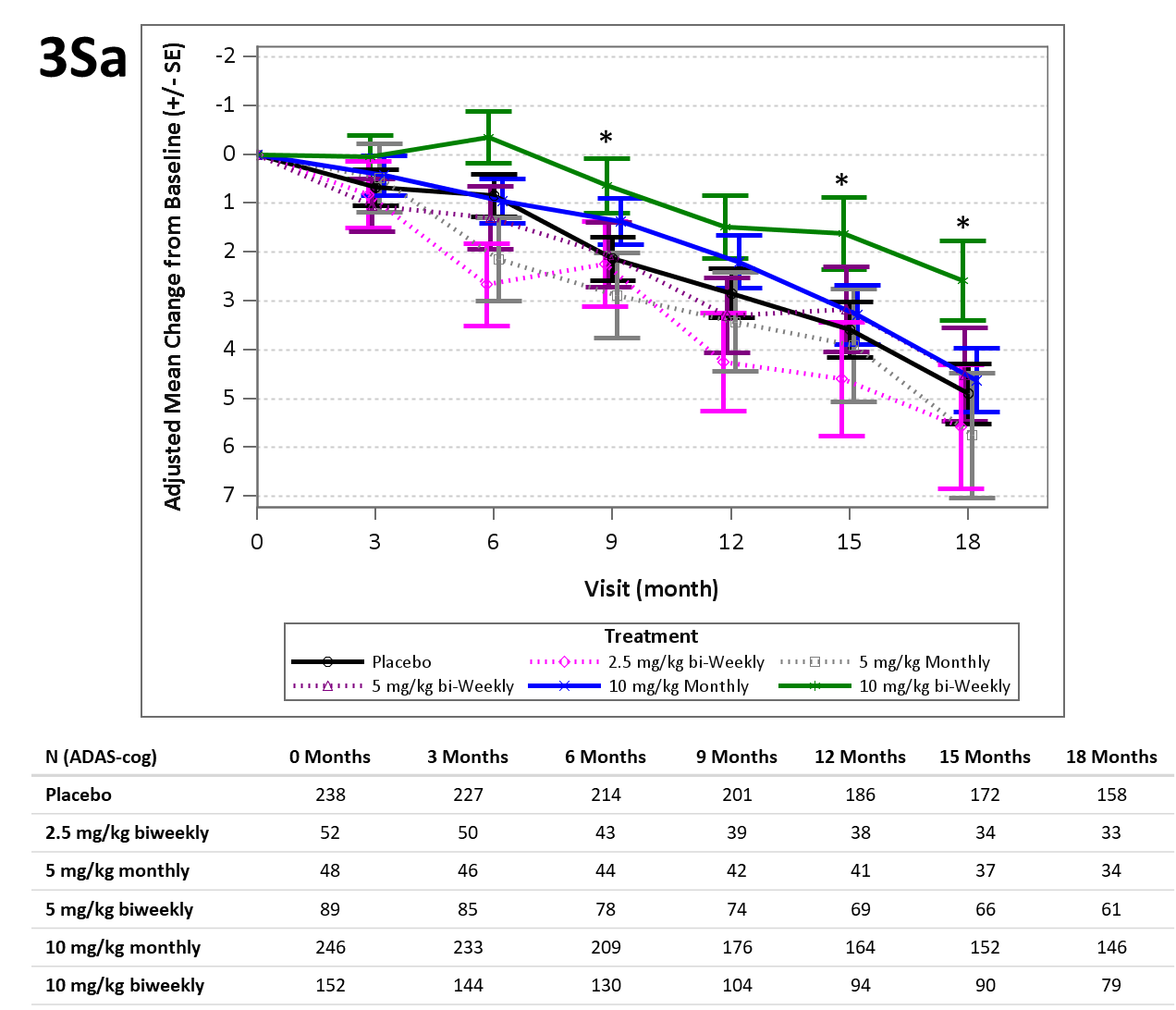


**
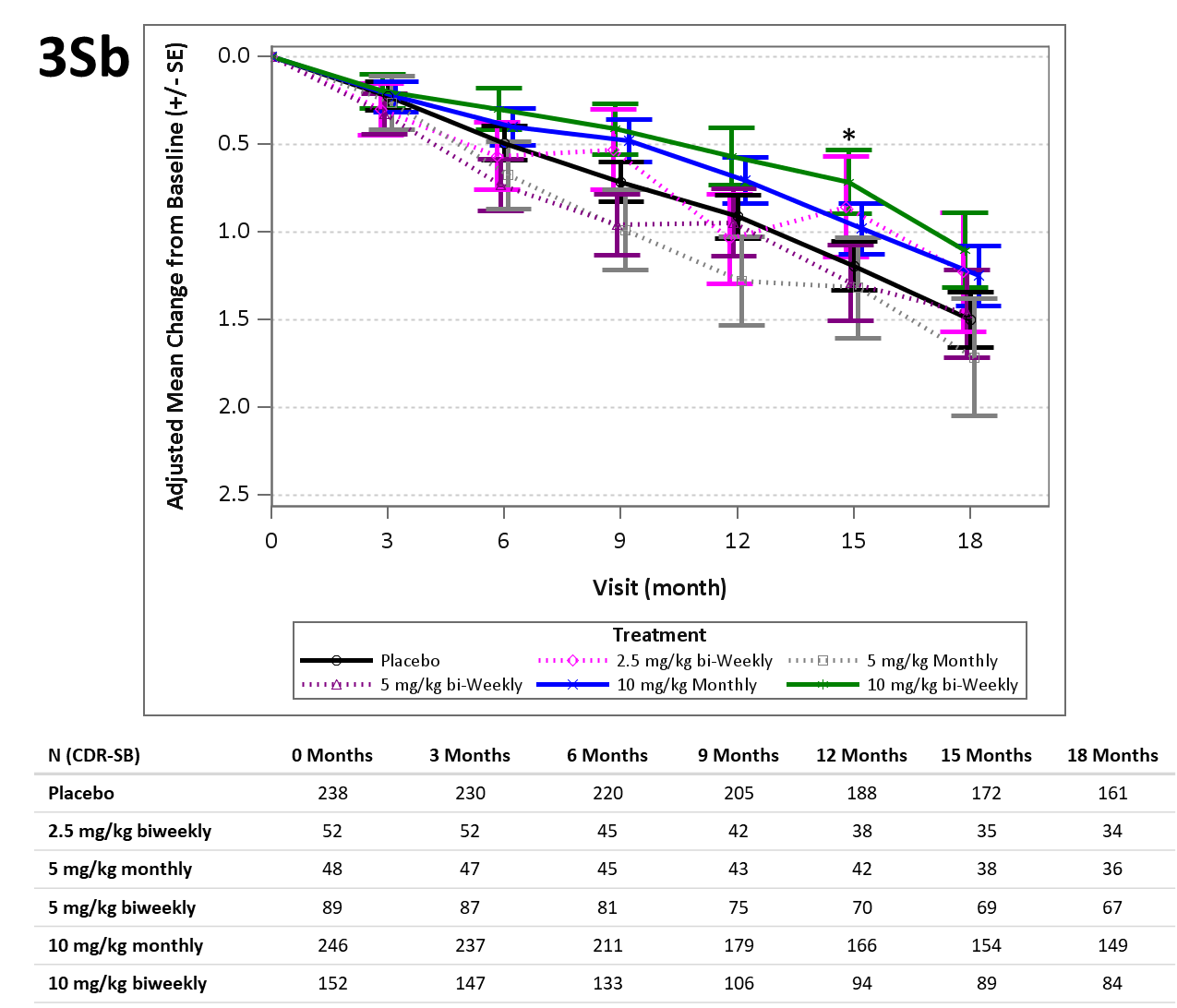
**

**Supplemental Figure S4.** Results for Total Hippocampal Volume (S4A), Whole Brain Volume (S4B), and Ventricular Volume for All Dosing Groups

**Supplemental Figure S4A.**  Change from Baseline for Total Hippocampal Volume (S4A) for All Dosing Groups


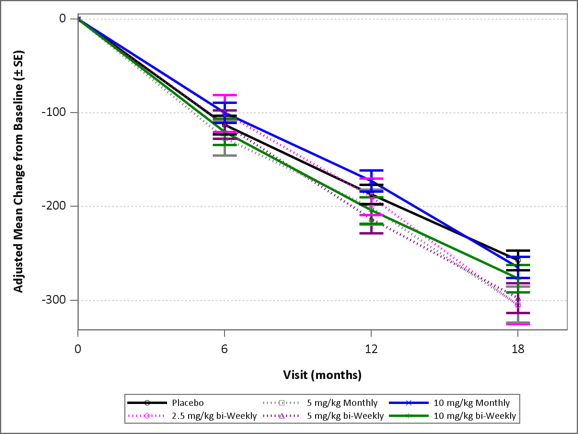


**Supplemental Figure S4B.**  Change from Baseline for Whole Brain Volume (S4B) for All Dosing Groups

**
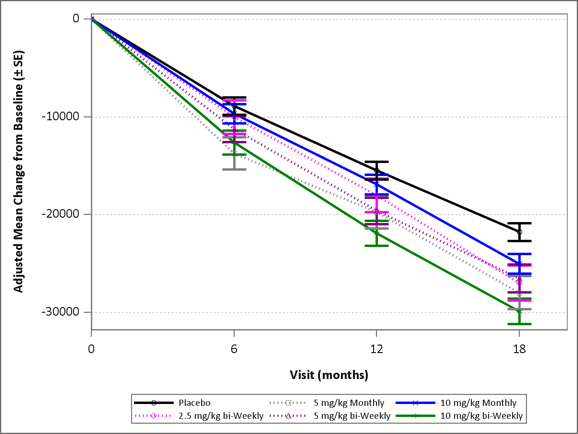
**

**Supplemental Figure S4C.**  Change from Baseline for Ventricular Volume for All Dosing Groups


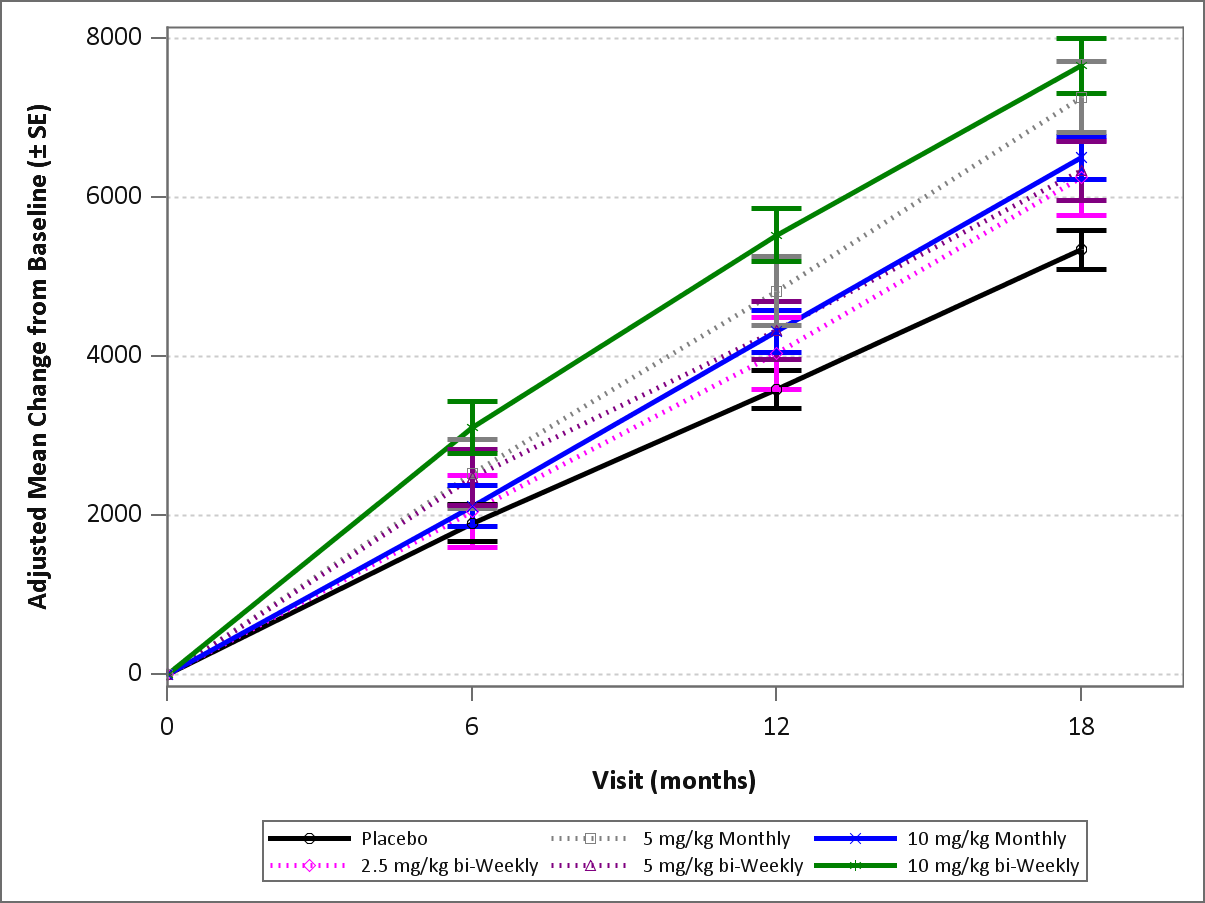


**Supplemental Figure S5.** Change from baseline in neurogranin and neurofilament light chain measures

**Supplemental Figure S5A.** Change from baseline in neurogranin measures. The combined 10 mg/kg monthly and 10 mg/kg biweekly group is compared versus placebo. The number of subjects that were assessed at each time point are indicated in the table. The MMRM used treatment group, visit, clinical subgroup (MCI due to AD, Mild AD), the presence or absence of ongoing AD treatment at baseline, ApoE4 status (positive, negative), region, treatment group-by-visit interaction as factors, and baseline value as covariate.

**P*=0.015

**
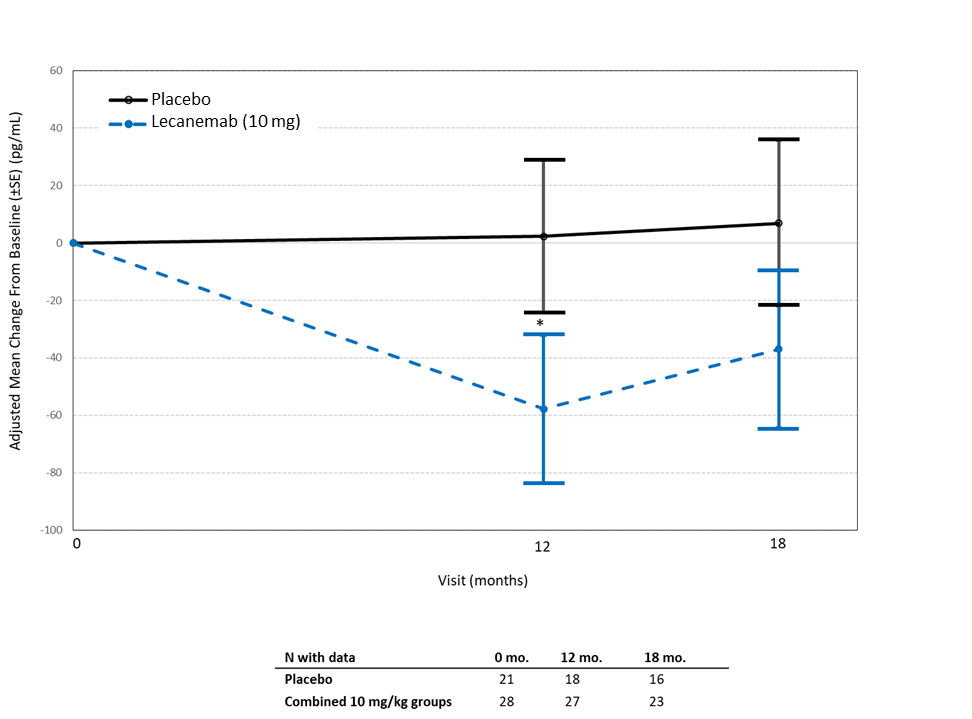
**

**Supplemental Figure S5B.** Change from baseline in neurofilament light chain measures. The combined 10 mg/kg monthly and 10 mg/kg biweekly group is compared versus placebo. The number of subjects that were assessed at each time point are indicated in the table. The MMRM used treatment group, visit, clinical subgroup (MCI due to AD, Mild AD), the presence or absence of ongoing AD treatment at baseline, ApoE4 status (positive, negative), region, treatment group-by-visit interaction as factors, and baseline value as covariate.

**
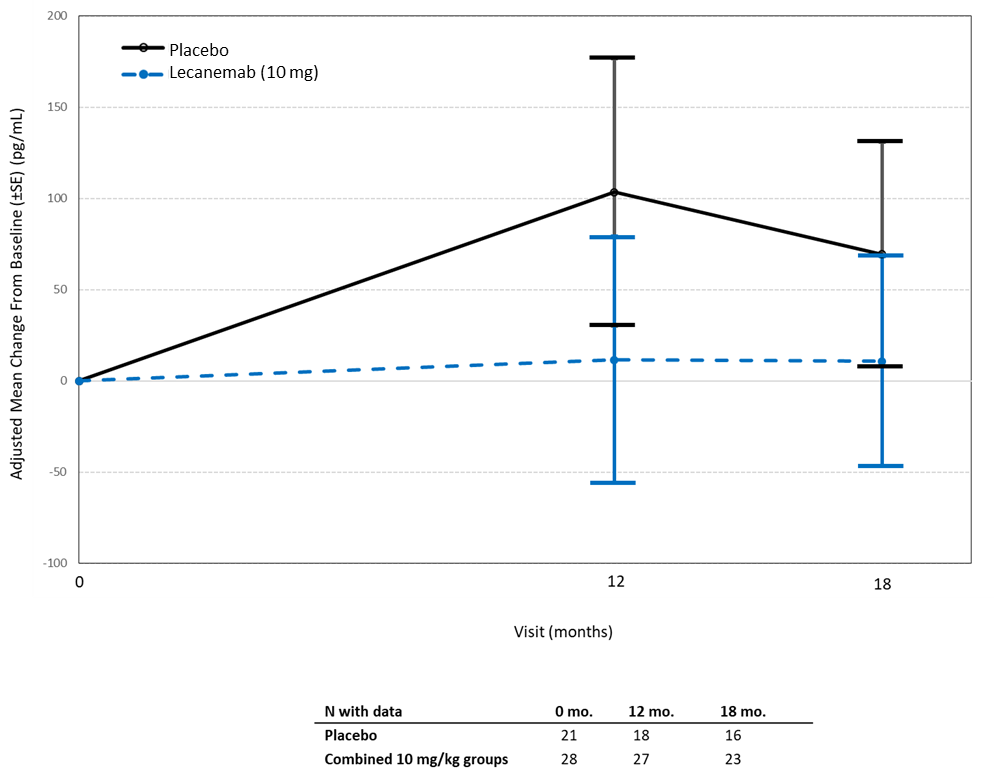
**

**
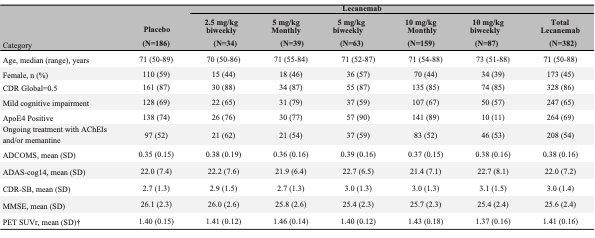
Supplemental Table S1.** Baseline Characteristics for Completers – Full Analysis Set

**Supplemental Table S2.** Baseline Characteristics for Subjects who Discontinued Treatment – Full Analysis Set


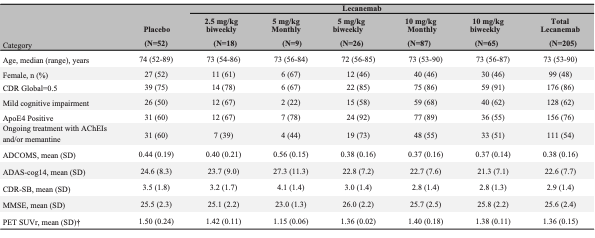


**Supplemental Table S3.** Bayesian Analysis of ADCOMS at 18 Months – Full Analysis Set.

**Supplemental Table S4.** Summary of MMRM Analyses for Change from Baseline in ADCOMS at 12 Months – Full Analysis Set.


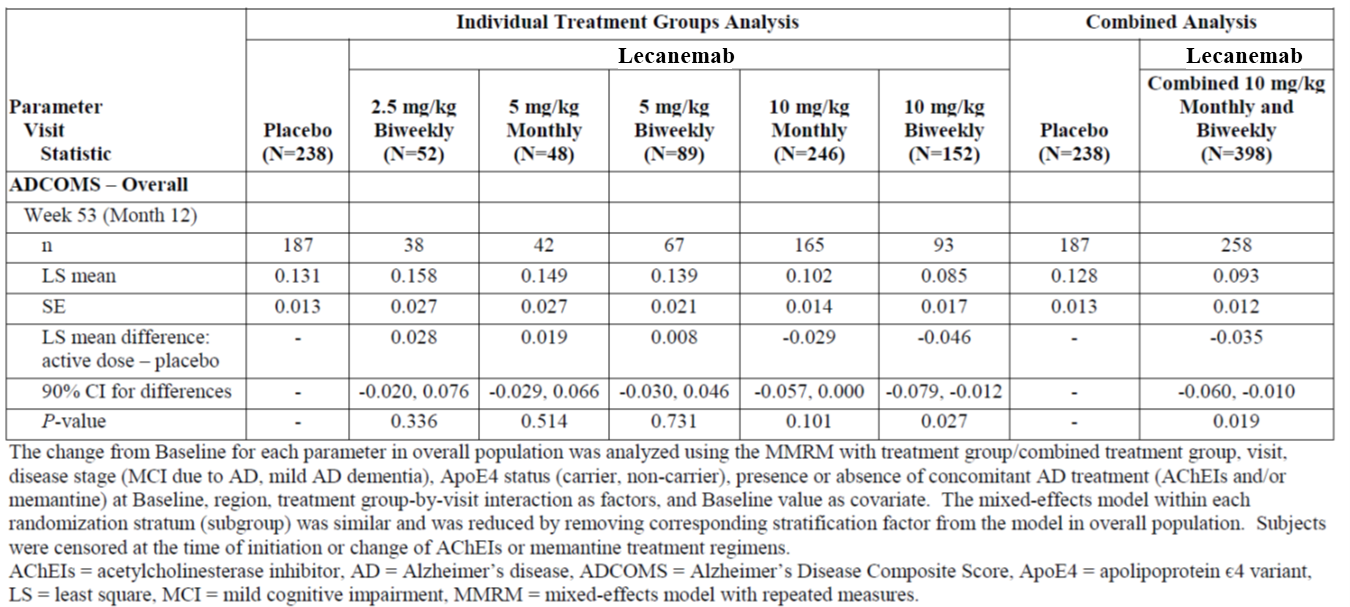


**Supplemental Table S5.** Summary of MMRM Analyses for Change from Baseline in ADCOMS at 18 Months – Full Analysis Set.


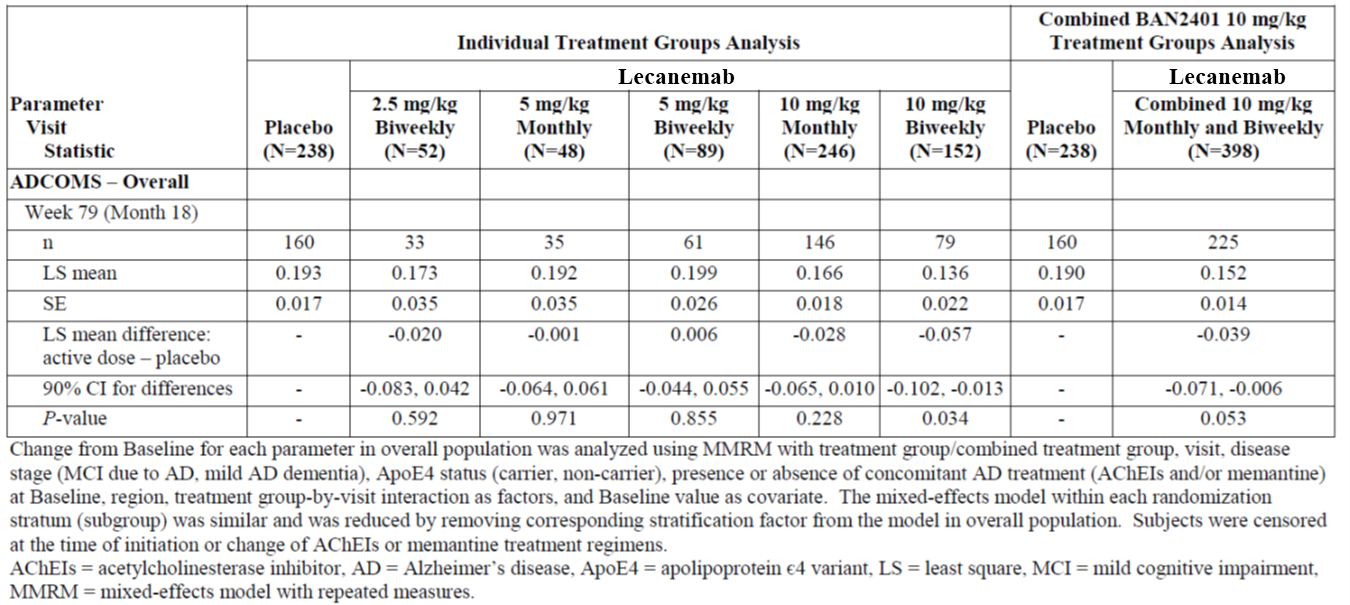


**Supplemental Table S6.** Bayesian Analysis of CDR-SB at 18 Months – Full Analysis Set.

**Supplemental Table S7.** Summary of MMRM Analyses for Change from Baseline in CDR-SB at 18 Months – Full Analysis Set.


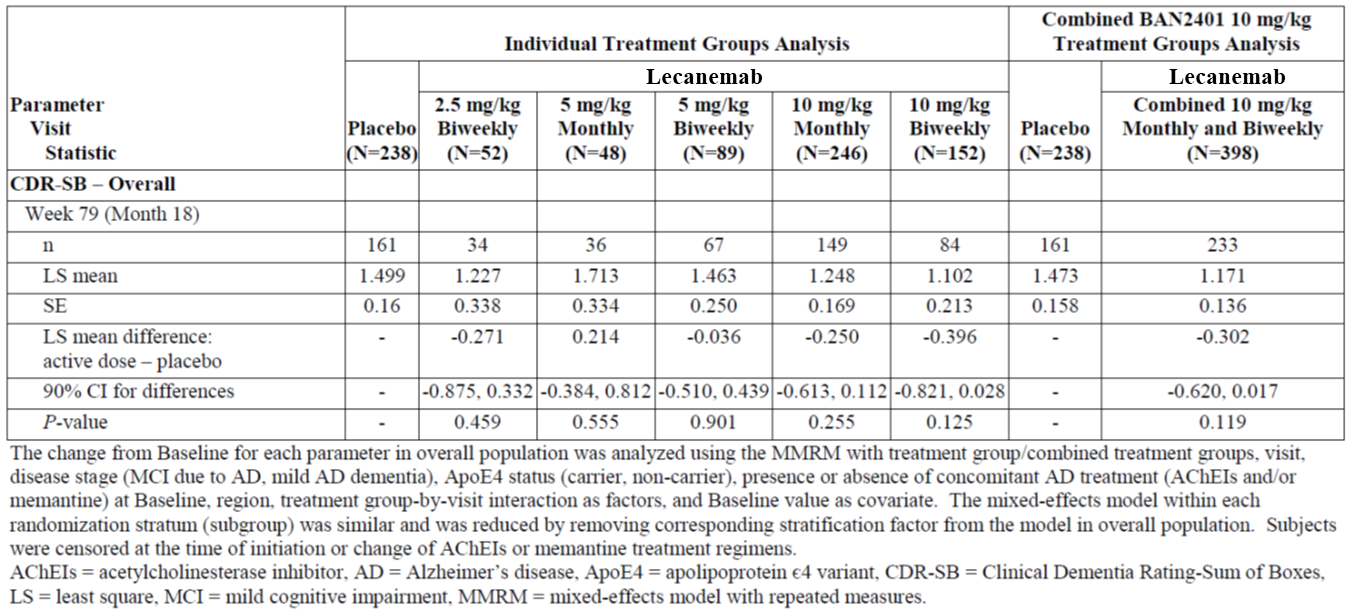


**Supplemental Table S8.** Bayesian Analysis of ADAS-Cog14 at 18 Months – Full Analysis Set.

**Supplemental Table S9.** Summary of MMRM Analyses for Change from Baseline in ADAS-Cog14 at 18 Months – Full Analysis Set.


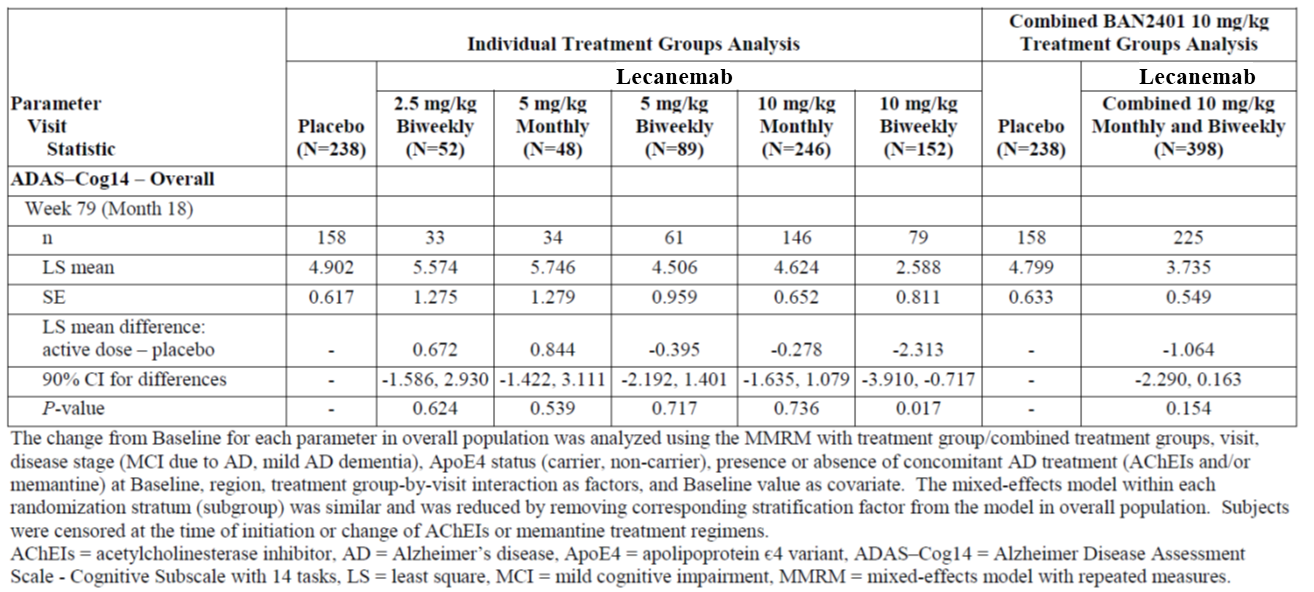


**Supplemental Table S10.** Summary of MMRM Analyses for ADCOMS at 18 Months for Disease Stage (MCI due to AD and Mild AD Dementia) Subgroups - Full Analysis Set

|  | Lecanemab | | | | | |
| --- | --- | --- | --- | --- | --- | --- |
| Visit  Strata Level Statistic | Placebo (N = 238) | 2.5 mg/kg bi-Weekly (N = 52) | 5 mg/kg Monthly (N = 48) | 5 mg/kg bi-Weekly (N = 89) | 10 mg/kg Monthly  (N = 246) | 10 mg/kg bi-Weekly (N = 152) |
| Week 79 |  |  |  |  |  |  |
| MCI due to AD |  |  |  |  |  |  |
| n | 111 | 22 | 30 | 37 | 101 | 47 |
| Least Square Mean | 0.169 | 0.106 | 0.154 | 0.160 | 0.140 | 0.113 |
| SE | 0.018 | 0.037 | 0.034 | 0.029 | 0.019 | 0.025 |
| LS Mean Difference: Active Dose - Placebo |  | -0.062 | -0.014 | -0.008 | -0.029 | -0.056 |
| 90% Confidence Interval for Differences |  | -0.128, 0.004 | -0.076, 0.047 | -0.063, 0.046 | -0.068, 0.010 | -0.105, -0.007 |
| p-value |  | 0.121 | 0.700 | 0.800 | 0.227 | 0.058 |
| Mild AD |  |  |  |  |  |  |
| n | 49 | 11 | 5 | 24 | 45 | 32 |
| Least Square Mean | 0.230 | 0.300 | 0.297 | 0.248 | 0.205 | 0.149 |
| SE | 0.036 | 0.075 | 0.092 | 0.052 | 0.039 | 0.043 |
| LS Mean Difference: Active Dose - Placebo |  | 0.069 | 0.067 | 0.018 | -0.026 | -0.081 |
| 90% Confidence Interval for Differences |  | -0.066, 0.205 | -0.095, 0.229 | -0.084, 0.119 | -0.109, 0.058 | -0.171, 0.009 |
| p-value |  | 0.397 | 0.493 | 0.776 | 0.615 | 0.140 |
| The change from baseline for each parameter in overall population is analyzed using the mixed effects model with repeated measures (MMRM) with treatment group, visit, clinical subgroup (MCI due to AD, Mild Alzheimer's Disease Dementia), the presence or absence of ongoing AD treatment (ie, AChEIs and/or memantine) at baseline, APOE4 status (positive, negative), region, treatment group-by-visit interaction as factors, and baseline value as covariate. The mixed effects model within each randomization stratum (subgroup) is similar and is reduced by removing corresponding stratification factor from the model in overall population.  Subjects are censored at the time of initiation or change of AChEIs or memantine treatment regimens. | | | | | | |

**Supplemental Table S11.** Summary of MMRM Analyses for ADAS–Cog14 at 18 Months for Disease Stage (MCI due to AD and Mild AD Dementia) – Full Analysis Set

|  | Lecanemab | | | | | |
| --- | --- | --- | --- | --- | --- | --- |
| Visit  Strata Level Statistic | Placebo (N = 238) | 2.5 mg/kg bi-Weekly (N = 52) | 5 mg/kg Monthly (N = 48) | 5 mg/kg bi-Weekly (N = 89) | 10 mg/kg Monthly  (N = 246) | 10 mg/kg bi-Weekly (N = 152) |
| Week 79 |  |  |  |  |  |  |
| MCI due to AD |  |  |  |  |  |  |
| n | 112 | 22 | 29 | 37 | 101 | 47 |
| Least Square Mean | 4.633 | 3.572 | 5.168 | 4.042 | 3.630 | 1.925 |
| SE | 0.684 | 1.428 | 1.331 | 1.119 | 0.722 | 0.968 |
| LS Mean Difference: Active Dose - Placebo |  | -1.061 | 0.535 | -0.591 | -1.002 | -2.707 |
| 90% Confidence Interval for Differences |  | -3.571, 1.450 | -1.840, 2.910 | -2.659, 1.477 | -2.486, 0.482 | -4.559, -0.855 |
| p-value |  | 0.486 | 0.710 | 0.638 | 0.266 | 0.016 |
| Mild AD |  |  |  |  |  |  |
| n | 46 | 11 | 5 | 24 | 45 | 32 |
| Least Square Mean | 5.360 | 9.443 | 6.720 | 4.891 | 6.424 | 3.172 |
| SE | 1.216 | 2.469 | 3.054 | 1.734 | 1.294 | 1.426 |
| LS Mean Difference: Active Dose - Placebo |  | 4.083 | 1.360 | -0.469 | 1.063 | -2.188 |
| 90% Confidence Interval for Differences |  | -0.367, 8.532 | -3.980, 6.699 | -3.825, 2.886 | -1.699, 3.825 | -5.170, 0.793 |
| p-value |  | 0.131 | 0.675 | 0.817 | 0.525 | 0.227 |
| The change from baseline for each parameter in overall population is analyzed using the mixed effects model with repeated measures (MMRM) with treatment group, visit, clinical subgroup (MCI due to AD, Mild Alzheimer's Disease Dementia), the presence or absence of ongoing AD treatment (ie, AChEIs and/or memantine) at baseline, APOE4 status (positive, negative), region, treatment group-by-visit interaction as factors, and baseline value as covariate. The mixed effects model within each randomization stratum (subgroup) is similar and is reduced by removing corresponding stratification factor from the model in overall population.  Subjects are censored at the time of initiation or change of AChEIs or memantine treatment regimens. | | | | | | |

**Supplemental Table S12.** Summary of MMRM Analyses for CDR-SB at 18 Months for Disease Stage (MCI due to AD and Mild AD Dementia) – Full Analysis Set

|  | Lecanemab | | | | | |
| --- | --- | --- | --- | --- | --- | --- |
| Visit  Strata Level Statistic | Placebo (N = 238) | 2.5 mg/kg bi-Weekly (N = 52) | 5 mg/kg Monthly (N = 48) | 5 mg/kg bi-Weekly (N = 89) | 10 mg/kg Monthly  (N = 246) | 10 mg/kg bi-Weekly (N = 152) |
| Week 79 |  |  |  |  |  |  |
| MCI due to AD |  |  |  |  |  |  |
| n | 112 | 23 | 30 | 39 | 101 | 49 |
| Least Square Mean | 1.168 | 0.757 | 1.402 | 1.080 | 1.072 | 1.010 |
| SE | 0.162 | 0.341 | 0.313 | 0.266 | 0.171 | 0.230 |
| LS Mean Difference: Active Dose - Placebo |  | -0.411 | 0.233 | -0.089 | -0.096 | -0.159 |
| 90% Confidence Interval for Differences |  | -1.014, 0.192 | -0.331, 0.798 | -0.585, 0.408 | -0.455, 0.262 | -0.603, 0.286 |
| p-value |  | 0.262 | 0.496 | 0.769 | 0.658 | 0.557 |
| Mild AD |  |  |  |  |  |  |
| n | 49 | 11 | 6 | 28 | 48 | 35 |
| Least Square Mean | 2.125 | 2.242 | 2.634 | 1.948 | 1.578 | 1.042 |
| SE | 0.354 | 0.740 | 0.887 | 0.500 | 0.377 | 0.416 |
| LS Mean Difference: Active Dose - Placebo |  | 0.117 | 0.509 | -0.177 | -0.547 | -1.083 |
| 90% Confidence Interval for Differences |  | -1.224, 1.458 | -1.054, 2.072 | -1.166, 0.812 | -1.373, 0.280 | -1.967, -0.198 |
| p-value |  | 0.885 | 0.591 | 0.768 | 0.275 | 0.044 |
| The change from baseline for each parameter in overall population is analyzed using the mixed effects model with repeated measures (MMRM) with treatment group, visit, clinical subgroup (MCI due to AD, Mild Alzheimer's Disease Dementia), the presence or absence of ongoing AD treatment (ie, AChEIs and/or memantine) at baseline, APOE4 status (positive, negative), region, treatment group-by-visit interaction as factors, and baseline value as covariate. The mixed effects model within each randomization stratum (subgroup) is similar and is reduced by removing corresponding stratification factor from the model in overall population.  Subjects are censored at the time of initiation or change of AChEIs or memantine treatment regimens. | | | | | | |

**Supplemental Table S13.** Summary of MMRM Analyses for Change from Baseline in Total Hippocampal Volume at 18 Months – Pharmacodynamic Analysis Set.

**
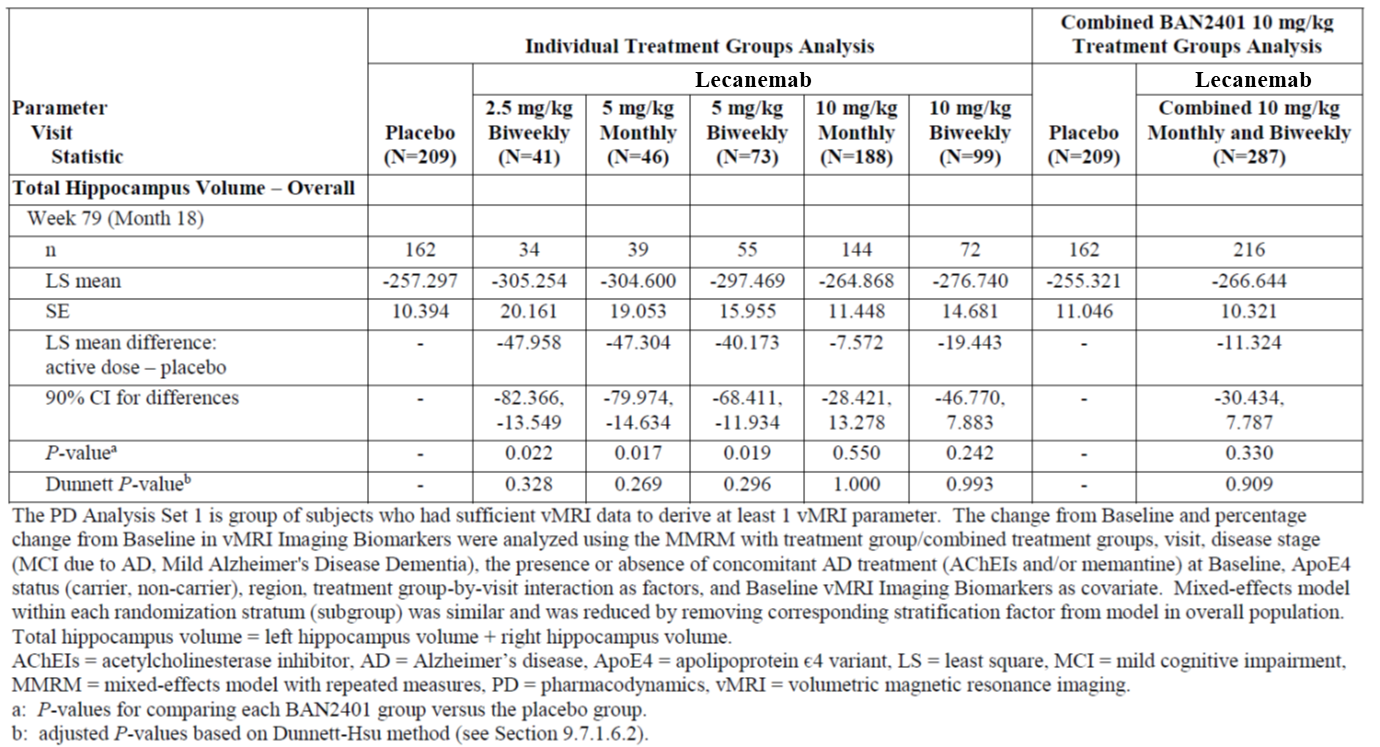
**

**Supplemental Table S14.** Summary of MMRM Analyses for Change from Baseline in vMRI Whole Brain Volume at 18 Months – MMRM Pharmacodynamic Analysis Set.

**
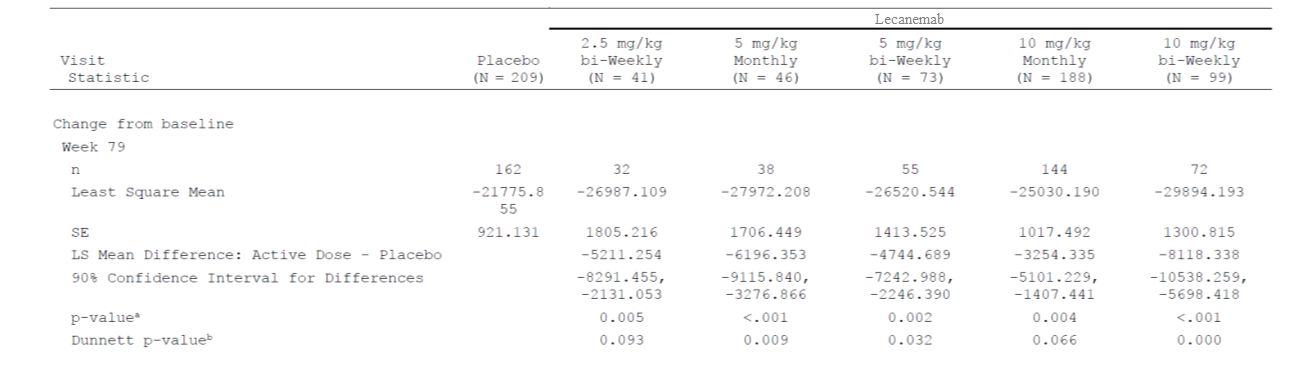
**

**Supplemental Table S15.** Summary of MMRM Analyses for Change from Baseline in vMRI Total Ventricular Volume at 18 Months – MMRM Pharmacodynamic Analysis Set.

**
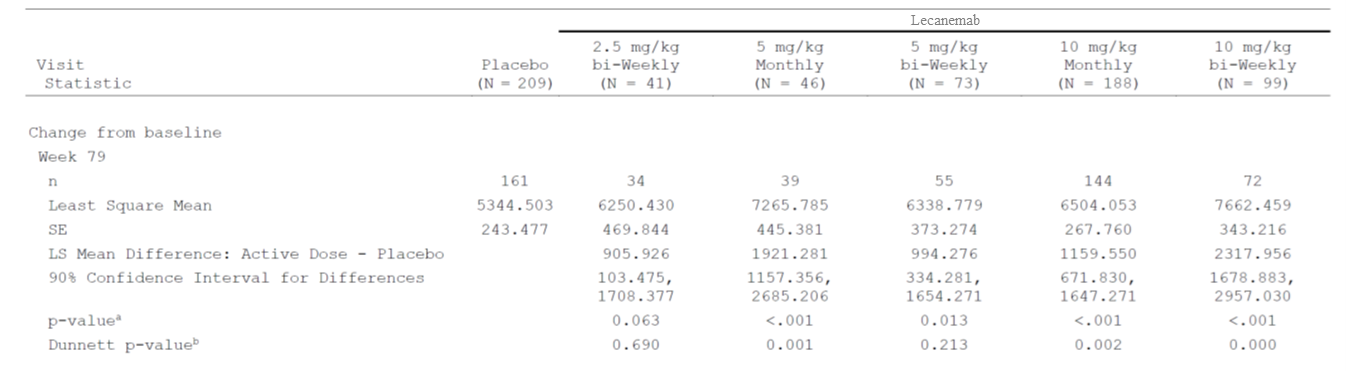
**

**Supplemental Table S16.** Bayesian Analysis of ADCOMS at 18 Months for ApoE4 Genotype (Carrier or Non-Carrier) Subgroups - Full Analysis Set.

| **ADCOMS** |  | **Change From Baseline** | | **Difference From Control** | | **Ratio to Control** | | **Posterior Quantities** | | | |
| --- | --- | --- | --- | --- | --- | --- | --- | --- | --- | --- | --- |
| **Strata Subgroup  Treatment Group** | **Total N** | **Mean** | **95% CI** | **Mean** | **95% CI** | **Mean** | **95% CI** | **Pr (Max)** | **Pr (ED_90_)** | **Pr Superiority** | **Pr (CSD)** |
| **ApoE4 Carriers** | | | | | | | | | | | |
| Placebo | 168 | 0.180 | (0.144, 0.216) | - | - | - | - | - | - | - | - |
| 2.5 mg/kg biweekly | 38 | 0.149 | (0.085, 0.213) | -0.031 | (-0.105, 0.042) | 0.834 | (0.458, 1.262) | 0.090 | 0.106 | 0.804 | 0.515 |
| 5 mg/kg monthly | 37 | 0.155 | (0.098, 0.214) | -0.026 | (-0.092, 0.043) | 0.867 | (0.529, 1.268) | 0.048 | 0.072 | 0.778 | 0.452 |
| 5 mg/kg biweekly | 81 | 0.158 | (0.116, 0.202) | -0.023 | (-0.078, 0.034) | 0.884 | (0.613, 1.215) | 0.012 | 0.013 | 0.789 | 0.400 |
| 10 mg/kg monthly | 218 | 0.139 | (0.108, 0.171) | -0.041 | (-0.089, 0.006) | 0.780 | (0.565, 1.040) | 0.058 | 0.080 | 0.956 | 0.679 |
| 10 mg/kg biweekly | 45 | 0.096 | (0.027, 0.154) | -0.084 | (-0.161, -0.015) | 0.537 | (0.148, 0.905) | 0.792 | 0.728 | 0.992 | 0.936 |
| **ApoE4 non-carriers** | | | | | | | | | | | |
| Placebo | 70 | 0.146 | (0.092, 0.201) | - | - | - | - | - | - | - | - |
| 2.5 mg/kg biweekly | 14 | 0.154 | (0.068, 0.243) | 0.008 | (-0.094, 0.111) | 1.095 | (0.442, 2.000) | 0.209 | 0.209 | 0.442 | 0.230 |
| 5 mg/kg monthly | 11 | 0.149 | (0.074, 0.226) | 0.002 | (-0.090, 0.096) | 1.058 | (0.476, 1.888) | 0.191 | 0.215 | 0.481 | 0.241 |
| 5 mg/kg biweekly | 8 | 0.161 | (0.092, 0.245) | 0.014 | (-0.074, 0.113) | 1.143 | (0.581, 2.000) | 0.082 | 0.076 | 0.386 | 0.166 |
| 10 mg/kg monthly | 28 | 0.143 | (0.082, 0.205) | -0.003 | (-0.085, 0.079) | 1.018 | (0.518, 1.751) | 0.218 | 0.226 | 0.531 | 0.257 |
| 10 mg/kg biweekly | 107 | 0.135 | (0.095, 0.174) | -0.011 | (-0.079, 0.056) | 0.960 | (0.577, 1.566) | 0.300 | 0.274 | 0.630 | 0.290 |
| Only subjects with non-missing data at both Baseline and the relevant post-Baseline visit are included in the change from Baseline summary statistics.  ApoE4 = apolipoprotein є4 variant, CSD = clinically significant difference, ED_90_ = dose regimen with at least 90% of the d_max_ treatment effect, Max = maximum, Pr = probability.  Source: Appendix 16.1.9 | | | | | | | | | | | |

**Supplemental Table S17.** Sensitivity Analyses for Efficacy Assessments.

| **Efficacy Assessment** | **Sensitivity Analysis Results Summary** |
| --- | --- |
| ADCOMS | The results for the ANCOVA analysis of change from Baseline at 18 months in ADCOMS in the 10 mg/kg biweekly group versus placebo using multiple imputations (Full Analysis Set) were similar in direction and magnitude to the MMRM  analyses. The LS mean (SE) change from Baseline was 0.198 (0.022) for the 10 mg/kg biweekly group compared to 0.139 (0.029) for placebo. The LS mean difference of 10 mg/kg biweekly from placebo was -0.059 (*P*=0.036; 90% CI: -0.115, -0.004).  The results for the ANCOVA analysis of change from Baseline at 18 months in ADCOMS in the 10 mg/kg biweekly group versus placebo using control-based multiple imputations (Full Analysis Set) were similar in direction and magnitude to previous analyses. The LS mean (SE) change from Baseline was 0.190 (0.022) for the 10 mg/kg biweekly group compared to 0.147 (0.027) for placebo. The LS mean difference of 10 mg/kg biweekly from placebo was -0.043 (*P*=0.097; 90% CI: -0.093, -0.008). |
| CDR-SB | The results for the ANCOVA analysis of change from Baseline at 18 months in CDR-SB in the 10 mg/kg biweekly group versus placebo using multiple (Full Analysis Set) show the LS mean (SE) change from Baseline was 1.225 (0.300) for the 10 mg/kg biweekly group compared to 1.591 (0.224) for placebo. The LS mean difference of 10 mg/kg biweekly from placebo was -0.366 (*P*=0.220; 90% CI: -0.950, 0.219).  The ANCOVA analysis of change from Baseline at 18 months in CDR-SB in the 10 mg/kg biweekly group versus placebo using control-based multiple imputations (Full Analysis Set) shows the LS mean (SE) change from Baseline was 1.268 (0.265) for the 10 mg/kg biweekly group compared to 1.508 (0.216) for placebo. The LS mean difference of 10 mg/kg biweekly from placebo was -0.240 (*P*=0.347; 90% CI: -0.740, 0.260). |
| ADAS-Cog | The results for the ANCOVA analysis of change from Baseline at 18 months in ADAS–Cog14 in the 10 mg/kg biweekly group versus placebo using multiple imputations (Full Analysis Set) shows the LS mean (SE) change from Baseline was 2.848 (1.121) for the 10 mg/kg biweekly group compared to 5.686 (0.883) for placebo. The LS mean difference of 10 mg/kg biweekly from placebo was -2.838 (*P*=0.010; 90% CI: - 4.990, -0.686).  The results for the ANCOVA analysis of change from Baseline at 18 months in ADAS–Cog14 in the10 mg/kg biweekly group versus placebo using control-based multiple imputations (Full Analysis Set) shows the LS mean (SE) change from Baseline was 3.464 (1.070) for the 10 mg/kg biweekly group compared to 5.517 (0.873) for placebo. The LS mean difference of 10 mg/kg biweekly from placebo was -2.052 (*P*=0.042; 90% CI: -4.028, -0.077). |

**Supplementary Appendix A:** Study Protocol

See protocol provided in separate document.

**Supplementary Appendix B:** Simulation Plan.

See Appendix 13.2 of Statistical Analysis Plan provided in separate document.

**Supplementary Appendix C:** Additional Detail on Regulatory Authority Requested Changes to Allocation of APOE4+ Subjects.

During the study, the data safety monitoring board (DSMB) monitored the overall safety profile of lecanemab with attention directed to events of ARIA. Early in the study conduct (between 300 and 350 subject randomized), following a regularly scheduled DSMB meeting which included review of reported events of ARIA (including 3 symptomatic cases in APOE4+ homozygous subjects at 10 mg/kg biweekly, suggesting that ApoE4 homozygous individuals on 10 mg/kg biweekly have the highest risk of developing symptomatic ARIA–E), the DSMB recommended that newly enrolled ApoE4 homozygous subjects should no longer be dosed at 10 mg/kg biweekly, and to amend the study design by adding a Week 9 MRI scan. This recommendation was adopted. In parallel, cumulative ARIA safety data from Study 201 were requested by one Regulatory Authority. All 8 subjects with ARIA-E at the time of review were APOE4 carriers, with 5/8 ARIA-E cases occurring at the top dose of 10 mg/kg biweekly. Ex US Regulatory Authorities asked the study sponsor to stop randomizing all ApoE4 carriers (heterozygous and homozygous) to 10 mg/kg biweekly in all countries, to discontinue all ApoE4 carrier subjects already randomized to the 10 mg/kg biweekly dose with less than 6 months of exposure to lecanemab (which included 25 subjects with no symptoms and no ARIA- E) and to institute a recruitment suspension at all European sites to enable the collection of additional safety data. These changes were subsequently adopted. Following a scientific advice meeting and a Clarification Meeting with the Expert Advisory Group (EAG) after additional safety reviews, the study was allowed to resume randomization in Europe (according to all related protocol changes) with the inclusion of an additional safety MRI scan at week 7. Randomization ratios before and after the interim analysis with 350 subjects are presented below in **Supplementary**

**Appendix C** **Table 1 and Table 2**, respectively, highlighting the impact of the design change on randomization allocations.

**Supplementary Appendix C Table 1**: Randomization Ratios Per Dose By Interim Analysis Prior to Design Changes Related to non-US Health Authority Input

| **No. Subjects Enrolled by Each Interim Analysis** | **Placebo** | **2.5 mg/kg Biweekly** | **5 mg/kg Monthly** | **5 mg/kg Biweekly** | **10 mg/kg Monthly** | **10 mg/kg Biweekly** |
| --- | --- | --- | --- | --- | --- | --- |
| 196 | 0.2412 | 0.1267 | 0.1593 | 0.0327 | 0.1989 | 0.2412 |
| 250 | 0.2327 | 0.1874 | 0.0946 | 0.0367 | 0.2327 | 0.2159 |
| 300 | 0.2703 | 0.0272 | 0.0548 | 0.0205 | 0.1902 | 0.4370 |

| **Supplementary Appendix C Table 2**: Randomization Ratios Per Dose By Interim Analysis After Design Changes Related to non-US Health Authority Input | | | | | | | | |
| --- | --- | --- | --- | --- | --- | --- | --- | --- |
| **No. Subjects Enrolled by Each Interim Analysis** | **Placebo** | **2.5 mg/kg Biweekly** | **5 mg/kg Monthly** | **5 mg/kg Biweekly** | **10 mg/kg Monthly** | **10 mg/kg Biweekly** |  |  |
| **ApoE4 Carriers** | | | | | | | |  |
| 350 | 0.2914 | 0.0124 | 0.0617 | 0.0080 | 0.6265 | 0.0000 |  |  |
| 400 | 0.3013 | 0.0081 | 0.0079 | 0.0073 | 0.6754 | 0.0000 |  |  |
| 450 | 0.3034 | 0.0050 | 0.0031 | 0.0247 | 0.6638 | 0.0000 |  |  |
| 500 | 0.2947 | 0.0075 | 0.0062 | 0.0971 | 0.5945 | 0.0000 |  |  |
| 550 | 0.3019 | 0.0087 | 0.0114 | 0.0812 | 0.5968 | 0.0000 |  |  |
| 600 | 0.2858 | 0.0167 | 0.0204 | 0.1842 | 0.4929 | 0.0000 |  |  |
| 650 | 0.2802 | 0.0173 | 0.0266 | 0.2447 | 0.4312 | 0.0000 |  |  |
| 700 | 0.2842 | 0.0263 | 0.0272 | 0.2291 | 0.4332 | 0.0000 |  |  |
| 750 | 0.2663 | 0.0183 | 0.0310 | 0.4063 | 0.2781 | 0.0000 |  |  |
| 800 | 0.2759 | 0.0330 | 0.0640 | 0.2585 | 0.3686 | 0.0000 |  |  |
| **ApoE4 Non-Carriers** | | | | | | | |  |
| 350 | 0.2914 | 0.0000 | 0.0000 | 0.0000 | 0.0000 | 0.7086 |  |  |
| 400 | 0.3013 | 0.0030 | 0.0029 | 0.0027 | 0.2530 | 0.4371 |  |  |
| 450 | 0.3034 | 0.0020 | 0.0013 | 0.0100 | 0.2688 | 0.4145 |  |  |
| 500 | 0.2947 | 0.0000 | 0.0000 | 0.0000 | 0.0000 | 0.7053 |  |  |
| 550 | 0.3019 | 0.0000 | 0.0000 | 0.0000 | 0.0000 | 0.6981 |  |  |
| 600 | 0.2858 | 0.0000 | 0.0000 | 0.0000 | 0.0000 | 0.7142 |  |  |
| 650 | 0.2802 | 0.0000 | 0.0000 | 0.0000 | 0.0000 | 0.7198 |  |  |
| 700 | 0.2842 | 0.0000 | 0.0000 | 0.0000 | 0.0000 | 0.7158 |  |  |
| 750 | 0.2663 | 0.0000 | 0.0000 | 0.0000 | 0.0000 | 0.7337 |  |  |
| 800 | 0.2759 | 0.0000 | 0.0000 | 0.0000 | 0.0000 | 0.7241 |  |  |
| ApoE4 = apolipoprotein є4 variant | | | | | | | |  |

**Supplementary Appendix D:** Principal Investigators from Participating Enrolling Centers.

United States: Craig Curtis, BioClinica Orlando; Marina Raikhel, Torrance Clinical Research Institute, Inc; Omid Omidvar, Collaborative Neuroscience Network, LLC; John Stoukides, Rhode Island Mood & Memory Research Institute; Rober Riesenberg, Atlanta Center for Medical Research; Mark Brody, Brain Matters Research Inc.; William Smith, New Orleans Center for Clinical Research; Martin Farlow, Indiana University School of Medicine; Keith Edwards, Empire Neurology, PC; Danna Jennings, Institute for Neurodegenerative Disorders; Meenakshi Patel, Valley Medical Primary Care; Allan Levey, Emory University Cognitive Neurology Clinic & ADRC; Christopher Van Dyck, Yale University School of Medicine; Horacio Capote, Dent Neurologic Institute; Donald Royall, The University of Texas Health Science Center at San Antonio; Sanjiv Sharma, Advanced Memory Research Institute of NJ PC; Geoffrey Ahern, University of Arizona; James Sutton, Pacific Neuroscience Medical Group; David Hart, The Neurology Group, LLP; Robert Stern, Boston University School of Medicine; M. Saleem Ismail, Psychiatry and Alzheimer’s Care of Rochester, PLLC; David Weisman, Abington Neurological Associates; John DeQuardo, Synexus Clinical Research US, Inc.; Jose Gamez, Galiz Research, LLC. John Nardandrea, Renstar Medical Research; Djamchid Lotfi, Clinical Trial Network; Amanda Smith, USF Suncoast Gerontology Center; Michael Downing, FutureSearch Trials of Dallas, LP; Yuval Zabar, Lahey Clinic Inc; William McElveen, Bradenton Research Center Inc.; Elias Granadillo Deluque, Medical College of Wisconsin; Aaron Ellenbogen, Quest Research Institute; Marshall Nash, NeuroStudies.net, LLC; Richard Singer, Infinity Clinical Research, LLC; Frederick Schaerf, Neuropsychiatric Research Center; Concetta Forchetti, Alexian Bro Neuro Institute; Mark Fisher, Lynn Health Science Institute; Scott Losk, Summit Research Network (Oregon), Inc.; Jaron Winston, Senior Adult Specialty Research; Nancy Barbas, University of Michigan Hospital; Nader Oskooilar, Pharmacology Research Institute; Mary Stedman, Stedman Clinical Trials, LLC; Jonathan Liss, Columbus Regional Medical Center; Cynthia Murphy, The Memory Clinic; Andrea Bozoki, Michigan State University; Gregory Jicha, University of Kentucky Health Center; John Scott, National Clinical Research Inc (Richmond); Marc Agronin, Miami Jewish Health System; Stephen Flitman, Xenoscience Inc.; Joel Ross, Memory Enhancement Center of America, Inc.; Michael Tuchman, Palm Beach Neurological Center; Matthew Macaluso, Kansas University Medical Center Research Institute; Jose De La Gandara, Quatum Laboratories Inc.; Anton Porteinsson, University of Rochester; Thomas Vidic, Elkhart Clinic; Gad Marshall, Brigham and Women’s Hospital; Jeffrey Kaye, Oregon Health & Sciences University; David Geldmacher; Uuniversity of Alabama at Birmingham; Mark Goldstein, JEM Research Institute; Michael Lin, Weill Cornell Medical College New York Presbyterian Hospital; David Watson, Alzheimer’s Research and Treatment Center; David Subich, BioClinica The Villages; Steven Potkin, The Regents of the University of California; Mark Hernandez, Berma Research Group; Gary Tunell, Texas Neurology, PA; Joseph Rodd, American Neuropsychiatric Research Institute Inc.; Fernando Salvato, CCM Clinical Research Group Inc; Michael Plopper, Sharp Mesa Vista Hospital. Misael Gonzalez, Doctors Research Institute. Mohammad Bolouri; ANI Neurology, PLLC dba Alzheimer’s Memory Center.

Canada: Alain Robillard, opital Maisonneuve-Rosemont d/b/a CIUSSS de l'Est-de-l'Île-de-Montréal; Ziad Nasreddine, Neuro Rive Sud Clinic/Center for Diagnosis & Research on Alzheimer's Disease; Sharon Cohen, Toronto Memory Program, Neurology Research, Inc.; Jennie Wells, St. Joseph’s Health Care, Parkwood Institute; Jennifer Ingram, Kawartha Centre; Tilak Mendis, Parkinson’s and Neurodegenerative Disorders Clinic.

United Kingdom: Fraser Inglis, Glasgow Memory Clinic Ltd.

France: Jacques Hugon, Hopital Lariboisiere.

Germany: Peter Franz, Praxis Dr. med. Volker Schumann; Matthias Riepe, Bezirkskrankenhaus Guenzburg; Volker Schumann, Praxis Dr. med. Volker Schumann.

Italy: Ubaldo Bonuccelli, Azienda Ospedaliero Universitaria Pisana; Carlo De Lena, Umberto I Pol. di Roma-Università di Roma La Sapienza; Flavio Nobili, Azienda Ospedaliero Universitaria San Martino; Elio Scarpini, Fondazione IRCCS CA' Granda Ospedale Maggiore Policlinico; Orazio Zanetti, IRCCS Centro San Giovanni di Dio Fatebenefratelli; Carlo Caltagirone, Fondazione Santa Lucia IRCCS.

Netherlands: Niels Prins, Brain Research Center.

Spain: Fernandez Felix Vinuela Fernandez, Hospital Universitario Virgen Macarena; Gurutz Linazasoro Cristobal , Policlinica Guipuzcoa; Ernest Martinez  Balaguer, Hospital General de Catalunya; Merce Boada Rovira, Fundacio ACE; Teresa Moreno Ramos, Hospital Universitario Clinico San Carlos; Jesus Lopez Arrieta, Hospital de Cantoblanco.

Sweden: Martin Ingelsson, Akademiska Sjukhuset; Henrik Östlund, Skånes Universitetssjukhus; Michael Jonsson, Sahlgrenska Universitetssjukhuset, Mölndal Sjukhus.

Japan: Tatsushi Toda, Kobe University Hospital; Hiroo Yoshikawa, Hyogo College of Medicine; Hirotaka Nagashima, Shinjuku Research Park Clinic; Sadao Katayama, Katayama Medical Clinic; Higashi Yasuto, Himeji Central Hospital s Clinic; Chigusa Watanabe, NHO Hiroshima-Nishi Medical Center; Mitsunori Ishikawa, Ishikawa Clinic; Hirofumi Sakurai, Tokyo Medical University Hospital; Renpei Sengoku, Tokyo Metropolitan Geriatric Hospital; Yuichi Maruki, Synapse Saitama Neuropsychiatry Center; Takao Takeshima, Kotobukikai Tominaga Hopital; Satoru Kosaka, Saneikai Tsukazaki Hospital; Akira Terashima, Hyogo Brain and Heart Center.

Republic of Korea: Sang Yun Kim, Seoul National University Bundang Hospital; Jae-Hong Lee, Asan Medical Center; Kyung Won Park, Dong A University Hospital; Seung-Ho Ryu, Konkuk University Medical Center; Young Ho Sohn, Severance Hospital Yonsei University.
