# Supplementary material for: A randomized, double-blind, phase 2b proof-of-concept clinical trial in early Alzheimer’s disease with lecanemab, an anti-Aβ protofibril antibody
Source: Alzheimers Res Ther. 2021 Apr 17;13:80. doi: 10.1186/s13195-021-00813-8 (PMC8053280; doi:10.1186/s13195-021-00813-8)
Supplement: Supplementary file 1 — Additional file 1: Supplemental Figure S1. CONSORT 2010 Flow Diagram. Supplemental Figure S2. Change from Baseline for all Treatment Groups in the Alzheimer's Disease Composite Score (ADCOMS). Supplemental Figure S3. Results for ADAS-cog (3SA) and CDR-SB (3SB) for All Dosing groups. Supplemental Figure S4. Results for Total Hippocampal Volume (S4A), Whole Brain Volume (S4B), and Ventricular Volume for All Dosing Groups (S4C). Supplemental Figure S5. Change from Baseline in Neurogranin Measures (5SA). Change from Baseline in Neurofilament Light Chain Measures (5SB). Supplemental Table S1. Baseline Characteristics for Completers – Full Analysis Set. Supplemental Table S2. Baseline Characteristics for Subjects who Discontinued Treatment – Full Analysis Set. Supplemental Table S3. Bayesian Analysis of ADCOMS at 18 Months – Full Analysis Set. Supplemental Table S4. Summary of MMRM Analyses for Change from Baseline in ADCOMS at 12 Months – Full Analysis Set. Supplemental Table S5. Summary of MMRM Analyses for Change from Baseline in ADCOMS at 18 Months – Full Analysis Set. Supplemental Table S6. Bayesian Analysis of CDR-SB at 18 Months – Full Analysis Set. Supplemental Table S7. Summary of MMRM Analyses for Change from Baseline in CDR-SB at 18 Months – Full Analysis Set. Supplemental Table S8. Bayesian Analysis of ADAS-Cog14 at 18 Months – Full Analysis Set. Supplemental Table S9. Summary of MMRM Analyses for Change from Baseline in ADAS-Cog14 at 18 Months – Full Analysis Set. Supplemental Table S10. Summary of MMRM Analyses for ADCOMS at 18 Months for Disease Stage (MCI due to AD and Mild AD Dementia) Subgroups - Full Analysis Set. Supplemental Table S11. Summary of MMRM Analyses for ADAS–Cog14 at 18 Months for Disease Stage (MCI due to AD and Mild AD Dementia) – Full Analysis Set. Supplemental Table S12. Summary of MMRM Analyses for CDR-SB at 18 Months for Disease Stage (MCI due to AD and Mild AD Dementia) – Full Analysis Set. Supplemental Table S13. Summary of MMRM Ana [file 13195_2021_813_MOESM1_ESM.zip › BAN2401-G000-201--protocol.pdf]

1 TITLE PAGE

CLINICAL STUDY PROTOCOL

**Study Protocol Number:**	BAN2401-G000-201
**Study Protocol Title:**	A Placebo-Controlled, Double-Blind, Parallel-Group, Bayesian Adaptive Randomization Design and Dose Regimen-finding Study with an Open-Label Extension Phase to Evaluate Safety, Tolerability and Efficacy of BAN2401 in Subjects With Early Alzheimer's Disease
**Sponsor:**	Eisai Inc.<br>155 Tice Boulevard<br>Woodcliff Lake,<br>New Jersey 07677<br>US<br><br>Eisai Ltd.<br>European Knowledge Centre<br>Mosquito Way<br>Hatfield, Hertfordshire<br>AL10 9SN UK<br><br>Eisai Co., Ltd.<br>4-6-10 Koishikawa<br>Bunkyo-Ku,<br>Tokyo 112 8088<br>Japan<br>(revised per Amendment 07)
**Investigational Product Name:**	BAN2401
**Indication:**	Alzheimer's disease
**Phase:**	Phase 2
**Approval Date:**	v2.0, 20 Nov 2012 (Original Protocol)<br>v3.0, 22 Apr 2013 (Amendment 01)<br>v4.0, 27 Jun 2013 (Amendment 02)<br>v5.0, 18 Sep 2013 (Amendment 03)<br>v6.0, 09 Jul 2014 (Amendment 04)<br>v7.0, 11 Aug 2014 (Amendment 05)<br>v8.0, 20 Nov 2014 (Amendment 06)<br>v9.0, 26 Jun 2015 (Amendment 07)<br>v10.0, 30 Jul 2015 (Amendment 07)<br>v11.0, 18 Feb 2016 (Amendment 08)<br>v12.0, 09 Nov 2017 (Amendment 09)<br>v13.0, 16 Mar 2018 (Amendment 10)<br>v14.0, 14 Sep 2018 (Amendment 11)<br>v15.0, 15 Nov 2018 (Amendment 12)
**IND Number:**	105081
**EudraCT Number:**	2012-002843-11
**GCP Statement:**	This study is to be performed in full compliance with International Conference on Harmonisation of Technical Requirements for Registration of Pharmaceuticals for Human Use (ICH) and all applicable local Good Clinical Practice (GCP) and regulations. All required study documentation will be archived as required by regulatory authorities.
**Confidentiality Statement:**	This document is confidential. It contains proprietary information of Eisai (the sponsor). Any viewing or disclosure of such information that is not authorized in writing by the sponsor is strictly prohibited. Such information may be used solely for the purpose of reviewing or performing this study.

2 CLINICAL PROTOCOL SYNOPSIS

Compound No.:

BAN2401

Name of Active Ingredient:

BAN2401

Study Protocol Title:

A Placebo-Controlled, Double-Blind, Parallel-Group, Bayesian Adaptive Randomization Design and Dose Regimen-Finding Study with an Open-Label Extension Phase to Evaluate Safety, Tolerability and Efficacy of BAN2401 in Subjects With Early Alzheimer's Disease

Investigator(s):

Unknown

Site(s):

Approximately 125 sites including North America, Europe, and Asia-Pacific (revised per Amendment 07)

Study Period and Phase of Development: (revised per Amendments 07 and 08)

Core Study: Up to 18 months of treatment plus 3 months of follow-up

Extension Phase: Up to 24 months of additional treatment plus 3 months of follow-up (revised per Amendment 11)

Phase 2

Objectives:

CORE STUDY

Primary Objectives:

1. To evaluate the efficacy of BAN2401 compared to placebo by establishing the ED_{90} (as defined in the protocol) for BAN2401 on the Alzheimer's Disease Composite Score (ADCOMS) at 12 months of treatment in subjects with Early Alzheimer's Disease (EAD), defined as mild cognitive impairment (MCI) due to Alzheimer's disease (AD) – intermediate likelihood or mild Alzheimer's disease dementia
2. To assess the safety and tolerability of 3 doses and 2 dose regimens of BAN2401 in subjects with EAD

Key Secondary Objectives: (revised per Amendments 09 and 10)

1. To evaluate the effects of BAN2401 compared to placebo on brain amyloid pathophysiology at 18 months of treatment in subjects with EAD as measured by amyloid positron emission tomography (PET) (revised per Amendments 09 and 10)
2. To evaluate the efficacy of BAN2401 compared to placebo on the ADCOMS at 18 months of treatment in subjects with EAD (revised per Amendments 09 and 10)
3. To evaluate the efficacy of BAN2401 compared to placebo on the Clinical Dementia Rating – Sum of Boxes (CDR-SB) at 18 months of treatment in subjects with EAD (revised per Amendment 10)
4. To evaluate the efficacy of BAN2401 compared to placebo on Alzheimer's Disease Assessment Scale - Cognitive Subscale (ADAS-Cog) in subjects with EAD at 18 months
5. To evaluate the effects of BAN2401 compared to placebo at 18 months on clinical status separately within subjects with MCI and mild AD dementia for the following assessments: ADCOMS, CDR-SB, and ADAS-Cog (revised per Amendment 10)
6. To evaluate the effects of BAN2401 compared to placebo on cerebrospinal fluid (CSF) biomarkers (Aβ[1-42], t-tau, and p-tau) at 18 months of treatment in subjects with EAD (revised per

Amendment 10)

7. To evaluate the effects of BAN2401 compared to placebo on total hippocampal volume using volumetric magnetic resonance imaging (vMRI) at 18 months of treatment in subjects with EAD (revised per Amendments 09 and 10)

Secondary Objectives:

1. To evaluate the effects of BAN2401 compared to placebo on brain amyloid pathophysiology at 12 months of treatment in subjects with EAD as measured by amyloid PET (revised per Amendments 09 and 10)
2. To evaluate the effects of BAN2401 compared to placebo at 12 months on clinical status in subjects with EAD for the following assessments: ADCOMS, CDR-SB, and ADAS-Cog (revised per Amendments 09 and 10)
3. To evaluate the effects of BAN2401 compared to placebo at 12 months on clinical status separately within subjects with MCI and mild AD dementia for the following assessments: ADCOMS, CDR-SB, and ADAS-Cog (revised per Amendment 10)
4. To evaluate the effects of BAN2401 compared to placebo on CSF biomarkers (Aβ[1-42], t-tau, and p-tau) at 12 months of treatment in subjects with EAD (revised per Amendment 10)
5. To evaluate the effects of BAN2401 compared to placebo on total hippocampal atrophy as measured by vMRI at 6 and 12 months, and left and right hippocampus, whole brain and total ventricular volume as measured by vMRI at 6, 12, and 18 months of treatment in subjects with EAD (revised per Amendment 10)

Exploratory Objectives:

1. To evaluate the effects of BAN2401 compared to placebo on clinical status in subjects with EAD by assessment of MMSE, and FAQ at time points not listed in Key Secondary and Secondary objectives (revised per Amendment 10)
2. To explore the relationship between change from baseline in clinical status at 12 and 18 months of treatment and baseline brain amyloid pathophysiology in subjects with EAD as measured by amyloid PET (revised per Amendment 10)
3. To explore the relationship between change from baseline in clinical status and change from baseline in brain amyloid pathopohysiology at 12 and 18 months of treatment in subjects with EAD as measured by amyloid PET (revised per Amendment 10)
4. To explore efficacy of BAN2401 compared to placebo and the overall study population on the ADCOMS across all clinical assessment time points in Japanese subjects with EAD (revised per Amendments 07 and 10)
5. To characterize the population pharmacokinetics (PK) of BAN2401 in EAD subjects, and to examine the effect of intrinsic and extrinsic factors on the PK (revised per Amendment 08)

EXTENSION PHASE (revised per Amendments 08 and 11)

Primary Objective:

To evaluate the long-term safety and tolerability of BAN2401 in subjects with EAD

Secondary Objectives:

To assess if the treatment benefit in brain amyloid levels (as measured by amyloid PET) at the end of the Core Study will be maintained over time in the Extension Phase in subjects with EAD (revised per Amendment 11)

Exploratory Objectives:

1. To evaluate the clinical effects of BAN2401 in subjects with EAD on the ADCOMS, CDR-SB, ADAS-Cog, and MMSE over time
2. To assess time to disease progression as evaluated by Clinical Dementia Rating (CDR) global score during the Extension Phase
3. To explore the long-term effects of BAN2401 in subjects with EAD on total hippocampal volume and other biomarkers (eg, plasma biomarkers) at 12 and approximately 24 months in the Extension Phase (Visit 70 [Extension Week 53], Visit 95 [Extension Week 103])
4. To characterize population PK of BAN2401 in subjects enrolled in the Extension Phase of the study

Study Design:

This will be a multinational, multicenter, double-blind, placebo-controlled, parallel-group study, using a Bayesian design with response adaptive randomization (RAR) across placebo or 5 active arms of BAN2401 to determine clinical efficacy and to explore the dose response of BAN2401 using a composite clinical score (ie, ADCOMS) and a 24-month Extension Phase. (revised per Amendment 11)

The Core Study is an 18-month study with 5 dose regimens, which comprise 3 dose levels given biweekly (once every 2 weeks) and 2 dose levels given monthly (once every 4 weeks). The dose levels in the Core Study are 2.5, 5, and 10 mg/kg biweekly and 5 and 10 mg/kg monthly. (revised per Amendment 11)

Frequent interim analyses will be conducted to continually update randomization allocation on the basis of the primary clinical endpoint. (revised per Amendment 01) This approach allows for ongoing assessment of drug futility or evidence for early success and for continued changes in randomization that favor efficacious treatment arms. Thus, the Bayesian approach not only limits exposure of subjects to non-efficacious treatment arms but can also mitigate the risks associated with larger and longer trials required to demonstrate clinical efficacy by leading to more efficient project termination or early advancement to a successful Phase 3 program. After the 12-month assessments have been completed, treatment will continue to 18 months to follow the time course of any treatment effects observed at 12 months, and to evaluate biomarker and neuroimaging effects that may be consistent with potential disease modification. Should the OLE Phase be implemented, treatment will continue for up to 60 months (5 years) of additional treatment to allow for the assessment of the long-term safety and tolerability of BAN2401. (revised per Amendment 08)Subjects will be from 2 clinical subgroups, collectively designated as EAD for the purposes of this protocol: (a) MCI due to AD – intermediate likelihood and (b) mild Alzheimer's disease dementia. At study entry, subjects will be stratified according to clinical subgroup, apolipoprotein E (*APOE*) status (ie, apolipoprotein ϵ4 variant [*APOE4*] positive and negative) and the presence or absence of ongoing AD treatment (ie, acetylcholinesterase inhibitors [AChEIs] or memantine or both). Subjects will be diagnosed as either MCI due to AD or mild Alzheimer's disease dementia as defined by the protocol after completion of MMSE, Wechsler Memory Scale IV-Logical Memory (subscale) I (WMS-IV LMI), Wechsler Memory Scale IV-Logical Memory (subscale) II (WMS-IV LMII), and CDR. Once the diagnosis is made, the WMS-IV LMII inclusion criterion can be applied to all subjects. (revised per Amendments 01 and 06) Randomization into the 2 clinical subgroups will be reasonably balanced whereby at least 60% of the total number of subjects will have MCI due to AD – intermediate likelihood and at least 30% will have mild Alzheimer's disease dementia. *APOE4* status (positive or negative) must be confirmed for all subjects prior to randomization. Subjects who are confirmed *APOE4* positive (*APOE4* hetero- or homozygous) will not be randomized to the 10 mg/kg, biweekly dose. (revised per Amendment 05) The first 196 subjects will be randomized according to a fixed schedule (4:2:2:2:2:2; Placebo [4] to each of the active arms [2 each]). After 196 subjects have been randomized into the study, an interim analysis (IA) will be conducted and the RAR will guide subsequent randomization into dose groups. Interim analyses and RAR will be repeated after 250 subjects have been randomized and again after each additional 50 subjects until all 800 subjects are randomized, and will then be repeated at 3-month intervals until all subjects complete 12 months of treatment. (revised per Amendment 07) A Bayesian analysis will be conducted at 12 months of treatment to assess for early success, and at 18 months of treatment, regardless of whether early success is achieved at 12 months. (revised per Amendment 09)

<u>Drug Safety Monitoring Board</u>

A separate unblinded Drug Safety Monitoring Board (DSMB) will be employed at regular intervals to monitor the

overall safety of the study and will make recommendations to the sponsor as appropriate. In addition, the DSMB will meet when a predetermined number of subjects are randomized to assess whether the option for home infusions is feasible (per DSMB charter, if allowed and conducted according to country and local guidelines; home infusions will not be allowed in Germany. Upon implementation of Amendment 07, newly enrolled subjects will not be offered the option for home infusions. Subjects opting for home infusions before implementation of Amendment 07 will be allowed to continue with home infusions for the duration of their participation in the study. (revised per Amendments 01, 03, and 07) Details regarding DSMB membership and operational characteristics will be provided in the DSMB Charter. The DSMB will be responsible for monitoring the safety of the study only in the Core Study. (revised per Amendment 11)

Core Study (revised per Amendment 11)

The Core study will consist of a Prerandomization Phase and a Randomization Phase. (revised per Amendment 08)

The Prerandomization Phase (revised per Amendment 08) may last up to 60 days, and will consist of a Screening Period (up to 30 days duration) and a Baseline Period (up to 30 days duration). During the Screening Period (Day -60 to –31; Visit 1) and Baseline Period (Day -30 to -1; Visit 2), all subjects will be assessed for eligibility using clinical tests, safety MRIs, and amyloid PET assessments to confirm that subjects meet the diagnostic criteria for MCI due to AD – intermediate likelihood or mild Alzheimer's disease dementia and that they do not have other medical conditions that may interfere with study participation.

Clinical assessments for eligibility will be conducted in the morning (whenever possible) at the Screening Visit in the following order: MMSE, Wechsler Memory Scale –IV Logical Memory I and II (WMS-IV LMI and WMS-IV LMII), and CDR. The WMS-IV LMII is to be administered 20 to 30 minutes after the completion of LMI. Subject information should be collected in the 20 to 30 minutes between the WMS-IV LM1 and LMII. Subjects will also be evaluated on the Geriatric Depression Scale (GDS) for eligibility. (revised per Amendment 01) Subjects who pass this initial screen for eligibility will be assessed by safety magnetic resonance imaging (MRI) for brain abnormalities that may affect eligibility, and all subjects will undergo an additional scanning sequence immediately following the safety MRI for vMRI during the Screening visit. The safety and vMRI assessments conducted during Screening will also be used as the baseline MRI assessments for these respective measures in subjects who are eligible.

Further assessments will be conducted during the Baseline Period, including additional clinical evaluations, mandatory blood sampling for pharmacogenomics (*APOE4* status) per Appendix 3, and CSF sampling for those who consent to soluble CSF biomarker analysis. (revised per Amendment 01) At the Baseline Visit, clinical assessments are to be conducted in the morning (whenever possible) in the following order: MMSE, CDR, ADAS-Cog, and FAQ. For further assessment of eligibility, within 20 days after the Baseline Visit all qualified subjects will undergo amyloid PET to confirm the presence of amyloid in the brain. Baseline amyloid PET data will serve as the baseline data for subjects in the imaging subgroup (target n=306 total) who will receive amyloid PET at 12 and 18 months of treatment (revised per Amendments 02 and 07). *APOE4* status (positive or negative) must be confirmed for all subjects prior to randomization. Subjects who are confirmed *APOE4* positive (*APOE4* hetero- or homozygous) will not be randomized to the 10 mg/kg, biweekly dose. (revised per Amendment 05)

The Randomization Phase (revised per Amendment 08) will consist of an 18-month Treatment Period and a 3-month Follow-Up Period. Subjects will be randomized at Visit 3 (Day 1) to receive placebo or 1 of 5 treatment arms of BAN2401, administered as an approximate 60-minute intravenous (IV) infusion every 2 weeks. All subjects will undertake assessments of cognitive function, safety, pharmacokinetic (PK), safety MRI, and vMRI while select subjects will undertake additional CSF biomarkers or amyloid PET or both per the Schedule of Assessments. (revised per Amendment 01) At designated study visits, clinical assessments will be conducted in the morning (whenever possible) in the following order: MMSE, CDR, ADAS-Cog, and FAQ. The Follow-up Visit will take place 3 months after the last treatment.

A Visit 6 abbreviated MRI (safety MRI to detect amyloid related imaging abnormalities (ARIA) lasting 8 to 10 minutes, with no volumetric MRI [vMRI] sequences) will be conducted at European sites only. (revised per Amendment 07) An early safety MRI will also be conducted at Visit 7. (revised per Amendment 04) Clinical assessment of efficacy endpoints and safety MRIs will be conducted every 3 months until the End of Treatment or until Discontinuation from the study. (revised per Amendment 01) Volumetric MRI sequences will be taken from

all subjects immediately following all safety MRI assessments. Analysis of vMRI assessments will be conducted at the Screening Visit and at Visits 16, 29, and 42. In some cases, unscheduled visits will be needed to follow up on regularly scheduled safety or safety MRI findings, and the related assessments (outlined in the Schedule of Assessments) will depend on the nature of the unscheduled visit as determined by the investigator. Amyloid PET will be collected for subjects who consent to undergo this scan at 12 and 18 months of treatment. Physical examinations, safety assessments, assessments for antidrug antibodies, standard laboratory assessments, blood for PK and exploratory biomarker assessments, and CSF for soluble CSF biomarker assessments will be collected as specified in Appendix 3.

Subjects who have completed Visit 42 (Week 79) of the Core Study (and do not participate in the Extension Phase) will undergo the 3-month Follow-Up Visit after their last dose of study drug in the Core Study per the Schedule of Assessments. (revised per Amendment 08) Subjects who discontinue the Core Study or study drug early must comply with the Early Termination Visit (within 7 days after the last dose of study drug) and the Follow-up Visit (3 months after the last dose of study drug). (revised per Amendment 08) In addition, subjects who discontinue study drug in the Core Study are required to return after the Early Termination Visit for each scheduled visit when clinical efficacy assessments are to be conducted (Visits 9, 16, 22, 29, 35, and 42) per the Schedule of Procedures/Assessments for the Core Study. (revised per Amendments 06 and 08) At these visits, clinical efficacy assessments (MMSE, ADAS-Cog, CDR, and FAQ) will be conducted and information on concomitant medications, adverse events (AEs), and serious adverse events (SAEs) will be collected. Subjects who discontinue from study drug are considered on study as long as they return for their regularly scheduled clinical efficacy visits as outlined above. (revised per Amendment 01)

EXTENSION PHASE (REVISED PER AMENDMENTS 08 AND 11)

The Extension Phase will be initiated following the Core Study to allow subjects to receive open-label BAN2401 10 mg/kg biweekly for up to 24 months (2 years), until the drug is commercially available in the country where the subject resides, or until the benefit-to-risk ratio from treatment with BAN2401 is no longer considered favorable, whichever comes first. Any subject who completed study treatment (Visit 42 [Week 79] of the Core Study) and fulfills the Extension Phase inclusion and exclusion criteria will have the option to participate. Subjects who previously completed the Core Study (through the Follow-Up Visit, Visit 43) at any time before implementation of the Extension Phase, and/or fulfill the Extension Phase inclusion and exclusion criteria, will also be eligible to participate. Subjects who discontinued the Core Study will be eligible to participate in the Extension Phase, provided they meet the inclusion and exclusion criteria for the Extension Phase. (revised per Amendment 12)

All subjects will receive the BAN2401 10 mg/kg biweekly dose, including subjects who are confirmed *APOE4* positive (hetero- or homozygous). The Follow-up Visit in the Extension Phase will take place 3 months after the last dose of study drug. Subjects may discontinue from study drug for any reason. Subjects who discontinue the study drug must comply with the Early Termination Visit (within 7 days after the last dose of study drug) and the Follow Up Visit (3 months after the last dose of study drug).

Safety assessments will be performed and all AEs and SAEs will be recorded. Vital signs will be assessed when study drug is administered both at predose and after infusion. Hematology, blood chemistry assessments, and urinalysis will be performed at Extension Baseline (Extension Screening Visit), at Extension Phase Weeks 3, 7, 13, 19, 27, and every 6 months thereafter. Safety and vMRIs will be performed at baseline (Extension Screening Visit), at Extension Phase Weeks 9, 13, 27, and every 6 months thereafter. Clinical assessments will be administered at baseline (Extension Screening Visit) and every 6 months in the morning (whenever possible) in the following order: MMSE, CDR, and the ADAS-Cog. Blood for serum PK and anti-BAN2401 antibodies will be collected at Extension Phase Week 1 (Visit 44); Extension Weeks 3, 9, 13, 27, 39, 53, 79, 103 (last dosing visit), 105, and 117.

All subjects who underwent amyloid PET for inclusion in the Core Study should receive a baseline amyloid PET scan before dosing in the Extension Phase. The baseline amyloid PET scan must be conducted with the same imaging tracer that was used for inclusion at the baseline visit for the Core Study. In addition, qualified subjects located in the US and Japan will have the option to participate in the longitudinal PET substudy. (revised per Amendment 12) In this imaging substudy, florbetapir will be used in the US and flutemetamol will be used in Japan, and only subjects who consent to the longitudinal assessments per the Extension Phase Schedule of Assessments will be allowed to participate in this substudy. At the Extension Screening Visit, subjects who

consented to the longitudinal imaging substudy will be stratified into 2 cohorts based on their treatment allocation during the Core Study. Cohort 1 will have amyloid PET assessments performed at baseline (Extension Screening Visit), Visit 50 (Extension Week 13), Extension Phase Visit 70 [Extension Week 53], and Visit 96 [Extension Week 105]; Cohort 2 will have amyloid PET assessments performed at baseline (Extension Screening Visit), Visit 57 (Extension Week 27), at Extension Phase Visit 70 [Extension Week 53], and Visit 96 [Extension Week 105]. In Japan, those who consented to the longitudinal imaging substudy will only undergo amyloid PET at Extension Phase Visit 70 [Extension Week 53], and Visit 96 [Extension Week 105]. (Revised per Amendment 12)

During the Extension Phase, subjects who develop asymptomatic Amyloid Related Imaging Abnormality-hemorrhage (ARIA-H), will continue on the study uninterrupted per the Schedule of Assessments and do not require additional MRI follow-up outside the regularly scheduled assessments. Subjects who develop multiple (>10) asymptomatic cerebral microhemorrhages, superficial siderosis, or a single macrohemorrhage (greater than 10 mm at greatest diameter), will continue on the study uninterrupted per the Schedule of Assessments, and will undertake an unscheduled safety visit (with MRI) at approximately 30 days after the MRI features (asymptomatic ARIA-H) were first identified. Thereafter, all subjects who develop these events will have further safety visits (with MRI) at approximately every 30 days (which may be an unscheduled or a scheduled visit) until the asymptomatic ARIA-H has stabilized radiographicially. Subjects who develop symptomatic ARIA-H (including symptomatic microhemmorhages, symptomatic superficial siderosis, symptomatic macrohemorrhage) on MRI will have study drug administration temporarily stopped. All subjects who develop these events will undertake an unscheduled safety visit (with MRI) at approximately 30 days after the MRI features (ARIA-H) were first identified. Thereafter they will have further safety visits (with MRI) at approximately every 30 days (which may be unscheduled or scheduled visit) until the ARIA-H has stabilized and is no longer symptomatic. During treatment interruption, time will elapse on the Schedule of Assessments when the scheduled visit would otherwise occur. They may then resume treatment for the study duration and study assessments remaining treatment on the Schedule of Assessments. Resumption of treatment following symptomatic ARIA-H can only occur twice, after which the subject must be discontinued from the study. (revised per Amendment 12)

Subjects who develop asymptomatic, radiographically mild or moderate Amyloid Related Imaging Abnormality-Edema/Effusion (ARIA-E) on MRI will continue on the study uninterrupted per the Schedule of Assessments. They will undertake an unscheduled safety visit (with MRI) at approximately 30 days and another safety visit (with MRI) at approximately 90 days (which may be unscheduled or scheduled visit) after the MRI features were first identified. They will continue with study drug treatment if their ARIA-E does not become severe radiologically and remains asymptomatic.

Those subjects who develop symptomatic or radiographically severe treatment emergent ARIA-E will be temporarily stopped from study drug administration until the ARIA-E resolves radiographically, and will be followed up to resolution of the event (including symptoms, if any). They will undertake an unscheduled safety visit (with MRI) at approximately 30 days and another safety visit (with MRI) at approximately 90 days (which may be unscheduled or scheduled visit) after the symptomatic or severe ARIA-E was first identified. Thereafter, these subjects will have a safety visit (with MRI) at approximately 30 days (which may be an unscheduled or a scheduled visit) until the ARIA-E has resolved both radiologically and clinically. During treatment interruption, time will elapse on the Schedule of Assessments when the scheduled visit would otherwise occur. Once the ARIA-E has resolved both radiologically and clinically, they may then resume treatment for the study duration and study assessments on the Schedule of Assessments. Resumption of treatment following symptomatic ARIA-E can only occur twice, after which the subject must be discontinued from the study. (revised per Amendment 12)

Radiographic severity is defined as follows: (revised per Amendent 12)

No ARIA-E present: No signs of ARIA-E.

Questionable ARIA-E: Subtle sulcal or cortical fluid-attenuated inversion recovery (FLAIR) hyperintensity, most likely artifactual.

Mild ARIA-E: Mild FLAIR hyperintensity confined to sulcus and/or cortex/subcortex white matter (with or without gyral swelling and sulcal effacement), which affects an area of less than 5 cm in a single greatest dimension. Only a single region of involvement detected.

Moderate ARIA-E: Moderate involvement (area of FLAIR hyperintensity measuring 5-10 cm in single greatest dimensions), or more than one site of involvement, each measuring less than 10 cm in a single greatest

dimension.

Severe ARIA-E: Severe involvement (area of FLAIR hyperintensity measuring greater than 10 cm in single greatest dimension [white matter and/or sulcal involvement with associated gyral swelling and sulcal effacement]). One or more separate/independent sites of involvement may be noted.

Should a subject discontinue from study treatment from one of these events, such subjects will undergo the Early Termination Visit within 7 days of discontinuation and will undergo the 3 month Follow Up Visit per protocol. These subjects will continue to be followed with safety MRIs on a monthly basis thereafter, until the finding has either resolved or stabilized.

An end-of-study assessment will be performed for subjects who complete treatment in the Extension Phase (Visit 96, Extension Week 105). Subjects may discontinue from study drug for any reason in the Extension Phase. Subjects who discontinue study drug early must comply with the Early Termination Visit (within 7 days after the last dose of study drug) during which end-of-study assessments will be performed. Subjects who discontinue study drug early may also have unscheduled visits for safety assessments as needed. A Follow-up Visit will take place 3 months after the last dose of study drug for all subjects.

Number of Subjects:

Approximately 3200 subjects will be screened to provide a maximum of 800 randomized subjects. Sites in Japan will screen approximately 160 subjects in order to randomize at least 40 Japanese subjects, even if early success is declared or the study achieves 800 subjects randomized. (revised per Amendment 07)

Inclusion Criteria:

CORE STUDY

Diagnosis

Mild Cognitive Impairment due to Alzheimer's Disease – intermediate likelihood: (revised per Amendment 06)

1. Subjects who meet the National Institute of Aging – Alzheimer's Association (NIA-AA) core clinical criteria for mild cognitive impairment due to Alzheimer's disease – intermediate likelihood
2. Subjects who have a CDR score of 0.5 and a Memory Box score of 0.5 or greater at Screening and Baseline
3. Subjects who report a history of subjective memory decline with gradual onset and slow progression over the last 1 year before Screening; MUST be corroborated by an informant

Mild Alzheimer's Disease Dementia:

4. Subjects who meet the NIA-AA core clinical criteria for probable Alzheimer's disease dementia (revised per Amendment 01)
5. Subjects who have a CDR score of 0.5 to 1.0 and a Memory Box score of 0.5 or greater at Screening and Baseline

Key Inclusion Criteria **that must be met by ALL Subjects:**

6. Subjects with objective impairment in episodic memory as indicated by at least 1 standard deviation below age-adjusted mean in the WMS-IV LMII, as follows: (revised per Amendments 01 and 06)
   a) ≤15 for age 50 to 64 years
   b) ≤12 for age 65 to 69 years
   c) ≤11 for age 70 to 74 years
   d) ≤9 for age 75 to 79 years
   e) ≤7 for age 80 to 90 years

7. Positive amyloid load as indicated by 1 of the following:
    a. PET assessment of imaging agent uptake into brain
    b. CSF assessment of Aβ(1-42) (revised per Amendment 06)

    Subjects may consent to both the PET and CSF assessments, but need a positive amyloid result in only one of the 2 procedures to confirm eligibility (ie, even if 1 of the 2 results does not meet its respective eligibility criterion). Subjects who initially consent for only one of the amyloid screening assessments will only be allowed to subsequently consent for the second assessment should the first assessment result be positive or they have not yet been informed of the results of the first assessment. Subjects who consent to Amyloid PET or CSF Amyloid are not required to participate in the respective substudies. (revised per Amendments 06 and 07)

    Any historical amyloid positive PET scan may be used for eligibility upon evaluation by the central imaging contract research organization (CRO). For subjects who wish to participate in the longitudinal imaging substudy, historical amyloid PET scans may not be used as baseline scans for the longitudinal imaging substudy. Required details regarding historical images should be submitted to the CRO medical monitor for evaluation. (revised per Amendment 08)
8. Male or female subjects aged between 50 and 90 years, inclusive
9. MMSE score equal to or greater than 22, and equal to or less than 30 at Screening and Baseline, except for the following countries, where MMSE score must be equal to or greater than 22 and equal to or less than 28 at Screening and Baseline: United Kingdom, Spain, Germany, Sweden, France, and the Netherlands (revised per Amendment 03)
10. Body Mass Index (BMI) >17 and <35 at Screening (revised per Amendment 06)
11. Females must not be lactating or pregnant at Screening or Baseline (as documented by a negative human β-chorionic gonadotropin assay [ß-hCG]. A separate baseline assessment is required if a negative screening pregnancy test was obtained more than 72 hours before the first dose of study drug.
12. All females will be considered to be of childbearing potential unless they are postmenopausal (amenorrheic for at least 12 consecutive months, in the appropriate age group, and without other known or suspected cause) or have been sterilized surgically (ie, bilateral tubal ligation, total hysterectomy, or bilateral oophorectomy, all with surgery at least 1 month before dosing).
13. Females of childbearing potential must not have had unprotected sexual intercourse within 30 days before study entry and must agree to use a highly effective method of contraception (eg, total abstinence, an intrauterine device, a double-barrier method [such as condom plus diaphragm with spermicide], a contraceptive implant, an oral contraceptive, or have a vasectomized partner with confirmed azoospermia) throughout the entire study period and for 35 days after study drug discontinuation. If currently abstinent, the subject must agree to use a double-barrier method as described above if she becomes sexually active during the study period or for 35 days after study drug discontinuation. Females who are using hormonal contraceptives must have been on a stable dose of the same hormonal contraceptive product for at least 4 weeks before dosing and must continue to use the same contraceptive during the study and for 35 days after study drug discontinuation.
14. Subjects who are receiving an AChEIs or memantine or both for AD must be on a stable dose for at least 12 weeks prior to Baseline. Treatment-naïve subjects for AD can be entered into the study. Unless otherwise stated, subjects must have been on stable doses of all other (ie, non-AD related) permitted concomitant medications for at least 4 weeks prior to Baseline. Use of memantine will not be allowed for Japanese subjects (revised per Amendments 01 and 07)
15. Must have an identified caregiver/informant (defined as a person able to support the subject for the duration of the study and who spends at least 8 hours per week with the subject). The caregiver/informant must provide separate written informed consent. In addition, this person must be willing and able to provide follow-up information on the subject throughout the course of the study. This person must, in the opinion of the investigator, spend sufficient time with the subject on a regular basis such that the caregiver/informant can reliably fulfill the study requirements. A permanent caregiver/informant need not be living in the same residence with the subject. For such a caregiver/informant not residing with the

subject, the investigator has to be satisfied that the subject can contact the caregiver/informant readily during the times when the caregiver/informant is not with the subject. If in doubt about whether a subject's care arrangements are suitable for inclusion, the investigator should discuss this with the medical monitor. Caregivers/informants need only to be present at visits where clinical assessment of CDR and FAQ takes place. (revised per Amendment 01)

16. Provide written informed consent. If a subject lacks capacity to consent in the investigator's opinion, the subject's assent should be obtained, if required in accordance with local laws, regulations and customs, plus the written informed consent of a legal representative should be obtained (capacity to consent and definition of legal representative should be determined in accordance with applicable local laws and regulations). (revised per Amendment 07)
17. Willing and able to comply with all aspects of the protocol

EXTENSION PHASE (revised per Amendments 08, 11, and 12)

1. Have completed Visit 42 (Week 79) of the Core Study or who discontinued study drug during the Core Study due to any of the following reasons:
    a. ARIA-E
    b. ARIA-H (superficial siderosis, macrohemorrhage, or symptomatic microhemorrhage)
    c. Prohibited or restricted medications that were prohibited during Core Study conduct but are no longer prohibited in the Extension Phase
    d. Subjects who were APOE4 positive and receiving treatment with BAN2401 10 mg/kg biweekly
    e. Any reason for discontinuation not related to prohibited medications, including any AE that was considered not related to study drug, and that was not severe or life-threatening
2. Must continue to have an identified caregiver or informant who is willing and able to provide follow-up information on the subject throughout the course of the Extension Phase
3. Provide written informed consent. If a subject lacks capacity to consent in the investigator's opinion, the subject's assent should be obtained, if required in accordance with local laws, regulations and customs, plus the written informed consent of a legal representative should be obtained (capacity to consent and definition of legal representative should be determined in accordance with applicable local laws and regulations).
4. Must be able to physically attend clinic visits and be willing and able to comply with all aspects of the protocol

Exclusion Criteria:

CORE STUDY

Key Exclusion Criteria:

1. Any neurological condition that may be contributing to cognitive impairment above and beyond that caused by the subject's AD
2. History of transient ischemic attacks (TIA), stroke, or seizures within 12 months of Screening
3. Any psychiatric diagnosis or symptoms, (eg, hallucinations, major depression, or delusions) that could interfere with study procedures in the subject
4. GDS score ≥8 at Screening
5. Contraindications to MRI scanning, including cardiac pacemaker/defibrillator, ferromagnetic metal implants (eg, in skull and cardiac devices other than those approved as safe for use in MR scanners)
6. Evidence of other clinically significant lesions that could indicate a dementia diagnosis other than AD on brain MRI at Screening. All MRIs will be acquired using a standardized procedure that will be outlined in the Imaging Charter and Imaging Acquisition Guidelines (IAG) and will be read by an approved centralized reader.
7. Other significant pathological findings on brain MRI at Screening, including but not limited to: more than 4 microhemorrhages (defined as 10 mm or less at the greatest diameter); a single macrohemorrhage greater than 10 mm at greatest diameter; an area of superficial siderosis; evidence of vasogenic edema; evidence of cerebral contusion, encephalomalacia, aneurysms, vascular malformations, or infective lesions; evidence of multiple lacunar infarcts or stroke involving a major vascular territory, severe small vessel, or white matter disease; space occupying lesions; or brain tumors [(however, lesions diagnosed as meningiomas or arachnoid cysts and <1cm at their greatest diameter need not be exclusionary] (revised per Amendment 07)
8. Hypersensitivity to BAN2401 or any of the excipients, or to any monoclonal antibody treatment
9. Any immunological disease which is not adequately controlled, or which requires treatment with biologic drugs during the study
10. Subjects with a bleeding disorder that is not under adequate control (including a platelet count <50,000 or interational normalized ratio [INR] >1.5)
11. Subjects who have thyroid stimulating hormone (TSH) above normal range. Other tests of thyroid function with results outside the normal range should only be exclusionary if they are considered clinically significant by the investigator. This applies to all subjects whether or not they are taking thyroid supplements. (revised per Amendments 01 and 06).
12. Abnormally low serum Vitamin B12 levels for the testing laboratory (if subject is taking Vitamin B12 injections, level should be at or above the lower limit of normal [LLN] for the testing laboratory).
13. A prolonged QT/QTc interval (QTc >450 ms) as demonstrated by a repeated electrocardiogram (ECG)
14. Known to be human immunodeficiency virus (HIV) positive
15. Any other clinically significant abnormalities in physical examination, vital signs, laboratory tests or ECG at Screening or Baseline which in the opinion of the principal investigator (PI), require further investigation or treatment or which may interfere with study procedures or safety
16. Uncontrolled Type 1 or Type 2 diabetes mellitus. Evidence of uncontrolled diabetes mellitus includes hemoglobulin A1c (HbA1c) >9%.
17. Uncontrolled hypertension with a history of blood pressure consistently above 165/100 mm Hg at Screening
18. History of uncontrolled cardiovascular disease within 6 months of Screening, including acute coronary syndrome, clinically significant valvular heart disease, uncompensated heart failure (New York Heart Association [NYHA] Class III and Class IV), or uncontrolled arrhythmia
19. Subjects with malignant neoplasms within 3 years of Screening (except for basal or squamous cell carcinoma in situ of the skin, or localized prostate cancer in male subjects). Subjects who had malignant neoplasms but who have had at least 3 years of documented uninterrupted remission before Screening need not be excluded.
20. Has a "yes" answer to Columbia Suicide Severity Rating Scale (C-SSRS) suicidal ideation Type 4 or 5, or any suicidal behavior assessment within 6 months before Screening, at Screening, or at the Baseline Visit, or has been hospitalized or treated for suicidal behavior in the past 5 years before Screening

21. Known or suspected history of drug or alcohol abuse or dependence within 2 years before Screening or a positive urine drug test at Screening. Subjects who test positive for benzodiazepines or opioids in urine drug testing need not be excluded if in the clinical opinion of the investigator, this is due to the subject taking prior/concomitant medications containing benzodiazepines or opioids for a medical condition and not due to drug abuse.
22. Any other medical conditions (eg, cardiac, respiratory, gastrointestinal, renal disease) which are not stably controlled, or which in the opinion of the investigator(s) could affect the subject's safety or interfere with the study assessments
23. Subjects who are taking prohibited medications
24. Participation in a clinical study involving any therapeutic monoclonal antibody, protein derived from a monoclonal antibody, immunoglobulin therapy, or vaccine within 6 months before Screening (revised per Amendment 01)
25. Participation in a clinical study involving any new chemical entities for AD within 6 months prior to Screening unless it can be documented that the subject was in a placebo treatment arm
26. Participation in any other investigational medication or device study in the 8 weeks or 5 half-lives (whichever is longer) of the medication before randomization unless it can be documented that the subject was in a placebo treatment arm
27. Planned surgery which requires general, spinal or epidural anesthesia that would take place during the study. Planned surgery which requires only local anesthesia and which can be undertaken as day case without inpatient stay postoperatively need not result in exclusion if in the opinion of the principal investigator this operation does not interfere with study procedures and subject safety.
28. Severe visual or hearing impairment that would prevent the subject from performing psychometric tests accurately

EXTENSION PHASE (revised per Amendments 08 and 11)

29. Subjects who discontinued from the study drug or from the Core Study for reasons other than the following:
    a. ARIA-E
    b. ARIA-H (superficial siderosis, macrohemorrhage, or symptomatic microhemorrhage)
    c. Prohibited or restricted medications that were prohibited during Core Study conduct but are no longer prohibited in the extension phase (revised per Amendment 12)
    d. Subjects who were APOE4 positive and receiving treatment with BAN2401 10 mg/kg biweekly
    e. AE that was considered not related to study drug, and that was not severe or life-threatening
30. Females who are breastfeeding or pregnant at Extension Phase Baseline (Extension Screening Visit, as documented by a negative β-hCG assay). A separate baseline assessment is required if a negative screening pregnancy test was obtained more than 72 hours before the first dose of study drug.
31. Females of childbearing potential who:
32. Had unprotected sexual intercourse within 30 days before Extension Phase Baseline and who do not agree to use a highly effective method of contraception (eg, total abstinence, an intrauterine device, a double-barrier method [such as condom plus diaphragm with spermicide], a contraceptive implant, an oral contraceptive, or have a vasectomized partner with confirmed azoospermia) throughout the entire study period and for 28 days after study drug discontinuation.
33. Are currently abstinent, and do not agree to use a double-barrier method (as described above) or refrain from becoming sexually active during the study period or for 28 days after study drug discontinuation.
34. Are who are using hormonal contraceptives but are not on a stable dose of the same hormonal contraceptive product for at least 4 weeks before dosing and who do not agree to use the same contraceptive during the study and for 28 days after study drug discontinuation.

    (NOTE: All females will be considered to be of childbearing potential unless they are postmenopausal [amenorrheic for at least 12 consecutive months, in the appropriate age group, and without other known or suspected cause] or have been sterilized surgically [ie, bilateral tubal ligation, total hysterectomy, or bilateral oophorectomy, all with surgery at least 1 month before dosing]).
35. Subjects who develop the following conditions from the time of completion of the Core Study to the start of the Extension Phase:

a. Contraindications to MRI scanning, including cardiac pacemaker/defibrillator, ferromagnetic metal implants (eg, in skull and cardiac devices other than those approved as safe for use in MRI scanners)
b. Other significant pathological findings on brain MRI at Extension Phase Baseline (Extension Screening Visit), including but not limited to: any macro-hemorrhage (greater than 10 mm at greatest diameter), which is currently symptomatic or worsened since the Core Study; any area of superficial siderosis which is currently symptomatic or worsened since the Core Study; evidence of vasogenic edema, which is severe or symptomatic; aneurysms, vascular malformations, infective lesions, evidence of multiple lacunar infarcts or stroke involving a major vascular territory, severe small vessel, or white matter disease or space occupying lesions or brain tumors (however lesions diagnosed as meningiomas or arachnoid cysts and <1cm at their greatest diameter need not be exclusionary)
c. Any immunological disease which is not adequately controlled, or which requires treatment with biologic drugs during the study
d. Bleeding disorder that is not under adequate control (including a platelet count <50,000 or INR >1.5)
e. Receiving treatment with anticoagulant therapy but anticoagulation control is not optimized and is stable for at least 4 weeks before Extension Phase Baseline (Extension Screening Visit)
f. Any other clinically significant abnormalities in physical examination, vital signs, laboratory tests or ECG at Extension Phase Baseline (Extension Screening Visit), which in the opinion of the PI, require further investigation or treatment or which may interfere with study procedures or safety
g. Malignant neoplasms within 3 years of Extension Phase Baseline (Extension Screening Visit) (except for basal or squamous cell carcinoma in situ of the skin, or localized prostate cancer in male subjects). Subjects who had malignant neoplasms but who have had at least 3 years of documented uninterrupted remission before Extension Phase Baseline (Extension Screening Visit) need not be excluded
h. Known or suspected history of drug or alcohol abuse or dependence within 2 years before Extension Phase Baseline (Extension Screening Visit) or a positive urine drug test at Extension Phase Baseline (Extension Screening Visit). Subjects who test positive for benzodiazepines or opioids in urine drug testing need not be excluded if in the clinical opinion of the investigator, this is due to the subject taking prior/concomitant medications containing benzodiazepines or opioids for a medical condition and not due to drug abuse
i. Any other medical conditions (eg, cardiac, respiratory, gastrointestinal, renal disease) which are not stably controlled, or which in the opinion of the investigator(s) could affect the subject's safety or interfere with the study assessments
j. Subjects who are taking restricted or prohibited medications (revised per Amendment 12)
k. Participation in a clinical study involving any therapeutic monoclonal antibody, protein derived from a monoclonal antibody, immunoglobulin therapy, or vaccine during the time between the Core Study and Extension Phase Baseline (Extension Screening Visit)
l. Participation in a clinical study involving any new chemical entities for AD during the time between the Core Study and Extension Phase Baseline (Extension Screening Visit)
m. Participation in any other investigational medication or device study during the time between the Core Study and Extension Phase Baseline (Extension Screening Visit)
n. Planned surgery which requires general, spinal or epidural anesthesia that would take place during the study. Planned surgery which requires only local anesthesia and which can be undertaken as day case without inpatient stay postoperatively need not result in exclusion if in the opinion of the principal investigator this operation does not interfere with study procedures and subject safety

36. Severe visual or hearing impairment that would prevent the subject from performing psychometric tests accurately

Study Treatment(s) and Response Adaptive Randomization (RAR):

CORE STUDY

BAN2401 drug product is supplied as a sterile aqueous solution containing 10 mg/ml BAN2401, 25 mM sodium citrate/citric acid, 125 mM sodium chloride and 0.02% (w/v) polysorbate 80 at pH 5.7 in a 10-ml glass vial. BAN2401 will be administered in normal saline as 60 minute IV infusions. An infusion system containing a terminal 0.22-μm in-line filter is required for administration of BAN2401 drug product. (revised per Amendment 08)

BAN2401 will be administered on a mg/kg basis at doses of 2.5, 5, or 10 mg/kg biweekly (once every 2 weeks), or 5 or 10 mg/kg monthly (once every 4 weeks). All subjects will receive biweekly infusions, and subjects who have monthly dosing of BAN2401 will have placebo infusion alternating with BAN2401.

Response Adaptive Randomization

APOE4 status (positive or negative) must be confirmed for all subjects prior to randomization. (revised per Amendment 05) Subjects who are confirmed *APOE4* positive (hetero- or homozygous) will not be randomized to the 10 mg/kg, biweekly dose. (revised per Amendments 04 and 05). Randomization to placebo or 1 of 5 treatment arms of BAN2401 will be fixed for the first 196 subjects randomized in the study (4:2:2:2:2:2; placebo [4] to each of the active arms [2 each]). Randomization probabilities to each arm will be updated at each IA such that the randomization probability will be increased for the placebo arm and arms that represent the potential target dose (ED_{90}), and simultaneously decreased for other active arms.

EXTENSION PHASE (revised per Amendments 08 and 11)

BAN2401 drug product is currently supplied as a sterile aqueous solution containing 10 mg/mL BAN2401, 25 mM sodium citrate/citric acid, 125 mM sodium chloride and 0.02% (w/v) polysorbate 80 at pH 5.7 in a 10-mL glass vial. This current formulation of the drug product may be progressively phased out as stocks near the end of their shelf life and replaced by a newer formulation containing 100mg/mL BAN2401. The newer formulation (100 mg/mL BAN2401) was developed to reduce the number of vials of drug product required per dose and to reduce demand for storage space in a pharmacy. The newer (100 mg/mL BAN2401) formulation is supplied as a sterile aqueous solution containing 100 mg/mL BAN2401, 50 mmol/L citric acid, 0.05% (w/v) polysorbate 80, 350 mmol/L arginine, at pH 5.0, in glass vials containing 5 mL solution. The phasing out of the older formulation will only occur after appropriate regulatory authority approval for the newer formulation has been obtained, and adequate training of any pharmacy staff involved in the preparation of the final drug product for infusion has been completed. The appearance of the 2 different formulations after dilution in normal saline is identical.

All subjects, including those who are confirmed *APOE4* positive (hetero- or homozygous), will be administered the BAN2401 10 mg/kg biweekly dose. BAN2401 will be administered in normal saline as 60 minute IV infusions. An infusion system containing a terminal 0.22-μm in-line filter is required for administration of BAN2401 drug product.

Duration of Treatment (revised per Amendments 08 and 11)

Core Study: up to 18 months of treatment.

Extension Phase: up to 24 months (2 years) of additional treatment.

Concomitant Drug/Therapy:

Core Study:

The following therapies are not permitted for the listed intervals prior to Baseline and until the Follow-Up Visit (Visit 43, for subjects who participate in the Core Study only (revised per Amendments 08, 09, and 11)

- Immunoglobulin therapy, 6 months
- Biologic drugs, 6 months
- Anticoagulants (eg, warfarin, dabigatran), 7 days or 5 half-lives, whichever is longer

Short-term treatment (<4 weeks) with anticoagulants is permitted for randomized subjects who undergo procedures requiring anticoagulants for prophylaxis of thromboembolic disease after approval by the sponsor's medical monitor. While these subjects need not be discontinued, study drug will be temporarily suspended for these subjects during anticoagulant therapy. (revised per Amendment 06)

The following requirements apply to use of AChEIs or memantine or both for the treatment of AD: (revised per Amendment 01)

- Subjects should be on a stable dose for 12 weeks before Baseline, and subjects are not to change their dose of AD medications before randomization. (revised per Amendment 08)
- Subjects should remain on a stable dose while on study
- Memantine is not permitted for Japanese subjects (revised per Amendment 07)
- Subjects who initiate AD treatment or who adjust their AD dosing regimen while on study will continue to study completion. However, the clinical efficacy data (ie, derived Clinical Composite Score, MMSE, CDR-SB, ADAS-Cog, and FAQ) from these subjects will be censored at the time of AD treatment initiation or adjustment for the purposes of subsequent interim analyses and for the final analysis.

The following requirements apply to all other medications not intended to treat AD:

- Subjects must be on stable dose for at least 4 weeks prior to Baseline, except for medications which are administered as short courses of treatment (eg, anti-infectives) or which are to be used as needed (PRN).
- Medications which are used on a PRN basis or as a short course of treatment, and which are central nervous system (CNS) active and may affect cognitive function are not permitted during a period of 72 hours prior to cognitive testing.
- Subjects may receive (PRN) prophylactic medications prior to infusion of study drug (outlined in the protocol) to minimize the risk of immunologic reaction or infusion reaction during or shortly after the infusion.
- Subjects who initiate treatment or undertake dose adjustment with drugs not intended for treatment of cognitive impairment during the study may continue in the study if in the opinion of the Principal Investigator this will not interfere with study procedures or subject safety and are not prohibited above.

Extension Phase: (revised per Amendment 11)

- The following therapies are not permitted for the listed intervals from Extension Phase Baseline (Extension Screening Visit) until the Follow-Up Visit (Visit 97, Extension Week 117).
    - Immunoglobulin therapy, 6 months
    - Biologic drugs (except BAN2401), 6 months
- Subjects who require treatment with thrombolytic drugs do not have to be discontinued from the study, but study drug will be temporarily suspended for these subjects during thrombolytic therapy until stabilization or resolution of the medical condition which required thrombolytic drugs treatment.
- Subjects who are on anticoagulants at Extension Phase Baseline (Extension Screening Visit) are required to have their anticoagulation status optimized and stable for at least 4 weeks before Extension Screening Visit.
- Subjects who initiate AD treatment or who adjust their AD dosing regimen while on study will continue to study completion.

The following requirements apply to all other medications:

- Subjects must be on a stable dose for at least 4 weeks prior to Extension Phase Baseline (Extension Screening Visit), except for medications which are administered as short courses of treatment (eg, anti-infectives) or which are to be used as needed.
- Medications which are used on a PRN basis or as a short course of treatment, and which are CNS active

and may affect cognitive function are not permitted during a period of 72 hours prior to cognitive testing.

- Subjects may receive PRN prophylactic medications prior to infusion of study drug (outlined in the protocol) to minimize the risk of immunologic reaction or infusion reaction during or shortly after the infusion.
- Subjects who initiate treatment or undertake dose adjustment with drugs not intended for treatment of cognitive impairment during the study may continue in the study if in the opinion of the Principal Investigator this adjustment will not interfere with study procedures or subject safety and are not prohibited above.

Assessments:

Efficacy Assessments - (Core Study and Extension Phase)

ADCOMS: This composite clinical score represents a new approach to the analysis of selected items (12 total) from 3 fully validated and well-established clinical tools, including the MMSE, the CDR, and the ADAS-Cog. The data from 4 studies, including the Alzheimer's Disease Neuroimaging Initiative (ADNI) (MCI subset), ADCS-008, E2020-A001-412 and E2020-E033-415 have been used in a statistically validated model aimed at optimizing sensitivity to disease progression over time in the MCI population. The MMSE, the CDR, and the ADAS-Cog will each be administered to subjects using standard methods, and results will be used to calculate the ADCOMS.

Pharmacokinetic Assessments - (Core Study and Extension Phase)

Blood samples will be collected from all subjects for determination of serum BAN2401 levels at approximately 12-week intervals during the Randomization Phase.

Blood for serum PK will be collected at Extension Week 1 (Visit 44), Extension Weeks 3, 9, 13, 27, 39, 53, 79, 103 (last dosing visit), 105, and 117. (revised per Amendments 08 and 11)

Pharmacogenomic and Biomarker Assessments - (Core Study and Extension Phase)

Apolipoprotein ϵ4 variant (*APOE4*) genotyping will be conducted to allow stratification by *APOE* status (*APOE4* positive and negative). *APOE4* homozygous or heterozygous status will be used in the statistical analysis to determine the effects on treatment response and safety, including the development of ARIA, which include vasogenic edema, microhemorrhages and superficial hemosiderosis. Remaining DNA from the *APOE4* genotyping may be used to examine the role of DNA sequence variability in the absorption, distribution, metabolism, and elimination of BAN2401. Variations in BAN2401 exposure or the occurrence of AEs observed in the study population may be evaluated by correlation of single-nucleotide polymorphisms with PK, safety, or pharmacodynamic (PD) data.

Pharmacogenomic (PG) and biomarker samples obtained from participants of this study may be analyzed by global proteomic, metabolomic, or lipidomic and single or multiplex assays in an effort to identify predictive biomarkers for PK and PD. In addition, biomarkers identified in other BAN2401 or AD clinical studies may also be assessed in samples collected from subjects enrolled in this study.

vMRI imaging will be used to evaluate the effects of BAN2401 on rates of atrophy in the EAD population to provide evidence for disease modification. All subjects will undergo a vMRI imaging sequence immediately following all safety MRI assessments except the Visit 6 abbreviated MRI assessment (safety MRI to detect ARIA lasting 8 to 10 minutes, with no vMRI sequences) conducted only at European sites (revised per Amendment 07). vMRI sequences will be analyzed at the Screening Visit and at Visits 16, 29, and 42 (6, 12, and 18 months of treatment) during the Core Study. vMRI sequence collections will occur at all safety MRI assessments during the Extension Phase. (revised per Amendment 08) Total hippocampal, whole brain, and ventricular volumes will be assessed.

CSF concentrations of AD-related biomarkers (including, but not restricted to Aß and other isoforms, total tau [t-tau] and phospho-tau [p-tau]), will be measured in consenting subjects at Baseline and at 12 and 18 months of treatment during the Core Study only . (revised per Amendments 01 and 11)

Safety Assessments

Safety will be assessed by monitoring and recording all AEs and SAEs, monitoring of hematology, blood chemistry

and urinalysis, measurement of vital signs, ECGs, brain MRI, and the performance of physical examinations.

Core Study

During the Screening Period and at 3-month intervals during the Randomization Phase, all subjects will undergo noncontrast brain MRI, including FLAIR, Gradient Recalled Echo (GRE), T1 and diffusion-weighted sequences to determine the presence of focal lesions including, but not limited to, evidence for ischemic and hemorrhagic stroke, subdural hematoma, neoplasm, arteriovenous (AV) malformation, micro- and macrohemorrhages, superficial siderosis, and vasogenic edema. The results of this MRI assessment will also be used as the Baseline Period MRI, against which changes after drug administration will be compared. Details of the MRI protocol can be found in the IAG. Safety brain MRI assessments will be performed at Visit 7, every 3 months postdose, and as clinically necessary (in unscheduled visits). (revised per Amendment 04) MRI acquisition and interpretation will be organized centrally to ensure reproducibility and consistency between units involved.

The principal investigator is required to evaluate any immunological reactions to the study medication based on clinical findings and laboratory results from hematology, biochemistry and urinalysis, and decide if the subject should be withdrawn from treatment or receive prophylactic medications prior to the next infusion. Blood for anti-BAN2401 antibody assessments will be collected during the treatment period as specified in the Schedule of Assessments.

At any time during the treatment period, subjects will be withdrawn from further dosing if they develop any of the following features on MRI: vasogenic edema, macrohemorrhage, an area of superficial siderosis or a symptomatic microhemorrhage. If discontinued from study drug, subjects will undergo an Early Termination Visit (within 7 days after discontinuation) and will attend the Follow-up Visit (3 months after this decision). They will also undertake an unscheduled visit (with MRI) at approximately 30 days after the visit at which such MRI features were first identified. MRI will also be performed at approximately the time of the Follow-up Visit. Additional visits for safety may be arranged if clinically indicated in the opinion of the investigator, including after 90 days since the final dose of study drug. In addition, subjects who discontinue from study drug are expected to return after the Early Termination Visit for each scheduled visit when the clinical efficacy assessments are to be conducted per the Schedule of Procedures/Assessments At these visits, safety information (in addition to efficacy assessments) on concomitant medications, AEs, and SAEs will be collected. (revised per Amendment 01)

At any time during the Treatment Period, subjects who have nonsymptomatic, treatment-emergent microhemorrhages found on MRI, but none of the features which merit discontinuation of treatment (ie, vasogenic edema, macrohemorrhage, an area of superficial siderosis, or a symptomatic treatment-emergent microhemorrhage) may continue with treatment. These subjects will have safety assessments (including MRI) at approximately 30 days after the visit at which such MRI features were found. They will also continue with the scheduled MRI scans. The safety MRI must be reviewed by both the imaging vendor and a local reader prior to administration of the next dose of study drug for each respective visit. (revised per Amendment 06) Additional unscheduled MRI scans may be arranged on an individual subject basis in discussion with the study medical monitor.

An assessment of suicidality using the C-SSRS will be performed at Screening and Baseline and at designated time points.

Extension Phase (revised per Amendments 08 and 11)

During the Extension Phase, safety assessments will be performed according to the Extension Phase Schedule of Assessments and all AEs and SAEs recorded

Other Assessments

Amyloid PET and CSF Aβ(1-42)

Core Study

Amyloid PET imaging or CSF Aβ(1-42) assessment will be used to confirm that all subjects with EAD have amyloid deposition in the brain. (revised per Amendment 06) This criterion will allow for the definition of subjects with MCI due to AD – intermediate likelihood and will confirm amyloid pathology in mild Alzheimer's disease dementia subjects.

Amyloid PET or CSF Aβ(1-42) assessment is required at Baseline during the Prerandomization Phase for all subjects to qualify for study inclusion per the protocol, and subjects who consent to participate in the imaging subgroup (target n=306 total) will receive amyloid PET imaging at 12 and 18 months of treatment. Duration of the PET scan and its timing relative to injection of the imaging agent will be as per the imaging agent manufacturer's guidance. (revised per Amendments 02, 06, and 07)

Extension Phase (revised per Amendments 08, 11, and 12)

All subjects who underwent amyloid PET for inclusion in the Core Study should receive a baseline amyloid PET scan before dosing in the Extension Phase. The baseline amyloid PET scan must be conducted with the same imaging tracer that was used for inclusion at the baseline visit for the Core Study. In addition, qualified subjects located in the US and Japan will have the option to participate in the longitudinal PET substudy. Florbetapir will be used in the US and flutemetamol will be used in Japan for longitudinal amyloid PET analysis. At Extension Screening Visit, subjects who consented to the longitudinal imaging substudy will be stratified into 2 cohorts based on their treatment allocation during the Core Study. Cohort 1 will have amyloid PET assessments performed at baseline (Extension Screening Visit), Visit 50 (Extension Week 13), Extension Phase Visit 70 [Extension Week 53], and Visit 96 [Extension Week 105]; Cohort 2 will have amyloid PET assessments performed at baseline (Extension Screening Visit), Visit 57 (Extension Week 27), at Extension Phase Visit 70 [Extension Week 53], and Visit 96 [Extension Week 105]. In Japan, those who consented to the longitudinal imaging substudy will only undergo amyloid PET at Extension Phase Visit 70 [Extension Week 53], and Visit 96 [Extension Week 105].

Exploratory Plasma Biomarkers (revised per Amendment 11)

Blood will be collected from subjects at Baseline during the Prerandomization Phase prior to amyloid PET assessment and at 12 and 18 months of treatment to evaluate potential novel biomarkers of AD that may include amyloid isoforms, tau, microRNA, metabolites, and other protein biomarkers (eg, NFL) for association with AD diagnosis and amyloid load. Similarly, biomarker discovery and validation may be performed along with samples from AD subjects, to identify blood and genetic biomarkers which may be useful to predict subject PK and PD responses, treatment response, subject stratification or adverse effects related to BAN2401.

Bioanalytical Methods:

Cerebrospinal fluid levels of Aβ(1-42), t-tau, and p-tau will be assessed. Aβ(1-42), tau and p-tau biomarkers are proposed to be tested using commercially available kits validated for use by a CRO.

Serum concentrations of BAN2401 will be measured by the validated immunoprecipitation- liquid chromatography – tandem mass spectrometry (IP/LC-MS/MS) methods using anti-human IgG antibody to precipitate BAN2401 from a serum sample. CSF concentrations of BAN2401 will be measured by a separately validated IP/LC-MS/MS method. Precipitated BAN2401 will be isolated and will undergo proteolytic enzyme digestion to yield smaller peptides. The amount of peptide with a sequence unique to BAN2401 will be measured by liquid chromatography-tandem mass spectrometry (LC-MS/MS) to provide a quantification of BAN2401.

Statistical Methods: All statistical tests will be based on the 10% level of significance, except for the Bayesian methods on the primary endpoint . (revised per Amendments 09 and 10)

Study Endpoints

Core Study

Primary Endpoint:

- Change from baseline in the ADCOMS at 12 months

Key Secondary Endpoints: (revised per Amendments 09 and 10)

- Change from baseline at 18 months in brain amyloid pathophysiology as measured by amyloid PET
- Change from baseline in the ADCOMS at 18 months (revised per Amendment 09)
- Change from baseline in CDR-SB at 18 months
- Change from baseline in ADAS-Cog at 18 months

- Change from baseline in CSF biomarkers (Aβ[1-42], t-tau, and p-tau) at 18 months (revised per Amendment 10)
- Change from baseline in total hippocampal volume at 18 months using vMRI

Secondary Endpoints: (revised per Amendment 10)

- Change from baseline at 12 months in brain amyloid pathophysiology as measured by amyloid PET
- Change from baseline at 12 months on clinical status for the following assessments: ADCOMS, CDR-SB, and ADAS-Cog
- Change from baseline in CSF biomarkers (Aβ[1-42], t-tau, and p-tau) at 12 months
- Change from baseline in total hippocampal at 6 and 12 months, and in left and right hippocampus, whole brain, and total ventricular volume as measured by vMRI at 6, 12, and 18 months

Exploratory Endpoints:

- Change from baseline in clinical status at time points not analyzed in Key Secondary and Secondary sections for each of the following: ADCOMS, ADAS-Cog, CDR-SB, MMSE, and FAQ

Extension Phase (revised per Amendments 08 and 11)

Primary Endpoint

- Safety assessments will be based on medical review of AE reports and the results of vital sign measurements, ECG, physical examinations, clinical laboratory tests, anti-drug antibody (ADA) test results, and any relevant test of cognitive function to evaluate decline. Additionally, MRI assessments of microhemorrhage, vasogenic edema, and other clinically significant abnormalities will be evaluated.

Secondary Endpoints (revised per Amendment 11)

- Change from baselines in brain amyloid levels as measured by amyloid PET at 3 months (Visit 50 [Extension Week 13], Cohort 1) or 6 months (Visit 57 [Extension Week 27], Cohort 2), and 12 and 24 months in the Extension Phase (Visit 70 [Extension Week 53], Visit 96 [Extension Week 105])
- Change from end of Core Study in brain amyloid levels as measured by amyloid PET at the baseline of Extension Phase
- Proportion of amyloid positive subjects over time

Exploratory Endpoints

- Change from baselines in ADCOMS, CDR-SB, ADAS-Cog, and MMSE at each visit assessed (revised per Amendment 11)
- Time to worsening of CDR global scores, eg, the worsening of global CDR score is defined as an increase from baseline by at least 0.5 points on the global CDR scale on 2 consecutive scheduled visits at which global CDR is measured
- Change from baselines in total hippocampal volume and other biomarkers at 12, and 24 months in the Extension Phase (Visit 70 [Extension Week 53], Visit 96 [Extension Week 105]). (revised per Amendment 11)

Analysis Sets

Core Study

- The **Randomized Set** is the group of subjects who are randomized to study drug.
- The **Safety Analysis Set** is the group of subjects who receive at least 1 dose of study drug and have at least 1 postdose safety assessment.
- The **Full Analysis Set** is the group of randomized subjects who receive at least 1 dose of study drug and

have baseline and at least 1 postdose primary efficacy measurement.

- The **PK Analysis Set** is the group of subjects with at least 1 quantifiable BAN2401 serum concentration with a documented dosing history.
- The **PD Analysis Set** is the group of subjects who have sufficient PD data to derive at least 1 PD parameter.
- The **Per Protocol (PP) Analysis Set** is the subset of subjects in the Full Analysis Set who sufficiently complied with the protocol.

Extension Phase (revised per Amendments 08 and 11)

- The **OLE Full Analysis Set (OLE-FAS)** is the group of subjects who receive at least 1 dose of study drug during the Extension Phase and who have a baseline assessment and at least 1 postdose ADCOMS measurement during the Extension Phase. The baseline assessment is defined as the last measurement before 1st dose of BAN2401.
- The **OLE Safety Analysis Set** is the group of subjects who receive at least 1 dose of study drug during the Extension Phase and have at least 1 postdose safety assessment during the Extension Phase.
- The **OLE PK Analysis Set** is the group of subjects with at least 1 quantifiable BAN2401 serum concentration with a documented dosing history during the Extension Phase.
- The **OLE PD Analysis Set** is the group of subjects who have sufficient PD data to derive at least 1 PD parameter during the Extension Phase.

Efficacy Analyses

Core Study

In this Phase 2 dose-finding study of BAN2401 for the treatment of Early Alzheimer's disease, response adaptive randomization will be used to allocate subjects to placebo or 1 of the 5 active dose regimens with the goal of characterizing the dose response. The study will be monitored for early success and early futility. If the study continues to randomize subjects after futility is declared, it will be considered a failed study. (revised per Amendment 01)

Key definitions relevant to the Bayesian aspects of the adaptive design are given below.

Study Definitions

Clinically Significant Difference (CSD): The clinically significant difference used in the adaptive model relative to the change from baseline over time for placebo in the ADCOMS. The CSD for this study represents an approximate 25% reduction compared to placebo in the progression of AD as measured by the ADCOMS.

Maximum Effective Dose (d_{Max}): The dose that achieves the greatest treatment effect

ED_{90}: The 'simplest' dose regimen that achieves at least 90% of the d_{Max} treatment effect. The term 'simplest' reflects the preference for dosing frequency and amount when considering the treatment arms for the study and while taking efficacy into account (monthly administration is preferred over biweekly administration and lower doses are preferred over higher doses, with priority placed on frequency of administration). Thus, the rank order of prioritized doses (beginning with the simplest) is: 5 mg/kg Q4wk, 10 mg/kg Q4wk, 2.5 mg/kg Q2wk, 5 mg/kg Q2wk, and 10 mg/kg Q2wk.

Early Success: The study meets early success criteria if the probability that the ED_{90} at 12 months of treatment is better than the placebo by the CSD is at least 0.95. Early success is achieved before all subjects complete 12 months of treatment and can only be determined once ≥350 subjects are randomized. If early success is declared prior to full randomization, the randomization of new subjects will stop, outside Japan. (revised per Amendment 08) Sites in Japan will be allowed to randomize at least 40 Japanese subjects, even if the early success is declared or the study achieves 800 subjects randomized. All randomized Japanese subjects will receive up to 18 months of treatment in the Core Study and are eligible to participate in the Extension Phase if implemented. (revised per Amendments 07, 08, and 11)

Futility: The study meets statistical futility criteria if the probability that the ED_{90} is better than the placebo by the

CSD is less than 0.05 (with ≤300 subjects randomized) or 0.075 (with ≥350 subjects randomized) at 12 months of treatment. If the study meets statistical futility criteria, the sponsor will make the final decision pertaining to study futility after reviewing recommendations by the DSMB. Based on sponsor's final decision, if the study continues to randomize subjects, it will be considered a failed study. (revised per Amendment 01)

Study Success: The study will be considered a success if either of the following criteria are met for the 12-month treatment endpoint:

- The study meets early success criteria (as outlined above)
- The study reaches complete randomization and the Bayesian analysis at completion of 12 months of treatment results in an ED_{90} with at least an 80% probability of being better than the placebo by the CSD (revised per Amendment 09)

Analysis for the Primary Endpoint

The primary analysis will be based on subjects from the Full Analysis Set with the prespecified censoring rules applied. Japanese subjects who are randomized after early success is declared or the study achieves 800 subjects randomized will not be included in the primary analysis. (revised per Amendment 07) The primary endpoint is the change from baseline to 12 months in the ADCOMS. The dose-response of the primary endpoint is modeled with a 2-dimensional first-order normal dynamic linear model, where Normal and Inverse-Gamma priors are used. The primary efficacy analysis will calculate the Bayesian posterior probability that the dose identified is the most likely ED_{90} dose that achieves the CSD compared to the placebo arm. At each analysis, 3 Bayesian probabilities will be summarized for each active dose: the probability of being the ED_{90} dose, the probability of being statistically superior to placebo and the probability of being statistically superior to placebo by the CSD. The study will be considered a success in the Bayesian analysis at the completion of 12 months of treatment if there is at least an 80% probability that the ED_{90} achieves the CSD from placebo. (revised per Amendment 09)

For the clinical efficacy data (ie, derived Clinical Composite Score, MMSE, CDR-SB, ADAS-Cog, and FAQ), subjects will be censored at the time of initiation of new AChEIs or memantine treatment regimens if they were not on AChEIs or memantine at randomization, and will be censored at the time of dose adjustment of AChEIs or memantine if they were already on stable treatment of AChEIs or memantine at randomization. The value of the primary endpoint for censored subjects will be imputed using data up to the censoring time and Bayesian imputation methods.

After unblinding, the primary endpoint will be analyzed using the Bayesian methods described above as well as conventional statistical methods. The following additional Bayesian analyses will be conducted as sensitivity analyses (revised per Amendment 01):

- The primary endpoint will be analyzed regardless of initiation of new AChEIs or memantine or dose adjustment of stable AChEIs or memantine using the same Bayesian method as in the primary analysis. (revised per Amendment 01)
- In case of early success, the primary analysis as well as the above sensitivity analyses will be repeated after all subjects have completed 12 months of follow up or have been lost to follow up.

Statistical methods for the conventional analyses of primary efficacy endpoint will use a mixed effects model with repeated measures (MMRM) comparing placebo to the identified ED_{90} dose from the Bayesian analysis. (revised per Amendment 09) The model will include randomization stratification variables; clinical subgroup (MCI due to AD, mild AD), the presence or absence of ongoing AD treatment (ie, AChEIs or memantine or both), *APOE4* status (positive, negative) and baseline ADCOMS as covariates. These analyses will be performed on the full analysis set as well as the per protocol set based on the ITT principle. In case of early success, the conventional analyses will also be performed using the data collected prior to the IA. (revised per Amendment 01)

Analysis for the Key Secondary Endpoints

Key Secondary endpoints were ordered to reflect the most recent FDA Draft Guidance and EMA Final Guideline that prioritizes effects on pathophysiology in early AD, as measured by biomarkers (February, 2018). Conventional analysis will be performed using all available data at the time of success (if applicable), and at 18 months of

treatment, regardless of success. An MMRM will be used to compare the combined 10 mg dose group (including biweekly and monthly regimens) with placebo for the key secondary endpoints: (revised per Amendments 09 and 10) The rationale for combining the two 10-mg dose regimens is to account for the loss of subjects positive for *APOE4* in the 10 mg/kg biweekly dose group and the inability to randomize *APOE4* carriers to the 10 mg/kg biweekly group following a Regulatory request by European Health Authorities in July, 2014. The key Secondary endpoint based on clinical status (ADCOMS, CDR-SB, and ADAS-Cog) will be analyzed in subjects with EAD as well as separately within subjects with MCI and mild AD dementia. (revised per Amendment 10)

In addition, as sensitivity analysis, MMRM analysis will be performed for the following treatment comparisons: (revised per Amendment 10)

- Combining 2 high doses (10 mg biweekly + 10 mg monthly), 2 middle doses (5 mg biweekly + 5 mg monthly), and the 2.5 mg biweekly dose, resulting in 3 treatment comparisons with placebo (for dose response) (revised per Amendment 10)
- ED_{90} dose of BAN2401 versus placebo, where the ED_{90} dose of BAN2401 will be established by Bayesian analysis at 18 months as described in the next paragraph (revised per Amendment 10)
- By treatment regimen (10 mg biweekly, 10 mg monthly, 5 mg biweekly, 5 mg monthly, 2.5 mg biweekly, placebo) (revised per Amendment 10)
- Combining the top 3 doses (10 mg biweekly, 10 mg monthly, and 5 mg biweekly) versus placebo (revised per Amendment 10)

Change from baseline in the ADCOMS at 18 months will be analyzed, as a sensitivity analysis to the conventional analysis, using the same Bayesian methodology as that for analysis of change from baseline in the ADCOMS at 12 months, but using the full 18 months of efficacy data with the model projecting to 18 months of treatment. The Bayesian analysis of change from baseline in the ADCOMS at 18 months is positive if the analysis results in an ED_{90} dose with at least an 80% probability of being better than the placebo by the CSD. The ED_{90} dose will be identified based on change from baseline in the ADCOMS at the 18 month Bayesian analysis and will be used to compare ED_{90} dose of BAN2401 and placebo in the above sensitivity analyses for all key secondary endpoint analyses. (revised per Amendments 09 and 10)

<u>Analysis for Other Secondary Endpoints</u>

The same analysis described above for key secondary endpoints will be performed for other secondary endpoints. (revised per Amendment 10)

<u>Analysis for Exploratory Endpoints</u>

The same analysis described above for key secondary endpoints will be performed for exploratory endpoints. (revised per Amendment 10)

The effect of baseline brain amyloid pathopohysiology as measured by amyloid PET on change from baseline in clinical status at 12 and 18 months of treatment will be explored using correlation analysis and evaluated using statistical models as appropriate. (revised per Amendment 10)

The relationship between change from baseline in clinical status and change from baseline in brain amyloid pathopohysiology at 12 and 18 months of treatment as measured by amyloid PET will be explored by correlation analysis. (revised per Amendment 10)

Change in ADCOMS and its components, ADAS-Cog, CDR-SB and MMSE across all clinical assessment time points between BAN2401 and placebo in Japanese subjects will be evaluated using the descriptive statistics within Japanese subjects as well as with the overall population. (revised per Amendments 07 and 10)

<u>Extension Phase (revised per Amendments 08 and 11)</u>

An MMRM will be used to analyze the change from baseline secondary efficacy endpoints. Proportion of amyloid positive subjects over time will be summarized. MMRM will also be used to analyze the following exploratory endpoints:

- Change from baseline in the ADCOMS, CDR-SB, ADAS-Cog score, and MMSE score

For each endpoint at a scheduled visit in the Extension Phase, a 90% 1-sided confidence interval will be constructed to test the null hypothesis that the treatment difference between subjects who took placebo in Core Study and those who took 10 mg/kg biweekly in the Core Study is no more than 50% of the treatment difference at the end of Core Study. If the lower limit of this confidence interval is greater than 0, the null hypothesis is rejected. In addition, comparisons between subjects who took placebo at Core Study and those who took 10 mg/kg biweekly in the Core Study will be performed for each endpoint at scheduled visits in the Extension Phase. (revised per Amendment 11)

Time to worsening of CDR global scores will be analyzed on the FAS using Cox regression model. Time to worsening of CDR global score is defined as time from the first treatment in the Extension Phase to worsening of the CDR score (ie, the first worsening in 2 consecutive scheduled visits). For subjects whose CDR scores have not worsened by the end of study, the time to worsening of the CDR score will be censored at the date of last CDR assessment for these subjects. (revised per Amendment 11)

Change from baseline in total hippocampal volume and other biomarkers will be summarized using descriptive statistics at scheduled visits in the Extension Phase.

Additional analyses and summaries will be performed as appropriate.

Pharmacokinetic and Pharmacodynamic Analyses (Core Study and Extension Phase)

Pharmacokinetics:

The Safety Analysis Set will be used for individual BAN2401 serum and CSF concentration listings (Core Study only). The PK Analysis Set will be used for summaries of BAN2401 serum and CSF concentrations (Core Study only). (revised per Amendment 11)

A population PK approach will be used to characterize the PK of BAN2401. The effect of covariates (ie, demographics) on BAN2401 PK will be evaluated. The PK model will be parameterized for clearance (CL) and volumes of distribution. Derived exposure parameters such as area under the concentration-time curve (AUC) will be calculated from the model using the individual posterior estimate of CL and dosing history.

Pharmacodynamics:

The PD Analysis Set will be used for the summaries and analyses of CSF biomarkers (Core Study only). (revised per Amendment 11) CSF Aβ(1-42), t-tau, and p-tau will be assessed and data presented graphically. Baseline levels and changes in t-tau and p-tau will be correlated with changes in CSF Aβ(1-42), imaging markers, and *APOE4* status.

Summary statistics for CSF biomarkers will be assessed for evidence of a dose relationship.

Pharmacokinetic-Pharmacodynamic:

The PK/PD relationship between CSF biomarker levels (Core Study only) and serum PK parameters or CSF concentrations of BAN2401 will be explored graphically and any emergent relationship will be explored through population PK/PD modeling. (revised per Amendment 11) These serum PK parameters include steady state (SS), maximum observed concentrations (C_{max}), and

$AUC_{(0-t)}$ derived from the population PK model. The PK/PD relationship between serum PK parameters and CSF concentrations of BAN2401 with other biomarkers may also be explored using similar methods.

Additionally, the relationship between various serum PK parameters or CSF concentrations of BAN2401 (Core Study only) and the ADCOMS at 12, 18, 30, and 42 months, and the relationship between various serum PK parameters or CSF concentrations of BAN2401 (Core Study only) and the change from Baseline for 12, 18, 30, and 42 months in the ADAS-Cog, the CDR, and the MMSE will be explored graphically. (revised per Amendments 08 and 11) Any emergent relationship will be explored through population PK/PD modeling.

The relationship between exposure to BAN2401 and most frequent AEs will also be explored.

Safety Analyses

Core Study

Evaluations of safety will be performed on the Safety Analysis Set of the Core Study. (revised per Amendment 08) The incidence of AEs (including changes from baseline in physical examination), out-of-normal-range laboratory safety test variables, abnormal ECG findings, out-of-range vital signs, and suicidality (C-SSRS), along with change from baseline in laboratory safety test variables, ECGs, brain MRI, ADAs (screening and neutralizing) and vital sign measurements, will be summarized by treatment group using descriptive statistics.

Extension Phase (revised per Amendment 08)

Safety analysis will be performed similarly to the Core Study. All safety analyses will be based on Safety Analysis Set of the Extension Phase. The incidence of treatment emergent adverse events (TEAEs), laboratory test variables, ECG findings, vital signs, brain MRI, and ADAs will be summarized using descriptive statistics.

Interim Analyses (Core Study only) (revised per Amendment 11)

An unblinded independent Interim Monitoring Committee (IMC) will provide oversight to ensure that the response adaptive randomization process and interim analyses perform as expected. An independent data analysis group will perform all of the interim analyses and provide the results to the IMC.

Randomization to placebo or 1 of 5 dose arms of BAN2401 will be fixed for the first 196 subjects randomized in the study (4:2:2:2:2:2; placebo [4] to each of the active arms [2 each]). After this initial burn-in, adaptive randomization will begin. Randomization probabilities to each arm will be updated at each interim analyses such that the randomization probability will be increased for the placebo arm and arms that represent the potential target dose (ED_{90}), and simultaneously decreased for other active arms. Subjects who are confirmed *APOE4* positive (*APOE4* hetero- or homozygous) will not be randomized to the 10 mg/kg, biweekly dose. (revised per Amendment 05) Details of the estimation of the randomization probabilities will be presented in the Statistical Analysis Plan.

The study will be monitored for early success and early futility (see definitions in Study Definitions). The first IA will be conducted when 196 subjects are randomized, again when 250 subjects are randomized, and after each additional 50 subjects until all 800 subjects are randomized. Sites in Japan will randomize at least 40 Japanese subjects, even if early success is declared or the study achieves 800 subjects randomized. In these circumstances, subjects in Japan will be allocated treatment according to the latest (or last) allocation schedule. If the study reaches its maximum of 800 randomized subjects, additional interim analyses will be conducted every 3 months until all subjects complete 12 months of treatment. Only those subjects who were randomized before the target of 800 was met will contribute to these additional interim analyses, except for the full 12 and 18 month Bayesian analyses. The study will be monitored for futility at each IA and for success starting at the 350-subject IA, until the full 12 months of treatment have been completed. (revised per Amendment 09) Each IA will be conducted on data for ADCOMS, which is the derived score optimized to capture AD-related clinical progression. If the efficacy or futility boundary is reached, the IMC will communicate directly with the DSMB members. (revised per Amendment 07) The study may be stopped for safety reasons at any time.

Sample Size Rationale

Core Study

Primary Endpoint - ADCOMS

The sample size and design characteristics to test the hypothesis under primary objective based on the primary endpoint, the ADCOMS at 12 months, were determined by means of simulations. Extensive trial simulations have shown that a total of 800 subjects will be sufficient to demonstrate that the most likely ED_{90} dose achieves a CSD from placebo with a probability of at least 95% in the interim analyses and greater than 80% in the final analysis if the trial does not stop for early success. There is at least 80% probability of study success for the dose response scenarios where the treatment was considered to have a clinically meaningful treatment effect. Simulations have also shown that if there is no efficacy at all for any dose, the probability of falsely claiming superiority to placebo is no more than 10.0% assuming a 20% dropout rate.

For each of the 6 treatment groups (5 active dose regimens and placebo), the final number of subjects per group will differ depending on the observed interim treatment responses. The simulation plan will be described in the

Appendix section of the Statistical Analysis Plan, which will present further details.

vMRI

The null hypothesis is that there is no difference between active dose and placebo. There are 5 null hypotheses corresponding to 5 active dose regimens. The alternative hypothesis is that at least 1 hull hypothesis is false (1-sided). The null hypotheses will be tested using the Dunnett method with 1-sided alpha of 0.05. The statistical power was estimated through simulation for a moderate dose-response assumption that the percent reduction in change from baseline of total hippocampal volume compared to placebo would be 15%, 20%, 25%, 15%, and 20% corresponding to the 5 dose regimens (2.5, 5, and 10 mg/kg biweekly, 5 and 10 mg/kg monthly interval), respectively. The estimated power for actual planned study sample size is 0.764 at 12 months and 0.694 at 18 months assuming an attrition rate of 20% at 12 months and an exponential dropout model. Under a stronger dose-response assumption (ie, the percent reduction in change from baseline of total hippocampal volume compared to placebo is twice as much as that in the moderate dose-response assumption), the estimated power is at least 99% at both 12 and 18 months.

Amyloid PET (Imaging Subgroup)

The null and alternative hypotheses and statistical test are the same as that for vMRI. To estimate the sample size for amyloid PET imaging subgroup at 18 months, a common standard deviation of change from baseline was estimated as 0.4 and a reasonable mean difference between treatment and placebo was estimated as 0.25 standard uptake value ratio (SUVR). The corresponding standard deviation and mean difference at 12 months were estimated as 0.27 and 0.17, respectively. Assuming a moderate dose response that the best dose regimen would achieve a difference of 0.25 (SUVR) and that the 2 middle and 2 low dose regimens would achieve a difference of 0.2 and 0.15 (SUVR), respectively, it would require a total 230 subjects from all 6 arms at 18 months to achieve an 80% power. This sample size took into account possible unequal number of subjects per arm. Under the same attrition rate assumption as that for vMRI, a total sample size of 306 at 12 months is sufficient to obtain 230 subjects at 18 months. The estimated power with 306 subjects at 12 months is approximately 85%. The postbaseline amyloid PET in the imaging subgroup will be planned for the first 306 subjects who have consented and who are still on treatment at 12 months. (revised per Amendments 02 and 07)

Extension Phase (revised per Amendments 08 and 11)

There is no sample size calculation for the Extension Phase.

3 TABLE OF CONTENTS

REVISION HISTORY i
1 TITLE PAGE 1
2 CLINICAL PROTOCOL SYNOPSIS 2
3 TABLE OF CONTENTS 26
LIST OF IN-TEXT TABLES 29
LIST OF IN-TEXT FIGURES 29
LIST OF APPENDICES 29
4 LIST OF ABBREVIATIONS AND DEFINITIONS OF TERMS 30
5 ETHICS 33
5.1 Institutional Review Boards/Independent Ethics Committees 33
5.2 Ethical Conduct of the Study 34
5.3 Subject Information and Informed Consent 34
6 INVESTIGATORS AND STUDY PERSONNEL 36
7 INTRODUCTION 37
7.1 Indication 37
7.1.1 Current Therapeutic Options 37
7.1.2 BAN2401 37
7.1.2.1 Therapeutic Pathway 37
7.1.2.2 Mechanism of Action 38
7.1.2.3 Clinical Experience with BAN2401 38
7.2 Study Rationale 42
7.2.1 Clinical Assessment Rationale 43
7.2.1.1 Rationale for Conducting the Study in MCI due to AD – Intermediate Likelihood and Mild Alzheimer's Disease Dementia Subjects 43
7.2.1.2 Efficacy Assessment – Composite Clinical Score 44
7.2.2 Biomarker Rationale 46
7.2.2.1 Amyloid PET 46
7.2.2.2 Volumetric MRI 47
7.2.2.3 Soluble Biomarkers in Cerebrospinal Fluid (CSF) 48
8 STUDY OBJECTIVES 49
9 INVESTIGATIONAL PLAN 52
9.1 Overall Study Design and Plan 52
9.1.1 Prerandomization Phase 55
9.1.1.1 Screening Period 56
9.1.1.2 Baseline Period 58
9.1.2 Randomization Phase 59
9.1.2.1 Treatment Period 59
9.1.2.2 Extension Phase 61
9.1.2.3 Follow-up Period 61

9.1.2.4 Unscheduled Visits 62
9.2 Discussion of Study Design, Including Choice of Control Groups 62
9.3 Selection of Study Population 63
9.3.1 Inclusion Criteria 63
9.3.2 Exclusion Criteria 66
9.3.3 Removal of Subjects From Therapy or Assessment 68
9.4 Treatments 69
9.4.1 Treatments Administered 69
9.4.2 Identity of Investigational Product(s) 70
9.4.2.1 Chemical Name, Structural Formula of BAN2401 70
9.4.2.2 Comparator Drug 70
9.4.2.3 Labeling for Study Drug 71
9.4.2.4 Storage Conditions 71
9.4.3 Method of Assigning Subjects to Treatment Groups 71
9.4.4 Selection of Doses and Dose Regimens in the Study 72
9.4.5 Selection and Timing of Dose for Each Subject 74
9.4.6 Blinding 74
9.4.7 Prior and Concomitant Therapy 75
9.4.7.1 Drug-drug Interactions 75
9.4.7.2 Core Study Prohibited Concomitant Therapies and Drugs 75
9.4.8 Treatment Compliance 76
9.4.9 Drug Supplies and Accountability 76
9.5 Study Assessments 77
9.5.1 Assessments 78
9.5.1.1 Demography 78
9.5.1.2 Baseline Assessments 78
9.5.1.3 Efficacy Assessments 81
9.5.1.4 Pharmacokinetic, Pharmacodynamic, and Pharmacogenomic/Pharmacogenetic Assessments 82
9.5.1.5 Safety Assessments 85
9.5.1.6 Other Assessments 95
9.5.2 Schedule of Procedures/Assessments 96
9.5.2.1 Schedule of Procedures/Assessments 96
9.5.2.2 Total Volume of Blood and CSF 104
9.5.3 Appropriateness of Measurements 104
9.5.4 Reporting of Serious Adverse Events, Pregnancy, and Other Events of Interest 105
9.5.4.1 Reporting of Serious Adverse Events 105
9.5.4.2 Reporting of Pregnancy and Exposure to Study Drug Through Breastfeeding 106
9.5.4.3 Reporting of Other Events of Interest 106
9.5.4.4 Expedited Reporting 107
9.5.4.5 Breaking the Blind 108
9.5.4.6 Regulatory Reporting of Adverse Events 108

9.5.5 Completion/Discontinuation of Subjects 108
9.5.6 Abuse or Diversion of Study Drug 109
9.5.7 Confirmation of Medical Care by Another Physician 109
9.6 Data Quality Assurance 109
9.6.1 Data Collection 109
9.6.2 Clinical Data Management 110
9.7 Statistical Methods 110
9.7.1 Statistical and Analytical Plans 110
9.7.1.1 Study Endpoints 110
9.7.1.2 Definitions of Analysis Sets 111
9.7.1.3 Subject Disposition 112
9.7.1.4 Demographic and Other Baseline Characteristics 112
9.7.1.5 Prior and Concomitant Therapy 112
9.7.1.6 Efficacy Analyses 113
9.7.1.7 Pharmacokinetic, Pharmacodynamic, and Pharmacogenomic/Pharmacogenetic Analyses 117
9.7.1.8 Safety Analyses 118
9.7.1.9 Determination of Sample Size 120
9.7.2 Interim Analysis 121
9.7.3 Other Statistical/Analytical Issues 122
9.7.4 Procedure for Revising the Statistical Analysis Plan 122
10 REFERENCE LIST 123
11 PROCEDURES AND INSTRUCTIONS (ADMINISTRATIVE PROCEDURES) 126
11.1 Changes to the Protocol 126
11.2 Adherence to the Protocol 126
11.3 Monitoring Procedures 126
11.4 Recording of Data 127
11.5 Identification of Source Data 127
11.6 Retention of Records 127
11.7 Auditing Procedures and Inspection 128
11.8 Handling of Study Drug 128
11.9 Publication of Results 128
11.10 Disclosure and Confidentiality 129
11.11 Discontinuation of Study 129
11.12 Subject Insurance and Indemnity 129
12 APPENDICES 130

LIST OF IN-TEXT TABLES

Table 1	Mean (SD) of CSF and Serum Concentrations and CSF:Serum Ratios in Cohorts SAD6 (15 mg/kg) at 24 Hours Postdose, MAD1 (0.3 mg/kg), MAD2 (1 mg/kg), and MAD 3 (3 mg/kg) at 24 Hours Post-Dose 4, and MAD4 (10 mg/kg) at 24 Hours and 14 Days Post-Dose 7	40
Table 2	Summary of Treatment-Emergent ARIA-E by ApoE4 Status (Safety Analysis Set) (revised per Amendment 11)	42
Table 3	Sample Size Requirements for the ADCOMS Across 3 Treatment Effect Sizes Compared to ADAS-Cog and CDR in MCI Subjects and Enriched MCI Subjects	45
Table 4	BAN2401 Dose Regimens – Core Study	53
Table 5	Model-predicted CSF Exposures at Steady State at BAN2401 Doses of 2.5 to 10 mg/kg Biweekly, or 5 to 10 mg/kg Monthly	73
Table 6	Clinical Laboratory Tests (Revised per Amendment 11)	89
Table 7	Schedule of Screening and Baseline Procedures/Assessments (Visits 1 and 2), Study BAN2401-G000-201 – Core Study	98
Table 8	Schedule of Procedures/Assessments, Core Study BAN2401-G000-201, Panel A, Visits 3 through 27, Weeks 1 through 49 – Core Study	100
Table 9	Summary of Blood and CSF Sample Volumes	104
Table 10	Predictive Variables for the ADCOMS	114
Table 11	Summary of Blood Sample Volumes in the Extension Phase	148
**Table 12**	**Schedule of Procedures/Assessments for Study BAN2401-G000-201: Extension Phase**	151

LIST OF IN-TEXT FIGURES

Figure 1	Design of Study BAN2401-G000-201	55
Figure 2	Schematic Overview of Eligibility Assessments	56

LIST OF APPENDICES

Appendix 1	Sponsor's Grading for Laboratory Values	130
Appendix 2	List of Permitted and Prohibited Prior/Concomitant Medications	132
Appendix 3	Pharmacogenomic and Biomarker Bioanalysis	138
Appendix 4	EXTENSION PHASE (revised per Amendment 11)	141

4 LIST OF ABBREVIATIONS AND DEFINITIONS OF TERMS

Abbreviation	Term
Aβ	amyloid beta
Aβ(1-42)	amyloid beta monomer from amino acid 1 to 42
AChEI	acetylcholinesterase inhibitor
AD	Alzheimer’s disease
ADA	anti-drug antibody
ADAS-Cog	Alzheimer’s Disease Assessment Scale - Cognitive Subscale
ADCOMS	Alzheimer's Disease Composite Score
ADNI	Alzheimer’s Disease Neuroimaging Initiative
AE	adverse event
APOE	Apolipoprotein E
APOE4	apolipoprotein ϵ4 variant
ARIA	amyloid-related imaging abnormalities
ARIA-E	amyloid related imaging abnormality edema/effusion
ARIA-H	amyloid related imaging abnormality hemorrhage
ATC	Anatomical Therapeutic Chemical
AUC	area under the concentration vs. time curve
$AUC_{(0\text{-}14\ days)\_}$	area under the concentration vs. time curve between time 0 and 14 days
$AUC_{(0\text{-}\tau)}$	area under the concentration vs. time curve between time 0 and time τ at steady state, where τ is the dosing interval
$AUC_{(0\text{-}t)}$	area under the concentration vs. time curve between time 0 and time t
BAN2401	a humanized IgG1 monoclonal antibody
BMI	body mass index
CDR	Clinical Dementia Rating
CDR-SB	Clinical Dementia Rating – Sum of Boxes
CFR	Code of Federal Regulations
CL	clearance
C_{max}	maximum observed concentration
$C_{max,ss}$	maximum observed concentration at steady state
C_{min}	minimum observed concentration
$C_{min,ss}$	minimum observed concentration at steady state
CNS	central nervous system
CPMP	Committee for Proprietary Medicinal Products
CRA	Clinical Research Associate
CRP	C-reactive protein
CRO	contract research organization
CSD	clinically significant difference
CSF	cerebrospinal fluid
C-SSRS	Columbia-Suicide Severity Rating Scale
CV	curriculum vitae

Abbreviation	Term
d_{Max}	the dose that achieves the greatest treatment effect
DSMB	Drug Safety Monitoring Board
EAD	early Alzheimer's Disease
ECG	electrocardiogram
eCRF	electronic case report form
ED_{90}	the 'simplest" dose regimen that achieves at least 90% of the d_{Max} treatment effect
EU	European Union
FAQ	Functional Assessment Questionnaire (revised per Amendment 01)
FDA	United States Food and Drug Administration
FLAIR	fluid attenuating inversion recovery
GCP	Good Clinical Practice
GDS	Geriatric Depression Scale
GRE	Gradient Recalled Echo
Hb1Ac	hemoglobin 1Ac
IA	interim analysis
IAG	Imaging Charter and Imaging Acquisition Guidelines
IC_{50}	concentration that produces 50% inhibition
ICF	informed consent form
ICH	International Conference on Harmonisation
IEC	Independent Ethics Committee
IMC	Interim Monitoring Committee
INR	international normalized ratio
IP/LC-MS/MS	Immunoprecipitation liquid chromatography-tandem mass spectrometry
IRB	Institutional Review Board
IV	intravenous
IVRS	Interactive Voice Response System
LC-MS/MS	liquid chromatography – tandem mass spectrometry
LLN	lower limit of normal
LNH	low, normal, high compared to laboratory reference range
LP	lumbar puncture
mAb158	monoclonal antibody 158 (a code designation)
MAD	multiple ascending dose
MCI	mild cognitive impairment
MedDRA	Medical Dictionary for Regulatory Activities
MMRM	mixed-effects model with repeated measures
MMSE	Mini Mental State Examination
MRI	magnetic resonance imaging
NIA-AA	National Institute of Aging-Alzheimer's Association
NCI-CTCAE	National Cancer Institute - Common Terminology Criteria for Adverse Events

Abbreviation	Term
NOAEL	no observed adverse effect level
OLE	open-label Extension
PD	pharmacodynamic
PET	positron emission tomography
PG	pharmacogenomic
PI	principal investigator
PiB	Pittsburgh compound B
PK	pharmacokinetic
PLS	Partial Least Squares
PO	by the oral route
PRN	as needed
p-tau	phospho-tau
RAR	response adaptive randomization
SAD	single ascending dose
SAE	serious adverse event
SOC	system organ class
SOP	standard operating procedure
SUVR	standard uptake value ratio
SS	steady state
SUSAR	suspected unexpected serious adverse reaction
$t_{1/2}$	terminal elimination half-life
$t_{1/2,ss}$	terminal elimination half-life at steady state
T3	triiodothyronine
T4	thyroxine
TEAE	treatment-emergent adverse event
TEMAV	treatment-emergent markedly abnormal laboratory value
TSH	thyroid stimulating hormone
t-tau	total tau
ULN	upper limit of normal
vMRI	volumetric magnetic resonance imaging
VHP	Voluntary Harmonisation Procedure
WMS-IV LM I	Wechsler Memory Scale IV -Logical Memory (subscale) I
WMS-IV LM II	Wechsler Memory Scale IV -Logical Memory (subscale) II

5 ETHICS

5.1 INSTITUTIONAL REVIEW BOARDS/INDEPENDENT ETHICS COMMITTEES

The protocol, informed consent form (ICF), and appropriate related documents must be reviewed and approved by an Institutional Review Board (IRB) or Independent Ethics Committee (IEC) constituted and functioning in accordance with International Conference on Harmonisation (ICH) E6 Good Clinical Practice (GCP), Section 3, and any local regulations. Any protocol amendment or revision to the ICF will be resubmitted to the IRB/IEC for review and approval, except for changes involving only logistical or administrative aspects of the study (eg, change in Clinical Research Associate[s] [CRA], change of telephone number[s]). Documentation of IRB/IEC compliance with the ICH E6 and any local regulations regarding constitution and review conduct will be provided to the sponsor.

A signed letter of study approval from the IRB/IEC chairman must be sent to the principal investigator (PI) with a copy to the sponsor before study start and the release of any study drug to the site by the sponsor or its designee (ICH E6, Section 4.4). If the IRB/IEC decides to suspend or terminate the study, the investigator will immediately send the notice of study suspension or termination by the IRB/IEC to the sponsor.

Study progress is to be reported to IRB/IECs annually (or as required) by the investigator or sponsor, depending on local regulatory obligations. If the investigator is required to report to the IRB/IEC, he/she will forward a copy to the sponsor at the time of each periodic report. The investigator(s) or the sponsor will submit, depending on local regulations, periodic reports and inform the IRB/IEC of any reportable adverse events (AEs) per ICH guidelines and local IRB/IEC standards of practice. Upon completion of the study, the investigator will provide the IRB/IEC with a brief report of the outcome of the study, if required.

At the end of the study, the sponsor should notify the IRB/IEC and Competent Authority within 90 days. The end of the study will be the date of the last study visit for the last subject in the study. The sponsor should also provide the IRB/IEC with a summary of the study’s outcome.

In the case of early termination/temporary halt of the study, the investigator should notify the IRB/IEC and regulatory authorities within 15 calendar days, and a detailed written explanation of the reasons for the termination/halt should be given.

5.2 ETHICAL CONDUCT OF THE STUDY

This study will be conducted in accordance with standard operating procedures (SOPs) of the sponsor (or designee), which are designed to ensure adherence to GCP guidelines as required by the Committee for Proprietary Medicinal Products (CPMP) following:

Principles of the World Medical Association Declaration of Helsinki, 2008 version

ICH E6 Guideline for GCP (CPMP/ICH/135/95) of the European Agency for the Evaluation of Medicinal Products, Committee for Proprietary Medicinal Products (CPMP), International Conference on Harmonisation of Pharmaceuticals for Human Use (ICH)

- Title 21 of the United States Code of Federal Regulations (US 21 CFR) regarding clinical studies, including Part 50 and Part 56 concerning informed subject consent and IRB regulations and applicable sections of US 21 CFR Part 312

European Good Clinical Practice Directive 2005/28/EC and Clinical Trial Directive 2001/20/EC for studies conducted within any European Union (EU) country. All suspected unexpected serious adverse reactions (SUSARs) will be reported, as required, to the Competent Authorities of all involved EU member states.

- Other applicable regulatory authorities' requirements or directives

5.3 SUBJECT INFORMATION AND INFORMED CONSENT

As part of administering the informed consent document to the subject, the investigator must explain to each subject and caregiver/informant the nature of the study, its purpose, the procedures involved, the expected duration, the potential risks and benefits involved, any potential discomfort, potential alternative procedure(s) or course(s) of treatment available to the subject, and the extent of maintaining confidentiality of the subject's records. Each subject must be informed that participation in the study is voluntary, that he/she may withdraw from the study at any time, and that withdrawal of consent will not affect his/her subsequent medical treatment or relationship with the treating physician. This information must also be given to the caregiver/informant.

This informed consent should be given by means of a standard written statement, written in nontechnical language. The subject should understand the statement before signing and dating it and will be given a copy of the signed document. If a subject is unable to read or if the caregiver/informant is unable to read, an impartial witness should be present during the entire informed consent discussion. After the ICF and any other written information to be provided to subjects is read and explained to the subject and caregiver/informant, and after the subject has orally consented to his or her participation in the study and has signed and personally dated the ICF, the witness should sign and personally date the consent form. At the Screening Visit, the subject will be asked to sign an ICF before any study-specific procedures are performed. No subject can enter the study before his/her informed consent has been obtained. In the case of a subject lacking capacity to consent in the investigator's opinion, subject's assent plus written informed consent of a legal representative should be obtained, if required in accordance with local laws, regulations and customs. Capacity to consent and definition of legal representative

should be determined in accordance with applicable local laws and regulations. The Investigator shall re-assess consent capacity at periodic intervals during the subject's involvement in the study, and when a subject's family member expresses concern about the subject's study participation. The method and frequency of re-assessment of capacity is at the investigator's discretion (taking into account any relevant local laws and regulations). Should a subject decline to the point of lacking capacity in the investigator's opinion during the study, the investigator should obtain the assent of the subject and consent of a legal representative for the subject to continue in the study. (revised per Amendment 07)

The caregiver/informant should also consent to supporting the subject's participation in the study and will be provided with a separate written informed consent form for his or her participation in the study, to be signed before he or she participates in any further aspects of the study.

An unsigned copy of an IRB/IEC-approved ICF must be prepared in accordance with ICH E6, Section 4, and all applicable local regulations. Each subject must sign an approved ICF before study participation. The form must be signed and dated by the appropriate parties. The original, signed ICF for each subject will be verified by the sponsor and kept on file according to local procedures at the site.

All subjects must consent to either a baseline positron emission tomography (PET) scan or a baseline cerebrospinal fluid (CSF) sample to be used to determine study eligibility. Subjects will also be asked to sign separate optional ICFs to enter the CSF and imaging substudies, respectively. For those who consent to both CSF and PET collection for eligibility, samples will be collected as follows:

For the Core Study, subjects whose study eligibility is determined by amyloid PET and who have consented to both substudies will have: (revised per Amendment 08)

- Additional PET scans performed at 12 and 18 months
- CSF samples collected at Baseline, 12, and 18 months

For the Core Study, subjects whose study eligibility is determined on CSF and who have consented to both substudies will have: (revised per Amendment 08)

- PET scans performed at Baseline, 12, and 18 months
- Additional CSF samples collected at 12 and 18 months

None of the optional consents are required for study eligibility. (revised per Amendment 06)

For the Extension Phase, subjects will need to reconsent for the longitudinal amyloid PET substudy (subjects located in US and Japan only) before or at Extension Phase Baseline (Extension Screening Visit). (revised per Amendments 08, 09, and 11)

6 INVESTIGATORS AND STUDY PERSONNEL

This study will be conducted by qualified investigators under the sponsorship of Eisai (the sponsor) at approximately 125 investigational site(s) including North America, Europe, and Asia-Pacific. (revised per Amendment 07)

The name and telephone and fax numbers of the medical monitor and other contact personnel at the sponsor and at the contract research organization(s) (CRO[s]) are listed in the Investigator Study File or Regulatory Binder provided to each site.

7 INTRODUCTION

7.1 INDICATION

Alzheimer's disease (AD) is a progressive, neurodegenerative disorder of unknown etiology and the most common form of dementia among older people. In 2006, there were 26.6 million cases of AD in the world (range, 11.4-59.4 million).[1] The Alzheimer's Association has reported that in 2007 there were more than 5 million people in the US living with AD.[2] By the year 2050, the worldwide prevalence of AD is predicted to grow to 106.8 million (range, 47.2-221.2 million), while in the US alone the prevalence is estimated to be 11 to 16 million.[1,2]

The disease is clinically characterized by a global decline of cognitive function that progresses slowly and leaves end-stage patients bedridden. AD is the seventh leading cause of all deaths in the US and the fifth leading cause of death in Americans older than the age of 65 years.[2] AD patients typically survive for only 3 to 10 years after symptom onset, although extremes of 2 and 20 years are known.[3] Mortality due to AD is greatly underestimated because death certificates rarely attribute the cause of death to AD. Co-morbidities, including malnutrition, depression, urinary and fecal incontinence, are often associated with AD.[4] Cerebrovascular disease (including vascular dementia) is present in more than 30% of AD patients.[5] Such serious co-morbidity contributes considerably to the diminished lifespan of AD patients.

AD represents a significant economic burden across industrialized countries with a substantial impact on healthcare systems and the public purse as well as on patients and their families. Without doubt the greatest burden of AD falls on patients and caregivers/informants. They not only suffer from the devastating emotional, social, and physical consequences of this, but also bear a substantial economic burden. In the US alone total payments for 2010 are estimated at $172 billion, including $123 billion for Medicare and Medicaid.[2]

7.1.1 Current Therapeutic Options

At this time, there is no cure for AD and no way of slowing down the progression of this disease. This stems largely from our incomplete knowledge of the true pathophysiology of AD in which abnormalities of amyloid β peptide (Aβ) and tau processing, inflammation, oxidative stress, and vascular risk factors, amongst others are postulated to contribute. Present therapeutic agents for AD consist of symptomatic therapies which include the acetylcholinesterase inhibitors (AChEIs), such as donepezil, and the N-methyl-D-aspartate (NMDA)-receptor antagonist, memantine. These therapies improve the symptoms of AD such as cognitive decline and decline in activities of daily living and behavior, but do not alter the underlying progression of the disease. Thus, there is an urgent unmet medical need for drugs that slow or prevent the progression of the disease.

7.1.2 BAN2401

7.1.2.1 Therapeutic Pathway

Amyloid β (Aβ) peptides exist in a dynamic continuum of conformational states such that species tend to progress from monomeric Aβ, to soluble Aβ assemblies that include a range of low molecular weight oligomers to higher molecular weight protofibrils, and finally to insoluble

fibrils (plaques). Various lines of evidence support the "amyloid hypothesis" that Aβ plays a central role in the pathogenesis of AD, and a number of immunotherapies have been developed to reduce the amount of insoluble Aβ fibrils deposited in the brain. However, the quantity and progressive accumulation of insoluble amyloid plaques does not demonstrate a simple correlation with the clinical course of AD. To that end, Aβ aggregate species have been implicated in mediating early neurotoxic events that alter synaptic function, and increasing evidence suggests that these soluble Aβ aggregates may better correlate with disease severity and may contribute to early cognitive dysfunction, widespread neurodegeneration and dementia in AD. While therapeutic strategies continue to focus on removal of insoluble amyloid plaques, an additional approach to therapy may include reducing the toxic Aβ aggregates that may contribute to the neuronal degeneration characteristic of AD.[6,7,8]

7.1.2.2 Mechanism of Action

BAN2401 is a humanized IgG1 monoclonal antibody that distinguishes itself from other anti-amyloid antibodies in that it demonstrates low affinity for Aβ monomer, while it binds with high selectivity to soluble Aβ aggregate species. Although BAN2401 does interact with Aβ monomers and Aβ fibrils, it demonstrates approximately 1000-fold and 5-fold to 10-fold higher selectivity for soluble Aβ aggregate species as compared with Aβ monomers or insoluble fibrils, respectively. In contrast, bapineuzumab, another humanized monoclonal antibody with an N-terminal epitope, is a high affinity antibody across the entire spectrum of Aβ species, from monomers to soluble aggregates to insoluble fibrils.

Aβ monomers are considered to be largely inert (or possibly physiologically important), while Aβ fibrils are thought to be inert or locally toxic. Therefore, BAN2401 is hypothesized to selectively neutralize and clear the toxic Aβ species, whereas bapineuzumab has high affinity for all species (whether toxic, inert or physiologically important). With the relatively high abundance of Aβ monomers and insoluble fibrils compared to soluble aggregate species in the AD brain, BAN2401 is anticipated to have much greater relative activity against the primary toxic amyloid species implicated in AD pathology.

7.1.2.3 Clinical Experience with BAN2401

BAN2401 has been evaluated in the first-in-human study, BAN2401-A001-101, in subjects who have mild to moderate AD. The study was conducted in 2 parts: Part A was a single ascending dose (SAD) and Part B was a multiple ascending dose (MAD). Both parts were designed as randomized, double-blind, placebo-controlled, sequential ascending dose schemes. The BAN2401-A001-101 study was designed to allow the MAD part to be initiated while the SAD part was ongoing.

A total of 80 subjects were treated in the study. Specifically, 48 subjects have been dosed in 6 SAD cohorts, with 8 subjects (6 on active treatment and 2 on placebo) treated in each of the cohorts, ie, SAD1 at 0.1 mg/kg, SAD2 at 0.3 mg/kg, SAD3 at 1.0 mg/kg, SAD4 at 3.0 mg/kg, SAD5 at 10 mg/kg and SAD6 at 15 mg/kg. Similarly, a total of 32 subjects have been treated in each of the 4 MAD cohorts with 8 subjects (6 on active treatment and 2 on placebo) in each of

the cohorts. The MAD1, MAD2, and MAD3 cohorts each received 4 infusions of BAN2401 at 28-day intervals, and the MAD4 cohort received 7 infusions at 14-day intervals. Doses administered at each infusion were: MAD1, 0.3 mg/kg; MAD2, 1.0 mg/kg; MAD3, 3.0 mg/kg; MAD4, 10 mg/kg. (revised per Amendment 11)

Pharmacokinetic and Pharmacodynamic Summary

For all 6 SAD cohorts, noncompartmental analysis was used to estimate pharmacokinetic (PK) parameters. The median tmax occurred at approximately 1.75 to 2 hours from start of infusion. The mean maximum observed concentration (Cmax) and the area under the concentration versus time curve (AUC) values for BAN2401 increased approximately proportionally with BAN2401 dose across the range of 0.3 to 15 mg/kg for SAD cohorts. The half-life of BAN2401 ranged from approximately 85.2 to 163 hours (3.5 to 6.8 days) at 3 to 15 mg/kg.

In the MAD cohorts, the mean C_{max} and AUC values for the first dose of BAN2401 were each consistent with corresponding doses in the SAD cohorts. The mean Cmax and AUC of the first and last doses of BAN2401 in the MAD cohorts increased approximately dose proportionally across the range of 0.3 to 10 mg/kg. The mean half-life of BAN2401 of ranged from approximately 105 to 133 hours (4.4 to 5.5 days), which was consistent with that found in the SAD cohorts. The mean half-life of BAN2401 after the final dose (Dose 7) in MAD4 (10 mg/kg biweekly) was 120 hours (5 days), which was consistent with the half-life in the first dose in MAD cohorts and in the SAD cohorts. Consistent with the half-life in relation to frequency of dosing, minimal accumulation of BAN2401 was measured in these studies. Steady state (SS) was generally achieved after approximately 6 weeks of treatment. The minimum observed concentration (C_{min}) at steady state was approximately 40 μg/mL and the accumulation factor calculated was 1.39. (revised per Amendment 11)

Serum concentration data following BAN2401 single ascending doses of 1, 3, 10, and 15 mg/kg BAN2401 were modeled using a 2-compartment model. The final population PK model predicted that the serum half-life of BAN2401 would be 166.3 hours (6.9 days).

Subjects had CSF samples collected by lumbar puncture (LP) at baseline and 24 hours after the final dose (Dose 4) of study drug (Day 85) for MAD1-MAD3 cohorts. Across these 3 cohorts, the CSF:serum concentration of BAN2401 at 24 hours post Dose 4 was 0.05 to 0.1%, which is consistent with SAD data and predictions from preclinical models. In MAD4 (10 mg/kg every 2 weeks), all 8 subjects had CSF sampling at baseline and postdose. The first 4 subjects who were randomized had CSF sampling at 24 hours post Dose 7 (Day 85), whereas the remaining subjects had CSF sampling at 14 days post Dose 7 (Day 98). CSF concentrations were quantifiable from all 6 subjects treated with BAN2401, 3 each on Day 85 and Day 98. The CSF concentrations of BAN2401 at 24 hours after the final dose are shown in Table 1 with the corresponding CSF:serum concentration ratios.

Table 1 Mean (SD) of CSF and Serum Concentrations and CSF:Serum Ratios in Cohorts SAD6 (15 mg/kg) at 24 Hours Postdose, MAD1 (0.3 mg/kg), MAD2 (1 mg/kg), and MAD 3 (3 mg/kg) at 24 Hours Post-Dose 4, and MAD4 (10 mg/kg) at 24 Hours and 14 Days Post-Dose 7

	Day 2[a] or Day 85[b] (24 h postdose of final dose)			Day 10[a] or Day 98[c] (10[a] or 14[c] days postdose of final dose)		
Cohort /Dose	CSF (ng/mL)	Serum (μg/mL)	CSF:Serum Ratio	CSF (ng/mL)	Serum (μg/mL)	CSF:Serum Ratio
SAD6, 15 mg/kg	96.3 (45.1)	246 (65.8)	0.04% (0.03%)	72.2, 624[d]	100.7, 76.8[d]	0.07%, 0.81%[d]
MAD1, 0.3 mg/kg	4.34 (1.02)	4.49 (1.01)	0.09% (0.02%)	CSF not collected		
MAD2, 1 mg/kg	8.89 (5.31)	17.62 (8.85)	0.04% (0.03%)			
MAD3, 3 mg/kg	25.0 (13.3)	44.1 (4.55)	0.06% (0.04%)			
MAD4, 10 mg/kg	263 (106)	155 (98.1)	0.17% (0.12%)	115.6 (109)	37.5 (21.8)	0.31% (0.50%)

CSF = cerebrospinal fluid, MAD = multiple ascending dose, SAD = single ascending dose

a: SAD6: CSF was collected on Day 2 (24 h postdose) and Day 10 postdose.

b: MAD1-4: CSF was collected on Day 85 (24 h postdose of final dose).

c: MAD4: CSF was collected on Day 98 (14 days postdose of final dose, which was Dose 7).

d: Only 2 subjects treated with BAN2401 at 15 mg/kg had CSF sampling on Day 10 instead of the expected 3 subjects, because 1 of these subjects had CSF sampled on Day 2 in error instead of Day 10. Of the 2 subjects with CSF concentration on Day 10, 1 was found to have CSF concentration ~624 ng/mL (CSF:serum ratio ~0.81%), which was an outlier compared to the CSF:serum ratio of all other subjects who had CSF sampling for BAN2401 concentration. Thus for Day 10 CSF concentration the mean is not provided and only the 2 subjects' individual PK data are shown.

Population PK modeling using the CSF PK data from SAD6 and MAD1 – MAD4 cohorts was also conducted. Preliminary results from the population PK modeling suggested that in CSF, BAN2401 had a $t_{1/2}$ of 167.5 hours (7.0 days), which was similar to that in serum. The t_{max} in CSF was estimated to be 145 hours (6.0 days) postdose, which was slower than in serum, reflecting the slow penetration of BAN2401 through the blood brain barrier. These findings on $t_{1/2}$ and t_{max} in CSF based on the modeling were consistent with observed CSF:serum concentration ratios (Table 1).

In MAD1 to MAD4 cohorts, subjects had CSF collected at baseline and after the final dose of study drug for PD biomarkers. CSF PD biomarkers assayed included Aβ (1-42), total tau (t-tau), and phospho-tau (p-tau). Preliminary analysis of the CSF PD data showed no treatment differences in subjects on BAN2401 compared to placebo. There was no validated assay in CSF of AD subjects for aggregated forms of Aβ. Thus, it was not possible to evaluate the PD effects of BAN2401 on CSF Aβ aggregated forms.

Clinical Safety Summary

In summary, safety and tolerability data from Study BAN2401-A001-101 indicate that BAN2401 is a safe and well tolerated agent at single doses of up to 15 mg/kg IV and at multiple doses of up to 10 mg/kg IV biweekly. In particular, no safety concerns related to adverse central nervous system (CNS) effects, such as amyloid-related imaging abnormalities (ARIA), including

vasogenic edema, micro- and macrohemorrhages and superficial hemosiderosis, have been identified in the BAN2401-A001-101 study. Additionally, there have been no cases of classical acute or delayed hypersensitivity reactions or clinically significant inflammatory reactions of any kind. Although several cases of possible mild infusion reactions have been documented, all of these have been transient, not associated with increasing dose levels or treatment duration and have resolved spontaneous without any safety concerns. While mild and transient CRP elevations have been noted across the range of the SAD and the MAD doses, these largely represent isolated laboratory abnormalities, not associated with fibrinogen elevations, not accompanied by clinical symptoms and, overall, not representing any safety concerns. Similarly, positive anti-drug antibody (ADA) responses identified in the study have been largely asymptomatic and are not associated with any safety concerns. (revised per Amendment 11)

Emerging data from the Study 201 indicate that positive apolipoprotein ϵ4 variant (*APOE4*) (*APOE4* hetero- or homozygous individuals have a higher risk of developing symptomatic ARIA-E. (revised per Amendment 05) At the time of Amendment 04, there were 9 subjects with confirmed cases of ARIA-E, of which 3 cases were symptomatic and associated with brain MRI scans showing significant amounts of vasogenic edema consistent with ARIA-E. All 3 of these symptomatic ARIA-E cases took place in *APOE4* homozygous subjects. The study independent DSMB, having reviewed all unblinded data, recommended not to randomize *APOE4* homozygous individuals to the highest dose of 10 mg/kg biweekly. (revised per Amendment 04) At the time of Amendment 05, there were 10 subjects with confirmed cases of ARIA-E, of which 9 cases were in *APOE4* positive subjects (*APOE4* hetero- or homozygous). The EU Member States have provided a number of safety recommendations based on these data via the Voluntary Harmonisation Procedure (VHP) and expanded upon those provided by the independent DSMB. Specifically, the VHP committee requested that subjects who are confirmed *APOE4* positive (*APOE4* hetero- or homozygous) not be randomized to the 10 mg/kg, biweekly dose. The sponsor has agreed to this request. To permit ongoing assessment of risk factors that may be associated with ARIA-E, all subjects who underwent early termination due to ARIA were unblinded by the sponsor. (revised per Amendments 05, 06, and 11)

At the time of Amendment 11, the Core Study was completed. Preliminary data analysis indicated that the incidence of amyloid related imaging abnormality edema/effusion (ARIA-E) was less than 10% for the highest BAN2401 dose groups (10 mg/kg biweekly and monthly) and less than 8% overall for subjects receiving treatment with any dose level of BAN2401. There were 5 cases of symptomatic ARIA-E in BAN2401 treatment groups (all developed ARIA-E within the first 12 weeks of treatment): 1 severe ARIA-E on magnetic resonance imaging (MRI) at 2.5 mg/kg biweekly, reported as a non-serious AE; 1 mild ARIA-E on MRI at 10 mg/kg monthly, reported as an serious adverse event (SAE); 1 moderate ARIA-E on MRI at 10 mg/kg biweekly, reported as an SAE; and 2 severe ARIA-E on MRI at 10 mg/kg biweekly, both reported as SAEs. The breakdown of ARIA-E cases by ApoE4 status is provided in Table 2, with the incidence being higher in ApoE4 carriers (<15% at top dose) than non-carriers (8% at top dose). (revised per Amendment 11)

Table 2 Summary of Treatment-Emergent ARIA-E by ApoE4 Status (Safety Analysis Set) (revised per Amendment 11)

Category	Placebo (N=245) n (%)	BAN2401 2.5 mg/kg biweekly (N=52) n (%)	5 mg/kg monthly (N=51) n (%)	5 mg/kg biweekly (N=92) n (%)	10 mg/kg monthly (N=253) n (%)	10 mg/kg biweekly (N=161) n (%)	Total BAN2401 (N=609) n (%)
ARIA-E	2 (0.8)	1 (1.9)	1 (2.0)	3 (3.3)	25 (9.9)	16 (9.9)	46 (7.6)
APOE4 positive	2/174 (1.1)	1/38 (2.6)	1/40 (2.5)	3/84 (3.6)	23[a]/225 (10.2)	7/49 (14.3)	35/436 (8.0)
APOE4 negative	0/71	0/14	0/11	0/8	2/28 (7.1)	9/112 (8.0)	11/173 (6.4)

APOE4 = apolipoprotein ϵ4 variant, ARIA-E = amyloid related imaging abnormality edema/effusion, TEAE = treatment-emergent adverse event

a: One additional ApoE4 positive ARIA-E case was observed after 18 month treatment database lock and after treatment discontinuation; therefore, by definition was not treatment-emergent and is not counted in this table or TEAE description above

After 18 months of treatment in any BAN2401 dose group overall, there were few reported cases of ARIA-E (n=46/609, 7.6%). Most cases of ARIA-E (approximately 61%) occurred within the first 3 months of treatment, were mild to moderate in radiographic severity on MRI, were asymptomatic (92%), and typically resolved within 4 to 12 weeks. (revised per Amendment 11)

7.2 STUDY RATIONALE

Various lines of evidence support the “amyloid hypothesis” that Aβ plays a central role in the pathogenesis of AD, and a number of immunotherapies have been developed to reduce the amount of insoluble Aβ fibrils (plaques) deposited in the brain. However, the quantity and progressive accumulation of insoluble amyloid plaques does not demonstrate a simple correlation with the clinical course of AD. This shortcoming makes it difficult to identify a direct link between amyloid load and clinical symptomatology. Moreover, the ability to demonstrate an effect on slowing disease course is hampered by the lack of sensitive tools for detecting relatively subtle change in progression or treatment effect in early disease, highlighting the need for long study durations to ensure adequate separation from placebo. These impediments have resulted in the use of biomarkers as surrogates for clinical effect in Phase 2 in order to facilitate the development process for disease modifying agents. However, subsequent Phase 3 AD studies based solely on biomarker findings in Phase 2 have failed to show clinical effect, due in part, to the fact that biomarkers may not predict clinical outcomes for potential disease modifying agents.[9,10] These findings underscore the need for innovative approaches to Phase 2 AD clinical study design to mitigate the risks of embarking on larger and longer studies and suggest that Phase 2 studies of potential disease modifying agents should be designed to demonstrate clinical efficacy in addition to biomarker effects.

The present Phase 2 study, BAN2401-G000-201, employs the use of a Bayesian response adaptive randomization design with frequent interim analyses to continually update randomization such that more subjects are allocated to efficacious doses based on maturing clinical endpoint data. In addition, there will be frequent Drug Safety Monitoring Board

(DSMB) meetings to review the safety data during the Core Study. (revised per Amendments 08 and 11) This approach allows for ongoing assessment of drug futility or evidence for early success and can mitigate the risks associated with larger and longer trials required to demonstrate clinical efficacy by leading to more efficient project termination or early advancement to a successful Phase 3 program. BAN2401-G000-201 represents the core (potentially supportive) Phase 2 study for the BAN2401 program, and is designed to establish clinical efficacy with a new (more sensitive) clinical tool while exploring the dose response of BAN2401 in subjects with Early Alzheimer's disease (EAD). For the purposes of this protocol, EAD is defined as a continuum of subjects from mild cognitive impairment due to AD – intermediate likelihood to mild Alzheimer's disease dementia who are positive for amyloid pathology in the brain. Results from this study are expected to guide the decision to move into Phase 3 development and the selection of the dosing regimen to be taken into Phase 3 registration studies.

7.2.1 Clinical Assessment Rationale

7.2.1.1 Rationale for Conducting the Study in MCI due to AD – Intermediate Likelihood and Mild Alzheimer's Disease Dementia Subjects

There is a growing consensus within the Alzheimer's disease field that it may be necessary to initiate disease modifying treatments early in the disease course, before the onset of clinical dementia.[11] During the past 2 decades there have been a number of efforts to test AD treatments in early symptomatic patients suffering from mild cognitive impairment (MCI) without dementia. These trials have been largely negative, due in part to operational difficulties with diagnosis of the MCI population. New MCI diagnostic criteria have recently been offered by the National Institute on Aging – Alzheimer's Association (NIA-AA)[11] to address these difficulties by recommending the use of the term "MCI due to AD" to refer to the symptomatic predementia phase of AD where primary AD pathology has been confirmed. Moreover, it has recently been suggested that clinical trials targeting the early symptomatic AD population "abandon the distinction between MCI and dementia in describing the study population and endpoints" and "expand the diagnosis of AD to include the subset of subjects with MCI who have AD neuropathology and are likely to progress to AD dementia."[12] Thus, the field is moving toward a paradigm where clinical studies in early AD incorporate the use of AD pathology biomarkers to define a population consisting of predementia subjects with MCI due to AD and mild Alzheimer's disease dementia subjects.

BAN2401 represents a putative therapeutic agent that is hypothesized to selectively target soluble Aβ aggregate species thought to produce synaptic toxicity, and this antibody has been shown to be capable of clearing diffuse amyloid plaques, suggesting that BAN2401 has the potential to neutralize the soluble Aβ aggregate species that elicit synaptotoxic events and to alter plaque formation in the brain. Thus, it is hypothesized to be effective at early stages of the disease when amyloid has been deposited in the brain, but where the downstream neurodegenerative cascade thought to be triggered by the amyloid deposition is relatively early in its course. Treatment with BAN2401 and attempts to slow disease progression should be most fruitful at this early stage where limited brain tissue loss has been produced and associated clinical deficits are at a minimum.

For these reasons, the appropriate target population for this study is considered to be Early AD. We define Early AD as a continuum of AD severity from MCI due to AD – intermediate likelihood to mild Alzheimer's disease dementia. The study will enroll subjects with mild Alzheimer's disease dementia, who have amyloid pathology and have progressed to dementia,[13] but are hypothesized to be sufficiently early in the disease course to benefit from therapeutic effect. In addition, this protocol will enroll subjects with MCI due to AD – intermediate likelihood, identified in accordance with the NIA-AA criteria,[12] ie symptomatic but not demented AD subjects with evidence of brain amyloid pathology making them less heterogeneous and more similar to mild Alzheimer's disease dementia subjects in cognitive and functional decline as measured by the proposed clinical endpoint for this study, the Composite Clinical Score (Section 7.2.1.2; Section 9.5.1.3).

7.2.1.2 Efficacy Assessment – Composite Clinical Score

No well-established, validated clinical endpoints exist that are sensitive to change in MCI populations in clinical studies. In fact, there has been concern that the standard cognitive instrument, the Alzheimer Disease Assessment Scale-Cognitive Scale (ADAS-Cog), may not have sufficient sensitivity to change even in a mild AD population. Demonstration of an effect on slowing clinical progression in an EAD population is hampered by this lack of sensitive tools for measuring relatively subtle longitudinal changes or treatment effect in early disease. Heterogeneity of the MCI population as measured by established cognitive instruments, such as ADAS-Cog, presents another challenge for clinical studies in EAD and points to an acute need for a clinical tool that will be responsive to progression and treatment effects in EAD.

Given these considerations, clinical data from multiple MCI studies were used to develop a new score that would demonstrate maximum responsiveness to progression and to treatment in an MCI population and that would also perform well in a mild Alzheimer's disease dementia population. This score is described in Section 9.5.1.3, and is designated as the Composite Clinical Score.

Most notably, the use of the Alzheimer's Disease Composite Score (ADCOMS) allows for substantial sample size reductions in nonenriched and enriched MCI populations for a 12-month study by increasing sensitivity and reducing heterogeneity (Table 3).

Table 3 Sample Size Requirements for the ADCOMS Across 3 Treatment Effect Sizes Compared to ADAS-Cog and CDR in MCI Subjects and Enriched MCI Subjects

		MCI			MCI CSF Aβ(1-42) Positive		
	**Treatment Effect**	**Derived Clinical Composite Score**	**ADAS-Cog**	**CDR**	**Derived Clinical Composite Score**	**ADAS-Cog**	**CDR**
**Sample Size per Arm[a]**	25%	1442	9490	1910	399	3608	477
	50%	362	2373	479	101	903	120
	75%	162	1056	213	46	402	54
**% Reduction in sample size with the ADCOMS[b]**	25%-75%		85%	25%		89%	16%

Aβ(1-42) = amyloid beta monomer from amino acid 1 to 42, ADAS-Cog = Alzheimer Disease Assessment Scale-Cognitive subscale, ADCOMS = Alzheimer's Disease Composite Score, CDR = Clinical Dementia Rating, CSF = cerebrospinal fluid, MCI = mild cognitive impairment.
a: Sample size was determined using a 2-sided test with 80% power and alpha=0.05.
b: % reduction per arm over 12 months

In addition, the ADCOMS shows comparable sensitivity in the MCI due to AD – intermediate likelihood (CSF Aβ positive) subgroup and in mild Alzheimer’s disease dementia, indicating that subjects with MCI due to AD – intermediate likelihood progress similarly to mild Alzheimer’s disease dementia subjects according to the new score and allowing the use of a single clinical tool across the EAD population targeted in this study.

Summary of Efficacy Assessments

The Core Study was completed at the time of Amendment 11. Preliminary analysis of Core Study efficacy data demonstrated that at 18 months, there was a dose-dependent reduction in amyloid level in subjects treated with BAN2401. Compared with placebo, this reduction was statistically significant across all BAN2401 dose groups ($P<0.0001$). Furthermore, results of PET image visual read showed that there was a dose-dependent conversion from amyloid positive at baseline to amyloid negative at 18 months in subjects treated with BAN 2401, with 81% of subjects in the BAN2401 10 mg/kg biweekly dose group converted from amyloid positive to amyloid negative ($P<0.0001$) at 18 months.

Reduction in amyloid levels was also accompanied by dose-dependent slowing of clinical decline as measured by ADCOMS and ADAS-Cog over 18 months. The BAN2401 10 mg/kg biweekly dose demonstrated a statistically significant reduction on both the ADCOMS (30% reduction compared with placebo; $P=0.034$) and ADAS-Cog (47% reduction compared with placebo; $P=0.017$). A statistically significant reduction in clinical decline on ADCOMS was observed as early as 6 months ($P<0.05$) and at 12 months ($P<0.05$). Consistent with these results, a dose-dependent slowing in cognitive decline from baseline was also demonstrated by

the CDR-SB. At 18 months, subjects treated with BAN2401 10 mg/kg biweekly demonstrated 26% less cognitive decline compared with placebo. (revised per Amendment 11)

7.2.2 Biomarker Rationale

7.2.2.1 Amyloid PET

Amyloid positron emission tomography (PET) imaging will be performed in this study for 2 primary reasons: 1) to confirm the presence of amyloid pathology in the brain of MCI and mild Alzheimer's disease dementia subjects in the screening phase of the study; and 2) to evaluate the effects of BAN2401 on amyloid levels in the brain, both by whole brain analysis (the average of 5-6 cortical regions) and brain region analysis.

A large body of clinical findings and clinical pathology data have shown that amyloid imaging provides a sensitive in vivo approach to detect amyloid plaques across the cortex. Studies with ^{11}C-Pittsburgh Compound B (^{11}C-PiB) show that 20% to 30% of healthy controls, 60% to 70% of MCI subjects, and 80% to 90% of Alzheimer's disease dementia subjects are positive for amyloid.[14,15,16,17] Apolipoprotein ε4 variant (*APOE4*) carriers show significantly greater rates of amyloid retention when compared to noncarriers, supporting the rationale for inclusion of apolipoprotein E (*APOE*) genotype as a key covariate in the analysis of the baseline and longitudinal PET amyloid data.[18] The rationale for performing amyloid imaging in MCI subjects is supported by the fact that amyloid positive MCI subjects are likely to convert to AD within 2 to 3 years. Further, the presence of brain amyloid shows strong correlations with smaller hippocampal volume and worse performance on assessments of episodic memory.[19] Several ^{18}F amyloid tracers, including florbetapir and flutemetamol[20] have been developed with substantially longer half-life compared to ^{11}C (20 minutes). Florbetapir, floretaben and flutemetamol are currently used in the Alzheimer's Disease Neuroimaging Initiative 2 (ADNI 2) and have been approved by the Food and Drug Administration (FDA) and in Europe (revised per Amendments 02 and 07). Florbetapir, flutemetamol, florbetaben, or any approved agent may be used in Canada, the EU, or any other region in which the study is conducted. For Screening purposes, the imaging agent of choice will be florbetapir at study sites in the US, while in other regions the choice of imaging agent will depend on local availability of imaging agent, thus allowing for use of florbetaben or flutemetamol as well as florbetapir. While, florbetapir is the preferred agent in the US, flutemetamol, florbetaben, or any approved agent may be used in the US if needed. For the longitudinal amyloid PET substudy (Core Study), only florbetapir will be used in the US, while in other regions the choice of imaging agent will depend on local availability of the imaging agent, but the same tracer must be used across all PET assessments per subject. Florbetapir will be used in the US and flutemetamol will be used in Japan for longitudinal amyloid PET analysis in qualified subjects who participate in the imaging substudy in the Extension Phase (US and Japan only). (revised per Amendments 02, 06, 07, 11, and 12)

The inclusion of amyloid PET imaging to evaluate treatment effects is based on recent data showing that the murine homolog, monoclonal antibody 158 (mAb158), significantly reduced plaque load in aged Tg2576 mice (approximately 19 months old) treated for 3 months (50 mg/kg, weekly, IP) compared to untreated controls. Amyloid plaque load was reduced in hippocampus

by 12.9% and cortex by 9.5% with a possible preferential effect on diffuse (6E10-positive) versus core plaque. In a separate study conducted in 12.5 month old Tg2576 mice, 18 weeks treatment with 12 and 24 mg/kg mAb158 reduced 6E10-positive plaques.

Recent longitudinal amyloid PET studies show annual increases of 1.5%, 2.1%, and 5.7% in the healthy control, MCI and AD groups, respectively until the late stages of AD when levels tend to plateau or decline.[7] Such changes have shown to only weakly correlate with declines in cognitive functioning. In light of the above findings, amyloid PET will be conducted at 12 and 18 months of treatment in a subgroup of subjects from any participating country (revised per Amendments 02 and 07) (imaging subgroup) to demonstrate target engagement and to assess amyloid clearance as a potential mechanism of action for BAN2401.

7.2.2.2 Volumetric MRI

Volumetric magnetic resonance imaging (vMRI) will be used to evaluate the effects of BAN2401 on rates of atrophy in the EAD study population across the different treatment arms and to provide evidence that effective treatment is associated with disease modification. A large body of data has demonstrated that structural MRI reliably tracks the course of the disease. Atrophy tends to occur preferentially in the lateral temporal and posterior parietal regions during the early stages of disease (early AD) and progresses towards the anterior regions of the brain (frontal cortex) in its later stages. For this reason, hippocampal volume has been selected as the secondary endpoint and it is predicted that clinically efficacious doses will be associated with a slowing in the rate of hippocampal atrophy compared to rates observed in the placebo and nonefficacious treatment arms. Whole brain and ventricular volumes will be considered exploratory endpoints.

Annualized rates of change of 2%-3% have been reported for the AD brain compared to 1% in MCI subjects and <1% in age-matched controls. However, subjects with MCI due to AD – intermediate likelihood (CSF Aβ <192 or positive amyloid imaging) show rates of atrophy comparable to those observed in Alzheimer's disease dementia subjects,[21,22,23,24] supporting the notion that such subjects are on similar trajectories. Lower CSF Aβ(1-42), amyloid positivity on PET imaging, and higher CSF tau in addition to the presence of the *APOE4* allele have been associated with greater rates of brain atrophy and will be entered as covariates in various proposed analyses.[25,26] Hippocampal atrophy has been shown to be a significant predictor of clinical progression, independent of amyloid levels in the brain.[27] Brain volume (total ventricular volume, whole brain, temporal lobe) generally shows a modest correlation with performance on a number of cognitive scales.[21,22,23,28] These relationships and the effects of treatment will be further explored. Although it is hypothesized that treatment will be associated with declines in rates of atrophy, worsening atrophy has been described following immunotherapy treatment. Reasons for this paradoxical effect remain unclear but may be possibly related to osmotic shifts or the effects of inflammatory factors produced by activated microglia in response to treatment. To address this issue, baseline brain volumes will be entered in the analysis of volumetric change and consideration will be given to undertaking comprehensive regional analysis, including cortical thickness maps, which may be less susceptible to such effects.

vMRI sequences will be collected at all study visits at which safety MRIs are conducted except for the Visit 6 abbreviated MRI assessment (safety MRI to detect ARIA lasting 8 to 10 minutes, with no vMRI sequences) conducted only at European sites (revised per Amendment 07). vMRI analysis will be conducted at the Screening Visit and Visits 16, 29, and 42 (6, 12, and 18 months of treatment). Recent advances in analysis software have made structural imaging more sensitive to detect small changes and able to reliably measure rates of atrophy at 6-month intervals, particularly in the posterior parietal and the lateral temporal regions during the early stages of disease.[22] Given these observations, a demonstration of significant slowing or stabilization in the rate of atrophy in response to treatment compared to placebo will offer further support for disease modification and will therefore represent a key element in the drug development strategy for BAN2401.

7.2.2.3 Soluble Biomarkers in Cerebrospinal Fluid (CSF)

BAN2401 is a low-affinity Aβ(1-42) monomer antibody that targets soluble Aβ aggregates and has been shown to clear Aβ plaques in preclinical studies. Since BAN2401 targets soluble aggregate Aβ species, it is possible that BAN2401 may alter the dynamics of Aβ aggregation and result in changes in total Aβ(1-42) monomer levels. Moreover, it is postulated that clearance of Aβ plaque (and possibly aggregates) may lead to changes in total Aβ monomer levels as a byproduct of the fibril degradation process. For these reasons, changes in Aβ(1-42) levels will be explored in CSF and may provide a central pharmacological readout for target engagement of BAN2401.

Total tau and p-tau are sensitive biomarkers of neurodegeneration. It has been demonstrated that they increase in parallel to progression of Alzheimer's disease. Cerebrospinal fluid (CSF) tau measurements have been correlated with cerebral atrophy. The ratios of p-tau and t-tau to Aβ(1-42) levels have provided useful predictors of cognitive decline and conversion to AD.[28] Recent work from a Phase 2 mild-to-moderate AD study using bapineuzimab suggests that decreases in p-tau may be a downstream effect of altering amyloid load using Aβ-targeted immunotherapeutics.[29] Therefore, monitoring CSF levels of p-tau and t-tau may provide supportive evidence of disease modification by BAN2401.

Baseline levels of Aβ(1-42), t-tau, and p-tau will also help elucidate the extent of amyloid and tau pathology at entry. Considered together with baseline levels of Aβ and baseline imaging variables, these soluble biomarkers may correlate with differences in treatment response.

8 STUDY OBJECTIVES

CORE STUDY

Primary Objectives:

1. To evaluate the efficacy of BAN2401 compared to placebo by establishing the ED_{90} (as defined in the protocol) for BAN2401 on the ADCOMS at 12 months of treatment in subjects with Early Alzheimer's Disease (EAD), defined as mild cognitive impairment (MCI) due to Alzheimer's disease (AD) – intermediate likelihood or mild Alzheimer's disease dementia
2. To assess the safety and tolerability of 3 doses and 2 dose regimens of BAN2401 in subjects with EAD

Key Secondary Objectives: (revised per Amendments 09 and 10)

1. To evaluate the effects of BAN2401 compared to placebo on brain amyloid pathophysiology at 18 months of treatment in subjects with EAD as measured by amyloid PET (revised per Amendments 09 and 10)
2. To evaluate the efficacy of BAN2401 compared to placebo on the ADCOMS at 18 months of treatment in subjects with EAD (revised per Amendments 09 and 10)
3. To evaluate the efficacy of BAN2401 compared to placebo on the Clinical Dementia Rating – Sum of Boxes (CDR-SB) at 18 months of treatment in subjects with EAD (revised per Amendment 10)
4. To evaluate the efficacy of BAN2401 compared to placebo on ADAS-Cog in subjects with EAD at 18 months (revised per Amendment 10)
5. To evaluate the efficacy of BAN2401 compared to placebo at 18 months on clinical status separately within subjects with MCI and mAD dementia for the following assessments: ADCOMS, CDR-SB, and ADAS-Cog (revised per Amendment 10)
6. To evaluate the effects of BAN2401 compared to placebo on CSF biomarkers (Aβ[1-42], t-tau, and p-tau) at 18 months of treatment in subjects with EAD (revised per Amendment 10)
7. To evaluate the effects of BAN2401 compared to placebo on total hippocampal volume using volumetric magnetic resonance imaging (vMRI) at 18 months of treatment in subjects with EAD (revised per Amendments 09 and 10)

Secondary Objectives:

1. To evaluate the effects of BAN2401 compared to placebo on brain amyloid pathophysiology at 12 months of treatment in subjects with EAD as measured by amyloid PET (revised per Amendments 09 and 10)
2. To evaluate the effects of BAN2401 compared to placebo at 12 months on clinical status in subjects with EAD for the following assessments: ADCOMS, CDR-SB, and ADAS-cog (revised per Amendments 09 and 10)

3. To evaluate the effects of BAN2401 compared to placebo at 12 months on clinical status separately within subjects with MCI and mild AD dementia for the following assessments: ADCOMS, CDR-SB, and ADAS-Cog (revised per Amendment 10)
4. To evaluate the efficacy of BAN2401 compared to placebo on CSF biomarkers (Aβ[1-42], t-tau, and p-tau) at 12 months of treatment in subjects with EAD (revised per Amendment 10)
5. To evaluate the effects of BAN2401 compared to placebo on total hippocampal atrophy as measured by vMRI at 6 and 12 months, and left and right hippocampus, whole brain and total ventricular volume as measured by vMRI at 6, 12, and 18 months of treatment in subjects with EAD (revised per Amendment 10)

Exploratory Objectives:

1. To evaluate the effects of BAN2401 compared to placebo on clinical status in subjects with EAD by assessment ADCOMS, ADAS-Cog, CDR-SB, MMSE and FAQ at time points not listed in Key Secondary and Secondary objectives (revised per Amendment 10)
2. To explore the relationship between change from baseline in clinical status at 12 and 18 months of treatment and baseline brain amyloid pathophysiology in subjects with EAD as measured by amyloid PET (revised per Amendment 10)
3. To explore the relationship between change from baseline in clinical status and change from baseline in brain amyloid pathophysiology at 12 and 18 months of treatment in subjects with EAD as measured by amyloid PET (revised per Amendment 10)
4. To explore efficacy of BAN2401 compared to placebo and the overall study population on the ADCOMS across all clinical assessment time points in Japanese subjects with EAD (revised per Amendments 07 and 10)
5. To characterize the population pharmacokinetics (PK) of BAN2401 in EAD subjects, and to examine the effect of intrinsic and extrinsic factors on the PK (revised per Amendment 08)

EXTENSION PHASE (REVISED PER AMENDMENTS 08 AND 11)

Primary Objective

To evaluate the long-term safety and tolerability of BAN2401 in subjects with EAD

Secondary Objective

To assess if the treatment benefit in brain amyloid levels (as measured by amyloid PET) at the end of the Core Study will be maintained over time in the Extension Phase in subjects with EAD

Exploratory Objectives

1. To evaluate the clinical effects of BAN2401 in subjects with EAD on the ADCOMS, CDR-SB, ADAS-Cog, and MMSE over time
2. To assess time to disease progression as evaluated by Clinical Dementia Rating (CDR) global score during the Extension Phase
3. To explore the long-term effects of BAN2401 in subjects with EAD on total hippocampal volume and other biomarkers (eg, plasma biomarkers) at 12 and approximately 24 months in the Extension Phase (Visit 70 [Extension Week 53], Visit 95 [Extension Week 103])
4. To characterize population PK of BAN2401 in subjects enrolled in the Extension Phase of the study

9 INVESTIGATIONAL PLAN

9.1 OVERALL STUDY DESIGN AND PLAN

This double-blind, parallel-group, placebo-controlled, multicenter and multinational study will utilize a dose-finding response adaptive randomization (RAR) design to evaluate the safety, tolerability, and efficacy of BAN2401 in subjects who have MCI due to Alzheimer's disease – intermediate likelihood, or who have mild Alzheimer's disease dementia. The study will take place over approximately 130 months at approximately 125 sites including North America, Europe, and Asia-Pacific countries. (revised per Amendment 08) Approximately 3200 subjects will be screened to provide a approximately 800 subjects to receive BAN2401 or matching placebo. Sites in Japan will screen approximately 160 subjects in order to randomize at least 40 Japanese subjects, even if early success is declared or the study achieves 800 subjects randomized. (revised per Amendment 07) An Extension Phase will be implemented to allow for up to 24 months (2 years) of additional treatment. (revised per Amendments 08, 09, and 11)

The present study incorporates a Bayesian RAR design with frequent interim analyses during the Core Study to continually update randomization allocation on the basis of the primary clinical endpoint. (revised per Amendment 01) This approach allows for ongoing assessment of drug futility or evidence for early success and for continued changes in randomization that favor efficacious treatment arms. Thus, the Bayesian approach not only limits exposure of subjects to nonefficacious treatment arms but can also mitigate the risks associated with larger and longer trials required to demonstrate clinical efficacy by leading to more efficient project termination or early advancement to a successful Phase 3 program. In the Core Study, after the 12-month assessments have been completed, treatment will continue to 18 months to follow the time course of any treatment effects observed at 12 months, and to evaluate biomarker and neuroimaging effects that may be consistent with potential disease modification (revised per Amendment 08).

Subjects will be from 2 clinical subgroups, collectively designated as EAD for the purposes of this protocol: (a) MCI due to AD – intermediate likelihood and (b) mild Alzheimer's disease dementia . At study entry, subjects will be stratified according to clinical subgroup, APOE status (*APOE4* positive and negative), and the presence or absence of ongoing AD treatment with AChEIs or memantine or both. (revised per Amendment 01) Randomization into the 2 clinical subgroups (see Inclusion Criteria, Section 9.3.1) is to remain reasonably balanced whereby at least 60% of the total number of subjects will have MCI due to AD – intermediate likelihood clinical subgroup and at least 30% will have mild Alzheimer's disease dementia. *APOE4* status (positive or negative) must be confirmed for all subjects prior to randomization. (revised per Amendment 05) Subjects who are confirmed *APOE4* positive (*APOE4* hetero-or homozygous) will not be randomized to the 10 mg/kg, biweekly dose. *APOE4* negative subjects will be randomized into 1 of 6 treatment groups (Table 4), while *APOE4* positive subjects will be randomized into 1 of 5 treatment groups as explained in Sections 9.4.3. (revised per Amendments 04 and 05). Randomization in the Core Study will initially be fixed to each of these dose groups (4:2:2:2:2:2 ratio, placebo to each of the 5 active treatment arms). (revised per Amendment 08) All study personnel and subjects will be blinded with respect to the dose

regimens. After 196 subjects have been randomized into the study, an IA will be conducted and RAR will guide subsequent randomization into dose groups. Interim analyses and RAR will be repeated after 250 subjects have been randomized and again after each additional 50 subjects until all 800 subjects are randomized, and will then be conducted at 3-month intervals until all subjects complete 12 months of treatment. (revised per Amendment 07) A Bayesian analysis will be conducted at 12 months of treatment to assess for early success, and at 18 months of treatment, regardless of whether early success is achieved at 12 months. (revised per Amendment 09)

Table 4 BAN2401 Dose Regimens – Core Study

Study Arm	BAN2401 Dose (mg/kg)	Infusion frequency
1	Placebo	Biweekly[a]
2	2.5	Biweekly[a]
3	5.0	Biweekly[a]
4	10.0[b]	Biweekly[a]
5	5.0	4-week intervals[c]
6	10.0	4-week intervals[c]

a: Biweekly = 2-week intervals

b: Subjects who are confirmed *APOE4* positive (*APOE4* hetero-or homozygous) will not be randomized to the 10 mg/kg, biweekly dose. (revised per Amendments 04 and 05)

c: Subjects receiving study drug at 4-week intervals will receive placebo at intervening 2-week time points. (revised per Amendment 04)

Oversight of the interim analyses and RAR will be conducted by an independent Interim Monitoring Committee (IMC). The IMC will consist of 3 members who are external to the company with expertise in Bayesian adaptive design in clinical trials.

The interim analyses and RAR will be conducted by an unblinded external independent data analysis group in accordance with the protocol. This independent group will provide the IA outcomes to the IMC at each interim look. The IMC will ensure the integrity of the IA and response adaptive randomization process through review of primary efficacy data. In addition, the IMC will act in an independent advisory capacity to monitor the Bayesian IA outcomes according to the early success and futility boundaries pre-specified in the protocol. The IMC will inform the sponsor if the boundaries for early success or futility have been crossed. If the study continues to randomize subjects after statistical futility boundary confirmed by the IMC is crossed, it will be considered a failed study. The IMC will not be charged with any subject safety issues, as this will be the responsibility of an independent DSMB. (revised per Amendment 01)

A separate unblinded DSMB will be employed at regular intervals to monitor the overall safety of the Core Study and will make recommendations to the Sponsor as appropriate. (revised per Amendments 08 and 11) In addition, the DSMB will meet when a predetermined number of

subjects are randomized to assess whether the option for home infusions is feasible (per DSMB charter, if allowed and conducted according to country and local guidelines; home infusions will not be allowed in Germany. Upon implementation of Amendment 07, newly enrolled subjects will not be offered the option for home infusions. Subjects opting for home infusions before implementation of Amendment 07 will be allowed to continue with home infusions for the duration of their participation in the study. (revised per Amendments 01, 03, and 07) Details regarding DSMB membership and operational characteristics will be provided in the DSMB Charter. The DSMB will be responsible for monitoring the safety of the study only in the Core Study. (revised per Amendment 11)

If the statistical futility boundary as confirmed by the IMC is crossed, the IMC will inform the DSMB. The DSMB will make risk-benefit assessments after reviewing emerging safety and efficacy signals and will provide its recommendations to the sponsor. The sponsor will make the final decision pertaining to study futility after reviewing recommendations by the DSMB. (revised per Amendment 01)

The following estimates are provided:

- The study will end when the last visit for the last subject is concluded.
- The study is planned to begin no later than December 2012 and to end in approximately 2023 or until the drug is commercially available in the country where the subject resides, whichever comes first. (revised per Amendments 07 and 08)
- The maximum estimated period for each subject on study is 50 months. (revised per Amendment 08) (revised per Amendment 11)

Administration of BAN2401 to an individual subject may be stopped on the basis of safety-related findings as outlined in Section 9.3.3.

An overview of the study design is presented in Figure 1.

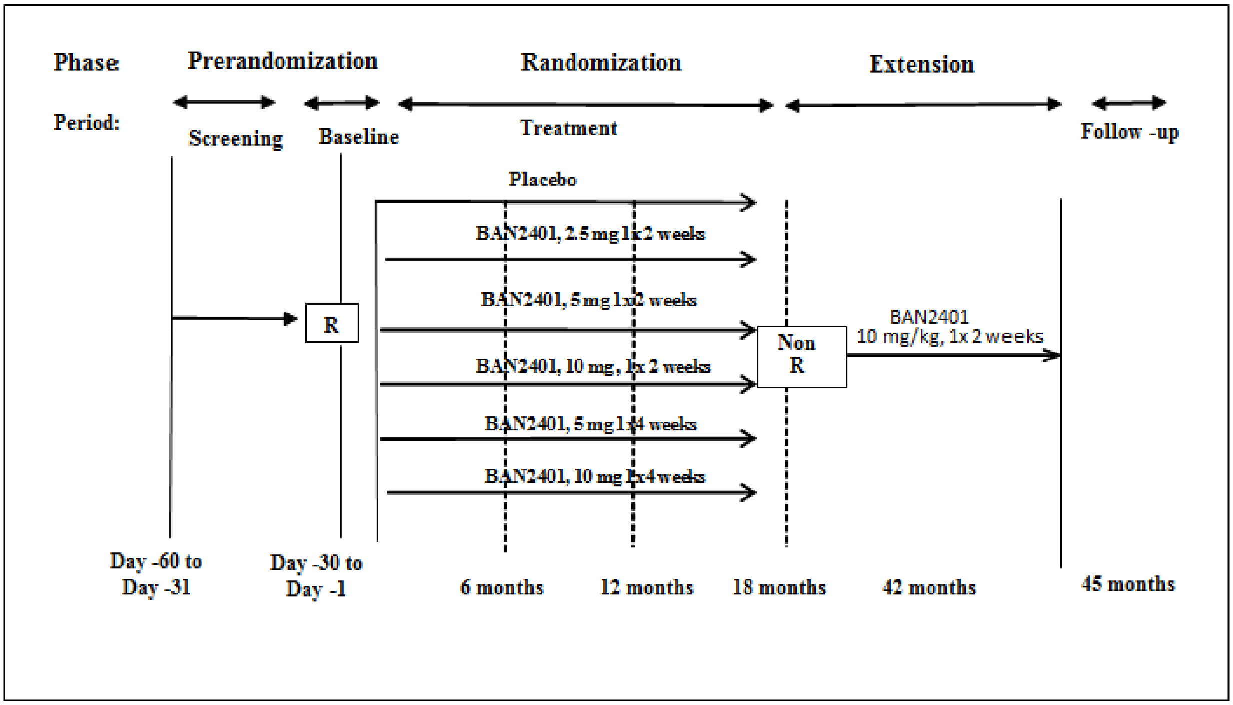

Figure 1 Design of Study BAN2401-G000-201

(revised per Amendments 08 and 11)

Subjects not continuing into the Extension Phase will have a follow-up visit 3 months after the last dose of study drug. However, subjects who previously completed the Core Study (through the Follow-Up Visit, Visit 43) at any time before implementation of the Extension Phase, and/or fulfill the Extension Phase inclusion and exclusion criteria, will also be eligible to participte.

R = randomization.

9.1.1 Prerandomization Phase

The Prerandomization Phase in the Core Study will last for up to 60 days and will include a Screening Period and a Baseline Period. (revised per Amendment 08) The assessments conducted during this phase will include a series of assessments specifically to determine eligibility for randomization into the study. Subjects will be diagnosed according to NIA-AA criteria as either MCI due to AD or mild Alzheimer's disease dementia as defined by the protocol after completion of MMSE, Wechsler Memory Scale IV-Logical Memory (subscale) I (WMS-IV LMI), Wechsler Memory Scale IV-Logical Memory (subscale) II (WMS-IV LMII), and CDR. Once the diagnosis is made, the WMS-IV LMII inclusion criterion can be applied to all subjects. (revised per Amendments 01 and 06) A schematic overview of the process for determining eligibility is shown in Figure 2.

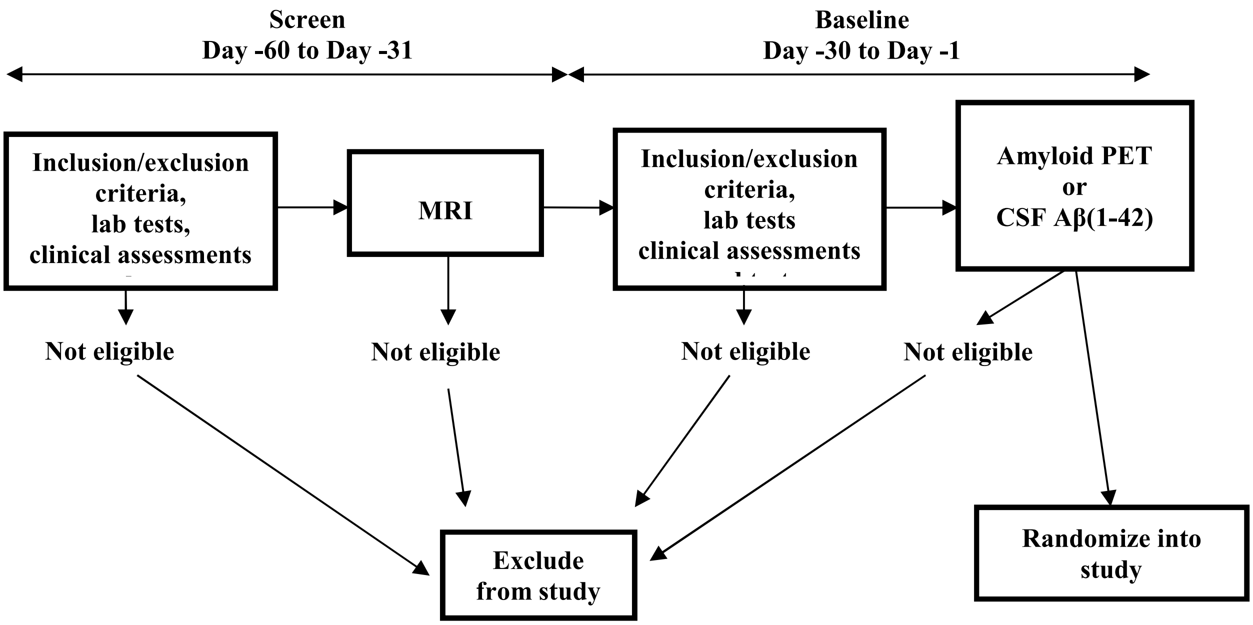

Figure 2 Schematic Overview of Eligibility Assessments

CSF = cerebrospinal fluid, MRI = magnetic resonance imaging, PET = positron emission tomography.
(revised per Amendment 06)

9.1.1.1 Screening Period

The Screening Visit will occur during the Screening Period, between Day -60 and Day -31. Screening assessments and timing thereof are shown in the Schedule of Screening and Baseline Assessments/Procedures (Table 7).

The purpose of the Screening Period is to obtain informed consent to participate in the study and to establish protocol eligibility. Informed consent will be obtained after the study has been fully explained to each subject and caregiver/informant and before the conduct of any screening procedures or assessments. Procedures to be followed when obtaining informed consent are detailed in Section 5.3. No subject will undergo any further study procedures unless this informed consent has been obtained. All subjects must consent to either a baseline PET scan or a baseline CSF sample to be used to determine study eligibility. Subjects will also be asked to sign separate optional ICFs to enter the CSF and imaging substudies, respectively. For those who consent to both CSF and PET collection for eligibility, samples will be collected as follows:

Subjects in the Core Study whose study eligibility is determined by amyloid PET and who have consented to both substudies will have: (revised per Amendment 08)

- Additional PET scans performed at 12 and 18 months
- CSF samples collected at Baseline, 12, and 18 months.

Subjects whose study eligibility is determined on CSF and who have consented to both substudies will have: (revised per Amendment 08)

- PET scans performed at Baseline, 12, and 18 months
- Additional CSF samples collected at 12 and 18 months

None of the optional consents are required for study eligibility. (revised per Amendment 06)

For the Extension Phase, subjects will need to reconsent for the longitudinal amyloid PET substudy before the Extension Screening visit in order to participate. (revised per Amendments 08, 09, and 11)

Consent to blood collection for pharmacogenomic testing is mandatory for eligibility to enter the study. (revised per Amendment 06)

Study site personnel will interview the subject and caregiver/informant to obtain demographic and clinical information as described in Section 9.5.1.1, and will review inclusion and exclusion criteria as described in Sections 9.3.1 and 9.3.2. Prior medications and medications currently being taken by the subject will be reviewed. The Screening Disposition electronic case report form (eCRF) page must be completed to indicate whether the subject is eligible to participate in the study and to provide reasons for screen failure, if applicable.

Subjects who remain eligible for study entry after review of inclusion and exclusion criteria and prior and concomitant medications will then undergo safety assessments. Vital signs will be assessed, height and weight measurements will be taken, and a complete physical examination will be conducted as described in Section 9.5.1.2. Blood will be drawn for a serum pregnancy test in females of childbearing potential only (Section 9.5.1.2), and for clinical laboratory tests in all subjects (Section 9.5.1.2). A urine sample will be collected for drug screening (Section 9.5.1.2). Clinical assessments for eligibility will be conducted during the Screening Visit as described in Section 9.5.1.2. Every effort should be made to conduct these assessments in the morning, but if this is not possible due to the timing of the visit, they should be conducted at the same time of day at all subsequent visits. Testing during the Screening Visit should begin with the clinical assessments in the following order: Mini-mental State Examination (MMSE), Wechsler Memory Scale-IV Logical Memory (subscale) I and II (WMS-IV LMI and LMII), and CDR. The WMS-IV LMII is to be administered 20 to 30 minutes after the completion of LMI. Subject information should be collected in the 20 to 30 minutes between the WMS-IV LM1 and LMII. Subjects who meet criteria for objective memory impairment as determined by the WMS LMII score (See Inclusion Criteria) will also be evaluated on the Geriatric Depression Scale (GDS) for eligibility. Subjects who meet criteria for the GDS will be administered the Columbia Suicide Severity Rating Scale (C-SSRS) as a safety assessment (Section 9.5.1.5), and all remaining Screening Visit assessments will be conducted. The caregiver/informant needs to be present at this visit. (revised per Amendment 01)

Subjects who are eligible following the Screening Visit for eligibility will then undergo non-contrast brain MRI for brain abnormalities that may exclude study participation

(Section 9.5.1.5). The MRI imaging will include fluid attenuating inversion recovery (FLAIR), Gradient Recalled Echo (GRE), T1 and diffusion-weighted sequences to determine the presence of focal lesions that may exclude study participation. An additional scanning sequence will be conducted in all subjects immediately following the safety MRI to obtain vMRI data, as described in Section 9.5.1.3. Results of these MRI assessments will be used as the Baseline Period MRI, against which changes after drug administration will be compared.

9.1.1.2 Baseline Period

Baseline assessments and timing thereof are listed in Table 7 and described in Section 9.5.1.2.

During the Baseline Period, which will extend from Day -30 to Day -1, the subject will return to the study site for additional assessments.

Inclusion/exclusion criteria will again be reviewed, vital signs and weight will be recorded, and a single 12-lead electrocardiogram (ECG) will be conducted. (revised per Amendment 01) A routine physical examination will be conducted and a urine pregnancy test will be conducted for females of childbearing potential. Blood will be collected from all subjects for clinical laboratory tests. The MMSE, the CDR, the ADAS-Cog, and the Functional Assessment Questionnaire (FAQ) will be administered to the subject in the stated order. The caregiver/informant needs to be present at this visit. The C-SRSS will also be administered.

Blood will be collected from all subjects for pharmacogenomic analyses of *APOE4* and blood may be collected for exploratory biomarker analyses as outlined in Appendix 3. (Section 9.5.1.4). (revised per Amendment 01)

Cerebrospinal fluid may be obtained at this visit from subjects who have consented to this procedure, for PK and PD assessments (Section 9.5.1.4, Appendix 3). (revised per Amendment 07)

For further assessment of eligibility, all subjects who pass the preceding screens will then undergo amyloid PET or CSF Aβ(1-42) assessment (revised per Amendment 06) within 20 days after the Baseline Visit, as an eligibility criterion (Section 9.5.1.2, Section 9.5.1.6). Baseline amyloid PET data will serve as the baseline data for amyloid PET results at 12 and 18 months of treatment for those subjects who consent to be included in the imaging subgroup (total target n = 306). The Baseline Visit CSF sample will be used to confirm eligibility and for use as the baseline CSF assessment for those subjects who consent to be included in the CSF substudy. For subjects who screen fail at the Baseline assessment, the Screening Disposition eCRF page must be completed to indicate whether the subject is eligible to participate in the study and to provide reasons for screen failure, if applicable. (revised per Amendments 06 and 07)

Subjects who complete the Baseline Period and meet the criteria for inclusion/exclusion (Section 9.3.1 and Section 9.3.2) will begin the Randomization Phase.

APOE4 status (positive or negative) must be confirmed for all subjects prior to randomization. Subjects who are confirmed *APOE4* positive (*APOE4* hetero- or homozygous) will not be randomized to the 10 mg/kg, biweekly dose. (revised per Amendment 05)

Some subjects who screen fail during the Prerandomization Phase may be eligible for rescreening following sponsor approval. Note that when a subject is rescreened, a new subject number will be issued. All screening and baseline assessments should be repeated *with the following exceptions*:

- **MRI** – No repeat needed if the date of the rescreen is ≤90 days from the date of the original screening MRI.
- **Pharmacogenetics** – If a sample was collected, processed, and *APOE4* results issued during the previous screening procedure, another sample is not required to be collected when a subject is rescreened. Great care must be taken to link the APOE status information with the new subject number.
- **PET/CSF** – No repeat needed for eligibility if confirmed amyloid positive (via CSF or PET) during the original screening procedure. If a subject will participate in the longitudinal PET or CSF substudies, quantitative PET or CSF collection should be repeated if the original PET/ CSF collection was done >6 months from the date of the original screening procedure. (revised per Amendment 07)

The new subject number will be linked to the initial subject number and the outcomes for procedures that need not be repeated as outlined above. (revised per Amendment 06)

9.1.2 Randomization Phase

The Randomization Phase of the Core Study will consist of a Treatment Period of 18 months (78 weeks) duration and a Follow-up Period 3 months after the last dose of study drug. Subjects whose screening assessments and evaluations are completed and reviewed by the PI, or designee (including confirmation of categorization by the 2 clinical subgroups (MCI due to AD – intermediate likelihood or mild Alzheimer's disease dementia), by APOE status (APOE4 positive and negative), and by ongoing treatment with AChEIs or memantine or both) and who continue to meet all of the inclusion/exclusion criteria will enter the Randomization Phase. (revised per Amendment 07) As described in Section 9.4.3, the first 196 subjects will be randomized in a 4:2:2:2:2:2 ratio; Placebo (4) to each of the active treatment arms (2 each). Randomization probabilities to each arm will be updated at each IA such that the randomization probability will be increased for the placebo arm and arms that represent the potential target dose (ED_{90}; the dose that achieves 90% maximum efficacy) and simultaneously decreased for other active arms. Subjects who complete the scheduled visits including the Follow-up Visit will be considered to have completed the study.

9.1.2.1 Treatment Period

Subjects will be randomized at the beginning of the Treatment Period, and will receive study drug by infusion at every visit except the last visit, except for those subjects participating in the Extension Phase, as indicated in the Schedule of Assessments/Procedures (Table 8, and Table 12 for the Extension Phase). (revised per Amendment 08) Double-blinding to study treatments will

be in effect throughout the Core Study. Doses will not be changed. (revised per Amendment 08) If a given subject cannot tolerate a give dose regimen, the subject will be discontinued from the study and will undergo an Early Termination Visit. During the Treatment Period, a subject may develop acute medical conditions unrelated to the study drug which in the opinion of the PI, may make them unsuitable to receive the study drug for 1 or more dose administrations. In this situation, the investigator should exercise clinical judgment as to whether or not to withhold a dose. As a general principle, temporary suspension of dosing may be considered and resumed after the acute medical condition has improved.

The DSMB will review the safety of the study drug after a predetermined number of subjects have been randomized to assess whether the option for home infusions is feasible (per DSMB charter, if allowed and conducted according to country and local guidelines; home infusions will not be allowed in Germany. Upon implementation of Amendment 07, newly enrolled subjects will not be offered the option for home infusions. Subjects opting for home infusions before implementation of Amendment 07 will be allowed to continue with home infusions for the duration of their participation in the study. (revised per Amendments 01, 03, and 07) If the DSMB allows this option of home administrations, site permitting, the investigator has to assess if a subject is suitable for home infusions after the first 4 months of treatment have taken place in the clinic. If a subject has shown acceptable tolerability for the infusions and is considered by the investigator to be at low risk of developing AEs related to study drug infusion which may require acute medical treatment, and has shown no clinically significant findings on other safety measures related to study drug infusion during the first 4 months of their treatment, they may be allowed to receive infusions at home during subsequent study visits that do not otherwise require the subject to be in the clinic.

At all study visits, safety assessments will consist of vital signs (both predose and after study drug infusion) and querying for AEs. Queries for concomitant medications will also take place at study visits, at approximately 6-week intervals. During the first 3 months, study procedures including clinical laboratory tests, routine physical examinations, 12-lead ECG recordings, blood collection for serum anti-BAN2401 assays, concomitant medication information, clinical assessments (MMSE, CDR, ADAS-Cog, and FAQ), and AE queries will be conducted as shown in Table 8. (revised per Amendment 08)

Beginning with Week 13, the following safety assessments will take place at approximately 3-month intervals: weight measurements, routine physical examinations, 12-lead ECG recordings, a urine pregnancy test (females of childbearing potential only), clinical laboratory tests (and an additional time point at 19 weeks), blood samples for anti-BAN2401 assays, and the C-SSRS. Psychometric tests will be administered at these intervals. Blood samples will also be taken for PK assessments at time points specified in Table 8. (revised per Amendment 08) A safety MRI will be conducted at any time following the preceding visit and prior to each of the following visits according to the Schedule of Procedures/Assessments: Visit 6 at European sites only (revised per Amendment 07), Visits 7, 9, 16, 22, 29, 35, and 42 of the Core Study, Early Termination, and at the Follow-up Visit (Visit 43). In all cases, the safety MRI must be reviewed by the imaging vendor and a local reader prior to receiving the next dose of study drug.

(revised per Amendment 06) Details are given in Table 8 for the Core Study and Table 12 for those subjects participating in the Extension Phase. (revised per Amendment 08)

As shown in Table 8 (revised per Amendment 08), vMRI sequences will be collected at all visits where safety MRI is conducted except for the Visit 6 abbreviated MRI assessment (safety MRI to detect ARIA lasting 8 to 10 minutes, with no vMRI sequences) conducted at European sites only (revised per Amendment 07). vMRI data will be analyzed at Screening and at Visits 16, 29, and 42 (6, 12, and 18 months of treatment). In consenting subjects, an amyloid PET scan will be conducted and CSF will be collected at 12 and 18 months in the Core Study. (revised per Amendments 08 and 11)

9.1.2.2 Extension Phase

The Extension Phase will be initiated following the Core Study to allow subjects to receive open-label BAN2401 10 mg/kg biweekly for up to 24 months (2 years), until the drug is commercially available in the country where the subject resides, or until the benefit-to-risk ratio from treatment with BAN2401 is no longer considered favorable, whichever comes first. Any subject who completed study treatment (Visit 42 [Week 79] of the Core Study) and fulfills the Extension Phase inclusion and exclusion criteria (Appendix 4) will have the option to participate. Subjects who previously completed the Core Study (through the Follow-Up Visit, Visit 43) at any time before implementation of the Extension Phase and/or fulfill the Extension Phase inclusion and exclusion criteria (Appendix 4) will also be eligible to participte. (revised per Amendment 11)

All subjects will receive the BAN2401 10 mg/kg biweekly dose, including subjects who are confirmed *APOE4* positive (hetero- or homozygous). The Follow-up Visit in the Extension Phase will take place 3 months after the last dose of study drug. Subjects may discontinue from study drug for any reason. Subjects who discontinue the study drug must comply with the Early Termination Visit (within 7 days after the last dose of study drug) and the Follow Up Visit (3 months after the last dose of study drug).

All subjects who underwent amyloid PET for inclusion in the Core Study should receive a baseline amyloid PET scan before dosing in the Extension Phase. The baseline amyloid PET scan must be conducted with the same imaging tracer that was used for inclusion at the baseline visit for the Core Study. In addition, qualified subjects located in the US and Japan will have the option to participate in the longitudinal PET substudy. In Japan, those who consented to the longitudinal imaging substudy will only undergo amyloid PET at Extension Phase Visit 70 [Extension Week 53], and Visit 96 [Extension Week 105]. Florbetapir will be used in the US and flutemetamol will be used in Japan in this imaging substudy, and only subjects who consent to the longitudinal assessments per the Extension Phase Schedule of Procedures/Assessments (Table 12) will be allowed to participate in this substudy. (revised per Amendments 11 and 12)

For a full description of all extension-related components of the study, see Appendix 4. (revised per Amendment 08)

9.1.2.3 Follow-up Period

Subject who are not eligible or who do not elect to participate in the Extension Phase will be required to continue into the Follow-Up Period in the Core Study. (revised per Amendment 08)

During the Follow-Up Period, a single visit will take place approximately 3 months after the last dose of study drug. At this visit, safety assessments will be conducted including vital signs, a routine physical examination, weight measurements, a single 12-lead ECG, clinical laboratory tests, blood for serum anti-BAN2401, a safety MRI, the C-SSRS, and review of concomitant medications and AEs. (revised per Amendment 01) Clinical tests will be administered consisting of the MMSE, the CDR, the ADAS-Cog, and the FAQ.

Subjects who discontinue the study or study drug early must comply with the Early Termination Visit (within 7 days after the last dose of study drug) and the Follow-up Visit (3-months after the last dose of study drug). Unscheduled visits for safety assessments may also be conducted. Subjects who discontinue due to APOE status are expected to return for an Early Termination Visit and 3-month Follow-Up Visit (ie, 3 months after the last dose of study drug). (revised per Amendment 06). In addition, all subjects who discontinue from study drug are expected to return after the Early Termination Visit for each scheduled visit when clinical assessments of efficacy are to be conducted per the Schedule of Procedures/Assessments (Table 8). (revised per Amendment 08) At these visits, clinical efficacy assessments (MMSE, CDR, ADAS-Cog, and FAQ) will be conducted and information on concomitant medications, AEs, and SAEs will be collected. Regularly scheduled clinical efficacy visits do not need to be attended if they fall within 8 days (visit window) after the Early Termination Visit or within +/-8 days of the 3-month Follow-Up Visit. Subjects who discontinue from study drug are considered on study as long as they return for their regularly scheduled clinical efficacy visits as outlined above. (revised per Amendment 01)

9.1.2.4 Unscheduled Visits

Study assessments that are expected to be performed at unscheduled visits are shown in Table 8. (revised per Amendment 08) The investigator will, however, have the discretion to deviate from this list at these visits and to perform other assessments according to specific visit needs.

9.2 Discussion of Study Design, Including Choice of Control Groups

The primary endpoints will be assessed after 12 months of treatment. Study drug will then continue for an additional 6 months in order to detect effects that are possibly not seen at 12 months and to assess the time course of any observed treatment effects that may be consistent with neuroimaging and biomarker findings.

The present study employs a Bayesian response adaptive randomization design with frequent interim analyses during the Core Study to continually update randomization allocation based on maturing clinical endpoint data from multiple sources. This approach allows for ongoing assessment of drug futility or evidence for early success and can mitigate the risks associated with larger and longer studies required to demonstrate clinical efficacy by leading to more efficient project termination or early advancement to a successful Phase 3 program.

To sample the anticipated dose response as fully as possible on the basis of data from the BAN2401-A001-101 study, subjects will be randomized to placebo and 5 dose regimens comprising 3 different dose levels and 2 different administration frequencies (monthly and

biweekly administration) to fully interrogate the dose response. The doses to be investigated are 10, 5, and 2.5 mg/kg biweekly, and 10 and 5 mg/kg monthly.

Randomization will be used in this study to avoid bias in the assignment of subjects to treatment, to increase the likelihood that known and unknown subject attributes (eg, demographics and baseline characteristics) are balanced across treatment groups, and to ensure the validity of statistical comparisons across treatment groups. For safety reasons, subjects who are confirmed *APOE4* positive (*APOE4* hetero- or homozygous) will not be randomized to the 10 mg/kg, biweekly dose. (revised per Amendments 04 and 05). After each IA, the randomization probability vector will be split between *APOE4* positive and *APOE4* negative strata to ensure no *APOE4* positive subjects are enrolled on the 10 mg/kg biweekly dose. The overall probabilities as suggested by RAR will still be preserved. (revised per Amendments 05 and 07) Blinding to treatment will be used to reduce potential bias during data collection and evaluation of endpoints. A control group is required for assessment of possible treatment effects in this proof-of-concept study.

9.3 Selection of Study Population

The study population will consist of subjects who have EAD. Two subpopulations will be evaluated: those who have MCI due to AD – intermediate likelihood, and those who have mild Alzheimer's disease dementia. Diagnostic criteria will consist of selected cognitive assessments (Section 9.5.1.2), followed by amyloid PET or CSF Aβ(1-42) assessment (revised per Amendment 06) to confirm deposition of amyloid in the brain. So as to ensure as much as possible that treatment effects are not confounded by disorders unrelated to AD, subjects will be evaluated per inclusion and exclusion criteria (Section 9.3.1 and Section 9.3.2). Subjects who do not meet all of the inclusion criteria or who meet any of the exclusion criteria will not be eligible to receive study drug.

9.3.1 Inclusion Criteria

<u>Diagnosis</u>

<u>Mild Cognitive Impairment due to Alzheimer's Disease</u> – Intermediate Likelihood: (revised per Amendment 06)

1. Subjects who meet the NIA-AA core clinical criteria for mild cognitive impairment due to Alzheimer's disease - intermediate likelihood [12]
2. Subjects who have a CDR score of 0.5 and a memory box score of 0.5 or greater at Screening and Baseline
3. Subjects who report a history of subjective memory decline with gradual onset and slow progression over the last 1 year before Screening; **MUST** be corroborated by an informant

<u>Mild Alzheimer's Disease Dementia:</u>

4. Subjects who meet the NIA-AA core clinical criteria for probable Alzheimer's disease dementia[13]

5. Subjects who have a CDR score of 0.5 to 1.0 and a Memory Box score of 0.5 or greater at Screening and Baseline

<u>Key Inclusion Criteria **that must be met by ALL Subjects:**</u>

6. Subjects with objective impairment in episodic memory as indicated by at least 1 standard deviation below age-adjusted mean in the WMS-IV LMII, as follows:
   a) ≤15 for age 50 to 64 years
   b) ≤12 for age 65 to 69 years
   c) ≤11 for age 70 to 74 years
   d) ≤9 for age 75 to 79 years
   e) ≤7 for age 80 to 90 years

   (revised per Amendments 01 and 06)

7. Positive amyloid load as indicated by 1 of the following:
   a. PET assessment of imaging agent uptake into brain
   b. CSF assessment of Aβ(1-42) (revised per Amendment 06)

   Subjects may consent to both the PET and CSF assessments, but need a positive amyloid result in only one of the 2 procedures to confirm eligibility (ie, even if one of the 2 results does not meet its respective eligibility criterion). Subjects who initially consent for only one of the amyloid screening assessments will only be allowed to subsequently consent for the second assessment should the first assessment result be positive or they have not yet been informed of the results of the first assessment. Subjects who consent to Amyloid PET or CSF Amyloid are not required to participate in the respective substudies (revised per Amendments 06 and 07)

   Any historical amyloid positive PET scan may be used for eligibility upon evaluation by the central imaging CRO. For subjects who wish to participate in the longitudinal imaging substudy, historical amyloid PET scans may not be used as baseline scans for the longitudinal imaging substudy. Required details regarding historical images should be submitted to the CRO medical monitor for evaluation. (revised per Amendment 08)

8. Male or female subjects aged between 50 and 90 years, inclusive
9. MMSE score equal to or greater than 22, and equal to or less than 30 at Screening and Baseline, except for the following countries, where MMSE score must be equal to or greater than 22 and equal to or less than 28 at Screening and Baseline: United Kingdom, Spain, Germany, Sweden, France, and the Netherlands (revised per Amendment 03)
10. Body Mass Index (BMI) >17 and <35 at Screening (revised per Amendment 06)
11. Females must not be lactating or pregnant at Screening or Baseline (as documented by a negative beta-human chorionic gonadotropin assay [ß-hCG]). A separate baseline

assessment is required if a negative screening pregnancy test was obtained more than 72 hours before the first dose of study drug.

12. All females will be considered to be of childbearing potential unless they are postmenopausal (amenorrheic for at least 12 consecutive months, in the appropriate age group, and without other known or suspected cause) or have been sterilized surgically (ie, bilateral tubal ligation, total hysterectomy, or bilateral oophorectomy, all with surgery at least 1 month before dosing).
13. Females of childbearing potential must not have had unprotected sexual intercourse within 30 days before study entry and must agree to use a highly effective method of contraception (eg, total abstinence, an intrauterine device, a double-barrier method [such as condom plus diaphragm with spermicide], a contraceptive implant, an oral contraceptive, or have a vasectomized partner with confirmed azoospermia) throughout the entire study period and for 35 days after study drug discontinuation. If currently abstinent, the subject must agree to use a double-barrier method as described above if she becomes sexually active during the study period or for 35 days after study drug discontinuation. Females who are using hormonal contraceptives must have been on a stable dose of the same hormonal contraceptive product for at least 4 weeks before dosing and must continue to use the same contraceptive during the study and for 35 days after study drug discontinuation.
14. Subjects who are receiving AChEIs or memantine or both for AD must be on a stable dose for at least 12 weeks prior to Baseline. Treatment naïve subjects can be entered into the study. Unless otherwise stated, subjects must have been on stable doses of all other permitted concomitant medications (ie, non-AD related) for at least 4 weeks prior to Baseline. Use of memantine will not be allowed for Japanese subjects (revised per Amendments 01 and 07)
15. Must have an identified caregiver/informant (defined as a person able to support the subject for the duration of the study and who spends at least 8 hours per week with the subject). The caregiver/informant must provide separate written informed consent. In addition, this person must be willing and able to provide follow-up information on the subject throughout the course of the study. This person must, in the opinion of the investigator, spend sufficient time with the subject on a regular basis such that the caregiver/informant can reliably fulfill the study requirements. A permanent caregiver/informant need not be living in the same residence with the subject. For such a caregiver/informant not residing with the subject, the investigator has to be satisfied that the subject can contact the caregiver/informant readily during the times when the caregiver/informant is not with the subject. If in doubt about whether a subject's care arrangements are suitable for inclusion, the investigator should discuss this with the medical monitor. Caregivers/informants need only to be present at visits where clinical assessment of CDR and FAQ takes place. (revised per Amendment 01)
16. Provide written informed consent. If a subject lacks capacity to consent in the investigator's opinion, the subject's assent should be obtained, if required in accordance with local laws, regulations and customs, plus the written informed consent of a legal

representative should be obtained (capacity to consent and definition of legal representative should be determined in accordance with applicable local laws and regulations). (revised per Amendment 07)

17. Willing and able to comply with all aspects of the protocol

9.3.2 Exclusion Criteria

1. Any neurological condition that may be contributing to cognitive impairment above and beyond that caused by the subject’s AD
2. History of transient ischemic attacks, stroke, or seizures within 12 months of Screening
3. Any psychiatric diagnosis or symptoms, (eg, hallucinations, major depression, or delusions) that could interfere with study procedures in the subject
4. GDS score ≥8 at Screening
5. Contraindications to MRI scanning, including cardiac pacemaker/defibrillator, ferromagnetic metal implants (eg, in skull and cardiac devices other than those approved as safe for use in MR scanners)
6. Evidence of other clinically significant lesions that could indicate a dementia diagnosis other than AD on brain MRI at Screening. All MRIs will be acquired using a standardized procedure that will be outlined in the Imaging Charter and Imaging Acquisition Guidelines (IAG) and will be read by an approved centralized reader.
7. Other significant pathological findings on brain MRI at Screening, including but not limited to: more than 4 micro-hemorrhages (defined as 10 mm or less at the greatest diameter), a single macro-hemorrhage greater than 10 mm at greatest diameter; an area of superficial siderosis; evidence of vasogenic edema; evidence of cerebral contusion, encephalomalacia, aneurysms, vascular malformations, infective lesions, evidence of multiple lacunar infarcts or stroke involving a major vascular territory, severe small vessel, or white matter disease or space occupying lesions or brain tumors (however lesions diagnosed as meningiomas or arachnoid cysts and <1cm at their greatest diameter need not be exclusionary) (revised per Amendment 07)
8. Hypersensitivity to BAN2401 or any of the excipients, or to any monoclonal antibody treatment
9. Any immunological disease which is not adequately controlled, or which requires treatment with biologic drugs during the study
10. Subjects with a bleeding disorder that is not under adequate control (including a platelet count <50,000 or international normalized ratio [INR] >1.5)

11. Subjects who have thyroid stimulating hormone (TSH) above normal range. Other tests of thyroid function with results outside the normal range should only be exclusionary if they are considered clinically significant by the investigator. This applies to all subjects whether or not they are taking thyroid supplements. (revised per Amendments 01 and 06)
12. Abnormally low serum Vitamin B12 levels for the testing laboratory (if subject is taking Vitamin B12 injections, level should be at or above the lower limit of normal [LLN] for the testing laboratory).
13. A prolonged QT/QTc interval (QTc >450 ms) as demonstrated by a repeated ECG
14. Known to be human immunodeficiency virus (HIV) positive
15. Any other clinically significant abnormalities in physical examination, vital signs, laboratory tests or ECG at Screening or Baseline which in the opinion of the PI, require further investigation or treatment or which may interfere with study procedures or safety
16. Uncontrolled Type 1 or Type 2 diabetes mellitus. Evidence of uncontrolled diabetes mellitus includes hemoglobin A1c (HbA1c) >9%
17. Uncontrolled hypertension with a history of blood pressure consistently above 165/100 mm Hg at Screening
18. History of uncontrolled cardiovascular disease within 6 months of Screening , including acute coronary syndrome, clinically significant valvular heart disease, uncompensated heart failure (New York Heart Association Class III and Class IV), or uncontrolled arrhythmia
19. Subjects with malignant neoplasms within 3 years of Screening (except for basal or squamous cell carcinoma *in situ* of the skin, or localized prostate cancer in male subjects). Subjects who had malignant neoplasms but who have had at least 3 years of documented uninterrupted remission before Screening need not be excluded.
20. Has a “yes” answer to the C-SSRS suicidal ideation Type 4 or 5, or any suicidal behavior assessment within 6 months before Screening, at Screening, or at the Baseline Visit; or has been hospitalized or treated for suicidal behavior in the past 5 years before Screening
21. Known or suspected history of drug or alcohol abuse or dependence within 2 years before Screening or a positive urine drug test at Screening. Subjects who test positive for benzodiazepines or opioids in urine drug testing need not be excluded if in the clinical opinion of the investigator, this is due to the subject taking prior/concomitant medications containing benzodiazepines or opioids for a medical condition and not due to drug abuse.
22. Any other medical conditions (eg, cardiac, respiratory, gastrointestinal, renal disease) which are not stably controlled, or which in the opinion of the investigator(s) could affect the subject’s safety or interfere with the study assessments
23. Subjects who are taking prohibited medications (revised per Amendment 01)

24. Participation in a clinical study involving any therapeutic monoclonal antibody, protein derived from a monoclonal antibody, immunoglobulin therapy, or vaccine within 6 months before Screening (revised per Amendment 01)
25. Participation in a clinical study involving any new chemical entities for AD within 6 months prior to Screening, unless it can be documented that the subject was in a placebo treatment arm
26. Participation in any other investigational medication or device study in the 8 weeks or 5 half-lives (whichever is longer) of the medication before randomization, unless it can be documented that the subject was in a placebo treatment arm
27. Planned surgery which requires general, spinal or epidural anesthesia that would take place during the study. Planned surgery which requires only local anesthesia and which can be undertaken as day case without inpatient stay postoperatively need not result in exclusion if in the opinion of the principal investigator this operation does not interfere with study procedures and subject safety.
28. Severe visual or hearing impairment that would prevent the subject from performing psychometric tests accurately

9.3.3 Removal of Subjects From Therapy or Assessment

The investigator may withdraw the subject from the study at any time for safety or administrative reasons. The subject may stop study drug or withdraw from the study at any time for any reason.

In the Core Study, study drug will be stopped in a subject if any 1 of the following is observed in MRI of the brain: (revised per Amendment 11)

- Evidence of vasogenic edema
- Development of any macrohemorrhages, an area of superficial siderosis, or symptomatic treatment-emergent microhemorrhages

The MRI findings of subjects who develop such features will be investigated together with all accumulated data to date as described in the IAG.

Administration of study drug to an individual subject will be discontinued if any of the following clinical features is observed:

- Infusion reactions associated with administration of study drug, of Grade 3 severity or above as defined in the National Cancer Institute - Common Terminology Criteria for Adverse Events (NCI-CTCAE)
- Clinical features which indicate meningoencephalitis (eg, combination of 1 or more of the following: headache, worsening confusion, neck stiffness, impaired consciousness, focal neurological signs)
- Hypersensitivity reactions with clinical features of tissue injury (eg, arthritis, glomerulonephritis, mononeuritis multiplex) (revised per Amendment 08)

Subjects who meet any of the above criteria, whether on MRI or otherwise, will be discontinued from the study. Subjects who discontinue the study or study drug early must comply with the Early Termination Visit (within 7 days after the last dose of study drug) and the Follow-up Visit (3 months after the last dose of study drug). They may also have unscheduled visits for safety assessments. In addition, it is expected that subjects who discontinue study drug are required to return after the Early Termination Visit for each scheduled visit when clinical efficacy assessments are to be conducted per the Schedule of Procedures/Assessments. (revised per Amendment 06) At these visits, clinical efficacy assessments (MMSE, CDR, ADAS-Cog, and FAQ) will be conducted and information on concomitant medications, AEs, and SAEs will be collected. Regularly scheduled clinical efficacy visits do not need to be attended if they fall within 8 days (visit window) after the Early Termination Visit or within +/- 8 days of the 3-month Follow-Up Visit. Subjects who discontinue from study drug are considered on study as long as they return for their regularly scheduled clinical efficacy visits as outlined above. (revised per Amendments 08 and 11) The timing of unscheduled visits for safety assessments may be decided on a case-by-case basis in the clinical judgment of the investigator in consultation with the sponsor's medical monitor. (revised per Amendment 01)

The primary reason for discontinuation and all other reason(s) contributing to the subject's discontinuation from study drug(s) should be collected in the Early Discontinuation from Study Drug eCRF. In addition, the date of last dose of study drug(s) will be recorded on the Study Drug Administration eCRF page. If a subject discontinues study treatment and the study at the same time, the end-of-study procedures (Final Visit) will be followed (see Section 9.5.5).

9.4 TREATMENTS

9.4.1 Treatments Administered

For the Core Study, the test drug is BAN2401 and the control drug consists of placebo. As shown in Table 4, there will be 6 treatment arms. Subjects in Study Arms 1, 2, 3, and 4, respectively, will receive placebo, 2.5 mg/kg, 5 mg/kg, and 10 mg/kg BAN2401 once every 2 weeks for the duration of the Randomization Phase (18 months). Subjects assigned to Study Arms 5 and 6, respectively, will receive 5 and 10 mg/kg BAN2401 once every 4 weeks for the duration of the study. To maintain blinding and ensure equivalent frequency of infusion-related clinical procedures for all subjects, the subjects assigned to receive BAN2401 at 4-week intervals will have placebo infusions alternating with BAN2401. An infusion system containing a terminal 0.22-µm in-line filter is required for administration of BAN2401 drug product. (revised per Amendment 08)

Study drug doses will not be reduced during the study. If a subject is unable to tolerate the assigned study treatment, the treatment will be discontinued and no further infusions will be administered. Interruptions in scheduled dosing may be necessary in the clinical judgment of the investigator due to illness or other occurrences in study subjects that are not related to study drug but that might interfere with the efficacy or the safety of the study drug.

9.4.2 Identity of Investigational Product(s)

BAN2401 is a humanized IgG1 monoclonal antibody that binds to soluble Aβ aggregates. BAN2401 is manufactured at Patheon Ltd, United Kingdom (UK) and at Biogen, RTP, under contract to Eisai (revised per Amendment 08). During the Core Study, BAN2401 is supplied as a sterile clear solution for injection containing 10 mg/mL, in a single use 10-mL vial (total 100 mg/vial). The drug product is formulated in 25 mM sodium citrate, 125 mM sodium chloride, 0.02% (w/v) polysorbate 80 and has a pH of 5.7. (revised per Amendment 11)

During the Core Study, BAN2401 and placebo will be supplied by the sponsor in labeled containers. The product release certificates for BAN2401 and placebo will be included in the clinical study report for this protocol. (revised per Amendment 11)

During the Core Study, study drug will be packaged such that there is 1 vial per dispenser, and will be dispensed in a blinded manner by an unblinded pharmacist at each site according to the subject randomization created by the Interactive Voice Response System (IVRS). At the time that the study drug is dispensed for administration to the subject, the tear-off label on the dispenser must be removed and placed in the study drug accountability log at the time of dispensing. (revised per Amendment 11)

See Appendix 4 for the identity of the investigation product during the Extension Phase. (Revised per Amendment 11)

9.4.2.1 Chemical Name, Structural Formula of BAN2401

- Test drug code: BAN2401
- Generic name: not applicable
- Chemical name: not yet assigned
- Molecular formula: The heavy chain consists of 454 amino acids and the light chain has 219 amino acids.
- Molecular weight: The apparent molecular weight determined by matrix-assisted desorption ionization time-of-flight mass spectrometry (MALDI TOF-MS) for BAN2401 drug substance is 149,704 Da.
- Structural formula: BAN2401 is composed of 4 polypeptide chains, 2 identical gamma (γ) heavy chains consisting of 454 amino acids each and 2 identical kappa (κ) light chains consisting of 219 amino acids each. The 4 chains are held together by a combination of covalent (disulfide) and noncovalent bonds. There are 32 cysteine residues, and accordingly 16 potential disulfide bonds per residue. The heavy chain subunit contains 2 consensus sequences for potential N-linked glycosylation.

9.4.2.2 Comparator Drug

Matching placebo will be manufactured at Patheon Ltd, UK, under contract to Eisai. It will be supplied as a sterile clear solution for injection, in single use 10-mL vials and should be diluted in 0.9% sodium chloride before use in accordance with the guidance provided.

9.4.2.3 Labeling for Study Drug

The investigational medicinal product, BAN2401, will be labeled in accordance with text that is in full regulatory compliance with each participating country and is translated into the required language(s) for each of those countries. The following information may be included:

(US only) Caution: New Drug – Limited by Federal Law (US) to Investigational Use Only

Protocol number

Name and address of the sponsor

Chemical name/drug identifier

Vial number

Lot number/batch number

Storage conditions, expiration date if necessary

9.4.2.4 Storage Conditions

Study drug will be stored in accordance with the labeled storage conditions. Temperature monitoring is required at the storage location to ensure that the study drug is maintained within an established temperature range. The investigator is responsible for ensuring that the temperature is monitored throughout the total duration of the study and that records are maintained; the temperature should be monitored continuously by using either an in-house validated data acquisition system, a mechanical recording device, such as a calibrated chart recorder, or by manual means, such that minimum and maximum thermometric values over a specific time period can be recorded and retrieved as required.

9.4.3 Method of Assigning Subjects to Treatment Groups

The study is to randomize a maximum of 800 subjects who have early AD. There will be 2 clinical subgroups: those with MCI due to Alzheimer's disease (AD) – intermediate likelihood, and those with mild Alzheimer's disease dementia (collectively referred to as EAD for the purposes of this protocol). The subjects will be randomized to receive 1 of 5 dose regimens of BAN2401, which comprise 3 dose levels given biweekly and 2 dose levels given monthly (2.5, 5, and 10 mg/kg biweekly and 5 and 10 mg/kg monthly [ie, once every 4 weeks]). The randomization of subjects will be stratified by the 2 clinical subgroups (see Inclusion Criteria; MCI due to AD – intermediate likelihood or mild Alzheimer's disease dementia), by APOE status (*APOE4* positive and negative), and by ongoing treatment with AChEIs or memantine or both. Enrollment into the 2 clinical subgroups will be reasonably balanced whereby at least 60% of the total number of subjects will have MCI due to AD – intermediate likelihood and at least 30% will have mild Alzheimer's disease dementia.

The first 196 subjects will be randomized according to a fixed schedule (4:2:2:2:2:2); Placebo (4) to each of the active arms (2 each). Interim analyses will be conducted when 196 and 250 subjects have been randomized and again after every 50 subjects until all 800 subjects are

randomized. The RAR will be updated according to results of interim analyses. Sites in Japan will randomize at least 40 Japanese subjects, even if the early success is declared or the study achieves 800 subjects randomized. In these circumstances, subjects in Japan will be allocated treatment according to the latest (or last) allocation schedule. If the study reaches its maximum of 800 randomized subjects, additional interim analyses will be conducted every 3 months until all 800 complete 12 months of treatment. Only those subjects who were randomized before the target of 800 was met will contribute to these additional interim analyses. (revised per Amendment 07) Each IA will be conducted on data for the derived Clinical Composite Score. On the basis of each IA, the randomization probabilities will be updated for all treatment arms to favor those arms for which the ADCOMS results more closely approximate the potential target dose (ED_{90}). Subjects who are confirmed *APOE4* positive (*APOE4* hetero- or homozygous) will not be randomized to the 10 mg/kg, biweekly dose. (revised per Amendments 04 and 05) The algorithm for computing response adaptive randomization probabilities is further explained in Section 9.7.2.

Oversight of the interim analyses and response adaptive randomization will be conducted by an IMC. The IMC will consist of 3 members who are external to the company and who have with expertise in Bayesian adaptive design for clinical trials.

The interim analyses and RAR will be conducted by an unblinded external independent statistical group in accordance with the protocol. This independent group will provide the IA outcomes to the IMC at each interim look. The IMC will ensure the integrity of the IA and response adaptive randomization process through review of primary efficacy data. In addition, the IMC will act in an independent advisory capacity to monitor the Bayesian IA outcomes according to the early success and futility boundaries prespecified in the protocol. The IMC will inform the sponsor if the boundaries for early success or futility have been crossed. The IMC will not be charged with any subject safety issues, as this will be the responsibility of an independent DSMB.

Subjects who were admitted to the Core Study with EAD and who completed Visit 42 (Week 79) of the Core Study and/or fulfill the Extension Phase inclusion and exclusion criteria (Appendix 4) are eligible to participate in the 24-month Extension Phase. Subjects who previously completed the Core Study (through the Follow-Up Visit, Visit 43) at any time before implementation of the Extension Phase, and/or fulfill the Extension Phase inclusion and exclusion criteria (Appendix 4), will also be eligible to participate. See Appendix 4 for details on assignment of study drug in the Extension Phase. (revised per Amendments 08 and 11)

9.4.4 Selection of Doses and Dose Regimens in the Study

At the present time, there are no nonclinical or clinical data on the PK/PD relationship for functional or behavioral endpoints with BAN2401. In nonclinical studies in Tg mice, BAN2401 treatment produced a reduction of CSF and brain soluble Aβ aggregate species. However, in the BAN2401-A001-101 study, there was no validated assay to measure CSF soluble Aβ aggregated forms. Thus, it was not possible to guide human dose selection based on PD effects of BAN2401 in human CSF.

In the absence of a relevant clinical PD effect, the planned dosing interval and proposed dose range for this study are based on the integrated review of the safety and PK information collected in the combined Phase 1 SAD/MAD study (BAN2401-A001-101) and nonclinical PK/PD modeling of soluble Aβ aggregate brain reduction in mice.

Pharmacokinetic and PD analyses in the Tg-APP ArcSwe mice relating CSF C_{min} to reductions in brain soluble Aβ aggregate concentrations yielded a CSF IC_{50} value of 15.1 ng/mL. Doses of 0.3 mg/kg to 10 mg/kg in Tg-APP ArcSwe mice treated for 17 weeks showed reductions in brain soluble Aβ aggregates. The mean CSF C_{min} of these doses ranged from 27 to 1300 ng/mL. To identify suitable doses, available PK data from SAD and MAD cohorts were modeled to yield CSF exposure estimates for dosing every 2 weeks versus monthly (Table 5). These doses provide a wide range of CSF concentrations. The predicted CSF C_{min} at SS ($C_{min,ss}$) in the present study covers the range of CSF concentrations present in the Tg-APP ArcSwe mice during the 17-week studies. The predicted CSF concentrations in Table 5 also exceed the CSF IC_{50} (15.1 ng/mL) for reduction of brain amyloid aggregates in the Tg-APP ArcSwe mice studies. The exposure of the NOAEL dose of BAN2401 (100 mg/kg) studied in nonhuman primates in the 39-week toxicology study ($C_{max,ss}$ = 4150 µg/mL, $AUC_{(0\text{-}14\ days)}$ = 926,000 µg·h/mL at SS) is at least 16- and 30-fold higher than the predicted serum C_{max} at steady state ($C_{max,ss}$) (251 µg/mL) and $AUC_{(0\text{-}\tau)}$ at SS (30864 µg·h/mL at 14 days dosing interval), respectively, for the highest dose of 10 mg/kg biweekly in the present study.

Table 5 Model-predicted CSF Exposures at Steady State at BAN2401 Doses of 2.5 to 10 mg/kg Biweekly, or 5 to 10 mg/kg Monthly

BAN2401 Dose	CSF $C_{max,ss}$ (ng/mL)	CSF $C_{min,ss}$ (ng/mL)	CSF $AUC_{(0\text{-}t),ss}$ (ng.h/mL)
2.5 mg/kg biweekly	65	44	19661
5 mg/kg biweekly	131	86	38943
10 mg/kg biweekly	261	169	77508
5 mg/kg monthly[a]	103	19	39007
10 mg/kg monthly[a]	206	37	77599

$AUC_{(0\text{-}t),ss}$ = area under the concentration vs. time curve at time t, steady state, CSF = cerebrospinal fluid, $C_{max,ss}$ = maximum observed concentration, $C_{min,ss}$ = minimum observed concentration at steady state
a: 'Monthly' doses are administered once every 4 weeks

It is recognized that a biweekly treatment regimen could represent a significant burden to AD subjects, particularly those who are early in the disease course and who remain highly functional. Exploring the possibility of monthly (ie, once every 4 weeks) dosing of BAN2401 is therefore of interest, and the current study design aims to assess this possibility as well. The doses of 5 mg/kg and 10 mg/kg monthly are predicted to have CSF Cmin,ss that cover the lower end of the range of CSF Cmin in the Tg-APP ArcSwe mice study. Therefore, these doses may still have clinical efficacy.

9.4.5 Selection and Timing of Dose for Each Subject

All infusions will be administered by study personnel. BAN2401 or placebo will be infused over an approximately 60-minute interval in a volume of 250 mL in normal saline. Drug administration will be performed after breakfast. The infusion pump model and other infusion components will be specified in a manual to be distributed to the study sites. The timing of infusions relative to other study procedures and assessments is given in the Schedule of Procedures/Assessments (Table 8 and **Table 12** for those subjects who participate in the Extension Phase). (revised per Amendment 08)

During the beginning of the study, infusions will take place in the clinic. The DSMB will review the safety of BAN2401 after a predetermined number of subjects have been randomized, and decide if it will be acceptable for the option of home infusions (per DSMB charter, if allowed and conducted according to country and local guidelines; home infusions will not be allowed in Germany) to be available to subjects following 4 months of treatment. Upon implementation of Amendment 07, newly enrolled subjects will not be offered the option for home infusions. Subjects opting for home infusions before implementation of Amendment 07 will be allowed to continue with home infusions for the duration of their participation in the study. (revised per Amendments 01, 03, and 07) The investigator should then decide for individual subjects (site permitting) if home infusions are suitable after they have had 4 months of infusions in clinic.

9.4.6 Blinding

This is a double-blind Core Study with an open-label Extension Phase. (revised per Amendment 08) During the Randomization Phase of the Core Study, the subject and all personnel involved with the conduct and the interpretation of the study, including the investigators, investigational site personnel, and Eisai will be blinded to the treatment codes, except that the study pharmacist will be unblinded. Randomization data will be kept strictly confidential until study completion when the last subject completes the last visit in the Extension Phase (revised per Amendment 08), filed securely by an appropriate group in Eisai, and accessible only to authorized persons as per Standard Operating Procedures until the time of unblinding. Designated independent statisticians will be allowed access to the blinded data in order to perform interim analyses and response adaptive randomization (see Section 9.4.3 and Section 9.7.2 for details).

Study drug will be dispensed by an unblinded pharmacist at each site according to the subject randomization generated by the IVRS vendor. A master list of all treatments and the subject numbers associated with them will be maintained in a sealed envelope by the clinical supply vendor, the IVRS vendor, and the sponsor. In the event that emergency conditions require knowledge of the study treatment given, the blind may be broken via the code breaker facility within the IVRS. Emergency procedures for revealing drug codes are given in Section 9.5.4.5. If possible, before breaking the blind, the investigator should consult with the sponsor to ascertain the necessity of breaking the code. The investigator is to make a careful note of the date, time of opening, and the reason for breaking the code.

Subjects in Treatment Arms 5 and 6 will receive BAN2401 infusions once every 4 weeks, whereas all other subjects will receive infusions at biweekly intervals. To maintain blinding across all treatment arms, subjects in Treatment Arms 5 and 6 will receive blinded placebo infusions at 2-week intervals preceding and following each infusion of BAN2401. Refer to Appendix 4 for treatment of subjects in the Extension Phase. (revised per Amendments 08 and 11)

9.4.7 Prior and Concomitant Therapy

Any medication (including over-the-counter medications) or therapy administered to the subject during the study (starting at the date of informed consent) will be recorded on the Prior and Concomitant Medication eCRF or the Non-Pharmacological Procedures eCRF. The investigator will record on the Adverse Event eCRF any adverse event (AE) for which the concomitant medication/therapy was administered. If the concomitant medication/therapy is being administered for a medical condition present at the time of entry into the study, the investigator will record the medical condition on the Medical History and Current Medical Condition eCRF.

9.4.7.1 Drug-drug Interactions

Not applicable

9.4.7.2 Core Study Prohibited Concomitant Therapies and Drugs

Anticoagulants (eg, warfarin, dabigatran) are not permitted for -7 days or 5 half-lives, whichever is longer, before Baseline until the Follow-Up Visit (Appendix 2, Listing 1). (revised per Amendment 01). Subjects who need to start chronic (>4 weeks) anticoagulant treatment during the study for concomitant diseases will be withdrawn from study drug. However, short-term (<4 weeks) treatment with anticoagulants is permitted for randomized subjects who undergo procedures requiring anticoagulants for prophylaxis of thromboembolic disease after approval by the sponsor's medical monitor. While these subjects need not be discontinued, study drug will be temporarily suspended during anticoagulant therapy. (revised per Amendment 06)

Immunoglobulin therapy and therapy with biologic drugs are not permitted for 6 months prior to the Baseline until the Follow-Up Visit. (revised per Amendment 01)

Subjects who are on AChEIs or memantine or both are required to be on a stable dose for 12 weeks before Baseline. During the study, if a subject starts, changes dose, or stops any of these medications, the subject will continue with study visits and assessments to study completion but the data from that subject will be censored as described in Section 9.7.1.6. Use of memantine will not be allowed for Japanese subjects (revised per Amendments 01 and 07)

Medications which are used as needed (PRN) or as short courses of treatment and which are central nervous system-active and may affect cognitive function are not permitted during a period of 72 hours prior to cognitive testing (Appendix 2, Listing 3).

Subjects who are taking other medications will be required to be on a stable dose for at least 4 weeks prior to Baseline, except for medications which are administered as short courses of

treatment (eg, anti-infectives) or which are to be used PRN. (revised per Amendment 01) During the study, subjects who initiate treatment with drugs not intended for treatment of cognitive impairment during the study or who change the doses of such drugs may continue in the study if in the opinion of the Principal Investigator these drugs will not interfere with study procedures or subject safety and are not prohibited above. However, if such drugs may affect cognitive function, subjects should not undertake cognitive assessments until they have been on a stable dose for at least 4 week.

Subjects may receive prophylactic medications as needed prior to infusion of study drug to minimize the risk of immunologic reaction or infusion reaction during or shortly after the infusion.

Restricted and permitted drugs for the Extension Phase are described in Appendix 2. (revised per Amendments 11 and 12)

9.4.8 Treatment Compliance

All study treatments will be administered by study personnel. The scheduled infusion may occur up to 8 days earlier or later than the scheduled date if advisable, as for example due to illness. (revised per Amendment 01) If 1 or more infusions are missed, the investigator and sponsor should evaluate whether or not the subject should continue in the study.

9.4.9 Drug Supplies and Accountability

In compliance with local regulatory requirements, drug supplies will not be sent to the investigator until the following documentation has been received by the sponsor:

- A signed and dated confidentiality agreement
- A copy of the final protocol signature page, signed and dated by both the sponsor and investigator
- Written proof of approval of the protocol, the ICFs, and any other information provided to the subjects by the IRB/IEC for the institution where the study is to be conducted
- A copy of the IRB/IEC-approved ICF and any other documentation provided to the subjects to be used in this study
- The IRB/IEC membership list and statutes or Health and Human Services Assurance number
- A copy of the certification and a table of the normal laboratory ranges for the reference laboratory conducting the clinical laboratory tests required by this protocol
- An investigator-signed and dated Form FDA 1572, where applicable
- (in EU): Approval by a Designated Qualified Person
- Financial Disclosure form(s) for the PI and all subinvestigators listed on Form FDA 1572, where applicable

- A signed and dated curriculum vitae (CV) of the PI including a copy of the PI's current medical license or medical registration number on the CV
- A signed and dated clinical trials agreement
- A copy of the regulatory authority approval for the country in which the study is being conducted and the Import License

The investigator and the study staff or if regionally required, the head of the medical institution or the designated pharmacist will be responsible for the accountability of all study drugs (dispensing, inventory, and record keeping) following the sponsor's instructions and adherence to GCP guidelines as well as local or regional requirements.

Under no circumstances will the investigator allow the study drugs to be used other than as directed by this protocol.

The site must maintain an accurate and timely record of the following: receipt of all study drugs, administration of study drugs to the subject, collection and reconciliation of unused study drugs that are shipped to site, and return of reconciled study drugs to the sponsor or (where applicable) destruction of reconciled study drugs at the site. This includes, but may not be limited to: (a) documentation of receipt of study drugs, (b) study drug dispensing/return reconciliation log, (c) study drug accountability log, (d) all shipping service receipts, (e) documentation of returns to the sponsor, and (f) certificates of destruction for any destruction of study drugs that occurs at the site. All forms will be provided by the sponsor. Any comparable forms that the site wishes to use must be approved by the sponsor.

The study drug and inventory records must be made available, upon request, for inspection by a designated representative of the sponsor or a representative of a health authority (eg, FDA, MHRA). As applicable, all unused study drugs and empty and partially empty containers from used study drugs are to be returned to the investigator by the subject and together with unused study drugs that were shipped to the site but not administered to subjects are to be returned to the sponsor's designated central or local depot(s) during the study or at the conclusion of the study, unless provision is made by the sponsor for destruction of study drugs and containers at the site. Upon completion of drug accountability and reconciliation procedures by the site's personnel and documentation procedures by the sponsor's personnel, study drugs that are to be returned to the sponsor's designated central or local depot(s) must be boxed and sealed and shipped back to the central or local depot(s) following all local regulatory requirements. In some regions, study drugs may be removed from the site and hand delivered to the central or local depot by sponsor representatives.

Drug accountability will be reviewed during site visits and at the completion of the study.

9.5 Study Assessments

Refer to Appendix 4 for a description of Assessments in the Extension Phase. (revised per Amendment 11)

9.5.1 Assessments

9.5.1.1 Demography

Subject demography information will be collected at the Screening Visit. Demographic information is to include date of birth (or age), sex, race/ethnicity, native language, and years of education.

9.5.1.2 Baseline Assessments

MEDICAL HISTORY AND CLINICAL ASSESSMENTS

At the Screening Visit, assessments will be conducted to ensure that subjects do not have medical conditions which may interfere with study participation. Medical and surgical history, current medications including AChE inhibitors and memantine, and current medical conditions will be recorded at this Visit. (revised per Amendment 01) All pertinent medical and surgical history within 10 years must be noted in the Medical History and Current Medical Conditions eCRF.

A complete physical examination will be performed at the Screening Visit. This physical examination will include evaluations of the head, eyes, ears, nose, throat, neck, chest (including heart and lungs), abdomen, limbs, skin, and a complete neurological examination. A urogenital examination will only be required in the presence of clinical symptoms related to this region. Documentation of physical examinations will be included in the source documentation at the site. Significant findings at the Screening Visit will be recorded on the Medical History and Current Medical Conditions eCRF.

Additionally, at the Screening Visit, inclusion/exclusion criteria and prior medications will be reviewed with the subject and caregiver/informant, vital signs will be assessed, height and weight will be recorded for calculation of BMI, blood will be collected for clinical laboratory tests, and a serum pregnancy test (females of childbearing potential only) will be conducted. A urine sample will be collected and tested for common drugs of use/abuse: (eg, amphetamine, barbiturates, benzodiazepines, cocaine, methamphetamine, methadone, opiates, phencyclidine, tetrahydrocannabinol and tricyclic antidepressants).

At the Baseline Visit, inclusion/exclusion criteria and prior medication data will again be reviewed. Vital signs will again be measured and blood will again be collected for clinical laboratory tests and for exploratory plasma biomarker assessments. In addition, a single 12-lead ECG will be conducted. (revised per Amendment 01)

PSYCHOMETRIC ASSESSMENTS

At the Screening Visit, all subjects will be assessed for eligibility, using psychometric assessments to confirm that subjects meet the diagnostic criteria for MCI due to AD – intermediate likelihood or mild Alzheimer's disease dementia. These clinical assessments will begin the Screening assessments and will consist of the MMSE, the WMS-IV LMI and LMII,

and the CDR, and will be administered in that order. These assessments will be administered in the morning whenever possible, and at the same time of day in all subsequent visits. The WMS-IV LMII is to be administered 20 to 30 minutes after the completion of LMI. Subject information should be collected in the 20 to 30 minutes between the WMS-IV LM1 and LMII. (revised per Amendment 01) Subjects will also be evaluated on the GDS for eligibility. Caregivers/informants (defined as persons able to support the subject for the duration of the study) should be present at this visit. Subjects who are considered ineligible for this study based on these assessments should not continue with the rest of the assessments for this visit. (revised per Amendment 01)

At the Baseline Visit, in the morning when possible, subjects will be administered the MMSE, the CDR, the ADAS-Cog, and the FAQ in that order. Test results from the CDR will again be used as inclusion criteria. Caregivers/informants should be present at these visits when the clinical assessments are administered.

The MMSE, the CDR, the ADAS-Cog, and the FAQ will be administered as indicated in the Schedule of Assessments during the Randomization Phase in the order stated.

The C-SSRS will be administered at Screening, Baseline, and at designated Visits as indicated in the Schedule of Assessments.

Mini-Mental State Examination (MMSE)

The MMSE is a cognitive instrument commonly used for screening purposes, but also often measured longitudinally in AD clinical trials.[30] It is a 30-point scale with higher scores indicating less impairment and lower scores indicating more impairment. Seven items measuring orientation to time and place, registration, recall, attention, language and drawing will be assessed.

Wechsler Logical Memory Test

The WMS-IV Logical Memory II Test (Delayed Paragraph Recall)[31] consists of 2 stories for the assessment of free recall. This modified version now contains a paragraph recall for the older age range of 65 to 90 years of age, where a new story was developed with content more relevant for this age group. The new story is shorter and repeated once to enable an adequate floor through age 90. Stories are not repeated for subjects who do not fall into the age range. Immediate (LMI) and delayed recall (LMII) scores will be obtained. Test result criteria for inclusion in the study based on LMII are provided in Section 9.3.1.

Clinical Dementia Rating (CDR)

The CDR is a clinical scale that describes 5 degrees of impairment in performance on each of 6 categories of function including memory, orientation, judgment and problem solving, community affairs, home and hobbies, and personal care.[32] The ratings of degree of impairment obtained on each of the 6 categories of function are synthesized into 1 global rating of dementia CDR score (ranging from 0 to 3). A sum of boxes score provides an additional measure of change where each category has a maximum possible score of 3 points and the total score is a

sum of the category scores giving a total possible score of 0 to 18 with higher scores indicating more impairment. The global score will be used as a clinical measure of severity of dementia. Test result criteria for inclusion in the study are given in Section 9.3.1.

Geriatric Depression Scale

The Geriatric Depression Scale (GDS): (Short Form) is a self-reported, clinician-assisted scale designed to identify symptoms of depression in the elderly.[33] (revised per Amendment 01) The scale consists of 15 printed questions that the subject is asked to answer by circling yes or no on the basis of how they felt over the past week. The items are presented on a single page with more benign items presented first. Answers to 5 of the items are negatively oriented for depression (eg, Do you feel full of energy?) and 10 positively oriented (eg, Do you often feel helpless). One point is given for each appropriate positive or negative answer indicative of a symptom of depression, for a possible total of 15 points. Total scores of 0 to 5 are considered normal and scores of 6 to15 are considered depressed. Test result criteria for inclusion in the study are given in Section 9.3.2.

Alzheimer's Disease Assessment Scale-Cognitive (ADAS- cog)

The ADAS-Cog is the most widely used cognitive scale in Alzheimer's disease trials. It is a structured scale that evaluates memory (word recall, delayed word recall, and word recognition), reasoning (following commands), language (naming, comprehension), orientation, ideational praxis (placing letter in envelope) and constructional praxis (copying geometric designs).[34] Ratings of spoken language, language comprehension, word finding difficulty, ability to remember test instructions, maze, and number cancellation are also obtained. The modified version used in this study is scored from 0 to 90 points with a score of 0 indicating no impairment, and a score of 90 indicating maximum impairment.

IMAGING ASSESSMENTS

During the Screening Visit, subjects who meet psychometric assessment criteria for eligibility will undergo an eligibility assessment using safety MRI to detect brain abnormalities which may exclude study participation. Safety MRI assessments will be conducted at regularly scheduled visits according to the Schedule of Procedures/Assessments. In all cases, the safety MRI must be reviewed by both the imaging vendor and a local reader prior to administration of the next dose of study drug for each respective visit. (revised per Amendment 06) An additional scanning sequence will be conducted in all subjects to obtain vMRI assessments. The safety and vMRI assessments conducted during Screening will also be used as the baseline MRI assessments for these respective measures in eligible subjects. Methods for MRI assessments are described in Section 9.5.1.5.

For further assessments of eligibility, all qualified subjects will then undergo amyloid positron emission tomography (amyloid PET) or CSF Aβ(1-42) at the Baseline Visit to confirm deposition of amyloid in the brain. Baseline amyloid PET data will serve as the baseline data for subjects who consent to participate in the imaging subgroup (target n=306 total who complete 12 months of treatment in the Core Study) and who receive amyloid PET at 12 and 18 months of

treatment. (revised per Amendment 08) Methods for amyloid PET are described in Section 9.5.1.6. (revised per Amendments 02, 06, and 07)

Descriptions and detailed instructions for all imaging will be distributed to all imaging sites.

BIOMARKER AND PHARMACOGENETIC SAMPLING

At the Baseline Visit, blood samples will be drawn for pharmacogenomic assessment (mandatory *APOE4*) and for exploratory biomarkers consistent with Appendix 3, related to potential PD and safety readouts. (revised per Amendment 01)

Also at the Baseline Visit, consenting subjects will undergo CSF sampling by lumbar puncture. Lumbar punctures for CSF sampling will again be performed at 12 and 18 months after the start of treatment. Each subject should undergo all 3 LPs at approximately the same time of day. The Baseline LP CSF sample will be used for eligibility and for establishing the subject's baseline for the longitudinal assessment. (revised per Amendment 06)

9.5.1.3 Efficacy Assessments

RATIONALE FOR PRIMARY EFFICACY ENDPOINT

No well-established, validated clinical endpoints exist that are sensitive to change in MCI populations in clinical trials. In fact, there has been concern that the standard cognitive instrument, the ADAS-Cog, may not have sufficient sensitivity to change even in a mild Alzheimer's disease dementia population. Demonstration of an effect on slowing clinical progression in an EAD population is hampered by this lack of sensitive tools for measuring relatively subtle longitudinal changes or treatment effect in early disease. Heterogeneity of the MCI population as measured by established cognitive instruments, such as ADAS-Cog, presents another challenge for clinical trials in EAD and points to an acute need for a clinical tool that will be responsive to progression and treatment effects in EAD.

Clinical data were utilized from multiple MCI trials to develop a new score that would demonstrate maximum responsiveness to progression and to treatment in an MCI population and that would also perform well in a mild Alzheimer's disease dementia population. A partial least squares (PLS) regression model used placebo data from 4 MCI studies over 12 months to select the combination of cognitive and functional items which is most sensitive to change over time, using items from a variety of well-established and validated scales. The PLS regression coefficients from the model were used to form a weighted composite score. The resulting composite comprises ADAS-Cog, MMSE and CDR items. Responsiveness was assessed by comparing the Mean to Standard Deviation Ratio between the composite and standard scales in an MCI population. Responsiveness was also assessed in enriched MCI subgroups (CSF Aβ positive and apolipoprotein E4 [*APOE4*] positive), in the presence of a treatment effect and in a mild Alzheimer's disease dementia subject population combining placebo data from 3 studies.

The new score is termed the Composite Clinical Score, and represents a new approach to the analysis of selected items (12 total) from the ADAS-Cog (4 items), the MMSE (2 items), and the

CDR (all 6 items). The composite score is more sensitive to clinical progression compared to currently existing clinical batteries and requires smaller sample sizes for clinical studies in EAD. (revised per Amendment 01) It also shows sensitivity to treatment effects with AChEIs. The composite score shows similar behavior in MCI due to AD and Mild Alzheimer's disease dementia. This score assesses both cognitive and functional domains and can be offered as a single primary clinical endpoint. The Composite Clinical Score is calculated as described in Section 9.7.1.6. It has a range from 0 to 1.97.

To obtain a Composite Clinical Score each subject will be administered the MMSE, the CDR, and the ADAS-Cog. Items from these 3 psychometric tests will be used as described in the calculation in Section 9.7.1.6 to derive the composite score. The timing of these assessments is shown in the Schedule of Procedures/Assessments (Table 7 and Table 8 for the Core Study and for the Extension Phase). (revised per Amendment 08) They should be administered in the morning or (if not possible for a given subject) consistently at the same time of day.

9.5.1.4 Pharmacokinetic, Pharmacodynamic, and Pharmacogenomic/Pharmacogenetic Assessments

PHARMACOKINETIC ASSESSMENTS

During the Randomization Phase, blood samples (3 ml each) will be collected from all subjects for determination of serum BAN2401 levels at approximately 12-week intervals (Table 8). (revised per Amendment 08)

At Visit 3, blood will be taken for the BAN2401 assay approximately 4 hours after the end of infusion. Subjects must stay in clinic for the full 4 hours following infusion during this first infusion visit. At Visits 9, 16, 22, 29, 35, and 41, blood for this assay will be drawn both predose and at least 2 hours after the end of infusion. These samples should not be taken from the same arm as that in which the study drug is infused. Subjects must stay in clinic for at least 2 hours following infusion up through Week 13, Visit 9. After the Week 13 Visit (Visit 9), if no untoward effects of infusion are noted, or if infusion reactions can be prevented with prophylaxis as per Section 9.5.1.5, subjects may be discharged from clinic 30 minutes after the end of infusion if judged medically stable by the investigator. (revised per Amendment 07) Subjects are required to remain in clinic for at least 2 hours following infusion at visits where PK samples are taken (except Visit 3, the first infusion visit). PK samples should be taken at least 2 hours after the end of infusion. These samples can be taken any time after those 2 hours and should generally be taken just prior to the subject leaving the site. Each of the study sites will be provided with a laboratory manual describing the collection, handling, and shipping procedures for PK samples. For subjects who participate in the CSF substudy, serum PK sample should also be taken immediately following the CSF collection that is to take place 2 to 4 days after study drug infusion at Visit 29. For the same CSF substudy subjects, the CSF sample should be taken predose at Visit 41 and the predose serum PK sample at Visit 41 should be taken immediately after the CSF sampling. (revised per Amendment 06) Cerebrospinal fluid samples (see below, this Section, for CSF collection methods) will also be used for PK analyses. Blood for PK analyses may also be drawn at the first report of a Serious Adverse Event (SAE) and at its resolution. (revised per Amendment 01)

Samples from all subjects receiving active treatment will be analyzed. Analyses of serum concentrations of BAN2401 will be performed by a CRO using a validated tandem immunoprecipitation liquid chromatography – tandem mass spectrometry (IP/LC-MS/MS) method in which anti-human IgG antibody precipitates BAN2401 from a serum sample. Precipitated BAN2401 will be isolated and will undergo proteolytic enzyme digestion to yield smaller peptides. The amount of peptide with a sequence unique to BAN2401 will be measured by liquid chromatography – tandem mass spectrometry (LC-MS/MS) to provide a quantification of BAN2401. Concentrations of BAN2401 in CSF will be measured by a similar, validated IP/LC-MS/MS method.

Pharmacodynamic Assessments

Not applicable

Pharmacogenomic and Biomarker Assessments

From subjects who have consented to participate in the CSF substudy, CSF (12 mL) will be drawn via lumbar puncture at the Baseline Visit and at Visit 29 and Visit 41 (12 and 18 months) (Table 8 for the Core Study). (revised per Amendments 06 and 08) The LPs at Baseline and Visits 29 and 41 should each be conducted at approximately the same time of day (revised per Amendment 06). If for logistical reasons CSF fluid cannot be collected on the date specified for the Baseline Visit (Day -30 to Day -1), collection may take place at an earlier date provided that consent has been obtained. Each of the study sites will be provided with a laboratory manual describing the preparation of subjects for LP and the collection method (ie, only gravity drip method allowed), handling, and shipping procedures for PD samples. (revised per Amendment 07)

The Baseline LP CSF sample will be used for eligibility and for establishing the subject's baseline for the longitudinal assessment. CSF will be drawn via LP 2 to 4 days after the scheduled study Visit 29 (12 months). CSF samples should be collected predose on the day of the Visit 41 infusion. For those subjects who also consent to both CSF assessment and to amyloid PET, CSF should be collected 2 to 4 days after study drug infusion at Visit 29, and the amyloid PET procedure should be scheduled to take place on a separate day within 10 days of Visit 29, but after CSF collection. (revised per Amendment 06)

Cerebrospinal fluid concentrations of AD-related biomarkers including, but not restricted to Aβ and other isoforms, t-tau, and p-tau will be measured at Baseline and at 12 and 18 months of treatment in consenting subjects. (revised per Amendment 01) Amyloid and tau biomarkers are proposed to be tested using commercially available kits validated for use by a CRO. CSF samples that are left over from the above analyses may be stored and analyzed for biomarkers related to disease mechanism or study drug for up to 15 years after the completion of the study (defined as submission of the CSR to appropriate regulatory agencies).

Blood may also be drawn for exploratory plasma biomarker assessments at Baseline and at 12 and 18 months of treatment to evaluate potential novel biomarkers of AD that may include amyloid isoforms, tau, microRNA, metabolites, and other protein biomarkers (eg, NFL) for

association with AD diagnosis and amyloid load (see Appendix 3). (revised per Amendment 11) Similarly, biomarker discovery and validation may be performed to identify blood and genetic biomarkers which may be useful to predict subject PK and PD response, treatment response, subject stratification or adverse effects related to BAN2401. (revised per Amendment 01) During the Prerandomization Phase, plasma samples will also be collected from all subjects at Baseline prior to amyloid PET assessment and at Visits 3, 9, 16, 29, 35, and 42. See Appendix 4 for details on sample collection for those subjects who participate in the Extension Phase. (revised per Amendment 08)

vMRI sequences will be collected for all subjects immediately following all safety MRI scans (as described in Section 9.5.1.5) except the Visit 6 abbreviated MRI assessment (safety MRI to detect ARIA lasting 8 to 10 minutes, with no vMRI sequences) conducted only at European sites (revised per Amendment 07). vMRI data will be analyzed at the Screening Visit and at Visits 16, 29, and 42 (6, 12, and 18 months of treatment) . vMRI will be conducted at either 1.5T or 3T and across multiple scanner types and the type of scanner used will be documented. vMRI analysis will be performed by a central imaging laboratory on MRI scans obtained at the Screening Visit and Visits 16, 29, and 42 (6, 12, and 18 months) , according to standard QC and normalization procedures for measurements of the whole brain and total ventricular volume. Measurements of the right and left hippocampal volumes will be based on a validated algorithm and conducted in the same laboratory. Additional analysis of regions of interest and cortical thickness may also be performed. (revised per Amendment 01)

Apolipoprotein E4 (*APOE4*) genotyping will be conducted (Appendix 3) and *APOE4* negative or *APOE4* homozygous or heterozygous status will be used in the statistical analysis to determine the effects on treatment response and safety. This analysis will also assess possible relationships between *APOE4* genotype and the development of amyloid-related imaging abnormalities (ARIA), including vasogenic edema, microhemorrhages, macrohemorrhages, and superficial hemosiderosis. (revised per Amendment 01)

It is possible that future research and technological advances may identify genomic variants of interest, or allow alternative types of genomic analysis not foreseen at this time (Appendix 3). Because it is not possible to prospectively define every avenue of future testing, all samples collected will be single or double coded (according to ICHE15 guidelines: Definitions for Genomic Biomarkers, Pharmacogenomics, Pharmacogenetics, Genomic Data and Sample Coding Categories EMEA/CHMP/ICH/437986/2006) to maintain the ethical principles underlying the provision of informed consent. The focus of research will be the study of genetic factors likely to influence BAN2401 pharmacokinetics, therapeutic response, or susceptibility to Alzheimer's disease. DNA that is left over may be stored and analyzed for up to 15 years after the completion of the study (defined as submission of the CSR to appropriate regulatory agencies). The DNA may be used to examine the role of genetic variability in subjects' absorption, distribution, metabolism, and excretion, or development of AEs. Variation in BAN2401 exposure or AEs may be evaluated by correlation of single-nucleotide polymorphisms with PK, safety or PD data.

PG and biomarker samples obtained from study subjects may be analyzed by global proteomic, metabolomic, or lipidomic and single or multiplex assays in an effort to identify predictive biomarkers for PK and PD. In addition, biomarkers identified in other BAN2401 or AD clinical studies may also be assessed in samples collected from subjects enrolled in this study.

Pharmacogenomic and biomarker analyses will be performed and reported separately. Details of these analyses may be described in a separate analysis plan.

9.5.1.5 Safety Assessments

Safety assessments will consist of monitoring and recording all AEs and SAEs; regular monitoring of hematology, blood chemistry, and urine values; periodic measurement of vital signs and ECGs; and performance of physical examinations. Additional safety assessments specific to this study will include brain MRI, anti-BAN2401 antibody assays, and the C-SSRS.

ADVERSE EVENTS AND OTHER EVENTS OF INTEREST

An adverse event (AE) is any untoward medical occurrence in a subject or clinical investigation subject administered an investigational product. An AE does not necessarily have a causal relationship with the medicinal product. For this study, the study drug is BAN2401.

The criteria for identifying AEs are:

- Any unfavorable and unintended sign (including an abnormal laboratory finding), symptom, or disease temporally associated with the use of an investigational product, whether or not considered related to the investigational product
- Any new disease or exacerbation of an existing disease
- Any deterioration in nonprotocol-required measurements of a laboratory value or other clinical test (eg, ECG or x-ray) that results in symptoms, a change in treatment, or discontinuation of study drug
- Recurrence of an intermittent medical condition (eg, headache) not present pretreatment (Baseline)
- An abnormal laboratory test result should be considered an AE if the identified laboratory abnormality leads to any type of intervention, whether prescribed in the protocol or not.

A laboratory result should be considered by the investigator to be an AE if it:

- Results in the withdrawal of study drug
- Results in withholding of study drug pending some investigational outcome
- Results in an intervention, based on medical evaluation (eg, potassium supplement for hypokalemia)
- Results in any out of range laboratory value that in the investigator's judgment fulfills the definitions of an AE with regard to the subject's medical profile

- Worsens (increases) to Grade 2 or higher based on the Sponsor's Grading for Laboratory Values

All AEs observed during the study will be reported on the eCRF. All AEs, regardless of relationship to study drug or procedure, should be collected beginning from the time the subject signs the study ICF through the last visit (Visit 43, as shown in Table 8 for the Core Study). (revised per Amendments 08, 09, and 11) Serious AEs will be collected up to 3 months after the last dose of study drug or through the last visit, whichever is longer. This includes those subjects who discontinue from study drug and who return for regularly scheduled visits where clinical assessments are conducted. (revised per Amendment 01)

Abnormal laboratory values should not be listed as separate AEs if they are considered to be part of the clinical syndrome that is being reported as an AE. Any laboratory abnormality considered to constitute an AE should be reported on the Adverse Event eCRF.

It is the responsibility of the investigator to review all laboratory findings in all subjects and determine if they constitute an AE. Medical and scientific judgment should be exercised in deciding whether an isolated laboratory abnormality should be classified as an AE.

Abnormal ECG (QTc) results, if not otherwise considered part of a clinical symptom that is being reported as an AE, should be considered an AE if (1) the QTc interval is >450 ms and there is an increase of >60 ms from baseline, or (2) the QTc interval is >500 ms. Any ECG abnormality that the investigator considers as an AE should be reported as such.

It is the responsibility of the investigator to review the results of the C-SSRS in all subjects and to determine if any of these results constitutes an AE. Medical and scientific judgment should be exercised in deciding whether or not an isolated suicidality rating scale response should be classified as an AE.

Every effort must be made by the investigator to categorize each AE according to its severity and its relationship to the study treatment.

ASSESSING SEVERITY OF ADVERSE EVENTS

AEs will be graded on a 3-point scale (mild, moderate, severe) and reported in the detail indicated on the eCRF. The definitions are as follows:

Mild	Discomfort noticed, but no disruption of normal daily activity
Moderate	Discomfort sufficient to reduce or affect normal daily activity
Severe	Incapacitating, with inability to work or to perform normal daily activity

The criteria for assessing severity are different than those used for seriousness (see Serious Adverse Events and Other Events of Interest (Section 9.5.1.5) for the definition of an SAE).

Assessing Relationship to Study Treatment

Items to be considered when assessing the relationship of an AE to the study treatment are:

- Temporal relationship of the onset of the event to the initiation of the study treatment
- The course of the event, especially the effect of discontinuation of study treatment or reintroduction of study treatment, as applicable
- Whether the event is known to be associated with the study treatment or with other similar treatments
- The presence of risk factors in the study subject known to increase the occurrence of the event
- The presence of nonstudy, treatment-related factors that are known to be associated with the occurrence of the event

Classification of Causality

Not Related	A causal relationship between the study treatment and the AE is not a reasonable possibility.
Related	A causal relationship between the study treatment and the AE is a reasonable possibility. The investigator must further qualify the degree of certainty as "possible" or "probable."

Serious Adverse Events and Other Events of Interest

A serious adverse event (SAE) is any untoward medical occurrence that at any dose:

- Results in death
- Is life-threatening (ie, the subject was at immediate risk of death from the adverse event as it occurred; this does not include an event that, had it occurred in a more severe form or was allowed to continue, might have caused death)
- Requires inpatient hospitalization or prolongation of existing hospitalization
- Results in persistent or significant disability/incapacity
- Is a congenital anomaly/birth defect (in the child of a subject who was exposed to the study drug)

Other important medical events that may not be immediately life-threatening or result in death or hospitalization but, when based on appropriate medical judgment, may jeopardize the subject or may require intervention to prevent 1 of the outcomes in the definition of SAE listed above should also be considered SAEs. Medical and scientific judgment should be exercised in deciding whether expedited reporting is appropriate in such situations.

In addition to the above, **other events of interest** include pregnancy, any treatment-emergent significant laboratory abnormality, and vasogenic edema, macrohemorrhages, superficial siderosis, or new microhemorrhages on brain MRI, infusion reactions (any grade of severity as per NCI-CTCAE criteria), skin rash considered to be due to study drug, other hypersensitivity

reactions, or a “yes” response to C-SSRS suicidal ideation type 4 or 5, an increase in body temperature to greater than 38ºC within 24 hours postdose, or AEs associated with study drug overdose, or medication error. These events of interest are to be captured using the SAE procedures but are to be considered as SAEs only if they meet 1 of the above criteria. All AEs associated with events of interest are to be reported on the eCRF whether or not they meet the criteria for SAEs. All ‘other events of interest’ noted above, including those that are asymptomatic, are considered as AEs for the purposes of this protocol.

The following hospitalizations are not considered to be SAEs because there is no “adverse event” (ie, there is no untoward medical occurrence) associated with the hospitalization:

- Hospitalizations for respite care
- Planned hospitalizations required by the protocol
- Hospitalization planned before informed consent (where the condition requiring the hospitalization has not changed post study drug administration)
- Hospitalization for administration of study drug or insertion of access for administration of study drug
- Hospitalization for routine maintenance of a device (eg, battery replacement) that was in place before study entry

If possible, a blood sample for the measurement of study drug concentration should be drawn at the first report of an SAE or a severe unexpected AE and at its resolution. (revised per Amendment 01)

Laboratory Measurements

Clinical laboratory tests to be performed, including hematology, chemistry, and urinalysis, are summarized in Table 6. Subjects should be in a seated or supine position during blood collection. The Schedule of Procedures/Assessments (Table 7 and Table 8 for the Core Study and for the Extension Phase) shows the visits and time points at which blood (10 mL) for clinical laboratory tests and urine for urinalysis will be collected in the study. (revised per Amendment 08) All blood and urine samples will be collected and sent to the central laboratory on the day of collection. In cases of a safety concern, blood samples will be split (or 2 samples drawn) to allow a local laboratory analysis in addition to the central laboratory. Results from the central laboratory will be electronically transferred to the clinical database. If laboratory results suggest symptoms of hypersensitivity, the case should be discussed with the sponsor medical team and further investigations will be conducted as necessary.

Table 6 Clinical Laboratory Tests (Revised per Amendment 11)

Category	Parameters
**Hematology**	hematocrit, hemoglobin, platelets, RBC count, and WBC count with differential (basophils, eosinophils, lymphocytes, monocytes, neutrophils) (revised per Amendment 01)
**Chemistry**	
Electrolytes	Bicarbonate, chloride, potassium, sodium
Liver function tests	alanine aminotransferase, alkaline phosphatase, aspartate aminotransferase, direct bilirubin, total bilirubin
Renal function parameters	blood urea/blood urea nitrogen, creatinine
Other	albumin, calcium, cholesterol, globulin, glucose, lactate dehydrogenase, total protein, triglycerides, uric acid
**Urinalysis**	Color, appearance, bacteria, casts, crystals, epithelial cells, glucose, ketones, occult blood, pH, protein, RBCs, specific gravity, WBCs, bilirubin, urobilinogen, yeast, mucus, nitrite, leucocyte esterase
**Other**	At screening only: free T3, free T4, TSH, Vitamin B12, and HbA1c.<br><br>At screening and Extension Screening Visit:: clotting screen (prothrombin time [PT, INR], activated partial thromboplastin time [APTT])

HbA1c = hemoglobulin A1c, INR = international normalized ratio, RBC = red blood cell, T3 = triiodothyronine, T4 = thyroxine, TSH = thyroid stimulating hormone, WBC = white blood cell.

Clinical laboratory tests during the study will be performed by the central laboratory for this study. Laboratory certification will be included in the final clinical study report for this study.

Blood samples will be collected and stored and measurements of biomarkers related to hypersensitivity reactions may be conducted on these samples if safety data on hypersensitivity reactions (see Hypersensitivity Reactions below**)** indicate that it is appropriate to do so.

A laboratory abnormality may meet the criteria to qualify as an AE as described in this protocol (see Adverse Events and Other Events of Interest [Section 9.5.1.5]) and the eCRF Completion Guidelines. In these instances, the AE corresponding to the laboratory abnormality will be recorded on the Adverse Event eCRF.

For laboratory abnormalities meeting the criteria of SAEs (see Serious Adverse Events and Other Events of Interest, this Section), the site must fax or email the SAE report including the laboratory report (as regionally required) to the sponsor using the SAE form (see Reporting of Serious Adverse Events [Section 9.5.4.1]).

INFUSION REACTIONS

Study drug infusion reactions (within 24 hours of infusion) must be reported as AEs of interest. They will be graded according to the NCI-CTCAE, Version 4.0, grading of allergic/hypersensitivity reactions/cytokine release, as follows: (revised per Amendment 06)

- Grade 1: mild reaction, infusion interruption not indicated, intervention not indicated
- Grade 2: infusion interruption or treatment indicated, but responds promptly to symptomatic treatment (eg, antihistamines, nonsteroidal anti-inflammatory drugs (NSAIDs), IV fluids); prophylactic medications indicated for ≤24 hours
- Grade 3: prolonged (eg, not rapidly responsive to symptomatic medications and/or brief interruption of infusion); recurrence of symptoms following initial improvement; hospitalization required for clinical sequelae (eg, renal impairment)
- Grade 4: life-threatening consequences; urgent treatment needed (eg, vasopressor or ventilatory support)
- Grade 5: death

Infusion reactions of any grade outlined above (Grade 1-5) should be reported as AEs of special interest. Subjects who experience infusion reactions of Grade 3 or 4 will be discontinued from study drug and will have an Early Termination Visit and a Follow-up Visit 3 months after the last dose of study drug. In addition, subjects who discontinue study drug are required to return after the Early Termination Visit for each scheduled visit when the clinical assessments of efficacy are to be conducted per the Schedule of Procedures/Assessments. (revised per Amendment 06) At these visits, safety information (in addition to efficacy assessments) on concomitant medications, AEs, and SAEs will be collected. Unscheduled visits for safety may also be conducted as warranted in the clinical judgment of the investigator. (revised per Amendment 01)

The following are treatment guidelines for BAN2401 infusion reactions:

Grade 2: Stop the infusion. Administer diphenhydramine hydrochloride 25 to 50 mg orally (PO) or dexamethasone 10 mg intravenously (IV) and acetaminophen 650 mg to 1 g PO. (revised per Amendment 06) The investigator may also use other antihistamines, corticosteroids, and anti-inflammatory drugs as per local treatment guidelines. If the infusion reaction improves or resolves, infusion may be resumed if the investigator considers it safe to do so in his/her clinical judgment. If so, then resume the infusion at 50% of the prior rate once the infusion reaction has resolved; the infusion duration should not exceed 2 hours. Monitor for worsening of condition. (revised per Amendment 06)

For subsequent infusions, the investigator should consider premedicating with diphenhydramine hydrochloride 25 to 50 mg (PO or IV), dexamethasone (10 mg IV) and acetaminophen 650 mg to 1 g orally. Other antihistamines, corticosteroids, and anti-inflammatory drugs as per local treatment guidelines may be used as alternatives. Use of dexamethasone or any other

corticosteroids for premedication should be done with caution. (revised per Amendment 06) The investigator should consider administering infusions at 50% of the original study rate. If a subject does not experience infusion reactions upon the next several administrations, the investigator may stop premedicating the subject and may increase the infusion as per original study rate for subsequent infusions.

Grade 3: Stop the infusion and disconnect the infusion tubing from the subject. Administer diphenhydramine hydrochloride 25 to 50 mg PO or IV, dexamethasone 10 mg IV (or equivalent), acetaminophen 650 mg to 1 g orally, bronchodilators for bronchospasms, and IV fluids for hypotension. Administer other medications/treatments as medically indicated. Hospital admission for observation may be indicated. (revised per Amendment 06) Discontinue subject from study drug and conduct an Early Termination Visit within 7 days of discontinuation and a Follow-up Visit 3 months later. (revised per Amendment 01)

Grade 4: Stop the infusion and disconnect the infusion tubing from the subject. Administer diphenhydramine hydrochloride 25 to 50 mg PO or IV dexamethasone 10 mg IV (or equivalent), and acetaminophen 650 mg to 1 g orally, bronchodilators for bronchospasm, IV fluids and IV adrenaline for hypotension. Administer other medications/treatments as medically indicated. Hospital admission for treatment is likely indicated. Discontinue subject from study drug and conduct an Early Termination Visit within 7 days of discontinuation and a Follow-up Visit 3 months later. (revised per Amendment 01)

HYPERSENSITIVITY REACTIONS

Apart from infusion reactions, subjects will also be monitored for hypersensitivity reactions (thought to arise from Type II, III, IV, or other hypersensitivity mechanisms) by AEs, physical examinations, and laboratory tests. The investigator should discuss with the sponsor's medical team the clinical features or laboratory evidence associated with hypersensitivity reactions. Subjects who have hypersensitivity reactions with clinical features of tissue injury (eg, arthritis, glomerulonephritis, mononeuritis multiplex) will be discontinued from study drug but will comply with the Early Termination Visit (within 7 days after the last dose of study drug) and the Follow-up Visit (3 months after the last dose of study drug). (revised per Amendment 06) In addition, subjects who discontinue study drug are required to return after the Early Termination Visit for each scheduled visit when the clinical assessments of efficacy are to be conducted per the Schedule of Procedures/Assessments. At these visits, safety information (in addition to efficacy assessments) on concomitant medications, AEs, and SAEs will be collected. (revised per Amendment 01)

For hypersensitivity reactions in the skin, subjects may be treated with diphenhydramine hydrochloride 25 to 50 mg PO or IV. Dermatologic opinion may be sought with regard to treatment with topical corticosteroids. For hypersensitivity reactions in the skin, subjects need not be discontinued from study drug if such reactions are mild to moderate in intensity and respond to treatment, after discussion with sponsor medical monitor. Otherwise the investigator should discontinue study drug in subjects whose skin hypersensitivity reactions are severe, or respond poorly to treatment, or likely to worsen with continued exposure to study drug. (revised

per Amendment 06) For hypersensitivity reactions with other systemic manifestations or tissue injury, specialist opinion should be sought with regard to treatment.

VITAL SIGNS AND WEIGHT MEASUREMENTS

Vital sign measurements (ie, systolic and diastolic blood pressure [BP] [mmHg], pulse [beats per minute], respiratory rate [per minute], and body temperature [in centigrade]), and weight (kg) will be obtained using validated methods, before and after infusions as indicated in the Schedule of Procedures/Assessments (Table 7 and Table 8 for the Core Study). (revised per Amendment 08) All vital signs will be measured after the subject has been in a semi-supine position for 3 minutes. All BP measurements should be performed on the same arm, preferably by the same person. During Study Visits 3, 4, 5, and 6, vital signs should be obtained at least 2 hours after infusion. If at those visits no untoward effects of infusions on vital signs are detected ≥2 hours after infusion (revised per Amendment 06), these assessments at subsequent study visits may be conducted at a shorter interval after infusion.

PHYSICAL EXAMINATIONS

A complete physical examination will be performed at Screening as described in Section 9.5.1.2. During the Randomization Phase, routine physical examinations will take place as shown in the Schedule of Procedures/Assessments (Table 8). These examinations will include the cardiovascular system, respiratory system and neurological system. Health status will be evaluated by brief evaluation of the eyes, ears, nose, throat, and other physical conditions of note. The subject must be queried regarding changes in physical status since the last examination. A urogenital examination will only be required in the presence of clinical symptoms related to this region. Documentation of the physical examination will be included in the source documentation at the site. Only changes from screening physical examination findings that meet the definition of an AE will be recorded on the Adverse Events eCRF.

ELECTROCARDIOGRAMS

Single electrocardiograms will be obtained as designated on the Schedule of Procedures/Assessments Table 7 and Table 8 for the Core Study). (revised per Amendment 08) An ECG abnormality may meet the criteria of an AE as described in this protocol (see Adverse Events and Other Events of Interest [Section 9.5.1.5]) and the eCRF Completion Guidelines. In these instances, the AE corresponding to the ECG abnormality will be recorded on the Adverse Events eCRF.

For ECG abnormalities meeting criteria for an SAE (see Serious Adverse Events and Other Events of Interest [Section 9.5.1.5]), the site must fax or email the SAE report including the ECG report to the sponsor using the SAE form (see Reporting of Serious Adverse Events [Section 9.5.4.1]).

If QTc is found to be out of range, 2 additional ECGs will be done to allow evaluation of triplicate ECGs. The subject should be withdrawn if (1) the absolute value of the QTc becomes greater than 500 ms absolute value or (2) if the QTc increases by more than 60 ms from baseline and the QTc is greater than 450 msec.

BRAIN MRI

During the Screening Period (Day –60 to Day –31), and during the Randomization Phase as shown in the Schedule of Assessments (Table 7 and Table 8 for the Core Study). (revised per Amendment 08), all subjects will undergo non-contrast MRI. MRI may be conducted at any time following the immediately preceding visit and prior to each of the designated study visits during treatment phase as shown in the Schedule of Assessments. In all cases, the safety MRI must be reviewed by the imaging vendor and a local reader, with agreement that none of the abnormalities on MRI which require discontinuation of study drug are present, prior to a subject receiving the next dose of study drug. (revised per Amendment 06) In the event of an unscheduled visit, the investigator in consultation with the study medical monitor will determine whether or not a safety MRI should be conducted. If an Early Termination Visit takes place, regardless of reason, an MRI is to be conducted if not performed within the last 90 days. Sedation will be permitted prior to MRI. A sufficient interval should be allowed between sedation and designated study visits during which psychometric testing takes place, such that there is no carryover of sedative effects which would interfere with testing.

MRI imaging will include FLAIR, GRE, T1, and diffusion-weighted sequences to determine the presence of focal lesions including, but not limited to, evidence for ischemic and hemorrhagic stroke, subdural hematoma, neoplasm, arteriovenous (AV) malformation, micro- and macrohemorrhages, superficial siderosis, lacunar infarcts, white matter abnormalities, and vasogenic edema. The imaging sequence for the screening/safety MRI will include: T2 weighted anatomic MR images to rule out major white matter changes, cerebral infarction or other structural lesions and a high-resolution 3D T1-weighted scan with isotropic voxels with volumes of 1 mm^3 or less. In addition, both FLAIR for the detection of vasogenic edema and T2* Gradient Recall Echo for the detection of microhemorrhages will be acquired. The 3D dataset will be reconstructed in transverse slices in (digital imaging and communications in medicine) DICOM format. The results of this MRI assessment will also be used as the Baseline Period MRI, against which changes after drug administration will be compared. Further details of the MRI protocol can be found in the IAG. MRI acquisition and interpretation will be organized centrally to ensure reproducibility and consistency between units involved. (revised per Amendment 01)

At any time during the treatment period, subjects will be withdrawn from further dosing if they develop any of the following features on MRI: vasogenic edema, macrohemorrhage, an area of superficial siderosis or a symptomatic microhemorrhage. All of these events must be reported on the SAE form with the same reporting requirements as an SAE, but these events are not automatically considered serious events. (revised per Amendment 06) These subjects will undergo an Early Termination Visit within 7 days after discontinuation and a Follow-up Visit 3 months after the last dose of treatment. During this interval, they will also undertake an unscheduled visit (with MRI) at approximately 30 days after the visit at which such MRI features were first identified. Additional visits may be arranged if clinically indicated in the opinion of the investigator, and may occur later than 90 days after the final dose of study drug. In addition, subjects who discontinue study drug are required to return after the Early Termination Visit for each scheduled visit when the clinical assessments of efficacy are to be conducted per the

Schedule of Procedures/Assessments. (revised per Amendment 06) At these visits, safety information (in addition to efficacy assessments) on concomitant medications, AEs, and SAEs will be collected. (revised per Amendment 01)

Also, at any time during the treatment period, subjects who have non-symptomatic, treatment-emergent microhemorrhages found on MRI, but none of the features which merit discontinuation of treatment (ie, vasogenic edema, macrohemorrhage, an area of superficial siderosis, or a symptomatic treatment-emergent microhemorrhage) may continue with treatment. These subjects will have safety assessments (including MRI) at approximately 30 days after the visit at which such MRI features were found. They will also continue with the scheduled MRI scans. Additional unscheduled MRI scans may be arranged on an individual subject basis in discussion with the study medical monitor.

Anti-BAN2401 Antibody Assessment

Blood for anti-BAN2401 antibody assessment will be collected predose in a blinded fashion from all subjects during the treatment period at Visits 3, 9, 16, 22, 29, 35, and 42 and at the Early Termination Visit (if applicable) and the Follow-up Visit, as specified in the Schedule of Assessments (Table 8). (revised per Amendments 01 and 08) Anti-BAN2401 antibodies will be processed in an ongoing basis. The principal investigator is required to evaluate whether a subject has any infusion reactions or hypersensitivity to the study medication based solely on clinical findings and laboratory results from hematology, biochemistry and urinalysis, and to decide if the subject should be withdrawn from treatment or receive prophylactic medications prior to the next infusion based on these clinical assessments as outlined in the protocol.

Pregnancy Test

During the Randomization Phase, at study visits specified in Table 7 and Table 8 for the Core Study (revised per Amendment 08), urine samples for pregnancy tests will be taken from females of childbearing potential as defined in Section 9.3.1, Inclusion Criterion 12. Additionally, on the days on which PET scans take place, urine samples will be obtained from these subjects.

Columbia Suicide Severity Rating Scale

Subjects will be assessed for suicidal ideation and suicidal behavior using the C-SSRS according to the designed time points on the Schedule of Procedures/Assessments (Table 7, Table 8). A “yes” answer to type 4 or 5 suicidal ideation or a “yes” response to any suicidal behavior queries will trigger additional psychiatric evaluations by a psychiatrist to determine how the subject is to be managed clinically. All sites are required to identify a psychiatrist for referral purposes. The subject’s ability to continue to participate in the study will be determined by the investigator in consultation with the sponsor’s medical team. The types of C-SSRS forms will be specified in the eCRF or a separate manual to the protocol.

9.5.1.6 Other Assessments

**AMYLOID PET AND CSF Aβ(1-42) ASSESSMENT
(REVISED PER AMENDMENT 06)**

Amyloid PET imaging or CSF Aβ(1-42) assessment will be used to confirm that all subjects with EAD have amyloid deposition in the brain. This criterion will allow for the definition of subjects with MCI due to AD – intermediate likelihood and will confirm amyloid pathology in mild Alzheimer's disease dementia subjects. Florbetapir, flutemetamol, florbetaben, or any approved agent may be used in Canada, the EU, or any other region in which the study is conducted. For screening purposes, the imaging agent of choice will be florbetapir at study sites in the US, while in other regions the choice of imaging agent will depend on local availability of imaging agent, thus allowing for use of florbetaben or flutemetamol as well as florbetapir. (revised per Amendment 07) While florbetapir is the preferred agent in the US, flutemetamol, florbetaben, or any approved agent may be used in the US if needed. For the longitudinal amyloid PET substudy in the Core Study, only florbetapir will be used in the US, while in other regions the choice of imaging agent will depend on local availability of the imaging agent, but the same tracer must be used across all PET assessments per subject. (revised per Amendment 07) Florbetapir, flutemetamol, florbetaben, or any approved agent may be used in Canada, the EU, or any other region in which the study is conducted. (revised per Amendments 02 and 06) Certification of PET scanners, quality control, and analysis of the PET images will be performed by a central vendor. To determine eligibility at Baseline during the Prerandomization Phase, within 20 days after the Baseline Visit, each subject will undergo CSF Aβ(1-42) assessment or amyloid PET imaging with 1 of the aforementioned imaging agents. Eligibility will be based on a binary read (normal or abnormal) for PET, or a specified cut-off for CSF Aβ(1-42). (revised per Amendment 06)

Subjects may consent to both the PET and CSF assessments, but only need a positive amyloid result in only one of the 2 procedures to confirm eligibility (ie, even if one of the 2 results does not meet its respective eligibility criterion). Subjects who initially consent for only one of the amyloid screening assessments will only be allowed to subsequently consent for the second assessment should the first assessment result be positive or they have not yet been informed of the results of the first assessment. (revised per Amendments 06 and 07) To determine eligibility for the study according to CSF Aβ(1-42) levels, a cutoff value for CSF Aβ(1-42) collected using single LP during the Baseline Visit will be applied. Subjects with CSF Aβ(1-42) levels less than this value will be eligible for the study. (revised per Amendment 06)

To determine whether or not the PET scan results allow the subject to remain eligible for the study, uptake patterns will be categorized as "normal" (ie, absence of amyloid deposition) or "abnormal" (ie, presence of amyloid deposition) by 2 independent readers, who will be blinded to each other's observations. Each reader will perform a qualitative visual read of the PET scans and will separately categorize subjects as having either "normal" or "abnormal" uptake on the basis of the PET image pattern. Readers will have been trained and certified to recognize brain PET images with abnormal or normal patterns of uptake. Inter rater and intra rater reliability will be assessed at regular intervals throughout the study.

Each subject's MRI scan will be warped into a template space and the same parameters will be applied to the subject's PET scan, resulting in alignment of the MRI and amyloid PET to the template. The template regions of interest will be then used to sample the warped PET images. Quantification of amyloid levels will be derived using an automated algorithm based on the standard uptake value ratio (SUVR) in the region of interest from 5 to 6 regions for each subject, including the precuneus, anterior cingulate gyrus, frontal lobe, parietal lobe, temporal lobe and the occipital lobe at Baseline and at 12 and 18 months of treatment. A weighted average from these regions in addition to regional values will be derived at baseline and longitudinal assessments.

Subjects who consent to participate in the imaging subgroup (n=306 total who complete 12 months of treatment in the Core Study) will undergo amyloid PET scans at Visits 29 and 42 (12 and 18 months). Duration of the PET scan and its timing relative to injection of the imaging agent will as per the imaging agent manufacturer's guidance. (revised per Amendments 02 and 07) Subjects who participate in the imaging subgroup must undergo a baseline PET scan in this study (ie, historic PET scans are not allowed as baseline scans for the imaging subgroup).

Baseline CSF samples will be used for eligibility and for establishing the subject's baseline for the longitudinal assessment. CSF will be drawn via LP 2 to 4 days after the scheduled study Visit 29 (12 months) . CSF samples should be collected predose on the day of the Visit 41 infusion. For those subjects who also consent to both CSF assessment and to amyloid PET, CSF should be collected 2 to 4 days after study drug infusion at Visit 29, and the amyloid PET procedure should be scheduled to take place on a separate day within 10 days of Visit 29 but after CSF collection. (revised per Amendment 06)

FUNCTIONAL ASSESSMENTS QUESTIONNAIRE (FAQ) (REVISED PER AMENDMENT 01)

On the basis of interviews with caregivers/informants or qualified partners, subjects will be rated for ability to carry out ten complex activities of daily living: 1) manage finances, 2) complete forms, 3) shop, 4) perform games of skill or hobbies, 5) prepare hot beverages, 6) prepare balanced meal, 7) follow current events, 8) attend to television programs, books, and magazines, 9) remember appointments, and 10) travel out of the neighborhood.[35] Each activity will be rated as 0 (normal, does without difficulty), 1 (has difficulty but does by self), 2 (requires assistance), or 3 (dependent). Scores will be summed across items to provide a total disability score (higher scores = greater impairment; maximum score = 30). If an activity was never or very rarely performed premorbidly, it will be marked as "Not Applicable" and will not be included in the score. A proportional score will be derived for subjects who mark any activity as 'Not Applicable' as follows (achieved score / (30 – 3 times the number of activities marked 'Not Applicable')

9.5.2 Schedule of Procedures/Assessments

9.5.2.1 Schedule of Procedures/Assessments

Table 7 presents the Schedule of Screening and Baseline Procedures/Assessments for the Prerandomization Phase. Table 8 presents the Schedule of Procedures/Assessments for Visits 3 through 27 (Panel A), and Visits 28 through 43, including the Follow-up Visit (Panel B).

Further information regarding assessments in the Extension Phase is provided in Appendix 4.

Table 7 Schedule of Screening and Baseline Procedures/Assessments (Visits 1 and 2), Study BAN2401-G000-201 – Core Study

Phase	Prerandomization	
Period	Screening	Baseline
Visit	1	2
Day	-60 to -31	-30 to -1
**Procedures/ Assessments**		
Informed consent	X	
Demographics	X	
Medical history (including AD history) (revised per Amendment 01)	X	
Inclusion/Exclusion	X	X
Vital signs	X	X
Height, weight, and BMI[a]	X	X
Complete physical exam	X	
Routine physical examination		X
12-lead ECG		X
Serum pregnancy test[b]	X	
Urine pregnancy test[b]		X
Blood for laboratory tests	X	X
Urinalysis (revised per Amendment 06)	X	X
Urine drug screen	X	
GDS	X	
MMSE [c]	X	X
WMS-IV LMI and LMII[c]	X	
CDR[c]	X	X
ADAS-Cog[c]		X
FAQ[c]		X
C-SSRS	X	X
Safety MRI [d]	X	
vMRI (for PD)	X	
Amyloid PET[e]		X
Blood for PG[f]		X
Blood for exploratory PD biomarker analysis		X
CSF sampling (for eligibility (revised per Amendment 06), PD and PK) ,[g]		X
Prior / concomitant meds including AChE inhibitors	X	X
Adverse events	X	X

Table 7 Schedule of Screening and Baseline Procedures/Assessments (Visits 1 and 2), Study BAN2401-G000-201 – Core Study

AChE = acetylcholinesterase, AD = Alzheimer's disease, ADAS-Cog = Alzheimer's Disease Assessment Scale-Cognitive subscale, BMI = body mass index, CDR =Clinical Dementia Rating, CSF = cerebrospinal fluid, C-SSRS = Columbia Suicide Severity Rating Scale, ECG = electrocardiogram, FAQ = Functional Assessment Questionnaire, GDS = Geriatric Depression Scale, MCI = mild cognitive impairment, MMSE = Mini Mental State Examination, MRI = magnetic resonance imaging, PD = pharmacodynamic, PET = positron emission tomography, PG = pharmacogenomic, PK = pharmacokinetic, vMRI = volumetric MRI, WMS-IV LMI= Wechsler Memory Scale-Logical Memory (subscale) I, WMS-IV LM II= Wechsler Memory Scale-Logical Memory (subscale) II (revised per Amendment 01)

a: Height measured only at Screening. BMI to be calculated at screening only.

b: Pregnancy tests are only necessary for female subjects of childbearing potential. For those subjects, an additional urine pregnancy test must be done on the day of Amyloid PET scanning.

c: Clinical assessments must be administered before any other screening assessments are conducted. These are to be completed in the morning whenever possible, or consistently at the same time of day. The order of assessments for the Screening Visit is as follows: MMSE, WMS-IV LMI and II, and CDR. will be administered to all subjects and subjects will be diagnosed as either MCI due to AD or mild Alzheimer's disease dementia as defined by the protocol after completion of MMSE, WMS-IV LMI, WMS-IV LMII, and CDR. Once the diagnosis is made, the WMS-IV LMII inclusion criterion can be applied to all subjects. (revised per Amendment 06) The WMS-IV LMII is to be administered 20 to 30 minutes after the completion of LMI. Subject information should be collected in the 20 to 30 minutes between the WMS-IV LM1 and LMII. Subjects who are considered ineligible for this study based on these assessments should not continue with the rest of the assessments for this visit. The order of assessments for the Baseline Visit is as follows: MMSE, CDR, ADAS-Cog, and FAQ. Caregivers/informants need to be present at the Screening and Baseline Visits. (revised per Amendment 01)

d: For subjects who are approved for rescreening, safety MRI and vMRI need not be repeated if the date of the rescreen is <90 days from the date of the original screening MRI.

e: Amyloid PET should be done at least 10 days before the planned randomization date. For those subjects who consent to CSF and PET eligibility assessments, CSF collection should always precede amyloid PET, and the 2 assessments should be separated by at least 24 hours. For these subjects, a positive amyloid result is needed for only one of the procedures to confirm eligibility (ie, even if one of the 2 results renders the subject ineligible). Subjects who initially consent for only one of the amyloid screening assessments will only be allowed to subsequently consent for the second assessment should the first assessment result be positive. Subjects who consent to Amyloid PET or CSF Amyloid are not required to participate in the respective substudies. For subjects who are approved for rescreening, the amyloid PET need not be repeated for eligibility if confirmed amyloid positive during the original screening procedure. If a rescreened subject will participate in the longitudinal PET substudy, quantitative PET collection should be repeated if the original PET collection was done >6 months from the date of the original screening procedure. (revised per Amendments 06 and 07) .

f: For subjects who are approved for rescreening, pharmacogenetic testing need not be repeated if a sample was collected, processed, and *APOE4* results issued during the previous screening procedure. Great care must be taken to link the APOE status information with the new subject number.

g: For those subjects who consent to the longitudinal CSF substudy, Baseline samples will be used for eligibility assessment and to establish a baseline measure for the longitudinal assessments. CSF should be collected at or after the Baseline Visit, and at least 19 days (21 days for sites in the Asia-Pacific region) before the planned randomization date. For those subjects who consent to both CSF and PET eligibility assessments, CSF collection should always precede amyloid PET, and the 2 assessments should be separated by at least 24 hours. For these subjects, a positive amyloid result is needed for only one of the procedures to confirm eligibility (ie, even if one of the 2 results renders the subject ineligible). For subjects who are approved for rescreening, the CSF need not be repeated for eligibility if confirmed amyloid positive during the original screening procedure. If a rescreened subject will participate in the longitudinal CSF sub-study, quantitative CSF collection should be repeated if the original CSF collection was done > 6 months from the date of the original screening procedure. (revised per Amendments 06 and 07) .

Table 8 Schedule of Procedures/Assessments, Core Study BAN2401-G000-201, Panel A, Visits 3 through 27, Weeks 1 through 49 – Core Study

Phase	Randomization																								
Period	Treatment																								
Visit[a]	3	4	5	6	7	8	9	10	11	12	13	14	15	16	17	18	19	20	21	22	23	24	25	26	27
Week	1	3	5	7	9	11	13	15	17	19	21	23	25	27	29	31	33	35	37	39	41	43	45	47	49
**Procedures/ Assessments**																									
Vital signs[d]	X	X	X	X	X	X	X	X	X	X	X	X	X	X	X	X	X	X	X	X	X	X	X	X	X
Weight[e]	X						X							X						X					
Routine physical exam					X				X					X						X					
12-lead ECG					X				X					X						X					
Urine pregnancy test[f]							X							X						X					
Blood for laboratory tests[g]	X	X		X			X			X				X						X					
Urinalysis (revised per Amendment 06)	X	X		X			X			X				X						X					
MMSE[h]							X							X						X					
CDR[h]							X							X						X					
ADAS-Cog[h]							X							X						X					
FAQ[h]							X							X						X					
C-SSRS			X		X		X							X						X					
Safety MRI[i]					X		X							X						X					
Volumetric MRI (for PD)[j]					X		X							X						X					
Abbreviated MRI[k]				X																					
Informed consent[l]																									
Amyloid PET[m]																									
Randomization	X																								
Study drug administration	X	X	X	X	X	X	X	X	X	X	X[n]	X[n]	X[n]	X	X[n]	X[n]	X[n]	X[n]	X[n]	X	X[n]	X[n]	X[n]	X[n]	X[n]
Blood for serum BAN2401 PK[o]	X						X							X						X					
Blood for exploratory PD biomarker analysis																									
Blood for serum anti-BAN2401[p]	X						X							X						X					
CSF sampling[q]																									
Prior / concomitant meds	X			X			X			X			X			X			X			X			X
Adverse events	X	X	X	X	X	X	X	X	X	X	X	X	X	X	X	X	X	X	X	X	X	X	X	X	X

Table 8 Schedule of Procedures/Assessments, BAN2401-G000-201, Panel B, Visits 28 through 43, Weeks 51 through 90 – Core Study

Phase	Randomization																	
Period	Treatment															Early Termination Visit[b]	Follow-up Visit	Unscheduled Visit[c]
Visit[a]	28	29	30	31	32	33	34	35	36	37	38	39	40	41	42		43	--
Week	51	53	55	57	59	61	63	65	67	69	71	73	75	77	79		90	
**Procedures/ Assessments**																		
Vital signs[d]	X	X	X	X	X	X	X	X	X	X	X	X	X	X	X	X	X	X
Weight[e]		X						X									X	
Routine physical exam		X						X							X	X	X	X
12-lead ECG		X						X							X	X	X	X
Urine pregnancy test[f]		X						X							X	X		X
Laboratory tests[g]		X						X							X	X	X	X
Urinalysis (revised per Amendment 06)		X						X							X	X	X	X
MMSE[h]		X						X							X	X	X	
CDR[h]		X						X							X	X	X	
ADAS-Cog[h]		X						X							X	X	X	
FAQ[h]		X						X							X	X	X	
C-SSRS		X						X							X	X	X	X
Safety MRI[i]		X						X							X	X[i]	X	X
Volumetric MRI (for PD)[j]		X						X							X	X	X	X
Abbreviated MRI[k]																		
Informed consent[l]													X					
Amyloid PET[m]		X													X			
Randomization																		
Study drug administration	X[n]	X	X[n]	X[n]	X[n]	X[n]	X[n]	X	X[n]	X[n]	X[n]	X[n]	X[n]	X				
Blood for serum BAN2401 PK[o]		X						X						X		X		X
Blood for exploratory PD biomarker analysis		X													X			
Blood for serum anti-BAN2401[p]		X						X							X	X	X	X
CSF sampling[q]		X												X				
Prior / concomitant meds			X			X			X			X		X		X	X	X
Adverse events	X	X	X	X	X	X	X	X	X	X	X	X	X	X	X	X	X	X

Table 8 Schedule of Procedures/Assessments, Core Study BAN2401-G000-201 (Footnotes)

ADAS-Cog = Alzheimer's Disease Assessment Scale-Cognitive subscale, AE = adverse event, ARIA = amyloid-related imaging abnormalities, CDR = Clinical Dementia Rating, CSF = cerebrospinal fluid, C-SSRS = Columbia Suicide Severity Rating Scale, ECG = electrocardiogram, EU = European Union, FAQ = Functional Assessment Questionnaire, LP = lumbar puncture, MMSE = Mini-Mental State Examination, MRI = magnetic resonance imaging, PD = pharmacodynamic, PET = positron emission tomography, PK = pharmacokinetic. (revised per Amendment 01)

a: Assessments should take place on the first day of the study visit in the designated study week except as noted below (footnotes h, i, and j pertaining to imaging assessments, and footnote o pertaining to CSF sample collection). A visit window of ± 8 days will be allowed for each visit, except for Visit 3. A visit window of ± 7 days will be allowed for the follow-up visit (Visit 43). (revised per Amendment 01)

b: Subjects who discontinue the study or study drug early must comply with the Early Termination Visit (within 7 days after discontinuation the last dose of study drug) and the Follow-Up Visit (3-months after the last dose of study drug). They may also have unscheduled visits for safety assessments. Subjects who discontinue due to APOE status are expected to return for their Early Termination Visit and 3-month Follow-Up Visit (ie, 3 months after the last dose of study drug). Subjects who discontinue due to AE (including ARIA) are not allowed to rescreen for Study BAN2401-G000-201 (revised per Amendment 06). In addition, subjects who discontinue from study drug are expected to return after the Early Termination Visit for each scheduled visit when the clinical assessments of efficacy are to be conducted (Visits 9, 16, 22, 29, 35, and 42). At these visits clinical efficacy assessments (MMSE, CDR, ADAS-Cog, and FAQ) will be conducted and information on concomitant medications, AEs, and SAEs will be collected. Regularly scheduled clinical efficacy visits do not need to be attended if they fall within 8 days (visit window) of the Early Termination Visit or the 3-month Follow-Up Visit. Subjects who discontinue from study drug are considered on study as long as they return for their regularly scheduled clinical efficacy visits as outlined above. (revised per Amendment 01)

c: Unscheduled visits may be conducted at any time that safety or safety MRI data indicate per protocol or as clinically indicated in the judgment of the investigator. Note that assessments indicated under Unscheduled Visits need not always be conducted – actual assessments needed will be determined by the investigator and will be based on the specific visit needs.

d: Vital signs will be measured both at predose and after infusion. During Visits 3, 4, 5, and 6, vital signs should be recorded at least 2 hours after study drug infusion, in addition to predose. If at those visits no untoward effects of infusion on vital signs are detected ≥2 hours after infusion, these assessments at subsequent study visits may be conducted at a shorter interval after infusion. (revised per Amendment 06) -At visits where no infusion takes place (Visits 42 and 43), vital signs will be measured once. Vital sign measurements will consist of systolic and diastolic blood pressure (mmHg) measured after at least 3 minutes in a semi supine position, pulse (beats per minute), respiratory rate (per minute), and body temperature (in centigrade).

e: Weight will be taken in the clinic at designated visits (Visits 3, 9, 16, 22, 29, 35, and 43). If a subject misses a clinic visit where weight is to be collected, subsequent visits should use the most recent, previous collected weight for infusion calculations until the next clinic visit. Under such circumstances, weight is to be taken at the next clinic visit and entered into the IVRS even if the visit is not designated for weight data collection.

f: Females of childbearing potential only

g: Blood for laboratory tests will be taken 4 hours after the end of infusion at Visit 3 and predose at all other visits as indicated.

h: Scales are to be completed in the morning (or, if not possible, consistently at the same time of day) in the following order on the days indicated: MMSE, CDR, ADAS-Cog, and FAQ. Caregivers/informants (defined as a person able to support the subject for the duration of the study) need only to be present at visits where clinical assessment of MMSE, CDR, and ADAS-Cog takes place. (revised per Amendment 01)

i: MRI imaging should be conducted at any time following the immediately preceding visit and prior to each of the following Visits according the Schedule of Procedures/Assessments: Visits 7, 9, 16, 22, 29, 35, and 42 and at the Follow-up Visit (Visit 43). The MRI may be conducted on the same day as the immediately preceding visit, after dosing at that visit. In all cases, the safety MRI must be reviewed by the imaging vendor and a local reader prior to a subject receiving the next dose of study drug. In the event of an Unscheduled Visit, the investigator in consultation with the sponsor will determine whether or not a safety MRI should be conducted. If an Early Termination Visit takes place, an MRI is to be conducted if not already performed during the preceding 90 days. (revised per Amendments 04, 06, and 07)

j: A volumetric MRI sequence will be collected in ALL subjects immediately following all safety MRI assessments. Volumetric MRI data will be analyzed at the Screening Visit and at Visits 16, 29, and 42.

k: The Visit 6 abbreviated MRI will be conducted at EU sites only, and should be conducted at any time following the immediately preceding visit (Visit 5) and prior to dosing at Visit 6. The Visit 6 MRI may be conducted on the same day as Visit 5 after subjects have been dosed. This abbreviated MRI will be comprised of a safety MRI to detect ARIA, lasting approximately 8 to 10 minutes. Volumetric MRI sequences will not be conducted at this visit. The safety MRI must be reviewed by the imaging vendor and a local reader prior to a subject receiving the next dose of study drug. In the event of an Unscheduled Visit, the investigator in consultation with the sponsor will determine whether or not a safety MRI should be conducted. If an Early Termination Visit takes place, an MRI is to be conducted if not already performed during the preceding 90 days. (revised per Amendment 07).

l: Subjects can reconsent to enter the Extension Phase at this visit or any subsequent visit at which they are eligible. Subjects **must** consent to the longitudinal PET substudy for the Extension Phase to participate in the substudy. (revised per Amendment 11)

m: In the imaging subgroup only, amyloid PET imaging will be conducted on or within 10 days after the scheduled Visits 29 and 42 (12 and 18 months). For those subjects who also consent to CSF assessment in addition to PET, CSF should be collected 2 to 4 days after study drug infusion at Visit 29, and the amyloid PET procedure should be scheduled to take place on a separate day within 10 days of Visit 29, but after CSF collection. (revised per protocol Amendment 06) For those subjects who did not consent to the imaging substudy in the Core Study, a baseline scan can be taken at Visit 42 (Week 79). Subjects who consented to the imaging subgroup in the Core Study may continue into the OLE if they reconsent. (revised per Amendments 08 and 11).

n: Subjects may be given the option to receive home infusions (per DSMB charter, if allowed and conducted according to country and local guidelines; home infusions will not be allowed in Germany) for study drug administration during Visits 13, 14, and 15, and Visits 17 through 21, Visits 23 through 28, Visits 30 through 34, and Visits 36 through 40, inclusive, provided they express no clinical features related to drug infusion during the first 4 months of the study. Upon implementation of Amendment 07 newly enrolled subjects will not be offered the option for home infusions. Subjects opting for home infusions before the implementation of Amendment 07 will be allowed to continue with home infusions for the duration of their participation in the study. (revised per Amendments 01, 03, and 07)

o: At Visit 3, blood will be taken for the BAN2401 assay approximately 4 hours after the end of infusion (before subjects leave the clinic for home). Subjects must stay in clinic for the full 4 hours following infusion during this first infusion visit. Subjects must stay in clinic for at least 2 hours following infusion up through Week 13 (Visit 9). After the Week 13 (Visit 9) Visit, if no untoward effects of infusion are noted, or infusion reactions can be prevented with prophylaxis, then subjects may be discharged from clinic 30 min after the end of infusion if judged medically stable by the investigator. At Visits 9, 16, 22, 29, 35, and 41, blood will be taken for the BAN2401 assay both predose and at least 2 hours after the end of infusion (before subjects leave the clinic for home). Subjects are required to remain in clinic for at least 2 hours following infusion at visits where PK samples are taken (except Visit 3, the first infusion visit). PK samples should be taken at least 2 hours after the end of infusion. These samples can be taken any time after those 2 hours and should generally be taken just prior to the subject leaving the site. (revised per Amendment 01) For subjects who participate in the CSF sub-study, serum PK sample should also be taken immediately following the CSF collection that is to take place 2 to 4 days after study drug infusion at Visit 29. For the same CSF sub-study subjects, the CSF sample should be taken predose at Visit 41 and the predose serum PK sample at Visit 41 should be taken immediately after the CSF sampling. (revised per Amendments 06 and 07)

p: Blood for the BAN2401 anti-drug antibody assay will be taken as follows: predose at Visits 3, 9, 16, 22, 29, 35, and 42, and the Follow-up Visit (Visit 43). Blood for the BAN2401 anti-drug antibody assay will also be taken at the Early Termination Visit when applicable.

q: Those subjects who consent to CSF assessments should have CSF drawn via LP 2 to 4 days after the scheduled study Visit 29 (12 months) infusion, and serum PK samples should be taken immediately following the CSF collection. CSF samples should be collected predose on the day of the Visit 41 infusion. (revised per Amendment 06)

9.5.2.2 Total Volume of Blood and CSF

Table 9 presents the number of blood and CSF samples and the total volume of blood and CSF that will be collected throughout the Core Study. Additional samples may be taken at the discretion of the investigator if the results of any tests fall outside reference ranges or if clinical symptoms necessitate testing to ensure subject safety.

Table 9 Summary of Blood and CSF Sample Volumes

Assessment	Total number of collection time points	Sample volume, number of time points x volume per collection (mL)		Total volume (mL)
		Screening and Baseline Periods	Treatment and Follow-up Periods	
Clinical laboratory tests	13	2 x 12 mL	11 x 10 mL	134 mL
Blood sampling for serum anti-BAN2401	8	-	8 x 10 mL	80 mL
Blood sampling for serum BAN2401 PK	13	--	13 x3 mL	39 mL
APOE4 pharmacogenomics	1	1 x 6 mL	None	6 mL
Blood for exploratory biomarker analysis	3	1 x 6 mL	2 x 6 mL	18 mL
All blood samples, total volume collected				277 mL
CSF sampling	3	1 x up to 12 mL	2 x up to 12 mL	Up to 36 mL

APOE4 = apolipoprotein ϵ4 variant, CSF = cerebrospinal fluid, PG = pharmacogenomic, PK = pharmacokinetic

9.5.3 Appropriateness of Measurements

All clinical assessments are standard measurements commonly used in studies of Alzheimer's disease. The appropriateness of the Composite Clinical Score as an efficacy assessment is explained in Section 7.2.1.

Standard safety assessments incorporated into this protocol include vital signs, clinical laboratory assessments, physical examinations, ECG measurements, and reports of AEs. Amyloid-related imaging abnormalities (ARIAs) that have been associated with related therapeutic interventions in AD are readily detected by MRI as planned for this study, in accordance with FDA guidelines.

The anti-BAN2401 assay was developed under the FDA guidance for detecting ADAs, and criteria for evaluating human samples have been developed using a preclinical (rabbit) positive control. The assay is run under GLP.

The C-SSRS has been endorsed by the FDA as a measure of potential suicidality.[36]

9.5.4 Reporting of Serious Adverse Events, Pregnancy, and Other Events of Interest

9.5.4.1 Reporting of Serious Adverse Events

All SERIOUS ADVERSE EVENTS, regardless of their relationship to study treatment, must be reported on a completed SAE form by email or fax as soon as possible but no later than 24 hours after the investigator becomes aware of the event.

Deaths and life-threatening events should be reported immediately by telephone. The immediate report should be followed up within 1 business day by emailing or faxing the completed SAE form.

Serious adverse events, regardless of causality assessment, must be collected up to 3 months after the last dose of study or through the last visit, whichever is longer. This includes those subjects who discontinue from study drug and who return for regularly scheduled visits where clinical efficacy assessments are conducted. Imaging center personnel should promptly report to the investigator any SAEs which occur while the subject is at the imaging center. (revised per Amendment 01)

All SAEs must be followed to resolution or, if resolution is unlikely, to stabilization. Any SAE judged by the investigator to be related to the study treatment should be reported to the sponsor regardless of the length of time that has passed since study completion.

The detailed contact information for reporting of SAEs is provided in the Investigator Study File.

For urgent safety issues call: 1-310-728-5684 (24/7 number). For urgent safety issues, please ensure all appropriate medical care is administered to the subject and contact the appropriate study team member listed in the Investigator Study File.

It is very important that the SAE report form be filled out as completely as possible at the time of the initial report. This includes the investigator's assessment of causality.

Any follow-up information received on SAEs should be forwarded within 1 business day of its receipt. If the follow-up information changes the investigator's assessment of causality, this should also be noted on the follow-up SAE form.

Preliminary SAE reports should be followed as soon as possible by detailed descriptions including copies of hospital case reports, autopsy reports, and other documents requested by the sponsor.

The investigator must notify his/her IRB/IEC of the occurrence of the SAE, in writing, if required by their institution. A copy of this communication must be forwarded to the sponsor or its designee to be filed in the sponsor's Trial Master File.

9.5.4.2 Reporting of Pregnancy and Exposure to Study Drug Through Breastfeeding

Any pregnancy in which the estimated date of conception is either before the last visit or within 3 months of last study treatment or any exposure to study drug through breastfeeding during study treatment or within 3 months of last study treatment must be reported. (revised per Amendment 01)

If an adverse outcome of a pregnancy is suspected to be related to study drug exposure, this should be reported regardless of the length of time that has passed since the exposure to study treatment.

A congenital anomaly, death during perinatal period, an induced abortion, or a spontaneous abortion are considered to be an SAE and should be reported in the same time frame and in the same format as all other SAEs (see Reporting of Serious Adverse Events [Section 9.5.4.1]).

Pregnancies or exposure to study drug through breastfeeding must be reported by fax or email as soon as possible but no later than 1 business day from the date the investigator becomes aware of the pregnancy. The contact information for the reporting of pregnancies and exposure to study drug through breastfeeding is provided in the Investigator Study File. The Pregnancy Report Form must be used for reporting. All pregnancies must be followed to outcome. The outcome of the pregnancy must be reported as soon as possible but no later than 1 business day from the date the investigator becomes aware of the outcome.

A subject who becomes pregnant must be withdrawn from the study.

9.5.4.3 Reporting of Other Events of Interest

REPORTING OF ADVERSE EVENTS ASSOCIATED WITH STUDY DRUG OVERDOSE, MISUSE, ABUSE, OR MEDICATION ERROR

Adverse events associated with study drug overdose, misuse, abuse, and medication error refer to AEs associated with uses of the study drug outside of that specified by the protocol. Overdose, misuse, abuse, and medication error are defined as follows:

Overdose	Accidental or intentional use of the study drug in an amount higher than the protocol-defined dose
Misuse	Intentional and inappropriate use of study drug not in accordance with the protocol
Abuse	Sporadic or persistent intentional excessive use of study drug accompanied by harmful physical or psychological effects
Medication error	Any unintentional event that causes or leads to inappropriate study drug use or subject harm while the study drug is in the control of site personnel or the subject.

All AEs associated with an overdose should be captured on the Adverse Event eCRF. Adverse events associated with overdose, misuse, abuse, or medication error should be reported using the procedures detailed in Reporting of Serious Adverse Events (Section 9.5.4.1) even if the AEs do

not meet serious criteria. Abuse is always to be captured as an AE. If the AE associated with an overdose, or medication error does not meet serious criteria, it must still be reported using the SAE form and in an expedited manner but should be noted as nonserious on the SAE form and the Adverse Event eCRF.

REPORTING OF SIGNIFICANT LABORATORY ABNORMALITY

Any significant treatment-emergent laboratory abnormality observed during the clinical study should be entered on the Adverse Event eCRF and reported using the procedures detailed in Reporting of Serious Adverse Events (Section 9.5.4.1), even if the laboratory abnormality does not meet serious criteria. If the significant laboratory abnormality does not meet serious criteria, it must still be reported using the SAE form and in an expedited manner but should be noted as nonserious on the SAE form and the Adverse Event eCRF.

A laboratory result should be considered a treatment-emergent significant abnormality if the result:

- Is within normal limits at baseline and has increased in severity to meet the sponsor's grading criteria for laboratory values of Grade 3 or above
- Is outside normal limits at baseline and increases in severity to the sponsor's grading criteria for laboratory values of Grade 4 or above.
- Is otherwise considered by the investigator to meet serious criteria as defined in Section 9.5.1.5 (Serious Adverse Events and Other Events of Interest)

Significant laboratory abnormalities should not be listed as separate AEs or SAEs if they are considered to be part of the clinical syndrome that is being reported as an AE or SAE.

REPORTING OF STUDY-SPECIFIC EVENTS

Study-specific events, including pregnancy, any treatment-emergent significant laboratory abnormality, and vasogenic edema (ARIA-E), macrohemorrhages, superficial siderosis, or new cerebral microhemorrhages on brain MRI, infusion reactions (any grade of severity as per NCI-CTCAE criteria), skin rash considered to be due to study drug, other hypersensitivity reactions, or a "yes" response to C-SSRS suicidal ideation type 4 or 5, an increase in body temperature to greater than 38ºC within 24 hours postdose, or AEs associated with study drug overdose, or medication error, should be entered on the Adverse Event eCRF and reported using the procedures detailed in Reporting of Serious Adverse Events (Section 9.5.4.1), even if the study-specific event does not meet serious criteria. (revised per Amendment 08) If the event does not meet serious criteria, it must still be reported using the SAE form and in an expedited manner but should be noted as nonserious on the SAE form and the Adverse Event eCRF.

9.5.4.4 Expedited Reporting

The sponsor must inform investigators and regulatory authorities of reportable events, in compliance with applicable regulatory requirements, on an expedited basis (ie, within specific time frames). For this reason, it is imperative that sites provide complete SAE information in the manner described above.

9.5.4.5 Breaking the Blind

In the case of a medical emergency where the appropriate treatment of the subject requires knowledge of the study treatment given, the investigator may break the randomization code for an individual subject. In all such cases, the AE necessitating the emergency blind break will be handled as an SAE in accordance with the procedures indicated above. Any broken code will be clearly justified and documented. The medical monitor must be notified immediately of the blind break.

9.5.4.6 Regulatory Reporting of Adverse Events

Adverse events will be reported by the sponsor or a third party acting on behalf of the sponsor to regulatory authorities in compliance with local and regional law and established guidance. The format of these reports will be dictated by the local and regional requirements.

All studies that are conducted within any European country will comply with European Good Clinical Practice Directive 2005/28/EC and Clinical Trial Directive 2001/20/EC. All suspected unexpected serious adverse reactions (SUSARs) will be reported, as required, to the competent authorities of all involved European member states.

9.5.5 Completion/Discontinuation of Subjects

A subject may elect to discontinue the study at any time for any reason. Subjects who discontinue the study or study drug early must comply with the Early Termination Visit (within 7 days after the last dose of study drug) and the Follow-up Visit (3-months after the last dose of study drug). They may also have unscheduled visits for safety assessments. Subjects who discontinued the Core Study due to APOE status are expected to return for their Early Termination Visit and 3-month Follow-Up Visit (ie, 3 months after the last dose of study drug). In addition, subjects who discontinue from study drug are expected to return after the Early Termination Visit for each scheduled visit when the clinical assessments of efficacy are to be conducted (Visits 9, 16, 22, 29, 35, and 42) per the Schedule of Procedures/Assessments (Table 8). At these visits, clinical efficacy assessments (MMSE, CDR, ADAS-Cog, and FAQ) will be completed and information on concomitant medications, AEs, and SAEs will be collected from these subjects. Regularly scheduled clinical efficacy visits do not need to be attended if they fall within 8 days after the Early Termination Visit or within +/- 8 days of the 3-month Follow-Up Visit. Subjects who complete the Core Study may elect to continue into the Extension Phase if the criterion is met (see Appendix 4). Subjects who discontinue study drug in the Extension Phase will not be required to return after the Early Termination Visit for each scheduled visit when clinical efficacy assessments are conducted. Subjects in the Extension Phase who discontinue the study or study drug early must comply with the Early Termination Visit (within 7 days after the last dose of study drug) and the Follow-up Visit (3 months after the last dose of study drug). (revised per Amendments 08 and 11)

Subjects who discontinue from study drug are considered on study as long as they return for their regularly scheduled clinical efficacy visits as outlined above.

Subjects who discontinue due to AE will not be allowed to rescreen for the BAN2401-G000-201 study. Subjects who discontinue due to ARIA will be unblinded by the sponsor. (revised per Amendments 06 and 11)

The investigator will promptly explain to the subject involved that the study will be discontinued for that subject and provide appropriate medical treatment and other necessary measures for the subject. A subject who has ceased to return for visits will be followed up by mail, phone, or other means to gather information such as the reason for failure to return, the status of treatment compliance, the presence or absence of AEs, and clinical courses of signs and symptoms. This information will be recorded in the eCRF.

Subjects who discontinue early from the study will be discontinued for 1 of these primary reasons: AE(s), lost to follow-up, subject choice, withdrawal of consent, pregnancy, study terminated by sponsor, or other. In addition to the primary reason, the subject may indicate 1 or more of secondary reasons for discontinuation. Study disposition information will be collected on the Subject Disposition eCRF.

A subject removed from the study for any reason may not be replaced.

9.5.6 Abuse or Diversion of Study Drug

Not applicable. The study drug is to be administered by study site personnel.

9.5.7 Confirmation of Medical Care by Another Physician

The investigator will instruct subjects to inform site personnel when they are planning to receive medical care by another physician. At each visit, the investigator will ask the subject whether he/she has received medical care by another physician since the last visit or is planning to do so in the future. When the subject is going to receive medical care by another physician, the investigator, with the consent of the subject, will inform the other physician that the subject is participating in the clinical study.

9.6 Data Quality Assurance

This study will be organized, performed, and reported in compliance with the protocol, standard operating procedures, working practice documents, and applicable regulations and guidelines. Site audits will be conducted periodically by the sponsor's or the CRO's qualified compliance auditing team, which is an independent function from the study team responsible for conduct of the study.

9.6.1 Data Collection

Data required by the protocol will be collected on the eCRFs, which constitute into a validated data management system that is compliant with all regulatory requirements. As defined by ICH guidelines, the eCRF is a printed, optical, or electronic document designed to record all of the protocol-required information to be reported to the sponsor on each study subject.

Data collection on the eCRF must follow the instructions described in the CRF Completion Guidelines. The investigator has ultimate responsibility for the collection and reporting of all

clinical data entered on the eCRF. The investigator or designee as identified on Form FDA 1572 (where applicable) must sign the completed eCRF to attest to its accuracy, authenticity, and completeness.

Completed eCRF case books are the sole property of Eisai and should not be made available in any form to third parties without written permission from Eisai, except for authorized representatives of Eisai or appropriate regulatory authorities.

9.6.2 Clinical Data Management

All software applications used in the collection of data will be properly validated following standard computer system validation that is compliant with all regulatory requirements. All data, both CRF and external data (eg, laboratory data), will be entered into a clinical system.

9.7 Statistical Methods

All statistical analyses will be performed by the sponsor or designee after the database is locked and released for unblinding. (revised per Amendment 07) At each IA of the primary endpoint, a snapshot of the data will obtained and released for analysis. A copy of this snapshot will be archived. This process will also be followed for the safety data that will be used for analysis for the DSMB. Statistical analyses will be performed using SAS software or other validated statistical software as required. All statistical tests will be based on the 10% level of significance, except for the Bayesian methods on the primary endpoint . (revised per Amendments 09 and 10) Details of the statistical analyses will be included in a separate statistical analysis plan (SAP).

9.7.1 Statistical and Analytical Plans

The statistical analyses of the BAN2401-G000-201 study data are described in this section. Further details of the analysis plan will be provided in the SAP, which will be finalized before the first IA.

In this Phase 2 dose-finding study of BAN2401 for the treatment of Early Alzheimer's disease, response adaptive randomization will be used to allocate subjects to placebo control or 1 of the 5 active doses with the goal of characterizing the dose response. The study will be monitored for early success and early futility. The sponsor intends that the futility rule for stopping will be binding. If the study continues to randomize subjects after futility is declared, it will be considered a failed study. (revised per Amendment 01)

9.7.1.1 Study Endpoints

Primary Endpoint:

- Change from baseline in the ADCOMS at 12 months

Key Secondary Endpoints: (revised per Amendments 09 and 10)

- Change from baseline at 18 months in brain amyloid pathophysiology as measured by amyloid PET (revised per Amendments 09 and 10)

- Change from baseline in the ADCOMS at 18 months (revised per Amendment 09)
- Change from baseline in CDR-SB at 18 months (revised per Amendments 09 and 10)
- Change from baseline in ADAS-Cog at 18 months (revised per Amendments 09 and 10)
- Change from baseline in CSF biomarkers (Aβ[1-42], t-tau, and p-tau) at 18 months (revised per Amendment 10)
- Change from baseline in total hippocampal volume at 18 months using vMRI (revised per Amendments 09 and 10)

SECONDARY ENDPOINTS: (revised per Amendments 09 and 10)

- Change from baseline at 12 months in brain amyloid pathophysiology as measured by amyloid PET (revised per Amendments 09 and 10)
- Change from baseline at 12 months on clinical status for the following assessments: ADCOMS, CDR-SB, and ADAS-Cog (revised per Amendment 10)
- Change from baseline in CSF biomarkers (Aβ[1-42], t-tau, and p-tau) at 12 months (revised per Amendment 10)
- Change from baseline in total hippocampal at 6 and 12 months, and in left and right hippocampus, whole brain, and total ventricular volume as measured by vMRI at 6, 12, and 18 months (revised per Amendment 10)

EXPLORATORY ENDPOINTS:

- Change from baseline in clinical status at time points not analyzed in Key Secondary and Secondary sections for each of the following: ADCOMS, ADAS-Cog, CDR-SB, MMSE, and FAQ (revised per Amendment 10)

9.7.1.2 Definitions of Analysis Sets

The Randomized Set is the group of subjects who are randomized to study drug.

The Safety Analysis Set is the group of subjects who receive at least 1 dose of study drug and have at least 1 postdose safety assessment.

The Full Analysis Set is the group of randomized subjects who receive at least 1 dose of study drug and have baseline and at least 1 postdose primary efficacy measurement.

The PK Analysis Set is the group of subjects with at least 1 quantifiable BAN2401 serum concentration with a documented dosing history.

The PD Analysis Set is the group of subjects who have sufficient PD data to derive at least 1 PD parameter.

The Per Protocol (PP) Analysis Set is the subset of subjects in the Full Analysis Set who sufficiently complied with the protocol. The Per Protocol Analysis Set will be used to conduct a

sensitivity analysis of the primary efficacy endpoint. Details of the evaluability criteria will be determined before database lock and treatment unblinding and will be specified in the SAP.

9.7.1.3 Subject Disposition

The number of subjects screened, the number (percent) of subjects who failed screening, and the reasons for screen failure will be summarized, based on data reported on the Screening Disposition eCRF. The distribution of the number of randomized subjects enrolled by each site will be summarized for each randomized treatment group. The primary reasons for screen failures; did not meet inclusion/exclusion criteria, AE, lost to follow-up, withdrawal consent, and other, will be presented.

Study Completion: the number (percent) of randomized and treated subjects who completed the study and who discontinued from the study will be summarized according to the primary reason for discontinuation and secondary reason(s) for discontinuation, based on data reported on the Subject Disposition (Study Phase) CRF. The number (percent) will be presented by treatment group and total for subjects; randomized, not treated, treated, who completed the study, and discontinued from the study. The primary reasons for discontinuation from the study are; AE, lost to follow-up, subject choice, withdrawal consent, pregnancy, study terminated by sponsor, and other. The secondary reasons for discontinuation from the study are: AE, subject choice, and other.

Completion of Study Treatment: the number (percent) of randomized and treated subjects who completed study treatment and who discontinued from study treatment will be summarized according to the primary reason for discontinuation and also according to secondary reason(s) for discontinuation, based on data reported on both the Subject Disposition (Study Phase) eCRF and Early Discontinuation from Study Drug eCRF. The number (percent) will be presented by treatment group and total for subjects; randomized, not treated, treated, who completed study treatment, and discontinued from study treatment. The primary reasons for discontinuation from the study treatment are: AE, lost to follow-up, subject choice, withdrawal of consent, pregnancy, study terminated by sponsor, inadequate therapeutic response, and other (revised per Amendment 01). The secondary reasons for discontinuation from the study are: AE, subject choice, and other.

9.7.1.4 Demographic and Other Baseline Characteristics

Demographic and baseline characteristics for the Safety Analysis set and Full Analysis set will be summarized for each treatment group using descriptive statistics. Continuous demographic and baseline variables include age, WMS-IV LMII, Geriatric Depression scale, MMSE; categorical variables include age ≤65 years, age >65 years, sex, race, ethnicity, CDR score, memory box score of the CDR, positive amyloid load, *APOE4* genotype and clinical subgroup (MCI due to AD – intermediate likelihood or mild Alzheimer’s disease dementia).

9.7.1.5 Prior and Concomitant Therapy

All investigator terms for medications recorded in the eCRF will be coded to an 11-digit code using the World Health Organization Drug Dictionary. The number (percentage) of subjects

who took prior and concomitant medications will be summarized on the Safety Analysis Set by treatment group, Anatomical Therapeutic Chemical (ATC), and the World Health Organization Drug Dictionary preferred term. Prior medications are defined as medications that stopped before the first dose of study drug. Concomitant medications are defined as medications that (1) started before the first dose of study drug and were continuing at the time of the first dose of study drug, or (2) started on or after the date of the first dose of study drug and continued up to the last dose.

9.7.1.6 Efficacy Analyses

ADAPTIVE TRIAL DEFINITIONS

Clinically Significant Difference (CSD): The CSD used in the adaptive model relative to the change from baseline over time for placebo in the ADCOMS. The CSD for this study represents an approximate 25% reduction compared to placebo in the progression of AD as measured by the ADCOMS.

Maximum Effective Dose (d_{Max}): The dose that achieves the greatest treatment effect

ED_{90}: The 'simplest' dose regimen that achieves at least 90% of the d_{Max} treatment effect. The term 'simplest' reflects the preference for dosing frequency and amount when considering the treatment arms for the study and while taking efficacy into account (monthly administration is preferred over biweekly administration and lower doses are preferred over higher doses, with priority placed on frequency of administration). Thus, the rank order of prioritized doses (beginning with the simplest) is: 5 mg/kg Q4wk, 10 mg/kg Q4wk, 2.5 mg/kg Q2wk, 5 mg/kg Q2wk, and 10 mg/kg Q2wk.

Early Success: The study meets early success criteria if the probability that the ED_{90} is better than the placebo by the CSD is at least 0.95. Early success is achieved before all subjects complete 12 months of treatment and can only be determined once ≥350 subjects are randomized. If early success is declared prior to full enrollment, randomization of new subjects will stop, outside Japan. Sites in Japan will randomize up to 40 Japanese subjects even if early success is declared or the study achieves 800 subjects randomized. All randomized Japanese subjects in the study will receive up to 18 months of treatment in the Core Study and are eligible to participate in the Extension Phase if implemented. (revised per Amendments 07, 08, and 11)

Futility: The study meets statistical futility criteria if the probability that the ED_{90} is better than the placebo by the CSD is less than 0.05 (with ≤300 subjects randomized) or 0.075 (with ≥350 subjects randomized) at 12 months of treatment. If the study meets statistical futility criteria, the sponsor will make the final decision pertaining to study futility after reviewing recommendations by the DSMB. Based on the sponsor's final decision, if the study continues after statistical futility criteria are met, it will be considered a failed study. (revised per Amendment 01)

The study will be considered a success if either of the following criteria are met:

1. The study meets early success criteria

2. The study reaches complete randomization and the Bayesian analysis at completion of 12 months of treatment results in an ED_{90} with at least an 80% probability of being better than the placebo by the CSD (revised per Amendment 09)

ANALYSIS OF PRIMARY EFFICACY ENDPOINT (REVISED PER AMENDMENT 09)

The primary analysis will be based on subjects from the Full Analysis Set with the prespecified censoring rules applied. Japanese subjects who are randomized after early success is declared or the study achieves 800 subjects randomized will not be included in the primary analysis. (revised per Amendment 07) The primary endpoint is the change from baseline to 12 months in the ADCOMS. The ADCOMS is a weighted linear combination of 12 items from the 3 existing clinical scales, the ADAS-Cog, the MMSE, and the CDR. These 12 items consist of the predictive variables A4, A7, A8, A11, M1, M7, C1, C2, C3, C4, C5, and C6, which have been selected from the clinical scales, the ADAS-Cog, the MMSE, and the CDR. The names of these item and the corresponding scale names are described in Table 10.

Table 10 Predictive Variables for the ADCOMS

Scale	Item ID	Item Name	PLS weight
ADAS-Cog	A4	Delayed Word Recall	0.00847483
	A7	Orientation	0.017088
	A8	Word Recognition	0.003732761
	A11	Word Finding	0.016211
MMSE	M1	Orientation Time	0.041567
	M7	Drawing	0.038238
CDR	C1	Personal Care	0.054321
	C2	Community Affairs	0.1091
	C3	Home and Hobbies	0.089039
	C4	Judgment and Problem Solving	0.069493
	C5	Memory	0.058724
	C6	Orientation	0.078152

ADAS-Cog = Alzheimer's Disease Assessment Scale, cognitive subscale, CDR = Clinical Dementia Rating, ID = identification, MMSE = Mini Mental State Examination, PLS = Partial Least Squares.

The maximum ADCOMS is achieved when each item is assigned the maximum score. This maximum composite score is 1.97. The range of this new composite score is therefore between 0 and 1.97.

The primary endpoint is the change from baseline to 12 months in the ADCOMS. The dose response of the primary endpoint is modeled with a 2-dimensional first-order normal dynamic linear model, where Normal and Inverse-Gamma priors are used. The primary efficacy analysis will calculate the Bayesian posterior probability that the dose identified is the most likely ED90 dose that achieves the CSD compared to the placebo arm. At each Bayesian analysis, 3 Bayesian

probabilities will be summarized for each active dose: the probability of being the ED_{90} dose, the probability of being statistically superior to placebo, and the probability of being statistically superior to placebo by the CSD. The study will be considered a success in the Bayesian analysis at the completion of 12 months of treatment if there is at least an 80% probability that the ED90 achieves the clinically significant difference from placebo. (revised per Amendment 09)

For the clinical efficacy data (ie, derived Clinical Composite Score, MMSE, CDR-SB, ADAS-Cog, and FAQ), subjects will be censored at the time of initiation of new AChEIs or memantine treatment regimens if they were not on AChEIs or memantine at randomization, and will be censored at the time of dose adjustment of AChEIs or memantine if they were already on stable treatment with AChEIs or memantine at randomization. The value of the primary endpoint for censored subjects will be imputed using data up to the censoring time and the Bayesian imputation method.

After unblinding, the primary endpoint will be analyzed using the Bayesian methods described above as well as conventional statistical methods. The following additional Bayesian analyses will be conducted as sensitivity analyses: (revised per Amendment 01)

- The primary endpoint will be analyzed regardless of initiation of new AChEIs or memantine or dose adjustment of stable AChEIs or memantine, using the same Bayesian analysis method as in the primary analysis. (revised per Amendment 01)
- In case of early success, the primary analysis as well as the above sensitivity analyses will be repeated after all subjects have either completed 12 months follow up or are lost to follow up. (revised per Amendment 01)

Statistical methods for the conventional analyses of primary efficacy endpoint will use a mixed effects model with repeated measures (MMRM) comparing placebo to the identified ED_{90} dose from the Bayesian analysis (revised per Amendment 09). The model will include randomization of stratification variables, clinical subgroup (MCI due to AD, mild AD), the presence or absence of ongoing AD treatment (ie, AChEIs or memantine or both), *APOE4* status (positive, negative), and baseline Composite Clinical Score as covariates. These analyses will be performed on the full analysis set as well as the per protocol set based on the ITT principle. In case of early success, the conventional analyses will also be performed using the data collected prior to the IA. (revised per Amendment 01)

ANALYSIS OF KEY SECONDARY EFFICACY ENDPOINTS (REVISED PER AMENDMENTS 09 AND 10)

Key Secondary endpoints were ordered to reflect the most recent FDA Draft Guidance and EMA Guideline that prioritizes effects on pathophysiology in early AD, as measured by biomarkers (February, 2018). Conventional analysis will be performed using all available data at the time of success (if applicable), and at 18 months of treatment, regardless of success. An MMRM will be used to compare the combined 10 mg dose group (including biweekly and monthly regimens) with placebo for the key secondary endpoints. (revised per Amendments 09 and 10) The rationale for combining the two 10-mg dose regimens is to account for the loss of subjects positive for *APOE4* in the 10 mg/kg biweekly dose group and the inability to randomize *APOE4* carriers to the 10 mg/kg biweekly group following a Regulatory request by European Health

Authorities in July, 2014. The key Secondary endpoint based on clinical status (ADCOMS, CDR-SB, and ADAS-Cog) will be analyzed in subjects with EAD as well as separately within subjects with MCI and mild AD dementia. (revised per Amendment 10)

In addition, as sensitivity analysis, MMRM analysis will be performed for the following treatment comparisons: (revised per Amendment 10)

- Combining 2 high doses (10 mg biweekly + 10 mg monthly), 2 middle doses (5 mg biweekly + 5 mg monthly), and the 2.5 mg biweekly dose, resulting in 3 treatment comparisons with placebo (for dose response) (revised per Amendment 10)
- ED_{90} dose of BAN2401 versus placebo, where the ED_{90} dose of BAN2401 will be established by Bayesian analysis at 18 months as described in the next paragraph (revised per Amendment 10)
- By treatment regimen (10 mg biweekly, 10 mg monthly, 5 mg biweekly, 5 mg monthly, 2.5 mg biweekly, placebo) (revised per Amendment 10)
- Combining the top 3 doses (10 mg biweekly, 10 mg monthly, and 5 mg biweekly) versus placebo (revised per Amendment 10)

Change from baseline in the ADCOMS at 18 months will be analyzed, as a sensitivity analysis to the conventional analysis, using the same Bayesian methodology as that for analysis of change from baseline in the ADCOMS at 12 months, but using the full 18 months of efficacy data with the model projecting to 18 months of treatment. The Bayesian analysis of change from baseline in the ADCOMS at 18 months is positive if the analysis results in an ED_{90} dose with at least an 80% probability of being better than the placebo by the CSD. The ED_{90} dose will be identified based on change from baseline in the ADCOMS at the 18 month Bayesian analysis and will be used to compare ED_{90} dose of BAN2401 and placebo in the above sensitivity analyses for all key secondary endpoint analyses. (revised per Amendments 09 and 10)

ANALYSIS FOR OTHER SECONDARY EFFICACY ENDPOINTS

The same analysis described above for key secondary endpoints will be performed for other secondary endpoints. (revised per Amendment 10)

EXPLORATORY EFFICACY ANALYSES

The same analysis described above for key secondary endpoints will be performed for exploratory endpoints. (revised per Amendment 10)

The effect of baseline brain amyloid pathopohysiology as measured by amyloid PET on change from baseline in clinical status at 12 and 18 months of treatment will be explored using correlation analysis and evaluated using statistical models as appropriate. (revised per Amendment 10)

The relationship between change from baseline in clinical status and change from baseline in brain amyloid pathopohysiology at 12 and 18 months of treatment as measured by amyloid PET will be explored by correlation analysis. (revised per Amendment 10)

Change in ADCOMS and its components, ADAS-Cog, CDR-SB and MMSE across all clinical assessment time points between BAN2401 and placebo in Japanese subjects will be evaluated using the descriptive statistics within Japanese subjects as well as with the overall population. (revised per Amendments 07 and 10)

9.7.1.7 Pharmacokinetic, Pharmacodynamic, and Pharmacogenomic/Pharmacogenetic Analyses

PHARMACOKINETIC ANALYSES

The Safety Analysis Set will be used for individual BAN2401 serum and CSF concentration listings. The PK Analysis Set will be used for summaries of BAN2401 serum and CSF concentrations.

A population PK approach will be used to characterize the PK of BAN2401. The effect of covariates (ie, demographics) on BAN2401 PK will be evaluated. The PK model will be parameterized for clearance (CL) and volumes of distribution. Derived exposure parameters such as AUC will be calculated from the model using the individual posterior estimate of CL and dosing history.

PHARMACODYNAMIC ANALYSES

The PD Analysis Set will be used for the summaries and analyses of CSF biomarkers. CSF Aβ(1-42), t-tau, and p-tau will be assessed and data presented graphically. Baseline levels and changes in t-tau and p-tau will be correlated with changes in CSF Aβ(1-42), imaging markers, and *APOE4* status. Summary statistics for CSF biomarkers will be assessed for evidence of a dose relationship.

PHARMACOKINETIC-PHARMACODYNAMIC ANALYSES

The PK/PD relationship between CSF biomarker levels and serum PK parameters or CSF concentrations of BAN2401 will be explored graphically and any emergent relationship will be explored through population PK/PD modeling. These serum PK parameters include SS, Cmax and AUC(0-t) derived from the population PK model. The PK/PD relationship between serum PK parameters and CSF concentrations of BAN2401 with other biomarkers may also be explored using similar methods.

Additionally, the relationship between various serum PK parameters (eg, C_{max}) or CSF concentrations of BAN2401 (Core Study only) and the ADCOMS at 12, 18, 30, and 42 months, and the relationship between various serum PK exposure parameters or CSF concentrations of BAN2401 and the change from Baseline for 12, 18, 30, and 42 months in the ADAS-Cog, the CDR-SB, and the MMSE, will be explored graphically (revised per Amendments 08 and 11). Any emergent relationships will be explored through population PK/PD modeling.

The relationship between exposure to BAN2401 and most frequent AEs will also be explored.

PHARMACOGENOMIC/PHARMACOGENETIC ANALYSES

APOE4 genotypes or other important genomic/genetic findings will be used in the statistical analysis to determine the effects on treatment response and safety.

Exploratory pharmacogenomics and biomarker analyses may be performed and reported separately. Details of these analyses may be described in a separate analysis plan. (revised per Amendment 01)

9.7.1.8 Safety Analyses

Evaluations of safety will be performed on the Safety Analysis Set. The incidence of AEs (including changes from baseline in physical examination), out-of-normal-range laboratory safety test variables, abnormal ECG findings, anti-BAN2401 antibody titers, and out-of-range vital signs, suicidality (C-SSRS), along with change from baseline in laboratory safety test variables, ECGs, safety MRI, and vital sign measurements, will be summarized by treatment group using descriptive statistics. The safety analysis will be also be summarized by the following safety subgroups: age (≤65 years, >65 years), sex (male, female), race (white, black, Asian, other), *APOE4* status, and clinical subgroup (MCI due to AD – intermediate likelihood or mild Alzheimer's disease dementia). (revised per Amendment 07)

EXTENT OF EXPOSURE

Extent of exposure to study drug will be summarized as cumulative extent of exposure from Day 1 to Week 6 initially, and at 3 months intervals thereafter. The number and percent of subjects will be presented by treatment group. Duration of exposure will be summarized using descriptive statistics and by duration categories; Day 1 to 6 weeks, 6 weeks to 3 months, and 3-month intervals thereafter. The number and percent of subjects will be presented by each duration category and treatment group.

ADVERSE EVENTS

The AE verbatim descriptions (investigator terms from the eCRF) will be classified into standardized medical terminology using the Medical Dictionary for Regulatory Activities (MedDRA). Adverse events will be coded to the MedDRA (Version 15.0 or higher) lower level term (LLT) closest to the verbatim term. The linked MedDRA preferred term (PT) and primary system organ class (SOC) will also be captured in the database.

A treatment-emergent AE (TEAE) is defined as an AE that emerged during treatment or any time prior to the last study visit following the last dose of study drug, having been absent at pretreatment (Baseline) or:

- Reemerged during treatment, having been present at pretreatment (Baseline) but stopped before treatment, or
- Worsened in severity during treatment relative to the pretreatment state, when the AE was continuous.

TEAEs and AEs will be followed for the entire duration of the study. Only those AEs that are treatment emergent will be included in summary tables. All AEs, treatment emergent or otherwise, will be presented in subject data listings.

Treatment-emergent AEs (TEAEs) will be summarized by treatment group on the Safety Analysis Set. The incidence of TEAEs will be reported as the number (percentage) of subjects with TEAEs by SOC and PT. A subject will be counted only once within a SOC and PT, even if the subject experienced more than 1 TEAE within a specific SOC and PT. The number (percentage) of subjects with TEAEs will also be summarized by maximum severity (mild, moderate, or severe). The number (percentage) of subjects with TEAEs will also be summarized by relationship to study drug (possibly related, probably related, and not related).

Adverse events will be summarized by the following subgroups: age (≤65 years, >65 years), sex (male, female), race (white, black, other), clinical subgroup (MCI due to AD – intermediate likelihood and mild Alzheimer's disease dementia), the presence or absence of ongoing AD treatment (ie, AChEIs or memantine or both), and *APOE4* genotype.

The number (percentage) of subjects with TEAEs leading to death will be summarized by MedDRA SOC and PT for each treatment group. A subject data listing of all AEs leading to death will be provided.

The number (percentage) of subjects with treatment-emergent SAEs will be summarized by MedDRA SOC and PT for each treatment. A subject data listing of all SAEs will be provided.

The number (percentage) of subjects with TEAEs leading to discontinuation from study drug will be summarized by MedDRA SOC and PT for each treatment. A subject data listing of all AEs leading to discontinuation from study drug will be provided.

ANTI-DRUG (BAN2401) ANTIBODIES

The number (percentage) of subjects with positive and negative ADA, ADA titer categories (>0, 5, 25, 125, etc.), and neutralizing antibodies by visit and treatment group will be summarized (revised per Amendment 08). In addition, the correlation between ADA titer and PK profile or the correlation between ADA titer and efficacy will be evaluated using descriptive statistics and summary plots.

LABORATORY VALUES

Laboratory results will be summarized using Système International (SI) units, as appropriate. For all quantitative parameters listed in Section 9.5.1.5. Safety Assessments (Laboratory Measurements), the actual value and the change from baseline to each postbaseline visit and to the end of treatment (defined as the last on-treatment value) will be summarized by visit and treatment group using descriptive statistics. Qualitative parameters listed in Section 9.5.1.5 will be summarized using frequencies (number and percentage of subjects), and changes from baseline to each postbaseline visit and to end of treatment will be reported using shift tables. Percentages will be based on the number of subjects with both non-missing baseline and relevant post-baseline results.

Laboratory test results will be assigned a low/normal/high (LNH) classification according to whether the value was below (L), within (N), or above (H) the laboratory parameter's reference range. Within-treatment comparisons for each laboratory parameter will be based on 3-by-3 tables (shift tables) that compare the baseline LNH classification to the LNH

classification at each postbaseline visit and at the end of treatment. Similar shift tables will be used to compare the baseline LNH classification to the LNH classification for the highest and lowest value during the treatment period.

The Sponsor's Grading for Laboratory Values (Appendix 1) presents the criteria that will be used to identify subjects with treatment-emergent markedly abnormal laboratory values (TEMAV). Except for phosphate, a TEMAV is defined as a post-baseline value with an increase from baseline to a grade of 2 or higher. For phosphate, a TEMAV is defined as a postbaseline value with an increase from baseline to a grade of 3 or higher. When displaying the incidence of TEMAVs, each subject will be counted once in the laboratory parameter high and in the laboratory parameter low categories, as applicable.

VITAL SIGNS

Descriptive statistics for vital signs parameters (diastolic and systolic blood pressure, pulse, respiration rate, temperature, weight and changes from baseline) will be presented by visit and treatment group.

ELECTROCARDIOGRAMS

(revised per Amendment 01)

Abnormal ECG findings will be presented as shifts from baseline (normal/abnormal clinically significant/abnormal not clinically significant) to postbaseline (normal/abnormal clinically significant/abnormal not clinically significant) visits. The number and percent of subjects will be presented.

9.7.1.9 Determination of Sample Size

The sample size and design characteristics to test the hypothesis under the primary objective based on the primary endpoint, the Composite Clinical score at 12 months, were determined by means of simulations. Extensive trial simulations have shown that a total of 800 subjects will be sufficient to demonstrate that the most likely ED_{90} dose achieves a clinically significant difference (CSD) from placebo with a probability of at least 95% in the interim analyses and greater than 80% in the final analysis if the trial does not stop for early success. There is at least 80% probability of study success for the dose response scenarios where the treatment was considered to have clinically meaningful treatment effect. Simulations have also shown that if there is no efficacy at all for any dose, then the probability of falsely claiming superiority to placebo is no more than 10.0% assuming a 20% dropout rate.

For each of the 6 treatment groups (5 active dose regimens and placebo) the final number of subjects per group will differ depending on the observed interim treatment responses. The simulation plan will be described in the appendix section of the Statistical Analysis Plan, which will present further details.

vMRI

The null hypothesis is that there is no difference between active dose and placebo. There are 5 null hypotheses corresponding to 5 active dose regimens. The alternative hypothesis is that at

least 1 null hypothesis is false (1-sided). The null hypotheses will be tested using Dunnett method with 1-sided alpha of 0.05. The statistical power was estimated through simulation for a moderate dose-response assumption that the percent reduction in change from baseline of total hippocampal volume compared to placebo would be 15%, 20%, 25%, 15%, and 20% corresponding to the 5 dose regimens (2.5, 5, and 10 mg/kg biweekly, 5 and 10 mg/kg 4-week interval), respectively. The estimated power for actual planned study sample size is 0.764 at 12 months and 0.694 at 18 months, assuming an attrition rate of 20% at 12 months and an exponential dropout model. Under a stronger dose-response assumption (ie, the percent reduction in change from baseline of total hippocampal volume compared to placebo is twice as much as that in the moderate dose-response assumption), the estimated power is at least 99% at both 12 and 18 months.

AMYLOID PET (IMAGING SUBGROUP)

The null and alternative hypotheses and statistical test are the same as that for vMRI. To estimate the sample size for amyloid PET imaging subgroup at 18 months, a common standard deviation of change from baseline was estimated as 0.4 and a reasonable mean difference between treatment and placebo was estimated as 0.25 (SUVR). The corresponding standard deviation and mean difference at 12 months were estimated as 0.27 and 0.17, respectively. Assuming a moderate dose-response that the best dose regimen would achieve a difference of 0.25 (SUVR), and that the 2 middle and 2 low dose regimens would achieve a difference of 0.2 and 0.15 (SUVR), respectively, it would require a total 230 subjects from all 6 arms at 18 months to achieve an 80% power. This sample size took into account possible unequal number of subjects per arm. Under the same attrition rate assumption as that for vMRI, a total sample size of 306 at 12 months is sufficient to obtain 230 subjects at 18 months. The estimated power with 306 subjects at 12 months is approximately 85%. The postbaseline amyloid PET in the imaging subgroup will be planned for the first 306 subjects who have consented and who are still on treatment at 12 months. (revised per Amendments 02 and 07)

9.7.2 Interim Analysis

An unblinded independent Interim Monitoring Committee (IMC) will provide oversight to ensure that the response adaptive randomization process and interim analyses perform as expected. An independent data analysis group will perform all of the interim analyses and will provide the results to the IMC.

Randomization to placebo or 1 of 5 dose arms of BAN2401 will be fixed for the first 196 subjects randomized in the study (4:2:2:2:2:2; placebo [4] to each of the active arms [2 each]). After this initial burn-in, adaptive randomization will begin. Randomization probabilities to each arm will be updated at each interim analyses such that the randomization probability will be increased for the placebo arm and arms that represent the potential target dose (ED_{90}), and simultaneously decreased for other active arms. Subjects who are confirmed *APOE4* positive (*APOE4* hetero- or homozygous) will not be randomized to the 10 mg/kg, biweekly dose. (revised per Amendments 04 and 05). After each IA, the randomization probability vector will be split between *APOE4*+ and *APOE4*- strata to ensure no *APOE4* positive subjects are enrolled on the 10 mg/kg biweekly dose. The overall probabilities as suggested by RAR will

still be preserved. (revised per Amendments 05 and 07) Details of the estimation of the randomization probabilities will be presented in the Statistical Analysis Plan.

The study will be monitored for early success and early futility (see definitions in Study Definitions). The first IA will be conducted when 196 subjects are randomized, again when 250 subjects are randomized and again after each additional 50 subjects until 800 subjects are randomized. Sites in Japan will randomize at least 40 Japanese subjects, even if early success is declared or the study achieves 800 subjects randomized. In these circumstances, subjects in Japan will be allocated treatment according to the latest (or last) allocation schedule. If the study reaches its maximum of 800 randomized subjects, additional interim analyses will be conducted every 3 months until all subjects complete 12 months of treatment. Only those subjects who were randomized before the target of 800 was met will contribute to these additional interim analyses, except for the full 12 and 18 month Bayesian analyses. The study will be monitored for futility at each IA and for success starting at the 350 subject IA, until the full 12 months of treatment have been completed. (revised per Amendment 09) Each IA will be conducted on data for the derived Clinical Composite Score. If the efficacy or futility boundary is reached, the IMC will communicate directly with the DSMB members. (revised per Amendment 07)

The study may be stopped for safety reasons at any time.

9.7.3 Other Statistical/Analytical Issues

Not applicable

9.7.4 Procedure for Revising the Statistical Analysis Plan

If the SAP needs to be revised after the study starts, the sponsor will determine how the revision impacts the study and how the revision should be implemented. The details of the revision will be documented and described in the clinical study report.

10 REFERENCE LIST

1. Brookmeyer R, Johnson E, Ziegler-Graham K, Arrighi HM. Forecasting the global burden of Alzheimer's Disease. Alzheimer Dement. 2007;3:186-91.
2. Alzheimer's Association. Alzheimer's Association report. 2010 Alzheimer's disease facts and figures Alzheimer Dement. 2010;6:158-94.
3. Hebert LE, Scherr PA, Bienias JL, Bennett DA, Evans DA. Alzheimer disease in the U.S. population: prevalence estimates using the 2000 census. Arch Neurol. 2003;60:1119-1122.
4. Doraiswamy PM, Leon J, Cummings JL, Marin D, Neumann PJ. Prevalence and impact of medical comorbidity in Alzheimer's disease. J Gerontol A Biol Sci Med Sci. 2002;57(3):M173-M177.
5. Kalaria RN, Ballard C. Overlap between pathology of Alzheimer disease and vascular dementia. Alzheimer Dis Assoc Disord. 1999;13 Suppl 3:S115-23.
6. Dodort J-C and May P. Overview on rodent models of Alzheimer's disease. Curr Protocols Neurosci. 2005;9.22-1-9.22-6.
7. Englund H, Sehlin D, Johansson AS, Nilsson LN, Gellerfors P, Paulie S, et al. Sensitive ELISA detection of amyloid-β protofibrils in biological samples. J Neurochem. 2007;103:334-45.
8. Gotz J, Streffer JR, David D, Schild A, Hoerndli F, Pennanen L, et al. Transgenic animal models of Alzheimer's disease and related disorders: histopathology, behavior and therapy. Mol Psychiat. 2004;9:664-83.
9. Broich K, Weiergraber M, Hampel H. Biomarkers in clinical trials for neurodegenerative diseases: Regulatory perspectives and requirements. Prog Neurobiol. 2011;95:498-500.
10. Hampel H, Frank R, Broich K, Teipel SJ, Katz R, Hardy J, et al. Biomarkers for Alzheimer's disease: academic, industry and regulatory perspectives. Nat Rev Drug Discov. 2010;9:560-74.
11. Albert MS, DeKosky ST, Dickson D, Dubois B, Feldman HH, Fox NC, et al. The diagnosis of mild cognitive impairment due to Alzheimer's disease: recommendations from the National Institute on Aging-Alzheimer's Association workgroups on diagnostic guidelines for Alzheimer's disease. Alzheimer Dement. May;7(3):270-9. Epub 2011 Apr 21.
12. Aisen, PS, Andrieu S, Sampaio C, Carrillo M, Khachaturian ZS, Dubois B et al. Report of the task force on designing clinical trials in early (predementia) AD. Neurology. 2011;76:280–286.
13. McKahnn GM, Knopman DS, Chertkow H, Hyman BT, Jack CR, Jr., Kawas CH, et al. The diagnosis of dementia due to Alzheimer's disease: Recommendations from the National Institute on Aging – Alzheimer's Association workgroups on diagnostic guidelines for Alzheimer's disease. Ahzheimer Dement. 2011;7:263-9.
14. Aizenstein HJ, Nebes RD, Saxton JA, Price JC, Mathis CA, Tsopelas ND, et al. Frequent amyloid deposition without significant cognitive impairment among the elderly. Arch Neurol. 2008;65:1509–17.

15. Engler H, Forsberg A, Almkvist O, Blomquist G, Larsson E, Savitcheva I, et al. Two-year follow-up of amyloid deposition in patients with Alzheimer's disease. Brain. 2006;129:2856–66.

16. Mathis CA, Price JC, Klunk WE, Weissfield L, McNamee R, Wolk DA, et al. Longitudinal PIB measures in control, MCI, and AD subjects. J Nucl Med. 2008;49:35P.

17. Villemagne V, Pike K, Chetelat G, Ellis KA, Mulligan RS, Bourgeat P, et al. Longitudinal Assessment of Abeta and cognition in Aging and Alzheimer Disease. Ann Neurol. 2011;69:181-192.

18. Drzezga A, Grimmer T, Henriksen G, Muhlau M, Perneczky R, Miederer I, et al. Effect of APOE genotype on amyloid plaque load and gray matter volume in Alzheimer disease. Neurology. 2009;72:1487-94.

19. Moromino EC, Kluth JT, Madison CM, Rabinovici GD, Baker SL, Miller BL, et al. Episodic memory loss is related to hippocampal-mediated beta amyloid deposition in elderly subjects. Brain. 2009;132:1310-23.

20. Vandenberghe R, Van Laere K, Ivanoiu A, Salmon E, Bastin C, Triau E et al. 18F-flutemetamol amyloid imaging in Alzheimer disease and mild cognitive impairment. A Phase 2 trial. Ann Neurol. 2010;68:319-29.

21. Hua X, Lee S, Yanovsky I, Leow AD, Chou YY, Ho AJ et al. Optimizing power to track brain degeneration in Alzheimer's disease and mild cognitive impairment with tensor-based morphometry: an ADNI study of 515 subjects. Neuroimage. 2009;48:668–81.

22. Hua X, Lee S, Hibar DP, Yanovsky I, Leow AD, Toga AW, et al. Mapping Alzheimer's disease progression in 1309 MRI scans: Power estimates for different inter-scan intervals. Neuroimage. 2010;51:63-75.

23. Leung KK, Clarkson MJ, Bartlett JW, Clegg S, Jack CR, Jr, Weiner MW, et al. Robust atrophy rate measurement in Alzheimer's disease using multi-site serial MRI: Tissue-specific intensity normalization and parameter selection. Neuroimage. 2010;50:516–23.

24. Morra JH, Tu Z, Apostolova LG, Green AE, Avedissian C, Madsen SK, et al. Automated 3D mapping of hippocampal atrophy and its clinical correlates in 400 subjects with Alzheimer's disease, mild cognitive impairment, and elderly controls. Hum Brain Mapp. 2009;30:2766-88.

25. Fjell AM, Walhovd KB, Fennema-Notestine C, McEvoy LK, Hagler DJ, Holland D, et al. Brain Atrophy in Healthy Aging Is Related to CSF Levels of A{beta}1-42. 2010. Cereb Cortex. 2010;20:2069-79.

26. Fjell AM, Walhovd KB, Fennema-Notestine C, McEvoy LK, Hagler DJ, Holland D, et al. CSF biomarkers in prediction of cerebral and clinical change in mild cognitive impairment and Alzheimer's disease. J Neurosci. 2010;30:2088–101.

27. Jack CR Jr, Wiste HJ, Vemuri P, Weigand SD, Senjem ML, Zeng G, et al. Brain beta-amyloid measures and magnetic resonance imaging atrophy both predict time-to-progression from mild cognitive impairment to Alzheimer's disease. Brain. 2010;133:3336-48.

28. DeSouza LC, Chupina M, Lamarie F, Jardela C, Leclercq D, Colliota O, et al. CSF tau markers are correlated with hippocampal volume in Alzheimer's disease. Neurobiol Aging. 2012;33:1253-7.

29. Blennow K, Zetterberg H, Rinne JO, Salloway S, Wei J, Black R, et al. Effect of immunotherapy with bapineuzumab on cerebrospinal fluid biomarker levels in patients with mild to moderate Alzheimer's disease. Arch Neurol. 2012;69:1002-010.

30. Folstein MF, Folstein SE, McHugh PR. "Mini-mental state." A practical method for grading the cognitive state of patients for the clinician. J Psychiatr Res. 1975;12:189-98.

31. Drozdick LW, Holdnack JA, Hilsabeck RC. Essentials of WMS-IV Assessment. John Wiley and Sons, 2011.

32. Berg L, Miller JP, Storandt M, Duchek J, Morris JC, Rubin EH, et al. Mild senile dementia of the Alzheimer type: 2. Longitudinal assessment. Ann Neurol. 1988;23:477-84.

33. Sheikh JI, Yesavage JA. Geriatric depression scale (GDS): Recent evidence and development of a shorter version. Clin Gerontol. 1986;23:477-84.

34. Rosen WG, Mohs RC, Davis KL A new rating scale for Alzheimer's disease. Am J Psychiatry. 1984;141:1356-64.

35. Pfeffer RI, Kurosaki TT, Harrah CH Jr, Chance JM, Filos S. Measurement of functional activities in older adults in the community. J Gerontol. 1982;37:323-9.

36. Guidance for Industry Suicidality: Prospective Assessment of Occurrence in Clinical Trials. Draft guidance, September 2010, US Department of Health and Human Services, Food and Drug Administration, Center for Drug Evaluation and Research.

11 PROCEDURES AND INSTRUCTIONS (ADMINISTRATIVE PROCEDURES)

11.1 Changes to the Protocol

Any change to the protocol requires a written protocol amendment or administrative change that must be approved by the sponsor before implementation. Amendments specifically affecting the safety of subjects, the scope of the investigation, or the scientific quality of the study require submission to health or regulatory authorities as well as additional approval by the applicable IRBs/IECs. These requirements should in no way prevent any immediate action from being taken by the investigator, or by the sponsor, in the interest of preserving the safety of all subjects included in the study. If the investigator determines that an immediate change to or deviation from the protocol is necessary for safety reasons to eliminate an immediate hazard to the subjects, the sponsor's medical monitor [and the IRB/IEC for the site must be notified immediately. The sponsor must notify the health or regulatory authority as required per local regulations.

Protocol amendments that affect only administrative aspects of the study may not require submission to health or regulatory authority or the IRB/IEC, but the health or regulatory authority and IRB/IEC (or if regionally required, the head of the medical institution) should be kept informed of such changes as required by local regulations. In these cases, the sponsor may be required to send a letter to the IRB/IEC and the Competent Authorities (or, if regionally required, the head of the medical institution) detailing such changes.

11.2 Adherence to the Protocol

The investigator will conduct the study in strict accordance with the protocol (refer to ICH E6, Section 4.5).

11.3 Monitoring Procedures

The sponsor's/CRO's CRA will maintain contact with the investigator and designated staff by telephone, letter, or email between study visits. Monitoring visits to each site will be conducted by the assigned CRA as described in the monitoring plan. The investigator (or if regionally required, the head of the medical institution) will allow the CRO to inspect the clinical, laboratory, and pharmacy facilities to assure compliance with GCP and local regulatory requirements. The eCRFs and subject's corresponding original medical records (source documents) are to be fully available for review by the sponsor's representatives at regular intervals. These reviews verify adherence to study protocol and data accuracy in accordance with local regulations. All records at the site are subject to inspection by the local auditing agency and IRB/IEC review.

In accordance with ICH E6, Section 1.52, source documents include, but are not limited to the following:

- Clinic, office, or hospital charts
- Copies or transcribed health care provider notes which have been certified for accuracy after production

- Recorded data from automated instruments such as IxRS, x-rays, and other imaging reports, (eg, sonograms, computed tomography scans, magnetic resonance images, radioactive images, ECGs, rhythm strips, electroencephalograms, polysomnographs, pulmonary function tests) regardless of how these images are stored, including microfiche and photographic negatives
- Pain, quality of life, or medical history questionnaires completed by subjects
- Records of telephone contacts
- Diaries or evaluation checklists
- Drug distribution and accountability logs maintained in pharmacies or by research personnel
- Laboratory results and other laboratory test outputs (eg, urine pregnancy test result documentation and urine dip-sticks)
- Correspondence regarding a study subject's treatment between physicians or memoranda sent to the IRBs/IECs
- eCRF components (eg, questionnaires) that are completed directly by subjects and serve as their own source

11.4 Recording of Data

An eCRF is required and must be completed for each subject by qualified and authorized personnel. All data on the eCRF must reflect the corresponding source document, except when a section of the eCRF itself is used as the source document. Any correction to entries made on the eCRF must be documented in a valid audit trail where the correction is dated, the individual making the correct is identified, the reason for the change is stated, and the original data are not obscured. Only data required by the protocol for the purposes of the study should be collected.

The investigator must sign each eCRF. The investigator will report the eCRFs to the sponsor and retain a copy of the eCRFs.

11.5 Identification of Source Data

All data to be recorded on the eCRFs must reflect the corresponding source documents.

11.6 Retention of Records

The circumstances of completion or termination of the study notwithstanding, the investigator (or if regionally required, the head of the medical institution or the designated representative) is responsible for retaining all study documents, including but not limited to the protocol, copies of eCRFs, the Investigator's Brochure, and regulatory agency registration documents (eg, Form FDA 1572, ICFs, and IRB/IEC correspondence). In addition, the sponsor will send a list of treatment codes by study subject to the investigator after the clinical database for this study has been locked. The site should plan to retain study documents, as directed by the sponsor, for at least 2 years after the last approval of a marketing application in an ICH region and until there

are no pending or contemplated marketing applications in an ICH region or at least 3 years have elapsed since the formal discontinuation of clinical development of the investigational product.

It is requested that at the completion of the required retention period, or should the investigator retire or relocate, the investigator contact the sponsor, allowing the sponsor the option of permanently retaining the study records.

11.7 Auditing Procedures and Inspection

In addition to the routine monitoring procedures, the sponsor’s Clinical Quality Assurance department conducts audits of clinical research activities in accordance with the sponsor’s SOPs to evaluate compliance with the principles of ICH GCP and all applicable local regulations. If a government regulatory authority requests an inspection during the study or after its completion, the investigator must inform the sponsor immediately.

11.8 Handling of Study Drug

All study drug will be supplied to the principal investigator (or a designated pharmacist) by the sponsor. Drug supplies must be kept in an appropriate secure area (eg, locked cabinet) and stored according to the conditions specified on the drug labels. The investigator (or a designated pharmacist) must maintain an accurate record of the shipment and dispensing of the study drug in a drug accountability ledger, a copy of which must be given to the sponsor at the end of the study. An accurate record of the date and amount of study drug administered to each subject must be available for inspection at any time. The CRA will visit the site and review these documents along with all other study conduct documents at appropriate intervals once study drug has been received by the site.

All drug supplies are to be used only for this study and not for any other purpose. The investigator (or site personnel) must not destroy any drug labels or any partly used or unused drug supply before approval to do so by the sponsor. At the conclusion of the study and as appropriate during the study, the investigator (or a designated pharmacist) will return all used and unused study drug, drug containers, drug labels, and a copy of the completed drug disposition form to the an Eisai designated facility.

11.9 Publication of Results

All manuscripts, abstracts, or other modes of presentation arising from the results of the study must be reviewed and approved in writing by the sponsor in advance of submission pursuant to the terms and conditions set forth in the executed Clinical Trial Agreement between the sponsor/CRO and the institution/investigator. The review is aimed at protecting the sponsor's proprietary information existing either at the date of the commencement of the study or generated during the study.

The detailed obligations regarding the publication of any data, material results, or other information, generated or created in relation to the study shall be set out in the agreement between each investigator and the sponsor or CRO, as appropriate.

11.10 Disclosure and Confidentiality

The contents of this protocol and any amendments and results obtained during the study should be kept confidential by the investigator, the investigator's staff, and the IRB/IEC and will not be disclosed in whole or in part to others, or used for any purpose other than reviewing or performing the study, without the written consent of the sponsor. No data collected as part of this study will be used in any written work, including publications, without the written consent of the sponsor. These obligations of confidentiality and non-use shall in no way diminish such obligations as set forth in either the Confidentiality Agreement or Clinical Trial Agreement executed between the sponsor/CRO and the institution/investigator.

All persons assisting in the performance of this study must be bound by the obligations of confidentiality and non-use set forth in either the Confidentiality Agreement or Clinical Trial Agreement executed between the institution/investigator and the sponsor/CRO.

11.11 Discontinuation of Study

The sponsor reserves the right to discontinue the study for medical reasons or any other reason at any time. If a study is prematurely terminated or suspended, the sponsor will promptly inform the investigators/institutions and regulatory authorities of the termination or suspension and the reason(s) for the termination or suspension. The IRB/IEC will also be informed promptly and provided the reason(s) for the termination or suspension by the sponsor or by the investigator/institution, as specified by the applicable regulatory requirement(s).

The investigator reserves the right to discontinue the study should his/her judgment so dictate. If the investigator terminates or suspends a study without prior agreement of the sponsor, the investigator should inform the institution where applicable, and the investigator/institution should promptly inform the sponsor and the IRB/IEC and provide the sponsor and the IRB/IEC with a detailed written explanation of the termination or suspension. Study records must be retained as noted above.

11.12 Subject Insurance and Indemnity

The sponsor will provide insurance for any subjects participating in the study in accordance with all applicable laws and regulations.

12 APPENDICES

Appendix 1 Sponsor's Grading for Laboratory Values

Sponsor's Grading for Laboratory Values				
	**Grade 1**	**Grade 2**	**Grade 3**	**Grade 4**
**BLOOD/BONE MARROW**				
Hemoglobin	< LLN – 10.0 g/dL<br>< LLN – 100 g/L<br>< LLN – 6.2 mmol/L	< 10.0 – 8.0 g/dL<br>< 100 – 80 g/L<br>< 6.2 – 4.9 mmol/L	< 8.0 g/dL<br>< 80 g/L<br>< 4.9 mmol/L;<br>transfusion indicated	life-threatening consequences; urgent intervention indicated
Leukocytes (total WBC)	< LLN – 3.0 x 10^9/L<br>< LLN – 3000/mm^3	< 3.0 – 2.0 x 10^9/L<br>< 3000 – 2000/mm^3	< 2.0 – 1.0 x 10^9/L<br>< 2000 – 1000/mm^3	< 1.0 x 10^9/L<br>< 1000/mm^3
Lymphocytes	< LLN – 800/mm^3<br>< LLN – 0.8 x 10^9/L	< 800 – 500/mm^3<br>< 0.8 – 0.5 x 10^9/L	< 500 – 200/mm^3<br>< 0.5 – 0.2 x 10^9/L	< 200/mm^3<br>< 0.2 x 10^9/L
Neutrophils	< LLN – 1.5 x 10^9/L<br>< LLN – 1500/mm^3	< 1.5 – 1.0 x 10^9/L<br>< 1500 – 1000/mm^3	< 1.0 – 0.5 x 10^9/L<br>< 1000 – 500/mm^3	< 0.5 x 10^9/L<br>< 500/mm^3
Platelets	< LLN – 75.0 x 10^9/L<br>< LLN – 75,000/mm^3	< 75.0 – 50.0 x 10^9/L<br>< 75,000 – 50,000/mm^3	< 50.0 – 25.0 x 10^9/L<br>< 50,000 – 25,000/mm^3	< 25.0 x 10^9/L<br>< 25,000/mm^3
**METABOLIC/LABORATORY**				
Albumin, serum- low (hypoalbuminemia)	< LLN – 3 g/dL<br>< LLN – 30 g/L	< 3 – 2 g/dL<br>< 30 – 20 g/L	< 2 g/dL<br>< 20 g/L	life-threatening consequences; urgent intervention indicated
Alkaline phosphatase	> ULN – 3.0 x ULN	> 3.0 – 5.0 x ULN	> 5.0 – 20.0 x ULN	> 20.0 x ULN
ALT	> ULN – 3.0 x ULN	> 3.0 – 5.0 x ULN	> 5.0 – 20.0 x ULN	> 20.0 x ULN
AST	> ULN – 3.0 x ULN	> 3.0 – 5.0 x ULN	> 5.0 – 20.0 x ULN	> 20.0 x ULN
Bicarbonate, serum-low	< LLN – 16 mmol/L	< 16 – 11 mmol/L	<11 – 8 mmol/L	< 8 mmol/L
Bilirubin (hyperbilirubinemia)	> ULN – 1.5 x ULN	> 1.5 – 3.0 x ULN	> 3.0 – 10.0 x ULN	> 10.0 x ULN
Calcium, serum-low (hypocalcemia)	< LLN – 8.0 mg/dL<br>< LLN – 2.0 mmol/L	< 8.0 – 7.0 mg/dL<br>< 2.0 – 1.75 mmol/L	< 7.0 – 6.0 mg/dL<br>< 1.75 – 1.5 mmol/L	< 6.0 mg/dL<br>< 1.5 mmol/L
Calcium, serum-high (hypercalcemia)	> ULN – 11.5 mg/dL<br>> ULN – 2.9 mmol/L	> 11.5 – 12.5 mg/dL<br>> 2.9 – 3.1 mmol/L	> 12.5 – 13.5 mg/dL<br>> 3.1 – 3.4 mmol/L	> 13.5 mg/dL<br>> 3.4 mmol/L
Cholesterol, serum-high (hypercholesterolemia)	> ULN – 300 mg/dL<br>> ULN – 7.75 mmol/L	> 300 – 400 mg/dL<br>> 7.75 – 10.34 mmol/L	> 400 – 500 mg/dL<br>> 10.34 – 12.92 mmol/L	> 500 mg/dL<br>> 12.92 mmol/L
Creatinine	> ULN – 1.5 x ULN	> 1.5 – 3.0 x ULN	> 3.0 – 6.0 x ULN	> 6.0 x ULN
GGT (γ-Glutamyl transpeptidase)	> ULN – 3.0 x ULN	> 3.0 – 5.0 x ULN	> 5.0 – 20.0 x ULN	> 20.0 x ULN
Glucose, serum-high (hyperglycemia)	Fasting glucose value:<br>> ULN – 160 mg/dL<br>> ULN – 8.9 mmol/L	Fasting glucose value:<br>> 160 – 250 mg/dL<br>> 8.9 – 13.9 mmol/L	> 250 – 500 mg/dL;<br>> 13.9 – 27.8 mmol/L;<br>hospitalization indicated	> 500 mg/dL;<br>> 27.8 mmol/L;<br>life-threatening consequences
Glucose, serum-low (hypoglycemia)	< LLN – 55 mg/dL<br>< LLN – 3.0 mmol/L	< 55 – 40 mg/dL<br>< 3.0 – 2.2 mmol/L	< 40 – 30 mg/dL<br>< 2.2 – 1.7 mmol/L	< 30 mg/dL<br>< 1.7 mmol/L<br>life-threatening consequences;<br>seizures
Phosphate, serum-low (hypophosphatemia)	< LLN – 2.5 mg/dL<br>< LLN – 0.8 mmol/L	< 2.5 – 2.0 mg/dL<br>< 0.8 – 0.6 mmol/L	< 2.0 – 1.0 mg/dL<br>< 0.6 – 0.3 mmol/L	< 1.0 mg/dL<br>< 0.3 mmol/L<br>life-threatening consequences
Potassium, serum-high (hyperkalemia)	> ULN – 5.5 mmol/L	> 5.5 – 6.0 mmol/L	> 6.0 – 7.0 mmol/L<br>hospitalization indicated	> 7.0 mmol/L<br>life-threatening consequences
Potassium, serum-low (hypokalemia)	< LLN – 3.0 mmol/L	< LLN – 3.0 mmol/L;<br>symptomatic;<br>intervention indicated	< 3.0 – 2.5 mmol/L<br>hospitalization indicated	< 2.5 mmol/L<br>life-threatening consequences

Sponsor's Grading for Laboratory Values

	Grade 1	Grade 2	Grade 3	Grade 4
Sodium, serum-high (hypernatremia)	> ULN – 150 mmol/L	> 150 – 155 mmol/L	> 155 – 160 mmol/L hospitalization indicated	> 160 mmol/L life-threatening consequences
Sodium, serum-low (hyponatremia)	< LLN – 130 mmol/L	N/A	< 130 – 120 mmol/L	< 120 mmol/L life-threatening consequences
Triglyceride, serum-high (hypertriglyceridemia)	150 – 300 mg/dL<br>1.71 – 3.42 mmol/L	> 300 – 500 mg/dL<br>> 3.42 – 5.7 mmol/L	> 500 – 1000 mg/dL<br>>5.7 – 11.4 mmol/L	> 1000 mg/dL<br>> 11.4 mmol/L<br>life-threatening consequences
Uric acid, serum-high (hyperuricemia)	> ULN – 10 mg/dL<br>≤ 0.59 mmol/L without physiologic consequences	N/A	> ULN – 10 mg/dL<br>≤ 0.59 mmol/L with physiologic consequences	> 10 mg/dL<br>> 0.59 mmol/L<br>life-threatening consequences

ALT = alanine aminotransferase (serum glutamic pyruvic transaminase), AST = aspartate aminotransferase (serum glutamic oxaloacetic transaminase), GGT = γ-glutamyl transpeptidase, N/A = not applicable, LLN = lower limit of normal, ULN = upper limit of normal, WBC = white blood cell.
Based on Common Terminology Criteria for Adverse events (CTCAE) Version 4.0. Published: May 28, 2009 (v4.03: June 14, 2010).

Appendix 2 List of Permitted and Prohibited Prior/Concomitant Medications

Restricted medications are listed in Listing #1. Medications that are permitted with restrictions are listed in Listings #2, #3, and #4. (revised per Amendment 12)

These listings of restricted and permitted medications may not be all-inclusive listings. Please contact the medical monitor(s) if the investigators have any questions or concerns regarding any medication not listed that your subjects may be taking before enrollment or start during the course of the BAN2401-A001-201 study. (revised per Amendment 12)

Antibiotics, analgesics and anti-inflammatory drugs intended for short courses of treatment are permitted and do not need to follow the restrictions of Listing #4 for a stable dose of 4 weeks prior to Baseline. If these medications may affect cognitive function but the medications are clinically indicated, then cognitive testing should not take place until at least 72 hours after the last dose of the course of treatment.

Postrandomization, subjects who require anticoagulant treatment as prophylaxis for ≤4 weeks need not be withdrawn if during this period study drug treatment is suspended, after discussion with sponsor medical monitor. See Listing 1 below for a list of restricted medications. (revised per Amendments 01, 06, and 12)

Listing 1 Restricted Medications Within 4 Weeks Prior to Extension Baseline (Extension Screening Visit)Until Follow-up Visit

Generic Name	Trade Name
**Anticoagulants (See Appendix 4 for Anticoagulant use during Extension Phase)**	
Warfarin	Coumadin®, Jantoven
Dabigatran	Pradaxa
Enoxaparin	Lovenox
Dalteparin	Fragmin
Anisindione	Miradon
Tinzaparin	Innohep
Andeparin sodium	Normiflo
Heparin Sodium	Hemochron
Bivalirudin	Angiomax
Argatroban	
Rivaroxaban	Xarelto
Apixaban	Eliquis
Endoxaban	Savaysa
Lepirudin	Refludan
Fondaparinux	Arixtra®
Antithrombin III	ATryn, Thrombate III
Thrombolytics are permitted; however, treatment with study drug should be temporarily suspended during thrombolytic therapy until stabilization or resoluation of the medical condition that required thrombolytic drug treatment (thrombolytic examples below).	
Streptokinase	Streptase
Reteplase	Retavase, Retevase
Tenecteplase	TNKase
Urokinase	Abbokinase, Kinlytic
Alteplase, tPA	Activase, Cathflo Activase
**Antiplatelet drugs are permitted but the subject should be on a stable dose for 4 weeks before Extension Baseline (Extension Screening Visit).**	
Aspirin or clopidogrel is permitted in all subjects. Aspirin and clopidogrel in combinatioin is permitted in subjects who are not to undergo lumbar puncture.	Aspirin and Plavix<br>(only in subjects undertaking LP in biomarker subgroup)

Listing 1 (cont'd)
Restricted Medications Within 6 Months Prior to Baseline (Core Baseline or Extension Baseline (Extension Screening Visit) Until Follow-up Visit

Immunosuppressants & Immunoglobulin therapy	
Ranibizumab	Lucentis
Bevacizumab	Avastin
Infliximab	Remicade
Etanercept	Enbrel
Adalimumab	Humira
Omalizumab	Xolair
Efalizumab	Raptiva
Immune Globulin IV, IVIG, IGIV	Carimune, Flebogamma, Gamimune N, Gammagard, Gamunex, Gammar-P IV, Iveegam, Octagam, Panglobulin, Vigam, Polygam S/D, Privigen, Sandoglobulin, Venoglobulin-S
Other monoclonal antibodies not listed here	

Listing 2 Medications Which Need To Be on a Stable Dose for 12 Weeks Prior to Baseline (Core Baseline or Extension Baseline [Extension Screening Visit]) and Remain on the Same Stable Dose from Baseline to Termination Visit

Cognitive Enhancers	
Donepezil	Aricept
Rivastigmine	Exelon
Galantamine	Reminyl
Memantine	Namenda
Donepezil and Memantine combination (revised per Amendment 06)	Donamem (revised per Amendment 06)
Any other drug approved in the subject's country for treatment of cognitive impairment	

Listing 3 Permitted Medications Used on PRN Basis Which Are Not To Be Used Within 72 Hours of Cognitive Testing

Examples are shown below; this list is not exhaustive.

Generic Name	**Trade Name**
**Opiates**	
Morphine	Astramorph PF, Avinza, Depo Dur, Duramorph, Infumorph, Kadian, MS Contin, MSIR, Oramorph SR, RMS, Roxanol
**Opiate Agonists**	
Meperidine	Demerol, Meperitab
Fentanyl	Actiq, Duragesic, Fentora, Onsolis, Sublimaze
Codeine	Codeine
Oxycodone	Dazidox, Endocodone, Eth-OxyDose, OxyContin, OxyFast, OxyIR, Percolone, Roxicodone, Roxicodone Intensol
Other opioids or opioid agonists which are used on PRN basis	
**Benzodiazepines & sedatives**	
Estazolam	ProSom
Alprazolam	Alprazolam Intensol, Niravam, Xanax
Triazolam	Halcion
Midazolam	Versed
Lorazepam	Ativan
Flunitrazepam	Rohypnol
Temazepam	Restoril
Clonazepam	Ceberclon, Klonopin
Diazepam	Diastat, Dizac, Valium
Chlordiazepoxide	H-Tran, Librium
Flurazepam	Dalmane
Zopiclone	(various)
Zolpidem	Ambien; others
Ramelteon	Rozerem
Amitriptyline	(various)
Dothiepin	(various)
Risperidone	Risperdal
Olanzapine	Zyprexa
Promethazine	Phenergan; others
Other benzodiazepines, tricyclic antidepressants, anti-psychotics and other drugs which are used on PRN basis as sedatives	

If the following permitted medications are used on a PRN basis, see restrictions in Listing 3. If they are used chronically, they are required to be on a stable dose for at least 4 weeks prior to baseline (Core Baseline or Extension Baseline [Extension Screening Visit]). Changes in dose or initiation of new concomitant medications for chronic use (not PRN use) is permitted during the study but if these medications may affect cognitive function, no cognitive testing should be conducted until the dose has been stable for 4 weeks. Examples are given below:

Listing 4 Permitted Medications

Generic Name	Trade Name
**Antidepressants**	
Bupropion	Aplenzin, Budeprion, Buproban, Zyban, Wellbutrin
Nefazodone	Serzone
Rasagiline	Azilect
Selegiline	EMSAM
Isocarboxazid	Marplan
Phenelzine	Nardil
Tranylcypromine	Parnate
Citalopram	Celexa
Fluvoxamine	Luvox
Escitalopram	Lexapro
Paroxetine	Paxil, Pexeva
Fluoxetine	Prozac, Sarafem, Selfemra
Sertraline	Sertaraline, Zoloft
Duloxetine	Cymbalta
Venlafaxine Hydrochloride	Effexor
Desvenlafaxine	Pristiq
Amitriptyline Hydrochloride	Elavil
Clomipramine Hydrochloride	Anafranil
Desipramine Hydrochloride	Norpramin
Doxepin Hydrochloride	Prudoxin, Silenor, Sinequan, Zonalon
Imipramine Hydrochloride	Tofranil
Nortriptyline Hydrochloride	Aventyl, Pamelor
Trimipramine Maleate	Surmontil
Protriptyline Hydrochloride	Vivactil
Amoxapine	Amoxapine
Trazodone Hydrochloride	Desyrel, Oleptro
Maprotiline Hydrochloride	Ludiomil
Mirtazapine	Remeron Soltab
**Mood Stabilizers**	
Fluoxetine; Olanzapine	Symbyax
Carbamazepine	Carbatrol, Epitol, Equetro, Tegretol, Tegretol XR
Lamotrigine	Lamictal, Lamictal CD, Lamictal ODT, Lamictal XR
Valproic Acid	Depacon, Depakene, Depakote, Depakote ER, Divalproex Sodium, Stavzor, Valproate

Listing 4 Permitted Medications

Generic Name	Trade Name
**Antipsychotics**	
Acetophenazine	Tindal
Aripiprazole	Abilify
Asenapine	Saphris
Olanzapine	Zyprexa
Chlorpromazine	Thorazine
Haloperidol	Haloperidol, Haldol
Clozapine	Clozaril, Fazaclo
Fluphenazine	Prolixin
L-Methylfolate	Deplin, L-Methylfolate Calcium
Loxapine	Loxitane
Mesoridazine	Serentil
Perphenazine	Trilafon
Quetiapine	Seroquel
Risperidone	Risperdal
Thioridazine	Mellaril
Thiothixene	Navane
Trifluoperazine	Stelazine
**Benzodiazepines**	
Estazolam	ProSom
Alprazolam	Alprazolam Intensol, Niravam, Xanax
Triazolam	Halcion
Midazolam	Versed
Lorazepam	Ativan
Flunitrazepam	Rohypnol
Temazepam	Restoril
Clonazepam	Ceberclon, Klonopin
Diazepam	Diastat, Dizac, Valium
Chlordiazepoxide	H-Tran, Librium
Flurazepam	Dalmane
Zopiclone	
Zopidem	

Appendix 3 Pharmacogenomic and Biomarker Bioanalysis

Subjects enrolled in this clinical study will have blood and CSF (if consented) samples collected for Pharmacogenomic and biomarker analysis. The aim of this research is to identify factors that may influence subject's exposure to the study drug, as well as genetic factors that may have an effect on clinical response or potential AEs related to study treatment and to explore the role of genetic variability in response. Blood samples may be analyzed to determine subject's genotypes or sequence for a number of genes or non-coding regulatory regions. The research may include the investigation of polymorphisms in genes that are likely to influence the study drug pharmacokinetics or therapeutic response. Similarly, biomarker discovery and validation may be performed along with samples from AD subjects, to identify blood and genetic biomarkers which may be useful to predict subject PK/PD response to BAN2401.

Collection of the samples for Pharmacogenomic and biomarker analysis will be bound by the sample principles and processes outlined in the main study protocol.

Sample collection and handling
The pharmacogenomic and biomarker samples will be collected according to the study flow chart. If for operational or medical reasons the blood samples for Pharmacogenomic and biomarker analysis cannot be obtained at the pre-specified visit, the sample can be taken at any study center visit at the discretion of the investigator and site staff.

Security of the samples, use of the samples, retention of the samples
Sample processing, including DNA extraction and genotyping, sequencing or other analysis will be performed by a laboratory under the direction of the Sponsor. Processing, analysis and storage will be performed at a secure laboratory facility to protect the validity of the data and maintain subject privacy.

Pharmacogenomic and biomarker samples will only be used for the purposes described in this protocol by the Sponsor. Laboratories contracted to perform the analysis on behalf of the Sponsor will not retain rights to the samples beyond those necessary to perform the specified analysis, and will not transfer or sell those samples. The Sponsor will not sell the samples to a third party.

Pharmacogenomic and biomarker samples may be stored for up to 15 years after the completion of the study (defined as submission of the CSR to appropriate regulatory agencies). (revised per Amendment 01) At the end of the storage period, samples will be destroyed. Samples may be stored for longer if a Health Authority (or medicinal product approval agency) has active questions about the study. In this special circumstance, the samples will be stored until the questions have been adequately addressed.

It is possible that future research and technological advances may identify genomic variants of interest, or allow alternative types of genomic analysis not foreseen at this time. Because it is not possible to prospectively define every avenue of future testing, samples collected will be single or double coded (according to the ICH15 guidelines) in order to maintain subject privacy.

Right to withdraw

If during the time the Pharmacogenomic and biomarker samples are stored a participant would like to withdraw their consent for participation in this research, Eisai will destroy the samples, if they can still be identified (not anonymized). Once samples have been anonymized, it will not be possible to identify which samples have come from a particular individual. Therefore, it will not be possible to destroy subject samples after anonymization. Information from any assays that have already been completed at the time of withdrawal of consent will continue to be used as necessary to protect the integrity of the research project.

Subject privacy and return of data

No subject identifying information (eg initials, date of birth, government identifying number) will be associated with the samples. Samples that are processed for analysis (DNA/RNA extracted) may be double coded. Double coding involves removing the initial code and replacing with another code such that the subject can be re-identified by use of 2 code keys. The code keys are usually held by different parties. The key linking the sample ID to the subject number will be maintained separately from the sample. At this point, the samples will be double coded (the first code being the subject number) as long as the initial tube does not carry any personal identifiers or the random code assigned by the central laboratory or biorepository. Laboratory personnel performing genetic analysis will not have access to the "key". Laboratory personnel performing the analysis will not have access to the "key". Clinical data collected as part of the clinical trial will be cleaned of subject identifying information, and linked by use of the sample ID "key".

The Sponsor will take steps to insure that data are protected accordingly and confidentiality is maintained as far as possible. Data from subjects enrolled in this study may be analyzed worldwide, regardless of location of collection.

The Sponsor, its representatives and agents, may share anonymized data with persons and organizations involved in the conduct or oversight of this research. These include:

- Clinical research organizations retained by the Sponsor
- Independent ethics committees or institutional review boards that have responsibility for this research study
- National regulatory authorities or equivalent government agencies

At the end of the analysis, results may be presented in a final report which can include part or all of the anonymized data, in listing or summary format. Other publication (eg, in peer reviewed scientific journals) or public presentation of the study results will only include summaries of the population in the study, and no identified individual results will be disclosed.

All subjects must provide blood for *APOE4* for genotyping. Individual data will not be returned to subjects unless specifically required by law. (revised per Amendment 01)

In countries where local law requires the return of PG or biomarker data to subjects, no exploratory PG or biomarker analysis will be conducted on biosamples from subjects in that

jurisdiction. Additionally, no additional biosamples will be taken for exploratory PG or biomarker analysis from subjects in that jurisdiction. (revised per Amendment 01)

Appendix 4 EXTENSION PHASE (revised per Amendment 11)

PRIMARY OBJECTIVE

To evaluate the long-term safety and tolerability of BAN2401 in subjects with EAD

Secondary Objective

To assess if the treatment benefit in brain amyloid levels (as measured by amyloid PET) at the end of the Core Study will be maintained over time in the Extension Phase in subjects with EAD

EXPLORATORY OBJECTIVES

1. To evaluate the clinical effects of BAN2401 in subjects with EAD on the ADCOMS,CDR-SB, ADAS-Cog, and MMSE over time
2. To assess time to disease progression as evaluated CDR global score during the Extension Phase (revised per Amendment 12)
3. To explore the long-term effects of BAN2401 in subjects with EAD on total hippocampal volume and other biomarkers (eg, plasma biomarkers) at 12, and approximately 24 months in the Extension Phase (Visit 70 [Extension Week 53], Visit 95 [Extension Week 103])
4. To characterize population PK of BAN2401 in subjects enrolled in the Extension Phase of the study

ELIGIBILITY CRITERIA (revised per Amendment 12)

Inclusion:

1. Subjects who have completed Visit 42 (Week 79) of the Core Study or who discontinued study drug during the Core Study due to any of the following reasons:
    a. ARIA-E
    b. amyloid related imaging abnormality hemorrhage (ARIA-H) (superficial siderosis, macrohemorrhage, or symptomatic microhemorrhage)
    c. Prohibited or restricted medications that were prohibited during Core Study conduct but are no longer prohibited in the Extension Phase
    d. Subjects who were APOE4 positive and receiving treatment with BAN2401 10 mg/kg biweekly
    e. Any reason for discontinuation not related to prohibited medications, including any AE that was considered not related to study drug, and that was not severe or life-threatening
2. Must continue to have an identified caregiver or informant who is willing and able to provide follow-up information on the subject throughout the course of the Extension Phase
3. Provide written informed consent. If a subject lacks capacity to consent in the investigator's opinion, the subject's assent should be obtained, if required in accordance with local laws, regulations and customs, plus the written informed consent of a legal representative should be obtained (capacity to consent and definition of legal

representative should be determined in accordance with applicable local laws and regulations).

4. Must be able to physically attend clinic visits and be willing and able to comply with all aspects of the protocol

Exclusion:

1. Subjects who discontinued from the study drug or from the the Core Study for reasons other than the following:
    a. ARIA-E
    b. ARIA-H (superficial siderosis, macrohemorrhage, or symptomatic microhemorrhage)
    c. Prohibited or restricted medications that were prohibited during Core Study conduct but are no longer prohibited in the Extension Phase
    d. Subjects who were APOE4 positive and receiving treatment with BAN2401 10 mg/kg biweekly
    e. AE that was considered not related to study drug, and that was not severe or life-threatening
2. Females who are breastfeeding or pregnant at Extension Phase Baseline (Extension Screening Visit, as documented by a positive β-hCG assay). A separate baseline assessment is required if a negative screening pregnancy test was obtained more than 72 hours before the first dose of study drug.
3. Females of childbearing potential who:
    - Had unprotected sexual intercourse within 30 days before Extension Phase Baseline and who do not agree to use a highly effective method of contraception (eg, total abstinence, an intrauterine device, a double-barrier method [such as condom plus diaphragm with spermicide], a contraceptive implant, an oral contraceptive, or have a vasectomized partner with confirmed azoospermia) throughout the entire study period and for 28 days after study drug discontinuation.
    - Are currently abstinent, and do not agree to use a double-barrier method (as described above) or refrain from becoming sexually active during the study period or for 28 days after study drug discontinuation.
    - Are who are using hormonal contraceptives but are not on a stable dose of the same hormonal contraceptive product for at least 4 weeks before dosing and who do not agree to use the same contraceptive during the study and for 28 days after study drug discontinuation.

    (NOTE: All females will be considered to be of childbearing potential unless they are postmenopausal [amenorrheic for at least 12 consecutive months, in the appropriate age group, and without other known or suspected cause] or have been sterilized surgically [ie, bilateral tubal ligation, total hysterectomy, or bilateral oophorectomy, all with surgery at least 1 month before dosing]).
4. Subjects who develop the following conditions from the time of completion of the Core Study to the start of the Extension Phase:

a. Contraindications to MRI scanning, including cardiac pacemaker/defibrillator, ferromagnetic metal implants (eg, in skull and cardiac devices other than those approved as safe for use in MRI scanners)
b. Other significant pathological findings on brain MRI at Extension Phase Baseline (Extension Screening Visit), including but not limited to: any macro-hemorrhage (greater than 10 mm at greatest diameter), which is currently symptomatic or worsened since the Core Study; any area of superficial siderosis, which is currently symptomatic or worsened since the Core Study; evidence of vasogenic edema, which is severe or symptomatic; aneurysms, vascular malformations, infective lesions, evidence of multiple lacunar infarcts or stroke involving a major vascular territory, severe small vessel, or white matter disease or space occupying lesions or brain tumors (however lesions diagnosed as meningiomas or arachnoid cysts and <1cm at their greatest diameter need not be exclusionary)
c. Any immunological disease which is not adequately controlled, or which requires treatment with biologic drugs during the study
d. Bleeding disorder that is not under adequate control (including a platelet count <50,000 or INR >1.5)
e. Receiving treatment with anticoagulant therapy but anticoagulation control is not optimized and is stable for at least 4 weeks before Extension Phase Baseline (Extension Screening Visit)
f. Any other clinically significant abnormalities in physical examination, vital signs, laboratory tests or ECG at Extension Phase Baseline (Extension Screening Visit) which in the opinion of the PI, require further investigation or treatment or which may interfere with study procedures or safety
g. Malignant neoplasms within 3 years of Extension Phase Baseline (Extension Screening Visit) (except for basal or squamous cell carcinoma in situ of the skin, or localized prostate cancer in male subjects). Subjects who had malignant neoplasms but who have had at least 3 years of documented uninterrupted remission before Extension Screening Visit need not be excluded
h. Known or suspected history of drug or alcohol abuse or dependence within 2 years before Extension Phase Baseline (Extension Screening Visit) or a positive urine drug test at Extension Screening Visit. Subjects who test positive for benzodiazepines or opioids in urine drug testing need not be excluded if in the clinical opinion of the investigator, this is due to the subject taking prior/concomitant medications containing benzodiazepines or opioids for a medical condition and not due to drug abuse
i. Any other medical conditions (eg, cardiac, respiratory, gastrointestinal, renal disease) which are not stably controlled, or which in the opinion of the investigator(s) could affect the subject's safety or interfere with the study assessments
j. Subjects who are taking prohibited or restricted medications
k. Participation in a clinical study involving any therapeutic monoclonal antibody, protein derived from a monoclonal antibody, immunoglobulin therapy, or vaccine during the time between the Core Study and Extension Phase Baseline (Extension Screening Visit)

l. Participation in a clinical study involving any new chemical entities for AD during the time between the Core Study and Extension Phase Baseline (Extension Screening Visit)
m. Participation in any other investigational medication or device study during the time between the Core Study and Extension Phase Baseline (Extension Screening Visit)
n. Planned surgery which requires general, spinal or epidural anesthesia that would take place during the study. Planned surgery which requires only local anesthesia and which can be undertaken as day case without inpatient stay postoperatively need not result in exclusion if in the opinion of the principal investigator this operation does not interfere with study procedures and subject safety

5. Severe visual or hearing impairment that would prevent the subject from performing psychometric tests accurately

STUDY DESIGN AND PLAN

The Extension Phase will be initiated following Core Study to allow subjects to receive open-label BAN2401 10 mg/kg biweekly for up to 24 months (2 years) until the drug is commercially available in the country where the subject resides, or until the benefit-to-risk ratio from treatment with BAN2401 is no longer considered favorable, whichever comes first. All subjects will receive BAN2401 10 mg/kg biweekly dose during the Extension Phase, including subjects who are confirmed APOE4 positive (hetero- or homozygous). Any subject who completed Visit 42 (Week 79) of the Core Study will have the option to participate in the Extension Phase. Subjects who previously completed the Core Study (through the Follow-Up Visit; Visit 43) at any time before implementation of the Extension Phase, and/or fulfill the Extension Phase inclusion and exclusion criteria, will also be eligible to participate in the Extension Phase. Subjects who discontinued the Core Study will be eligible to participate in the Extension Phase, provided they meet the inclusion and exclusion criteria for the Extension Phase. (revised per Amendment 12)

All subjects who underwent amyloid PET for inclusion in the Core Study should receive a baseline amyloid PET scan before dosing in the Extension Phase. The baseline amyloid PET scan must be conducted with the same imaging tracer that was used for inclusion at the baseline visit for the Core Study. In addition, qualified subjects located in the US and Japan will have the option to participate in the longitudinal PET substudy. Florbetapir will be used in the US and flutemetamol will be used in Japan in this imaging substudy, and only subjects who consent to the longitudinal assessments per the Extension Phase Schedule of Procedures/Assessments (Table 12) will be allowed to participate in this substudy. (revised per Amendment 12)

The Follow-up Visit will take place 3 months after the last dose of study drug. Subjects may discontinue from study drug for any reason. Subjects who discontinue the study or study drug must comply with the Early Termination Visit (within 7 days after the last dose of study drug) and the Follow-Up Visit (3 months after the last dose of study drug).

The study will end when the last visit for the last subject has completed the Extension Phase.

EXTENSION PHASE ASSESSMENTS

All subjects will provide informed consent and subjects will be assessed for eligibility for the Extension Phase. Current medical history will be assessed at the Extension Phase Baseline visit (Extension Screening Visit) to ensure safety in continued study participation. A complete physical examination will be performed at Extension Phase baseline (Extension Screening Visit). Safety assessments will be performed and all AEs and SAEs recorded. Vital signs will be assessed when study drug is administered both at predose and after infusion. During Visits 44, 45, 46, and 47, vital signs should be obtained at least 2 hours after infusion.

Hematology, blood chemistry assessments, and urinalysis will be performed at Extension Baseline (Extension Screening Visit), at Extension Phase Weeks 3, 7, 13, 19, 27 (Visits 48, 50, 57, 70, 83 and 96), and every 6 months thereafter. Safety and vMRIs will be performed at Extension Baseline (Extension Screening Visit), at Extension Phase Weeks 9, 13, 27, and every 6 months thereafter.

Clinical assessments will be administered at baseline (Extension Screening Visit) and every 6 months in the morning (whenever possible) in the following order: MMSE, CDR, and the ADAS-Cog.

Florbetapir will be used in the US and flutemetamol will be used in Japan for longitudinal amyloid PET analysis in qualified subjects who participate in the longitudinal imaging substudy (US and Japan only). (revised per Amendment 12) At the Extension Screening Visit, subjects who consented to the longitudinal imaging substudy will be stratified into 2 cohorts based on their treatment allocation during the Core Study. Cohort 1 will have amyloid PET assessments performed at baseline (Extension Screening Visit), Visit 50 (Extension Week 13), Extension Phase Visit 70 [Extension Week 53], and Visit 96 [Extension Week 105]; Cohort 2 will have amyloid PET assessments performed at baseline (Extension Screening Visit), Visit 57 (Extension Week 27), at Extension Phase Visit 70 [Extension Week 53], and Visit 96 [Extension Week 105]. In Japan, those who consented to the longitudinal imaging substudy will only undergo amyloid PET at Extension Phase Visit 70 [Extension Week 53], and Visit 96 [Extension Week 105]. (revised per Amendment 12)

Blood for serum PK and anti-BAN2401 for PK and anti-BAN2401 antibodies will be collected at Extension Phase Week 1 (Visit 44), Extension Weeks 3, 9, 13, 27, 39, 53, 79, 103 (last dosing visit), 105, and 117. These samples should not be taken from the same arm as that in which the study drug is infused, and subjects must stay in clinic for at least 2 hours following infusion up through Visit 50. After this visit, if no untoward effects of infusion are noted, or if infusion reactions can be prevented with prophylaxis as per Section 9.5.1.5, subjects may be discharged from clinic 30 minutes after the end of infusion if judged medically stable by the investigator.

All AEs observed during the study will be reported on the eCRF. All AEs, regardless of relationship to study drug or procedure, should be collected beginning from the time the subject signs the study ICF through the last visit (Visit 97, as shown in **Table 12**). The following adverse events will require the collection of information sufficient to provide a detailed description of the event, treatment, and outcome to the Medical Monitor for the study: ARIA-E, ARIA-H, infusion reaction, skin rash considered to be related to study drug, other

hypersensitivity reactions, and a "yes" answer to Type 4 or 5 suicidal ideation, or a "yes" response to any suicidal behavior on the C-SSRS. It is the responsibility of the investigator to review the results of the C-SSRS and determine if any result constitutes an AE. These AEs do not need to be reported as SAEs unless they meet the criteria for Reporting of Serious Adverse Events (Section 9.5.4.1).

During the Extension Phase, subjects who develop asymptomatic ARIA-H, will continue on the study uninterrupted per the Schedule of Assessments and do not require additional MRI follow-up outside the regularly scheduled assessments. Subjects who develop multiple (>10) asymptomatic cerebral microhemorrhages, superficial siderosis, or a single macrohemorrhage (greater than 10 mm at greatest diameter), will continue on the study uninterrupted per the Schedule of Assessments, and will undertake an unscheduled safety visit (with MRI) at approximately 30 days after the MRI features (asymptomatic ARIA-H) were first identified. Thereafter, all subjects who develop these events will have further safety visits (with MRI) at approximately every 30 days (which may be an unscheduled or a scheduled visit) until the asymptomatic ARIA-H has stabilized radiographicially. Subjects who develop symptomatic ARIA-H (including symptomatic microhemmorhages, symptomatic superficial siderosis, symptomatic macrohemorrhage) on MRI will have study drug administration temporarily stopped. All subjects who develop these events will undertake an unscheduled safety visit (with MRI) at approximately 30 days after the MRI features (ARIA-H) were first identified. Thereafter they will have further safety visits (with MRI) at approximately every 30 days (which may be unscheduled or scheduled visit) until the ARIA-H has stabilized and is no longer symptomatic. During treatment interruption, time will elapse on the Schedule of Assessments when the scheduled visit would otherwise occur. They may then resume treatment for the study duration and study assessments remaining on the Schedule of Assessments. Resumption of treatment following symptomatic ARIA-H can only occur twice, after which the subject must be discontinued from the study. (revised per Amendment 12)

Subjects who develop asymptomatic, radiographically mild or moderate Amyloid Related Imaging Abnormality–Edema/Effusion (ARIA-E) on MRI will continue on the study uninterrupted per the Schedule of Assessments. They will undertake an unscheduled safety visit (with MRI) at approximately 30 days and another safety visit (with MRI) at approximately 90 days (which may be unscheduled or scheduled visit) after the MRI features were first identified. They will continue with study drug treatment if their ARIA-E does not become severe radiologically and remains asymptomatic.

Those subjects who develop symptomatic or radiographically severe treatment emergent ARIA E will be temporarily stopped from study drug administration until the ARIA-E resolves radiographically, and will be followed up to resolution of the event (including symptoms, if any). They will undertake an unscheduled safety visit (with MRI) at approximately 30 days and another safety visit (with MRI) at approximately 90 days (which may be unscheduled or scheduled visit) after the symptomatic or severe ARIA-E was first identified. Thereafter, these subjects will have a safety visit (with MRI) at approximately 30 days (which may be an unscheduled or a scheduled visit) until the ARIA-E has resolved both radiologically and clinically. During treatment interruption, time will elapse on the Schedule of Assessments when the scheduled visit would otherwise occur. Once the ARIA-E has resolved both radiologically

and clinically, they may then resume treatment for the study duration and study assessments on the Schedule of Assessments. Resumption of treatment following symptomatic ARIA-E can only occur twice, after which the subject must be discontinued from the study. (revised per Amendment 12)

Radiographic severity is defined as follows: (revised per Amendent 12)

No ARIA-E present: No signs of ARIA-E.

Questionable ARIA-E: Subtle sulcal or cortical fluid-attenuated inversion recovery (FLAIR) hyperintensity, most likely artifactual.

Mild ARIA-E: Mild FLAIR hyperintensity confined to sulcus and/or cortex/subcortex white matter (with or without gyral swelling and sulcal effacement), which affects an area of less than 5 cm in a single greatest dimension. Only a single region of involvement detected.

Moderate ARIA-E: Moderate involvement (area of FLAIR hyperintensity measuring 5-10 cm in single greatest dimensions), or more than one site of involvement, each measuring less than 10 cm in a single greatest dimension.

Severe ARIA-E: Severe involvement (area of FLAIR hyperintensity measuring greater than 10 cm in single greatest dimension [white matter and/or sulcal involvement with associated gyral swelling and sulcal effacement)). One or more separate/independent sites of involvement may be noted.

Should a subject discontinue from study treatment from one of these events, such subjects will undergo the Early Termination Visit within 7 days of discontinuation and will undergo the 3 month Follow Up Visit per protocol. These subjects will continue to be followed with safety MRIs on a monthly basis thereafter, until the finding has either resolved or stabilized.

An end-of-study assessment will be performed for subject who complete treatment in the Extension Phase (Visit 96). Subjects may discontinue from study drug for any reason in the Extension Phase. Subjects who discontinue study drug early must comply with the Early Termination Visit (within 7 days after the last dose of study drug) during which end-of-study assessments will be performed. Any subjects who consent to participation in the PET subtudy will have those assessments performed at the Early Termination visit. Subjects who discontinue study drug early may also have unscheduled visits for safety assessments as needed. The Follow-Up Visit will take place 3 months after the last dose of study drug for all subjects.

Table 11 Summary of Blood Sample Volumes in the Extension Phase

Assessment	Total Number of Collection Timepoints	Sample Volume, Number of Timepoints x Volume per Collection (ml)	Total Volume (ml)
		Extension Phase Treatment and Follow-up Periods	
Clinical laboratory tests	11	11 x 10 mL	110
Blood sampling for serum anti-BAN2401	10	10 x 10 mL	100
Blood sampling for serum BAN2401 PK	11	11 x 3 mL	33
Bloof for exploratory biomarker analysis	5	5 x 6 mL	30
All blood samples, total volume collected			273

PK = pharmacokinetic

STUDY DRUG SUPPLIES

A single dose of BAN240110 mg/kg biweekly will be administered.

Removal of Subjects from Therapy or Assessment

Subjects who discontinue from treatment during the Extension Phase will have a Follow-Up visit 3 months after the last dose of study drug.

Safety-related criteria for discontinuation of study drug are as follows:

- Infusion reactions associated with administration of study drug, of Grade 3 severity or above (as defined in the NCI-CTCAE) that do not lessen or resolve with treatment
- Clinical features which indicate meningoencephalitis (eg, combination of 1 or more of the following: headache, worsening confusion, neck stiffness, impaired consciousness, focal neurological signs)
- Hypersensitivity reactions with clinical features of tissue injury (eg, arthritis, glomerulonephritis, mononeuritis multiplex)

Treatment Administered and Identity of Investigational Product(s)

All subjects will receive BAN2401 10 mg/kg biweekly dose during the Extension Phase.

BAN2401 is a humanized IgG1 monoclonal antibody that binds to soluble Aβ aggregates. BAN2401 is manufactured at Patheon Ltd, United Kingdom (UK) and at Biogen, RTP, under contract to Eisai. It is currently supplied as a sterile clear solution for injection containing 10 mg/mL, in a single use 10-mL vial (total 100 mg/vial). The drug product is formulated in 25 mM sodium citrate, 125 mM sodium chloride, 0.02% (w/v) polysorbate 80 and has a pH of

5.7. This current formulation of the drug product may be progressively phased out as stocks near the end of their shelf life and replaced by a newer formulation containing 100mg/mL BAN2401. The newer formulation (100 mg/mL BAN2401) was developed to reduce the number of vials of drug product required per dose and to reduce demand for storage space in a pharmacy. The newer (100 mg/mL BAN2401) formulation is supplied as a sterile aqueous solution containing 100 mg/mL BAN2401, 50 mmol/L citric acid, 0.05% (w/v) polysorbate 80, 350 mmol/L arginine, at pH 5.0, in glass vials containing 5 mL solution. The phasing out of the older formulation will only occur after appropriate regulatory authority approval for the newer formulation has been obtained, and adequate training of any pharmacy staff involved in the preparation of the final drug product for infusion has been completed. The appearance of the 2 different formulations after dilution in normal saline is identical.

Study drug will be packaged such that there is 1 vial per dispenser, and will be dispensed by a pharmacist at each site according to the the Interactive Voice Response System (IVRS). At the time that the study drug is dispensed for administration to the subject, the tear-off label on the dispenser must be removed and placed in the study drug accountability log at the time of dispensing.

Prohibited Concomitant Therapies and Drugs

A list of restricted and permitted drugs is provided in Appendix 2.

- The following therapies are not permitted for the listed intervals from Extension Baseline (Extension Screening Visit) until the Follow-Up Visit (Visit 97 [Extension Week 117]).
    - Immunoglobulin therapy, 6 months
    - Biologic drugs (except BAN2401), 6 months
- Subjects who require treatment with thrombolytic drugs do not have to be discontinued from the study, but study drug will be temporarily suspended for these subjects during thrombolytic therapy until stabilization or resolution of the medical condition that required thrombolytic drug treatment.
- Subjects who are on anticoagulants at Extension Baseline (Extension Screening Visit) are required to have their anticoagulation status optimized and stable for at least 4 weeks before Extension Screening Visit.
- Subjects who initiate AD treatment or who adjust their AD dosing regimen while on study will continue to study completion.

The following requirements apply to all other medications:

- Subjects must be on a stable dose for at least 4 weeks prior to Extension Screening Visit, except for medications which are administered as short courses of treatment (eg, anti-infectives) or which are to be used as needed (PRN).
- Medications which are used on a PRN basis or as a short course of treatment, and which are CNS active and may affect cognitive function are not permitted during a period of 72 hours prior to cognitive testing.

- Subjects may receive PRN prophylactic medications prior to infusion of study drug (outlined in the protocol) to minimize the risk of immunologic reaction or infusion reaction during or shortly after the infusion.
- Subjects who initiate treatment or undertake dose adjustment with drugs not intended for treatment of cognitive impairment during the study may continue in the study if in the opinion of the principal investigator this adjustment will not interfere with study procedures or subject safety and are not prohibited above.

SCHEDULE OF PROCEDURES/ASSESSMENTS

Table 12 presents the Schedule of Procedures/Assessments for the Extension Phase.

Table 12 Schedule of Procedures/Assessments for Study BAN2401-G000-201: Extension Phase (revised per Amendment 12)

Phase		Extension (Visit 44 through Visit 75, Extension Phase Week 1 through Extension Phase Week Phase 63)																					
Period	Screen	Treatment																					
Visit(s)[a]	Scrn	44	45	46	47	48	49	50	51,52	53	54, 55, 56	57	58, 59	60	61, 62	63	64, 65	66	67, 68, 69	70	71, 72	73	74, 75
Extension Phase Week(s)	0	1	3	5	7	9	11	13	15,17	19	21, 23, 25	27	29, 31	33	35, 37	39	41, 43	45	47, 49, 51	53	55, 57	59	61, 63
**Procedures/ Assessments**																							
Informed consent	X																						
Medical history	X																						
Inclusion/Exclusion	X																						
Vital signs[b]	X	X	X	X	X	X	X	X	X	X	X	X	X	X	X	X	X	X	X	X	X	X	X
Weight[c]		X						X				X				X				X			
Routine physical exam	X											X								X			
12-lead ECG	X											X								X			
Serum pregnancy test[d]	X																						
Urine pregnancy test[d]		X						X				X				X				X			
Blood for laboratory tests[e]	X[l]		X		X			X		X		X								X			
Urinalysis	X		X		X			X		X		X								X			
Urine Drug Screen	X																						
MMSE[f]	X											X								X			
CDR[f]	X											X								X			
ADAS-Cog[f]	X											X								X			
C-SSRS	X											X								X			
Safety MRI[g]	X					X		X				X								X			
Volumetric MRI[h]	X					X		X				X								X			
Amyloid PET[i]	X							X[m]				X[m]								X			
Study drug administration		X	X	X	X	X	X	X	X	X	X	X	X	X	X	X	X	X	X	X	X	X	X
Blood for serum BAN2401 PK[j]		X	X			X		X				X				X				X			
Blood for exploratory PD biomarker analysis	X							X				X								X			
Blood for serum anti-BAN2401[k]		X	X			X		X				X				X				X			
Prior / concomitant meds	X				X			X		X		X		X		X		X		X		X	
Adverse events	X	X	X	X	X	X	X	X	X	X	X	X	X	X	X	X	X	X	X	X	X	X	X

Phase	Extension (Visit 76 through Visit 97, Extension Phase Week 65 through Extension Phase Week 117)															
**Period**	**Treatment**														**Follow-up**	
**Visit(s)[a]**	**76**	**77, 78**	**79**	**80, 81, 82**	**83**	**84, 85**	**86**	**87, 88**	**89**	**90, 91**	**92**	**93, 94**	**95**	**96**	**Early Termination Visit[n]**	**97**	**Unscheduled Visit[o]**
**Extension Phase Week(s)**	**65**	**67, 69**	**71**	**73, 75, 77**	**79**	**81, 83**	**85**	**87, 89**	**91**	**93, 95**	**97**	**99, 101**	**103**	**105**		**117**	
**Procedures /Assessments**																	
Vital signs[b]	X	X	X	X	X	X	X	X	X	X	X	X	X	X	X	X	X
Weight[c]	X				X				X							X	
Routine physical exam					X									X	X	X	X
12-lead ECG					X									X	X	X	X
Urine pregnancy test[d]	X				X				X					X	X		X
Blood for laboratory tests[e]					X									X	X	X	X
Urinalysis					X									X	X	X	X
MMSE[f]					X									X	X	X	
CDR[f]					X									X	X	X	
ADAS-Cog[f]					X									X	X	X	
C-SSRS					X									X	X	X	X
Safety MRI[g]					X									X	X[g]	X	X
Volumetric MRI[h]					X									X	X	X	X
Amyloid PET[i]														X	X		
Study drug administration	X	X	X	X	X	X	X	X	X	X	X	X	X				
Blood for serum BAN2401 PK[j]					X								X	X	X	X	X
Blood for exploratory PD biomarker analysis														X	X		
Blood for serum anti-BAN2401[k]					X									X	X	X	X
Prior / concomitant meds	X		X		X		X		X		X		X		X	X	X
Adverse events	X	X	X	X	X	X	X	X	X	X	X	X	X	X	X	X	X

ADAS-Cog = Alzheimer's Disease Assessment Scale-Cognitive subscale, CDR = Clinical Dementia Rating, CDR-SB = Clinical Dementia Rating – Sum of Boxes, ECG = electrocardiogram, FAQ = Functional Assessment Questionnaire, IVRS = Interactive Voice Response System, MMSE = Mini-Mental State Examination, MRI = magnetic resonance imaging, PD = pharmacodynamic, PET = positron emission tomography, PK = pharmacokinetic.

a Visit 3 should be conducted no more than 10 days after completion of the Screening Visit and confirmation of eligibility to continue in the study. Assessments should take place on the first day of the study visit in the designated study week except as noted below (footnotes g, h, and i pertaining to imaging assessments). A visit window of ±8 days will be allowed for each visit. A visit window of ± 7 days will be allowed for the follow-up visit (Visit 97).

b Vital signs will be measured both at predose and after infusion. During Visits 44, 45, 46, and 47, vital signs should be recorded at least 2 hours after study drug infusion, in addition to predose. If at those visits no untoward effects of infusion on vital signs are detected ≥2 hours after infusion, these assessments at subsequent study visits may be conducted at a shorter interval after infusion. Vital sign measurements will consist of systolic and diastolic blood pressure (mmHg) measured after at least 3 minutes in a semi supine position, pulse (beats per minute), respiratory rate (per minute), and body temperature (in centigrade).

c Weight will be taken in the clinic at designated visits. If a subject misses a clinic visit where weight is to be collected, subsequent visits should use the most recent, previous collected weight for infusion calculations until the next clinic visit. Under such circumstances, weight is to be taken at the next clinic visit and entered into the IVRS even if the visit is not designated for weight data collection.

d Females of childbearing potential only. For all females of childbearing potential, an additional urine pregnancy test must be done on the day of Amyloid PET scanning.

e Blood for laboratory tests will be taken predose at all visits as indicated.

f Scales are to be completed in the morning (or, if not possible, consistently at the same time of day) in the following order on the days indicated: MMSE, CDR (including CDR-Global and CDR-SB), and ADAS-cog. Caregivers/informants (defined as a person able to support the subject for the duration of the study) need only to be present at visits where clinical assessments take place.

g MRI imaging must be completed within the Screening Period and before the first dose of study drug in the Extension Phase. During the Treatment Period, MRI imaging will be conducted on separate days from the scheduled visits. MRI imaging should be conducted at any time following the immediately preceding visit and prior to each of the following Visits for **ALL** subjects according to the Schedule of Procedures/Assessments. In all cases, the safety MRI must be reviewed by the imaging vendor and a local reader prior to a subject receiving the next dose of study drug. In the event of an Unscheduled Visit, the investigator in consultation with the sponsor will determine whether or not a safety MRI should be conducted. If an Early Termination Visit takes place, an MRI is to be conducted if not already performed during the preceding 90 days. (revised per Amendment 12)

h A volumetric MRI sequence will be collected in ALL subjects immediately following all safety MRI assessments.

i Before dosing in the Extension Phase, all subjects who underwent amyloid PET for inclusion in the Core Study should receive a baseline amyloid PET scan. In Japan, those who consented to the longitudinal imaging substudy will only undergo amyloid PET at Extension Phase Visit 70 [Extension Week 53], and Visit 96 [Extension Week 105]. The baseline amyloid PET scan must be conducted with the same imaging tracer that was used for inclusion at the baseline visit for the Core Study. During Screening, Amyloid PET imaging will be conducted within 10 days **before** the first dose of study drug in the Extension Phase for qualified subjects. During the Treatment Period, Amyloid PET imaging will be conducted on or within 10 days after the scheduled 3 month (Cohort 1 only), 6 month (Cohort 2 only), and 12 month (all subjects in the PET substudy), and 24 month Visits (all subjects in the PET substudy) in the Extension Phase (Visit 50 [Week 13; Cohort 1 only]; Visit 57 [Week 27; Cohort 2 only], Visit 70 [Week 53; all subjects in the PET substudy], Visit 96 [Week 105; all subjects in the PET substudy], and at the Early Termination Visit [all subjects in the PET substudy]). (revised per Amendment 12)

j At Visit 44, blood will be taken for the BAN2401 assay approximately 4 hours after the end of infusion (before subjects leave the clinic for home). Subjects must stay in clinic for the full 4 hours following infusion during this first infusion visit. Subjects must stay in clinic for at least 2 hours following infusion up through Visit 50. After Visit 50, if no untoward effects of infusion are noted, or infusion reactions can be prevented with prophylaxis, then subjects may be discharged from clinic 30 minutes after the end of infusion if judged medically stable by the investigator. Blood will be taken for the BAN2401 assay both predose and at least 2 hours after the end of infusion (before subjects leave the clinic for home). Subjects are required to remain in clinic for at least 2 hours following infusion at visits where PK samples are taken (except Visit 44, the first infusion visit). PK samples should be taken at least 2 hours after the end of infusion. These samples can be taken any time after those 2 hours and should generally be taken just prior to the subject leaving the site.

k Blood for the BAN2401 anti-drug antibody assay will be taken predose at the specified Visits. Blood for the BAN2401 anti-drug antibody assay will also be taken at the Early Termination Visit when applicable.

l Clotting screen (prothrombin time [PT, INR], activated partial thromboplastin time [APTT]) will be performed at the Screening Visit.

m Subjects will have PET amyloid testing at Visit 50 (Extension Week 13) or Visit 57 (Extension Week 27) based on stratification. Cohort 1 will have amyloid PET assessments performed at Visit 50 (Extension Week 13); Cohort 2 will have amyloid PET assessments performed at Visit 57 (Extension Week 27).

n Subjects who discontinue the study or study drug early must comply with the Early Termination Visit (within 7 days after discontinuation the last dose of study drug) and the Follow-Up Visit (3-months after the last dose of study drug). They may also have unscheduled visits for safety assessments.

o Unscheduled visits may be conducted at any time that safety or safety MRI data indicate per protocol or as clinically indicated in the judgment of the investigator. Note that assessments indicated under Unscheduled Visits need not always be conducted – actual assessments needed will be determined by the investigator and will be based on the specific visit needs.

STATISTICAL METHODS (revised per Amendment 11)

Primary Endpoint

- Safety assessments will be based on medical review of AE reports and the results of vital sign measurements, ECG, physical examinations, clinical laboratory tests, anti-drug antibody (ADA) test results, and any relevant test of cognitive function to evaluate decline. Additionally, MRI assessments of microhemorrhage, vasogenic edema, and other clinically significant abnormalities will be evaluated.

Secondary Endpoints

- Change from baselines in brain amyloid levels as measured by amyloid PET at 3 months (Visit 50 [Extension Week 13], Cohort 1) or 6 months (Visit 57 [Extension Week 27], Cohort 2), and 12 and 24 months in the Extension Phase (Visit 70 [Extension Week 53], Visit 96 [Extension Week 105])
- Change from end of Core Study in brain amyloid levels as measured by amyloid PET at the baseline of Extension Phase
- Proportion of amyloid positive subjects over time

Exploratory Endpoints

- Change from baselines in ADCOMS, CDR-SB, ADAS-Cog, and MMSE at each visit assessed
- Time to worsening of CDR global scores, eg, the worsening of global CDR score is defined as an increase from baseline by at least 0.5 points on the global CDR scale on 2 consecutive scheduled visits at which global CDR is measured
- Change from baselines in total hippocampal volume and other biomarkers at 12 and 24 months in the Extension Phase (Visit 70 [Extension Week 53], Visit 96 [Extension Week 105])

ANALYSIS SETS

- The **OLE Full Analysis Set (OLE-FAS)** is the group of subjects who receive at least 1 dose of study drug during the Extension Phase and who have a baseline assessment and at least 1 postdose ADCOMS measurement during the Extension Phase. The baseline assessment is defined as the last measurement prior to first dose of BAN2401 in the Extension Phase.
- The **OLE Safety Analysis Set** is the group of subjects who receive at least 1 dose of study drug during the Extension Phase and have at least 1 postdose safety assessment during the Extension Phase.
- The **OLE PK Analysis Set** is the group of subjects with at least 1 quantifiable BAN2401 serum concentration with a documented dosing history during the Extension Phase.
- The **OLE PD Analysis Set** is the group of subjects who have sufficient PD data to derive at least 1 PD parameter during the Extension Phase.

EFFICACY ANALYSES

An MMRM will be used to analyze the change from baseline secondary efficacy endpoints. Proporation of amyloid positive subjects over time will be summarized.

will also be used to analyze the following exploratory endpoints:

- Change from baseline in the ADCOMS, CDR-SB, ADAS-Cog score, and MMSE score

For each endpoint at a scheduled visit in the Extension Phase, a 90% 1-sided confidence interval will be constructed to test the null hypothesis that the treatment difference between subjects who took placebo in Core Study and those who took 10 mg/kg biweekly in the Core Study is no more than 50% of the treatment difference at the end of Core Study. If the lower limit of this confidence interval is greater than 0, the null hypothesis is rejected. In addition, comparisons between subjects who took placebo at Core Study and those who took 10 mg/kg biweekly in the Core Study will be performed for each endpoint at scheduled visits in the Extension Phase.

Time to worsening of CDR global scores will be analyzed on the FAS using Cox regression model. Time to worsening of CDR global score is defined as time from the first treatment in the Extension Phase to worsening of the CDR score (ie, the first worsening in 2 consecutive scheduled visits). For subjects whose CDR scores have not worsened by the end of study, the time to worsening of the CDR score will be censored at the date of last CDR assessment for these subjects.

Change from baseline in total hippocampal volume and other biomarkers will be summarized using descriptive statistics at scheduled visits in the Extension Phase.

Additional analyses and summaries will be performed as appropriate.

SAFETY ANALYSES

Safety analysis will be performed similarly to the Core Study. All safety analyses will be based on Safety Analysis Set of the Extension Phase. The incidence of TEAE, laboratory test variables, ECG findings, vital signs, brain MRI, ADAs will be summarized using the descriptive statistics. (See Section 9.7.1.8)

EXPLORATORY ANALYSES

No hypothesis testing will be performed for efficacy variables. Descriptive summary statistics will be generated for the efficacy endpoints as follows:

- Continuous variables will be summarized by number, mean, median, maximum, and minimum
- Categorical variables will be summarized by number and percentage

Additional summary/analyses will be performed using appropriate statistical methodologies if deemed necessary.

SAMPLE SIZE RATIONALE

There is no sample size calculation for the Extension Phase.

PROTOCOL SIGNATURE PAGE

(revised per Amendment 01)

**Study Protocol Number:**	BAN2401-G000-201
**Study Protocol Title:**	A Placebo-Controlled, Double-Blind, Parallel-Group, Bayesian Adaptive Randomization Design and Dose Regimen-finding Study with an Open Label-Extension Phase to Evaluate Safety, Tolerability and Efficacy of BAN2401 in Subjects With Early Alzheimer's Disease
**Investigational Product Name:**	BAN2401
**IND Number:**	105081
**EudraCT Number:**	2012-002843-11

SIGNATURES
Authors:

Chad Swanson, PhD<br>Study Director, Clinical Development<br>Neuroscience & General Medicine PCU<br>Eisai Inc.	Date
Robert Lai, MA, MB BChir, PhD, MRCP, FFPM<br>Medical Monitor & Clinical Pharmacologist<br>Clinical Development, Neuroscience & General Medicine PCU<br>Eisai Ltd.	Date
Jinping Wang, PhD<br>Director, Biostatistics<br>Neuroscience & General Medicine PCU<br>Eisai Inc.	Date

INVESTIGATOR SIGNATURE PAGE

**Study Protocol Number:**	BAN2401-G000-201
**Study Protocol Title:**	A Placebo-Controlled, Double-Blind, Parallel-Group, Bayesian Adaptive Randomization Design and Dose Regimen-finding Study with an Open-Label Extension Phase to Evaluate Safety, Tolerability and Efficacy of BAN2401 in Subjects With Early Alzheimer's Disease
**Investigational Product Name:**	BAN2401
**IND Number:**	105081
**EudraCT Number:**	2012-002843-11

I have read this protocol and agree to conduct this trial in accordance with all stipulations of the protocol and in accordance with International Conference on Harmonisation of Technical Requirements for Registration of Pharmaceuticals for Human Use (ICH) and all applicable local Good Clinical Practice (GCP) guidelines, including the Declaration of Helsinki.

Medical Institution

Investigator	Signature	Date
