# Supplementary material for: A randomized, double-blind, phase 2b proof-of-concept clinical trial in early Alzheimer’s disease with lecanemab, an anti-Aβ protofibril antibody
Source: Alzheimers Res Ther. 2021 Apr 17;13:80. doi: 10.1186/s13195-021-00813-8 (PMC8053280; doi:10.1186/s13195-021-00813-8)
Supplement: Supplementary file 1 — Additional file 1: Supplemental Figure S1. CONSORT 2010 Flow Diagram. Supplemental Figure S2. Change from Baseline for all Treatment Groups in the Alzheimer's Disease Composite Score (ADCOMS). Supplemental Figure S3. Results for ADAS-cog (3SA) and CDR-SB (3SB) for All Dosing groups. Supplemental Figure S4. Results for Total Hippocampal Volume (S4A), Whole Brain Volume (S4B), and Ventricular Volume for All Dosing Groups (S4C). Supplemental Figure S5. Change from Baseline in Neurogranin Measures (5SA). Change from Baseline in Neurofilament Light Chain Measures (5SB). Supplemental Table S1. Baseline Characteristics for Completers – Full Analysis Set. Supplemental Table S2. Baseline Characteristics for Subjects who Discontinued Treatment – Full Analysis Set. Supplemental Table S3. Bayesian Analysis of ADCOMS at 18 Months – Full Analysis Set. Supplemental Table S4. Summary of MMRM Analyses for Change from Baseline in ADCOMS at 12 Months – Full Analysis Set. Supplemental Table S5. Summary of MMRM Analyses for Change from Baseline in ADCOMS at 18 Months – Full Analysis Set. Supplemental Table S6. Bayesian Analysis of CDR-SB at 18 Months – Full Analysis Set. Supplemental Table S7. Summary of MMRM Analyses for Change from Baseline in CDR-SB at 18 Months – Full Analysis Set. Supplemental Table S8. Bayesian Analysis of ADAS-Cog14 at 18 Months – Full Analysis Set. Supplemental Table S9. Summary of MMRM Analyses for Change from Baseline in ADAS-Cog14 at 18 Months – Full Analysis Set. Supplemental Table S10. Summary of MMRM Analyses for ADCOMS at 18 Months for Disease Stage (MCI due to AD and Mild AD Dementia) Subgroups - Full Analysis Set. Supplemental Table S11. Summary of MMRM Analyses for ADAS–Cog14 at 18 Months for Disease Stage (MCI due to AD and Mild AD Dementia) – Full Analysis Set. Supplemental Table S12. Summary of MMRM Analyses for CDR-SB at 18 Months for Disease Stage (MCI due to AD and Mild AD Dementia) – Full Analysis Set. Supplemental Table S13. Summary of MMRM Ana [file 13195_2021_813_MOESM1_ESM.zip › simulation plan.pdf]

APPENDIX 6

Berry Consultants
Statistical Innovation

Phase II Dose Finding Study of BAN2401 in Patients with Mild Cognitive Impairment Due to Alzheimer's Disease and Mild Alzheimer's Disease

Submitted to Eisai
June 25, 2012

1. TABLE OF CONTENTS

1.0 INTRODUCTION 3
2.0 STATISTICAL MODELING 3
2.1 Primary Endpoint Modeling 3
2.2 Dose-Response Model Primary 3
2.3 Longitudinal Model 5
3.0 POSTERIOR QUANTITIES 6
3.1 Definition of Doses 6
3.2 Probability Superior to Control 6
3.3 Probability Superior to Control by Clinically Significant Difference 6
3.4 Probability of Phase III Success 7
4.0 ALLOCATION 7
5.0 EVALUATION OF TRIAL SUCCESS AND FUTILITY 8
5.1 Early Futility 8
5.2 Early Success 9
5.3 Trial Completion 9
5.4 Consideration of Phase III 9
5.5 Superior to Control 9
6.0 ACCRUAL AND DROPOUT 10
7.0 EXAMPLE TRIAL 10
8.0 SIMULATION SCENARIOS 15
8.1 Longitudinal Scenarios 15
8.2 Response Scenarios 16
9.0 OPERATING CHARACTERISTICS 16
10.0 CONCLUSIONS 20
APPENDIX FOR LONGITUDINAL PROFILES 21
APPENDIX FOR DROPOUT RATES 23

2. 1.0 INTRODUCTION

This is a phase II dose-finding study of BAN2401 for the treatment of Alzheimer's disease. The minimum sample size will be 196 patients and the maximum sample size will be 800 patients. We use response adaptive randomization to allocate patients between placebo control and 5 active doses with the goal of characterizing the dose response curve. The study will be monitored for early success and early futility. There are two schedules of administration, bi-weekly and once monthly. The primary endpoint is the change in a composite clinical score from baseline to 52 weeks. A difference from control of at least 0.03 would be considered a clinically significant difference (CSD).

3. 2.0 STATISTICAL MODELING

3.1 2.1 Primary Endpoint Modeling

The primary endpoint is the change from baseline to 52 weeks in a composite clinical score. We label the observations of the change from baseline in composite clinical score for subject *i*, at the 6, 12, 26, 39, and 52 week visits as $Y_{i,6}$, $Y_{i,12}$, $Y_{i,26}$, $Y_{i,39}$, and $Y_{i,52}$, respectively. We model the 52-week primary outcomes as normally distributed,

$$[Y_{i,52}] \sim N(\theta_d, \sigma^2),$$

where d_i is the treatment arm for subject *i*. The treatment arms are: d=0 (control), *d*=1 (3 mg/kg bi-weekly), *d*=2 (5 mg/kg bi-weekly), *d*=3 (10 mg/kg bi-weekly), *d*=4 (5 mg/kg monthly), and *d*=5 (10 mg/kg monthly).

3.2 2.2 Dose-Response Model Primary

We label the mean change from baseline to 52-weeks for dose group *d* as θ_d. We construct a dose-response model for the mean change from baseline for each treatment arm. The arms are modeled with a two-dimensional first-order normal dynamic linear model (NDLM). This model is a Gaussian random walk model. The structure is

$$[\theta_0] \sim N(0, 0.5^2)$$
$$[\theta_1] \sim N(0, 0.5^2)$$

and the NDLM structure for the bi-monthly doses:

$$[\theta_2] \sim N(\theta_1, \tau^2)$$

$$[\theta_3] \sim N(\theta_2, \tau^2)$$

The monthly doses are "connected" to the dose-response model through the respective bi-monthly doses. The connection through the NDLM first order structure results in the following priors

$$[\theta_4] \sim N(\theta_2, \tau^2)$$

and

$$[\theta_5] \sim N\left(\frac{\theta_3 + \theta_4}{2}, \frac{\tau^2}{2}\right).$$

The drift parameter (variance component), τ^2 is modeled with the following prior distributions:

$$[\tau^2] \sim IG(0.25, 0.0025)$$

where *IG(a,b)* is the inverse gamma distribution with shape parameter *a* and scale parameter *b*. This prior has the equivalent weight of 0.5 observations worth of weight that the value of τ is 0.1.

The prior distribution on the initial dose is selected to be a vague prior. An effect size of 0.03 is considered clinically significant and the standard error of the clinical scale is 0.15. Therefore these priors are quite weak and allow the data to shape the dose-response model. The prior for the "drift parameter" is selected to have minor weight that there will be similarity across the doses, and hence a minor amount of borrowing. With 5 doses the amount of borrowing is determined largely by the similarity in outcomes across doses.

The distribution of the variance of the primary endpoint, σ^2, is modeled with the following prior:

$$[\sigma^2] \sim IG(2.5, 0.056).$$

The prior is equivalent to a prior weight of 5 observations worth of information that the value of σ is 0.15. The value of 0.15 is based on initial modeling by Eisai. This prior allows the historical data estimate of 0.15 to have some role in the early part of the trial. When 300 subjects have been enrolled this prior will have a very minor role (5/305 weight is less than 2%).

3.3 2.3 LONGITUDINAL MODEL

At each interim analysis there will be subjects who have complete or incomplete information. Some subjects will have complete information meaning they have a 52-week value observed, $Y_{i,52}$. These subjects may also have their earlier values observed, $Y_{i,6}$, $Y_{i,12}$, $Y_{i,26}$, and $Y_{i,39}$. There will be subjects with earlier observations, but no 52-week observation. There will be subjects with no observations.

We utilize the information from subjects with incomplete information to the extent that the earlier values are correlated to the final 52-week value. A Bayesian model is built to learn from the accruing information on the association between the early values and the final endpoint at 52 weeks. Different sets of parameters (instances) are used for each arm in the trial, but the models correlating early to late outcomes are the same.

A linear regression model is created for the correlation between the 6-, 12-, 26-, and 39-week values and the 52-week value (we suppress the subject index *i* and refer to the change from baseline at weeks 6, 12, 26, 39, and 52 as Y_6, Y_{12}, Y_{26}, Y_{39}, and Z_{52}). A common model is used for each active dose and a separate model for the control. Each of the model instances is identical, with the following structure for early time period j:

$$\left[Z_{52} \mid Y_j\right] \sim N\left(\alpha_j + \beta_j Y_j, \lambda_j^2\right)$$
$$\left[\alpha_j\right] \sim N\left(0, 0.05^2\right)$$
$$\left[\beta_j\right] \sim N\left(0.80, 0.25^2\right)$$
$$\left[\lambda_j^2\right] \sim IG\left(2.5, 0.025\right).$$

The prior distribution for the standard deviation about the linear regression, λ, has a prior mean estimate of 0.10 with a weight of 5 observations. The joint posterior distribution of α_j, β_j, and λ_j , for j=6, 12, 26, and 39, are updated at each interim analysis (as described in Section 4.0) with the observed values of Y_{52} .

These prior distributions were selected from historical data provided by Eisai. The data were discounted to allow the new study to shape the posteriors, but these priors provide empirically based starting values.

At each interim analysis (as described in Section 4.0), this model is used, with Bayesian imputation within Markov chain Monte Carlo, to update the dose-response model at each interim analysis. The Markov chain Monte Carlo steps are as follows:

1) Simulate longitudinal model parameters from the updated joint posterior distribution
2) For patients that do not yet have a 52 week observation, impute the 52 week observation based on their most recent observation (6, 12, 26, or 39 weeks)
3) Simulate dose-response model parameters for each arm based on the "complete" (observed and imputed) 52-week data.
4) Repeat

4. 3.0 POSTERIOR QUANTITIES

4.1 3.1 DEFINITION OF DOSES

We characterize the dose-response curve by defining the maximum effective dose (d_{Max}) and the effective dose 90 (d_{ED90}). The maximum effective dose (d_{Max}) is the dose with the greatest treatment effect (greatest difference from control in mean change from baseline to 52 weeks). The d_{ED90} is the simplest dose that achieves at least 90% of the treatment effect achieved by d_{Max}. The simplest dose level is the smallest dose level with the lowest frequency of administration. The dose levels ordered by frequency of administration and amount are 5QM, 10QM, 3BM, 5BM, and 10BM. The doses identified as the d_{Max} and the d_{ED90} may be different doses or the same dose. For example, if only one dose is effective, it would be both the d_{Max} and d_{ED90}.

We estimate the probability each dose is d_{Max}, $\Pr(d = d_{Max})$, and the probability each dose is the d_{ED90}, $\Pr(d = d_{ED90})$. Based on the posterior distributions of the parameters in the dose response model, we sample 10,000 dose-response curves. We identify the d_{Max} and the d_{ED90} for each of these curves. The proportion of simulated dose-response curves where $d = d_{Max}$ is the $\Pr(d = d_{Max})$ for a given dose d. Similarly, the proportion of simulated dose-response curves where $d = d_{ED90}$ is the $\Pr(d = d_{ED90})$ for a given dose d.

4.2 3.2 PROBABILITY SUPERIOR TO CONTROL

For each dose we calculate the probability of being superior to control by comparing the posterior distribution of the mean change from baseline to 52-weeks between the active dose and the control.

We determine the probability of being superior to control by randomly drawing 10,000 treatment effects from the posterior distribution of θ_d for the active dose, and a matching 10,000 samples from the posterior distribution of θ_0 (treatment effects for the control). Thus, we have 10,000 pairs of active dose and control treatment effects. The proportion of pairs where the treatment effect for the active dose is greater than for control is the probability of being superior to control, $\Pr(\theta_d > \theta_0)$.

4.3

4.4 3.3 PROBABILITY SUPERIOR TO CONTROL BY CLINICALLY SIGNIFICANT DIFFERENCE

Similar to above in Section 3.2, we calculate the probability of being superior to control by at least the clinically significant difference (CSD) of 0.03 by comparing the posterior

distribution of the mean change from baseline to 52 weeks between the active dose and the control.

We determine the probability of being superior to control by at the least the CSD by sampling 10,000 treatment effects from the posterior distribution of θ_d for the active dose, and a matching 10,000 samples from the posterior distribution of θ_0 (treatment effect for the control). Thus, we have 10,000 pairs of active dose and control treatment effects. The proportion of pairs where the treatment effect for the active dose is greater than for control by at least 0.03 is the probability of being superior to control by at least the CSD, $\Pr(\theta_d > \theta_0 + 0.03)$.

4.5 3.4 Probability of Phase III Success

For each active dose, we calculate the predictive probability of success in a phase III trial. We assume a hypothetical future phase III trial with a fixed design that would equally randomize 500 patients between control and an active dose (250 per arm). The predictive probability of phase III success is the chance of statistical significance ($\Pr(\theta_0 > \theta_d) < 0.025$) versus control if the active dose was entered into the hypothetical future phase III trial.

This is different from the power for such a trial, in that power calculations typically assume a fixed treatment effect whereas the predictive probability of success averages the power over the posterior distribution of the treatment effect. Thus knowledge of the treatment effect and the uncertainty in that knowledge are formally incorporated.

We determine the predictive probability of phase III success for a dose, *d*, by randomly sampling one value from the posterior distribution of the treatment effect for that dose and a corresponding random sample from the posterior distribution of the treatment effect for control. Assuming these treatment effects, we calculate the corresponding power (i.e., the probability of phase III success or the probability that $\Pr(\theta_0 > \theta_d) < 0.025$ given the randomly sampled treatment effects) and record that result. We repeat this 10,000 times. The average of the resulting probabilities of phase III success is the predictive probability of phase III success.

5. 4.0 ALLOCATION

There will be an initial burn-in period in which patients will be allocated in blocks of 14 with 4:2:2:2:2:2 blocked to control and each of the 5 active dose arms respectively. There will be a total of 14 blocks and so a total of 196 patients will be randomized with 56 on the control and 28 on each of the active doses.

After this initial burn-in, adaptive randomization will begin. Adaptive randomization probabilities will be updated when 196, 250, 300, 350, 400, 450, 500, 550, 600, 650, 700,

and 750 patients have been enrolled. The randomization probability for each of the 5 active doses will be weighted according to the variance components,

$$V_d = \Pr(d = d_{ED90})\sqrt{\frac{Var(\theta_d)}{n_d + 1}} \text{ for d = 1, 2, 3, 4, 5}$$

where $\Pr(d = d_{ED90})$ is the probability the dose is the ED90, $Var(\theta_d)$ is the posterior variance of the treatment effect, and n_d is the current number of subjects allocated to dose *d*.

The randomization probability to the control is meant to mirror the randomization probability to the most likely ED90 dose. The randomization probability for the control is weighted according to

$$V_0 = \min\left\{\sum_{d=1}^{5} V_d\left(\frac{n_d}{n_d + n_0}\right), \max(V_1, V_2, V_3, V_4, V_5)\right\} \text{ for d = 0}$$

The randomization vector is then

$$q_d = \frac{V_d}{\sum_{j=0}^{5} V_j} \text{ for d = 0,1,2,3,4,5}$$

6. 5.0 EVALUATION OF TRIAL SUCCESS AND FUTILITY

Interim analyses are planned beginning when 196 patients are enrolled and after approximately every additional 50 patients are enrolled (after 250, 300, 350, etc) as described in Section 4.0. If the trial should continue to the maximum sample size of 800 patients, post accrual interim analyses are planned 3, 6, and 9 months after the 800th patient is enrolled. If the trial is not stopped early for futility, all enrolled patients will be followed for 18 months. The final evaluation of efficacy will occur when all enrolled patients have completed follow-up for the primary endpoint.

6.1 5.1 Early Futility

Interim monitoring for early futility will occur at each interim analysis during and post accrual. Interim monitoring for futility will be based on the dose identified as the ED90 (d_{ED90}). If, at the first three interim looks (when 196, 250, and 300 patients have been enrolled), there is less than a 5% probability that the d_{ED90} achieves a clinically

significant difference from control, the trial will stop early for futility. Formally if:

$$\Pr(\theta_{dED90} > \theta_0 + 0.03) < 0.05.$$

Beginning at the 350 patient look and continuing to the completion of the trial (complete enrollment and complete follow-up), the futility criteria is increased to 7.5%. Thus, it becomes easier to stop for futility once at least 350 patients have been enrolled. Formally the trial will stop for futility at these later interim analyses if,

$$\Pr(\theta_{dED90} > \theta_0 + 0.03) < 0.075.$$

6.2 5.2 Early Success

Interim monitoring for early success will occur at each interim analysis beginning when 350 patients have been enrolled. If there is greater than a 95% probability that the d_{ED90} achieves a clinically significant difference from control, the trial will stop for early success. Formally if:

$$\Pr(\theta_{dED90} > \theta_0 + 0.03) > 0.95.$$

6.3 5.3 Trial Completion

If the trial is not stopped early, either during accrual or post accrual, for either futility or success, then trial success will be evaluated at the completion of the trial, when both accrual and follow-up for the primary endpoint are complete. If, at the completion of the trial, there is greater than an 80% probability that the d_{ED90} achieves a clinically significant difference from control, this trial will be considered a success. Formally,

$$\Pr(\theta_{dED90} > \theta_0 + 0.03) > 0.80$$

6.4 5.4 Consideration of Phase III

At the conclusion of the study, the d_{ED90} will be considered as promising for phase III if the predictive probability of success in a future phase III trial is at least 80%.

6.5 5.5 Superior to Control

At the conclusion of the study, the d_{ED90} will be considered superior to control if the posterior probability the d_{ED90} has greater mean change at 52-weeks is at least 97.5%.

7. 6.0 ACCRUAL AND DROPOUT

We assume accrual will increase at a constant rate over the first 6 months of the study to a peak accrual rate of 32 patients per month. The table below shows the approximate accrual rate at each month during the 6 month ramp-up to the steady state accrual rate of 32 patients per month.

Accrual Ramp-Up: Patients per Month	
**Week**	**Patients Per Month**
1	5.33
2	10.66
3	16
4	21.33
5	26.66
6	32
Steady State Patients per Month from 6 Months	32

We assume an exponential rate of dropout such that 22% of patients have dropped out by 26 weeks and 40% have dropped out by 52 weeks. As a sensitivity analysis we also consider dropout rates of 20% and 30% by 52 weeks.

8. 7.0 EXAMPLE TRIAL

In this section we present an example trial to illustrate the adaptive design.

The first interim analysis occurs when 196 patients have been enrolled in a 4:2:2:2:2:2 ratio to control and each of the active doses. Table 7.1.A shows the observed data at this interim analysis. Some patients are newly enrolled or have no follow-up data yet. The longest follow-up of any patient is 26 weeks. The 5Q, 10Q, and 3BM doses have the greatest observed mean changes at 26 weeks, but the 10Q dose has a worse result at 12

weeks. Thus, the 5Q and 3BM doses have similar probability of being the ED90 (Table 7.1.B). The 5Q dose, the most likely ED90 has a 38% probability of being better than control by the clinically significant difference (CSD). The early futility stopping boundary is not met and so accrual to the trial continues with the 5Q and 3BM doses receiving a higher probability of randomization for the next 50 patients to be enrolled.

Table 7.1.A: Observed Data at the Interim Analysis with 196 Pts Enrolled

		**No Data**	**6 Weeks**		**12 Weeks**		**26 Weeks**		**39 Weeks**		**52 Weeks**	
	**N**	**N**	**N**	**Obs**	**N**	**Obs**	**N**	**Obs**	**N**	**Obs**	**N**	**Obs**
PBO	56	12	15	0.01	26	-0.002	3	-0.085	0	0	0	0
5Q	28	7	7	-0.019	10	0.028	4	0.04	0	0	0	0
10Q	28	7	8	0.005	11	-0.02	2	0.083	0	0	0	0
3BM	28	10	7	-0.003	10	0.016	1	0.114	0	0	0	0
5BM	28	10	6	0.008	9	0.024	3	0.005	0	0	0	0
10BM	28	7	10	0.008	8	0.023	3	-0.005	0	0	0	0

Table 7.1.B: Model Estimates

	**Model Est**	**Pr(ED90)**	**Pr(PBO)**	**Pr(CSD)**
PBO	-0.003	-	-	-
5Q	0.012	0.251	0.605	0.377
10Q	0.002	0.149	0.533	0.309
3BM	0.011	0.247	0.589	0.375
5BM	0.013	0.177	0.617	0.378
10BM	0.011	0.176	0.606	0.379

At the next interim analysis when 250 patients have been enrolled (Table 7.2.A) most patients still have 26 weeks or less of follow-up. The observed data is slightly worse for the 3BM dose. The 5Q, 10Q, and 3BM doses all have similar probability of being the ED90 (Table 7.2.B). Again, the early futility stopping boundary is not met and so accrual to the trial continues with the 5Q, 10Q, and 3BM doses receiving a similar probability of randomization for the next 50 patients to be enrolled.

Table 7.2.A: Observed Data at the Interim Analysis with 250 Pts Enrolled

	**No**	**6 Weeks**	**12 Weeks**	**26 Weeks**	**39 Weeks**	**52 Weeks**

		Data										
	N	N	N	Obs	N	Obs	N	Obs	N	Obs	N	Obs
PBO	67	13	15	-0.001	31	0.005	8	-0.047	0	0	0	0
5Q	38	13	8	-0.006	12	0.016	5	0.031	0	0	0	0
10Q	35	9	8	0.00	12	-0.006	6	0.005	0	0	0	0
3BM	39	15	8	0.001	12	0.008	3	0.011	1	0.244	0	0
5BM	35	11	7	-0.005	13	0.018	4	0.006	0	0	0	0
10BM	36	10	8	0.006	13	0.01	5	0.02	0	0	0	0

Table 7.2.B: Model Estimates				
	Model Est	Pr(ED90)	Pr(PBO)	Pr(CSD)
PBO	-0.004	-	-	-
5Q	0.007	0.218	0.586	0.341
10Q	0.004	0.201	0.56	0.314
3BM	0.01	0.252	0.602	0.381
5BM	0.008	0.161	0.585	0.348
10BM	0.009	0.168	0.593	0.355

Interim analyses are conducted again after 300 and 350 patients are enrolled and results are similar. The possibility for early success stopping begins at the 350 patient interim analysis. At the interim analysis when 400 patients have been enrolled, patients are beginning to complete the 52 weeks of follow-up for the primary endpoint (Table 7.3.A). Each of the BM doses have a positive observed effect at 39 weeks, but the 3BM dose also has a strong effect at 12 weeks and a patient with a large effect at 52 weeks. The 3BM dose is the most likely ED90 (Table 7.3.B). The probability it is better than control by the CSD is 61%. Neither the early futility nor early success criteria are met. Accrual continues with the 3BM dose receiving a higher probability of randomization for the next 50 patients to be enrolled.

Table 7.3.A: Observed Data at the Interim Analysis with 400 Pts Enrolled												
		No Data	6 Weeks		12 Weeks		26 Weeks		39 Weeks		52 Weeks	
	N	N	N	Obs	N	Obs	N	Obs	N	Obs	N	Obs
PBO	101	14	17	0.005	34	0.005	27	-0.012	9	-0.043	0	0
5Q	63	12	11	-0.008	24	0.004	11	-0.001	4	-0.014	1	-0.001
10Q	55	10	8	-0.005	18	0.016	12	-0.002	6	-0.029	1	0.208
3BM	73	17	17	0.006	27	0.027	9	0.007	2	0.036	1	0.174
5BM	53	11	5	0.006	18	0.014	14	0.011	3	0.095	2	-0.056
10BM	55	7	11	0.006	17	0.007	13	0.012	6	0.086	1	0.085

Table 7.3.B: Model Estimates				
	Model Est	Pr(ED90)	Pr(PBO)	Pr(CSD)
PBO	-0.005	-	-	-
5Q	0.001	0.07	0.57	0.284
10Q	0.024	0.277	0.744	0.502
3BM	0.037	0.414	0.838	0.606
5BM	0.003	0.042	0.568	0.281
10BM	0.026	0.196	0.761	0.507

Results are similar at the next interim analysis when 450 patients are enrolled. At the interim analysis with 500 patients enrolled, the 10BM dose now has the largest effect and the randomization probabilities will shift such that the 10BM dose will receive a greater weight (Tables 7.4.A and 7.4.B).

Table 7.4.A: Observed Data at the Interim Analysis with 500 Pts Enrolled												
		No Data	6 Weeks		12 Weeks		26 Weeks		39 Weeks		52 Weeks	
	N	N	N	Obs	N	Obs	N	Obs	N	Obs	N	Obs
PBO	129	17	16	0	38	0.012	26	-0.005	24	-0.001	8	0.016
5Q	66	6	4	-0.015	24	0.005	18	0	9	0.009	5	-0.015
10Q	69	8	10	-0.004	18	0.005	17	0.044	10	0.036	6	-0.021
3BM	111	27	16	-0.006	40	0.018	17	0.052	8	0.026	3	0.099
5BM	55	6	0	0	15	0.026	19	0.024	10	0.04	5	0.042
10BM	70	9	10	0.008	15	0.02	20	0.039	10	0.007	6	0.119

Table 7.4.B: Model Estimates				
	Model Est	Pr(ED90)	Pr(PBO)	Pr(CSD)
PBO	0.017	-	-	-
5Q	-0.001	0.03	0.3	0.09
10Q	0.01	0.028	0.415	0.104
3BM	0.035	0.206	0.692	0.355
5BM	0.033	0.115	0.698	0.331
10BM	0.066	0.621	0.934	0.729

Interim analyses are conducted with 550, 600, and 650 patients are enrolled and results are similar. At the interim analysis with 700 patients enrolled, the 10BM dose has the largest sample size and remains the most likely ED90 (Tables 7.5.A and 7.5.B). The estimated mean change from baseline to 52 weeks is 0.006 for control and 0.084 for the 10BM dose. The probability the 10BM dose is better than control by at least the clinically significant difference is 0.957,which is greater than the 0.95 required to stop for early success. Accrual to the trial stops, but all currently enrolled patients will continue

to be followed.

Table 7.5.A: Observed Data at the Interim Analysis with 700 Pts Enrolled													
		No Data	6 Weeks		12 Weeks		26 Weeks		39 Weeks		52 Weeks	
	N	N	N	Obs	N	Obs	N	Obs	N	Obs	N	Obs
PBO	185	21	26	0.001	45	0.013	29	0.001	26	0.035	38	-0.006
5Q	72	6	4	-0.023	13	0.004	15	0.016	14	0	20	-0.022
10Q	80	9	6	0.006	10	-0.007	16	0.031	21	0.042	18	0.023
3BM	134	16	11	-0.004	38	0.004	34	0.026	20	0.015	15	0.093
5BM	69	8	4	0.014	13	0.002	12	0.037	14	0.052	18	0.027
10BM	160	30	34	0.009	44	0.011	16	0.028	17	0.054	19	0.084

Table 7.5.B: Model Estimates				
	Model Est	Pr(ED90)	Pr(PBO)	Pr(CSD)
PBO	0.006	0	0	0
5Q	-0.007	0.002	0.313	0.059
10Q	0.026	0.034	0.803	0.357
3BM	0.049	0.221	0.941	0.702
5BM	0.031	0.034	0.842	0.419
10BM	0.074	0.709	0.996	0.957

The final analysis is conducted when all enrolled patients have completed follow-up. Data is not complete on all patients due to dropout (Table 7.6.A). The observed change from baseline to 52 weeks is 0.014 for control and 0.068 for the 10BM dose. The model estimates are 0.012 for control and 0.067 for the 10BM dose (Table 7.6.B). The probability this dose is better than control by the clinically significant difference is 92%.

Table 7.6.A: Observed Data at the Final Analysis													
		No Data	6 Weeks		12 Weeks		26 Weeks		39 Weeks		52 Weeks	
	N	N	N	Obs	N	Obs	N	Obs	N	Obs	N	Obs
PBO	185	7	12	-0.002	22	0.009	16	0.011	14	-0.037	114	0.014
5Q	72	5	2	-0.029	11	0.005	8	0.006	2	0.212	44	0.005
10Q	80	6	3	-0.023	7	0.005	7	0.005	4	0.039	53	0.037
3BM	134	10	9	-0.005	17	0.007	9	0.015	15	0.016	74	0.041

5BM	69	5	1	0.031	7	0.014	9	0.019	5	0.003	42	0.041
10BM	160	9	9	0.013	15	0.005	23	0.035	11	0.077	93	0.068

Table 7.6.B: Model Estimates

	Model Est	Pr(ED90)	Pr(PBO)	Pr(CSD)
PBO	0.012	0	0	0
5Q	0.017	0.018	0.591	0.133
10Q	0.035	0.066	0.881	0.371
3BM	0.036	0.061	0.904	0.383
5BM	0.037	0.09	0.879	0.419
10BM	0.067	0.765	0.999	0.923

9. 8.0 SIMULATION SCENARIOS

In order to characterize the performance of the trial design, we simulated the trial considering different scenarios.

9.1 8.1 Longitudinal Scenarios

The changes in the composite clinical score observed at each visit are correlated within each patient. We assume a correlation of 0.80 from visit to visit. In addition, we make assumes for the fraction of the 52 week treatment effect that will be observed at each visit. We consider three scenarios, linear, symptomatic, and late onset. Table 8.1 shows the assumed fraction of the 52 week treatment effect at each visit. The linear scenario assumes a steady growth in the treatment effect through the 52 weeks, while the symptomatic scenario assumes the treatment effect will be observed relatively quickly, and the late onset scenario assumes the treatment effect will be observed later.

Table 8.1: Longitudinal Profiles

	Week 6	Week 12	Week 26	Week 39	Week 52
Linear	0.12	0.23	0.50	0.75	1
Symptomatic	0.11	0.93	1	1	1
Late Onset	0.12	0.12	0.20	0.50	1

9.2 8.2 Response Scenarios

We consider 13 scenarios for the treatment effect on each arm.

Table 8.2 Response Scenarios							
		**Control**	**3BM**	**5BM**	**10BM**	**5QM**	**10QM**
**1**	Null	0.00	0.00	0.00	0.00	0.00	0.00
**2**	Small Effect	0.00	0.01	0.02	0.03	0.01	0.02
**3**	BM Good	0.00	0.02	0.04	0.06	0.02	0.04
**4**	Q Low	0.00	0.03	0.06	0.10	0.01	0.03
**5**	Both Good	0.00	0.03	0.06	0.10	0.03	0.06
**6**	U-Shape Good	0.00	0.06	0.08	0.06	0.05	0.06
**7**	U-Shape Low	0.00	0.04	0.06	0.04	0.03	0.04
**8**	Small Increases	0.00	0.05	0.055	0.06	0.05	0.055
**9**	One Works	0.00	0.00	0.00	0.06	0.00	0.00
**10**	All 0.04	0.00	0.04	0.04	0.04	0.04	0.04
**11**	All 0.06	0.00	0.06	0.06	0.06	0.06	0.06
**12**	All 0.08	0.00	0.08	0.08	0.08	0.08	0.08
**13**	Medium Effect	0.00	0.015	0.03	0.04	0.015	0.03

10. 9.0 OPERATING CHARACTERISTICS

Table 9.1 shows the probabilities of trial success and futility and the mean total sample size for each scenario shown in Table 8.2. We present operating characteristics assuming the linear longitudinal profile and a 40% dropout rate at 52 weeks. Operating characteristics based on the late onset and symptomatic longitudinal profiles are shown in the appendix. Operating characteristics based on the linear longitudinal profile but assuming a lower (20% and 30%) dropout rate at 52 weeks are also shown in the appendix. Results are similar across the three longitudinal profiles and the three dropout rates.

We show the probability of early success, probability of early futility, the probability the trial continues to completion (complete accrual and complete follow-up) and the total probability of trial success (either early or at trial completion).

Table 9.1: Probabilities of Trial Success and Futility						
**Scenario**		**Mean N**	**Pr(Stop Early Success)**	**Pr(Stop Early Futility)**	**Pr(Go To Trial Completion)**	**Pr (Success)**
**1**	Null	683.1	0.119	0.447	0.433	0.125
**2**	Small Effect	697.4	0.281	0.157	0.562	0.353
**3**	BM Good	656.7	0.660	0.044	0.296	0.801
**4**	Q Low	573.1	0.962	0.019	0.019	0.978
**5**	Both Good	562.4	0.970	0.010	0.019	0.987
**6**	U-Shape Good	582.9	0.921	0.012	0.067	0.972
**7**	U-Shape Low	646.4	0.684	0.035	0.280	0.817
**8**	Small Increases	612.1	0.831	0.019	0.150	0.927
**9**	One Works	668.5	0.544	0.121	0.335	0.692
**10**	All 0.04	654.2	0.612	0.042	0.346	0.748
**11**	All 0.06	592.2	0.897	0.013	0.090	0.961
**12**	All 0.08	535.9	0.986	0.005	0.010	0.994
**13**	Medium Effect	683.1	0.416	0.090	0.495	0.537

We evaluate Type I error by the probability of trial success in the scenario that there is no treatment effect. In the "Null" scenario, there is a 12.5% probability of trial success. This is the one-sided Type I error rate of this trial.

We evaluate power by the probability of trial success in alternative scenarios. For example, in scenarios where all doses are better than control by at least the CSD, such as "U-Shape Good" or "Small Increases" there is greater than a 90% probability of trial success. In the "Small Effect" scenario, there is only a 35% probability of trial success, but the treatment effect for each dose is at or below the clinically significant difference.

Table 9.2 shows the mean and standard deviation of the number of patients allocated to each treatment arm. The true ED90 for each scenario is shown in bold italics. The true ED90 dose tends to receive the greatest number of patients in each scenario.

Table 9.2: Mean and Standard Deviation of the Number of Patients Allocated to Each Treatment Arm								
		**Mean Subjects (SD)**	**Mean Subj Control (SD)**	**Mean Subj 5Q (SD)**	**Mean Subj 10Q (SD)**	**Mean Subj 3BM (SD)**	**Mean Subj 5BM (SD)**	**Mean Subj 10BM (SD)**
**1**	Null	683.1 (154.7)	179.1 (43.2)	***106.6 (45.2)***	103.6 (44.3)	111.2 (45.9)	88.1 (38.8)	94.1 (41.1)
**2**	Small Effect	697.4 (147.7)	182.2 (40.9)	100.9 (39.4)	108.5 (43.3)	103.3 (40.3)	91.7 (38.8)	***110.8 (46.7)***
**3**	BM Good	656.7 (149.8)	170.6 (41.4)	89.1 (32.4)	104.9 (40.5)	90.4 (31.7)	87.7 (35.7)	***114.1 (46.3)***
**4**	Q Low	573.1 (126.8)	148.9 (35.7)	68.1 (18.0)	76.0 (23.1)	82.0 (25.4)	82.3 (32.3)	***115.8 (44.6)***
**5**	Both Good	562.4 (125.2)	144.8 (34.7)	74.4 (21.5)	88.9 (31.2)	75.4 (21.3)	74.1 (26.3)	***104.7 (39.4)***
**6**	U-Shape Good	582.9 (136.4)	150.1 (36.9)	86.3 (29.4)	88.6 (31.3)	94.5 (31.9)	***86.2 (33.1)***	77.3 (27.2)
**7**	U-Shape Low	646.4 (150.5)	167.8 (41.2)	93.4 (34.8)	97.2 (37.7)	104.1 (38.2)	***98.5 (41.0)***	85.4 (32.5)
**8**	Small Increases	612.1 (146.1)	158.2 (39.7)	96.4 (35.6)	***95.2 (35.7)***	96.5 (34.6)	79.5 (29.7)	86.3 (32.8)
**9**	One Works	668.5 (150.7)	177.1 (43.3)	86.3 (32.1)	86.9 (32.3)	91.1 (33.3)	73.9 (27.9)	***153.1 (62.4)***
**10**	All 0.04	654.2 (152.9)	170.3 (41.8)	***105.5 (40.9)***	101.5 (40.3)	104.9 (39.3)	83.6 (33.2)	88.5 (35.4)
**11**	All 0.06	592.2 (140.2)	153.0 (37.9)	***97.1 (35.2)***	91.6 (33.4)	95.0 (32.1)	75.8 (27.8)	79.6 (28.5)
**12**	All 0.08	535.9 (114.9)	137.6 (30.8)	***88.2 (28.7)***	82.7 (26.1)	86.9 (26.2)	68.9 (21.9)	71.6 (22.0)
**13**	Medium Effect	683.1 (150.2)	178.1 (41.5)	96.1 (36.1)	110.6 (44.8)	98.4 (37.1)	92.0 (39.1)	***107.8 (44.9)***

Table 9.3 shows the probability each dose is identified as the ED90. The true ED90 for each scenario is shown in bold italics.

**Table 9.3: Dose-Finding - Pr(ED90)**						
		**5Q**	**10Q**	**3BM**	**5BM**	**10BM**
1	Null	***0.23***	0.209	0.241	0.15	0.171
2	Small Effect	0.122	0.255	0.107	0.166	***0.35***
3	BM Good	0.049	0.232	0.04	0.136	***0.542***
4	Q Low	0.001	0.009	0.01	0.082	***0.898***
5	Both Good	0.012	0.117	0.006	0.06	***0.805***
6	U-Shape Good	0.132	0.207	0.202	***0.385***	0.074
7	U-Shape Low	0.11	0.197	0.19	***0.421***	0.082
8	Small Increases	0.252	***0.274***	0.164	0.134	0.177
9	One Works	0.011	0.013	0.015	0.01	***0.951***
10	All 0.04	***0.302***	0.233	0.225	0.127	0.113
11	All 0.06	***0.344***	0.234	0.219	0.104	0.1
12	All 0.08	***0.377***	0.243	0.199	0.096	0.085
13	Medium Effect	0.083	0.288	0.082	0.18	***0.366***

Table 9.4 shows the probability of success by arm for each scenario. This is the probability each arm achieves at least a 95% probability of being better than control by the CSD during the trial, or if the trial should run to completion, at least an 80% probability of being better than control by the CSD. The true ED90 for each scenario is shown in bold italics.

**Table 9.4: Probability of Success Early or at Trial Completion**						
		**5Q**	**10Q**	**3BM**	**5BM**	**10BM**
1	Null	***0.031***	0.025	0.03	0.017	0.022
2	Small Effect	0.048	0.082	0.044	0.052	***0.126***
3	BM Good	0.06	0.165	0.048	0.112	***0.415***
4	Q Low	0.018	0.05	0.044	0.139	***0.727***
5	Both Good	0.048	0.17	0.041	0.1	***0.627***
6	U-Shape Good	0.14	0.192	0.205	***0.316***	0.12
7	U-Shape Low	0.095	0.15	0.158	***0.316***	0.098
8	Small Increases	0.208	***0.219***	0.17	0.136	0.193
9	One Works	0.02	0.017	0.018	0.011	***0.626***
10	All 0.04	***0.19***	0.172	0.171	0.104	0.11
11	All 0.06	***0.277***	0.219	0.207	0.124	0.135
12	All 0.08	***0.285***	0.232	0.217	0.139	0.122
13	Medium Effect	0.055	0.138	0.052	0.093	***0.198***

Table 9.5 show the probability each dose will have at least an 80% predictive probability of phase III trial success. The true ED90 for each scenario is shown in bold italics.

Table 9.5: Predictive Probability of Phase III Success > 80%						
		**5Q**	**10Q**	**3BM**	**5BM**	**10BM**
1	Null	***0.011***	0.007	0.009	0.005	0.005
2	Small Effect	0.022	0.058	0.017	0.037	***0.101***
3	BM Good	0.027	0.157	0.024	0.094	***0.435***
4	Q Low	0.001	0.008	0.009	0.078	***0.888***
5	Both Good	0.011	0.113	0.006	0.057	***0.800***
6	U-Shape Good	0.126	0.194	0.194	***0.375***	0.071
7	U-Shape Low	0.075	0.138	0.141	***0.348***	0.059
8	Small Increases	0.222	***0.247***	0.146	0.12	0.161
9	One Works	0.003	0.003	0.003	0.003	***0.641***
10	All 0.04	***0.196***	0.16	0.151	0.084	0.078
11	All 0.06	***0.325***	0.219	0.207	0.098	0.095
12	All 0.08	***0.375***	0.241	0.199	0.096	0.084
13	Medium Effect	0.028	0.12	0.025	0.073	***0.182***

11. 10.0 CONCLUSIONS

With a minimum of 196 and a maximum of 800 patients, this design has a one-sided Type I error rate of approximately 12.5% and a high probability (at least 80%) of success for many scenarios in which the treatment would be considered effective. Adaptive allocation tends to allocate the greatest number of patients to the true ED90 and the true ED90 has a high probability of being identified for consideration in a future phase III trial.

Berry Consultants
Statistical Innovation

12. APPENDIX FOR LONGITUDINAL PROFILES

The table below summarizes the operating characteristics of the design assuming the late onset longitudinal profile. Results are similar to those assuming the linear longitudinal profile. The probability of success differs by only 1% to 2%. The delay in observing the treatment effect slightly reduces the probability of early success and slightly increases the probability of early futility.

Probabilities of Trial Success and Futility - Late Onset Longitudinal Profile						
**Scenario**		**Mean N**	**Pr(Stop Early Success)**	**Pr(Stop Early Futility)**	**Pr(Go To Trial Completion)**	**Pr (Success)**
**1**	Null	680.5	0.134	0.432	0.434	0.140
**2**	Small Effect	685.5	0.295	0.171	0.534	0.364
**3**	BM Good	652.9	0.631	0.068	0.301	0.773
**4**	Q Low	590.3	0.940	0.032	0.028	0.964
**5**	Both Good	578.8	0.950	0.025	0.026	0.971
**6**	U-Shape Good	594.9	0.912	0.020	0.068	0.964
**7**	U-Shape Low	646.5	0.661	0.052	0.286	0.796
**8**	Small Increases	618.5	0.810	0.032	0.158	0.911
**9**	One Works	668.5	0.534	0.138	0.328	0.678
**10**	All 0.04	653.8	0.599	0.060	0.342	0.734
**11**	All 0.06	602.5	0.872	0.023	0.104	0.947
**12**	All 0.08	550.4	0.982	0.010	0.008	0.988
**13**	Medium Effect	676.6	0.421	0.116	0.464	0.528

The table below summarizes the operating characteristics of the design assuming the symptomatic longitudinal profile. Results are similar to those assuming the linear longitudinal profile. Observing the full treatment effect earlier in the course of follow-up slightly increases the probability of early success in scenarios where the treatment would be considered effective and slightly increases the probability of early futility in scenarios were the treatment would be considered not effective.

**Probabilities of Trial Success and Futility - Symptomatic Longitudinal Profile**						
**Scenario**		**Mean N**	**Pr(Stop Early Success)**	**Pr(Stop Early Futility)**	**Pr(Go To Trial Completion)**	**Pr (Success)**
**1**	Null	701.6	0.074	0.439	0.486	0.082
**2**	Small Effect	715.3	0.247	0.127	0.626	0.328
**3**	BM Good	662.2	0.648	0.023	0.329	0.804
**4**	Q Low	545.4	0.981	0.003	0.016	0.995
**5**	Both Good	533.4	0.982	0.002	0.016	0.995
**6**	U-Shape Good	563.7	0.940	0.002	0.058	0.983
**7**	U-Shape Low	648.7	0.689	0.016	0.296	0.835
**8**	Small Increases	605.0	0.827	0.006	0.168	0.933
**9**	One Works	679.6	0.566	0.085	0.350	0.722
**10**	All 0.04	665.0	0.593	0.027	0.380	0.738
**11**	All 0.06	576.9	0.901	0.003	0.096	0.966
**12**	All 0.08	500.3	0.993	0.000	0.006	0.999
**13**	Medium Effect	703.6	0.381	0.059	0.559	0.501

13. APPENDIX FOR DROPOUT RATES

The two tables below summarize the operating characteristics of the design assuming lower dropout rates of 20% and 30% at 52 weeks respectively. These results assume the linear longitudinal profile. In general these results are similar to those that assume a 40% dropout rate at 52 weeks. Lower dropout results in a slightly lower mean sample size and an slightly increased probability of trial success in scenarios where the treatment would be considered effective.

**Probabilities of Trial Success and Futility – 20% Dropout at 52 Weeks**						
**Scenario**		**Mean N**	**Pr(Stop Early Success)**	**Pr(Stop Early Futility)**	**Pr(Go To Trial Completion)**	**Pr (Success)**
**1**	Null	681.4	0.095	0.496	0.410	0.10
**2**	Small Effect	689.9	0.287	0.147	0.566	0.353
**3**	BM Good	642.0	0.693	0.038	0.269	0.817
**4**	Q Low	548.5	0.975	0.016	0.009	0.982
**5**	Both Good	536.4	0.985	0.007	0.008	0.993
**6**	U-Shape Good	560.6	0.950	0.009	0.041	0.984
**7**	U-Shape Low	632.8	0.714	0.029	0.258	0.846
**8**	Small Increases	593.3	0.863	0.015	0.122	0.944
**9**	One Works	662.6	0.611	0.099	0.291	0.760
**10**	All 0.04	645.1	0.632	0.037	0.332	0.768
**11**	All 0.06	569.9	0.914	0.011	0.075	0.970
**12**	All 0.08	508.5	0.994	0.002	0.004	0.998
**13**	Medium Effect	678.0	0.413	0.087	0.500	0.538

Berry Consultants
Statistical Innovation

Probabilities of Trial Success and Futility – 30% Dropout at 52 Weeks						
**Scenario**		**Mean N**	**Pr(Stop Early Success)**	**Pr(Stop Early Futility)**	**Pr(Go To Trial Completion)**	**Pr (Success)**
**1**	Null	687.5	0.102	0.459	0.440	0.106
**2**	Small Effect	695.1	0.284	0.147	0.569	0.353
**3**	BM Good	649.0	0.668	0.043	0.289	0.809
**4**	Q Low	559.3	0.970	0.018	0.013	0.981
**5**	Both Good	551.1	0.984	0.006	0.01	0.993
**6**	U-Shape Good	569.5	0.941	0.010	0.049	0.982
**7**	U-Shape Low	636.5	0.698	0.031	0.271	0.827
**8**	Small Increases	605.7	0.842	0.018	0.140	0.935
**9**	One Works	666.8	0.582	0.105	0.313	0.732
**10**	All 0.04	646.5	0.617	0.046	0.337	0.757
**11**	All 0.06	576.9	0.905	0.015	0.080	0.963
**12**	All 0.08	519.5	0.992	0.004	0.004	0.996
**13**	Medium Effect	681.2	0.409	0.094	0.497	0.528

APPENDIX 7
